# Comparison of Amitriptyline and US Food and Drug Administration–Approved Treatments for Fibromyalgia: A Systematic Review and Network Meta-analysis

**DOI:** 10.1001/jamanetworkopen.2022.12939

**Published:** 2022-05-19

**Authors:** Hussein M. Farag, Ismaeel Yunusa, Hardik Goswami, Ihtisham Sultan, Joanne A. Doucette, Tewodros Eguale

**Affiliations:** 1 Department of Pharmaceutical Economics and Policy, Massachusetts College of Pharmacy and Health Sciences, Boston; 2 Department of Clinical Pharmacy and Outcomes Sciences, College of Pharmacy, University of South Carolina, Colombia; 3 Biostatistics and Research Decision Sciences and Health Economics and Decision Sciences, Merck & Co, North Wales, Pennsylvania; 4 Health Economics and Outcomes Research Neuroscience, AbbVie, Cambridge, Massachusetts; 5 Department of Medicine, McGill University Health Centre, Montreal, Canada

## Abstract

**Importance:**

Amitriptyline is an established medication used off-label for the treatment of fibromyalgia, but pregabalin, duloxetine, and milnacipran are the only pharmacological agents approved by the US Food and Drug Administration (FDA) to treat fibromyalgia.

**Objective:**

To investigate the comparative effectiveness and acceptability associated with pharmacological treatment options for fibromyalgia.

**Data Sources:**

Searches of PubMed/MEDLINE, Cochrane Library, Embase, and Clinicaltrials.gov were conducted on November 20, 2018, and updated on July 29, 2020.

**Study Selection:**

Randomized clinical trials (RCTs) comparing amitriptyline or any FDA-approved doses of investigated drugs.

**Data Extraction and Synthesis:**

This study follows the Preferred Reporting Items for Systematic Reviews and Meta-analyses reporting guideline. Four independent reviewers extracted data using a standardized data extraction sheet and assessed quality of RCTs. A random-effects bayesian network meta-analysis (NMA) was conducted. Data were analyzed from August 2020 to January 2021.

**Main Outcomes and Measures:**

Comparative effectiveness and acceptability (defined as discontinuation of treatment owing to adverse drug reactions) associated with amitriptyline (off-label), pregabalin, duloxetine, and milnacipran (on-label) in reducing fibromyalgia symptoms. The following doses were compared: 60-mg and 120-mg duloxetine; 150-mg, 300-mg, 450-mg, and 600-mg pregabalin; 100-mg and 200-mg milnacipran; and amitriptyline. Effect sizes are reported as standardized mean differences (SMDs) for continuous outcomes and odds ratios (ORs) for dichotomous outcomes with 95% credible intervals (95% CrIs). Findings were considered statistically significant when the 95% CrI did not include the null value (0 for SMD and 1 for OR). Relative treatment ranking using the surface under the cumulative ranking curve (SUCRA) was also evaluated.

**Results:**

A total of 36 studies (11 930 patients) were included. The mean (SD) age of patients was 48.4 (10.4) years, and 11 261 patients (94.4%) were women. Compared with placebo, amitriptyline was associated with reduced sleep disturbances (SMD, −0.97; 95% CrI, −1.10 to −0.83), fatigue (SMD, −0.64; 95% CrI, −0.75 to −0.53), and improved quality of life (SMD, −0.80; 95% CrI, −0.94 to −0.65). Duloxetine 120 mg was associated with the highest improvement in pain (SMD, −0.33; 95% CrI, −0.36 to −0.30) and depression (SMD, −0.25; 95% CrI, −0.32 to −0.17) vs placebo. All treatments were associated with inferior acceptability (higher dropout rate) than placebo, except amitriptyline (OR, 0.78; 95% CrI, 0.31 to 1.66). According to the SUCRA-based relative ranking of treatments, duloxetine 120 mg was associated with higher efficacy for treating pain and depression, while amitriptyline was associated with higher efficacy for improving sleep, fatigue, and overall quality of life.

**Conclusions and Relevance:**

These findings suggest that clinicians should consider how treatments could be tailored to individual symptoms, weighing the benefits and acceptability, when prescribing medications to patients with fibromyalgia.

## Key Points

**Question:**

What pharmacological treatments for adults with fibromyalgia are associated with the highest efficacy and acceptability?

**Findings:**

In this systematic review and network meta-analysis of 36 randomized clinical trials (11 930 patients with fibromyalgia), duloxetine (120 mg) was associated with higher efficacy in treating pain and depression, while amitriptyline was associated with higher efficacy and acceptability in improving sleep, fatigue, and health-related quality of life outcomes.

**Meaning:**

These findings suggest that with the heterogeneity of fibromyalgia symptoms, pharmacological treatments should be tailored to individual symptoms, including pain, sleep problems, depressed mood, fatigue, and health-related quality of life.

## Teaser

This systematic review and network meta-analysis compares amitriptyline with Food and Drug Administration–approved treatments for fibromyalgia related to sleep, fatigue, and health-related quality of life outcomes.

## Introduction

Fibromyalgia is a common illness characterized by widespread chronic pain, physical exhaustion, cognitive difficulties, depressed mood, sleep problems, and deteriorated quality of life (QoL).^1^ In the general population, the prevalence of fibromyalgia symptoms ranges between 2% and 4%.^2^ The symptoms of fibromyalgia reduce health-related QoL, and pharmacological treatments can improve health outcomes.^1,2^

Three drugs are approved by the US Food and Drug Administration (FDA): the gabapentinoid pregabalin (approved in 2007) and serotonin and norepinephrine reuptake inhibitors (SNRIs) duloxetine (in 2008) and milnacipran (in 2009). Amitriptyline, a tricyclic antidepressant, is commonly used off-label for pain relief, fatigue, sleep disturbance, depression, and improving QoL for patients with fibromyalgia.^3^ Despite the well-established value of using amitriptyline for fibromyalgia, the off-label policy renders defining the true efficacy and acceptability profile of the drug ambiguous.^4^ The lack of head-to-head trials with FDA-approved treatments makes comparing the available treatments difficult. Notably, the 3 FDA-approved medications account for an estimated 70% of prescribed drugs for fibromyalgia treatment.^5^ A comparative evaluation of these FDA-approved medications with the most commonly used off-label treatment (amitriptyline) could guide clinicians in medical decision-making.

To our knowledge, no published studies have explicitly evaluated the comparative health outcomes of amitriptyline vs the FDA-approved drugs.^3^ Traditional pairwise meta-analysis, in which all included studies compare the same intervention with the same comparator, is not feasible to conduct because of the lack of direct comparisons between some treatments. Network meta-analysis (NMA) combines the direct and indirect sources of evidence associated with outcomes of a drug use, adding extra strength to the evidence.^6^ As such, it could be used to compare fibromyalgia treatments, circumventing the problems currently associated with their evaluation using the traditional pairwise meta-analysis approach. Hence, we performed an NMA of randomized clinical trials (RCTs) to evaluate the effectiveness and acceptability associated with amitriptyline and FDA-approved drugs for treating fibromyalgia.

## Methods

The reporting of this NMA follows the Preferred Reporting Items for Systematic Reviews and Meta-analyses (PRISMA) reporting guideline, and the PRISMA extension statement for Reporting of Systematic Reviews Incorporating Network Meta-analysis of health care interventions (PRISMA-NMA).^7,8^ The study is registered with PROSPERO, number CRD42018116204. First, we conducted a systematic review of the literature before conducting the NMA by pooling comparable studies that met our study’s eligibility criteria. An NMA was conducted rather than the traditional pairwise meta-analysis because it enables comparison of pooled estimates using direct and indirect sources of evidence.

### Literature Review

The MEDLINE/PubMed, Embase, Cochrane Library, and ClinicalTrials.gov databases were searched, from their inception until November 20, 2018, and updated on July 29, 2020. Key search terms included *fibromyalgia*, *pregabalin*, *duloxetine*, *milnacipran*, and *amitriptyline*. The study protocol and full search strategy are described in eAppendix 1 and eAppendix 2 in the Supplement. Reference lists of the selected articles were examined to ensure that all relevant articles were identified. Titles and abstracts were independently screened by 4 investigators (H.M.F., H.G., I.Y., and I.S.), and potentially relevant articles were selected for full-text screening. Any disagreement was resolved by consultation with a fifth investigator (T.E.). The study protocol and changes made to the protocol are provided eAppendix 3 in the Supplement.

### Study Selection

Double-blind RCTs comparing the off-label use of amitriptyline and FDA-approved doses of pregabalin, duloxetine, or milnacipran head-to-head or with placebo in adults (aged ≥18 years) with fibromyalgia were included, according to the post– and pre–American College of Rheumatology (ACR) criteria for diagnosing fibromyalgia.^9,10,11,12,13,14^ Studies were excluded if they were not RCTs, used other comparators (such as non-FDA approved doses of pregabalin, duloxetine, and milnacipran, intravenous lidocaine combined with amitriptyline, growth hormone, desvenlafaxine, all opioids, phenytoin, fluoxetine, paroxetine, cyclobenzaprine, and clonazepam), were published in languages other than English, involved nonhuman participants, or had fewer than 5 participants in any treatment group.

### Data Extraction and Outcome Measures

Four investigators (H.M.F., H.G., I.Y., and I.S.) independently extracted the data using the a priori standardized data extraction sheet. Outcomes included were pain, sleep problems, depression, fatigue, QoL, and acceptability (defined as discontinuations associated with adverse drug reactions). The hierarchy of tools for patient-reported outcomes assessment is shown in eTable 1 in the Supplement.

All trials were independently graded for validity by the same 4 investigators using the Jadad scale, which scores randomization, double-blinding, and patient withdrawals, giving an aggregate score for each trial (range, 0-5, with 0 indicating the weakest and 5 the strongest).^15^

### Risk of Bias Assessment

Risk of bias was assessed by 2 investigators (I.Y. and H.M.F.) using the Cochrane Risk of Bias Tool.^16^ Each study was classified as having low, medium, or high risk of bias.

### Assessment of Clinical Assumptions

Transitivity is the distribution of patient and study characteristics that are potential modifiers of treatment outcomes and must be sufficiently similar across trials before an indirect comparison. It is a fundamental assumption underlying NMA.^17^ The credibility of transitivity in the data was evaluated by qualitatively assessing the distribution of the potential modifiers across the different direct comparisons.^18^

### Statistical Analysis

We performed an NMA for each outcome using a bayesian multiple treatment comparison with random effects. Noninformative (vague) priors (mean = 0; variance = 10000) were used for all parameters to render them a priori independent, and to ensure the results were primarily driven by the data.^19,20^ All eligible trials and subgroups, excluding trials that did not report the effect estimates of the interventions, were analyzed. The summary odds ratios (ORs) for the acceptability (dichotomous) outcome and standardized mean differences (SMDs) for the pain, sleep problems, depression, fatigue, and QoL (continuous) outcomes were determined.^6^ Findings were considered statistically significant when the 95% credible interval (CrI) did not include the null value (0 for SMD and 1 for OR). For clinical interpretation, Cohen *d* for effect size was used; an SMD less than 0.40 was a small difference between the experimental and control groups; 0.40 to 0.70, a moderate difference; and greater than 0.70, a large difference.^21^ When no variability measures were reported, imputation of the maximum SD from another study using the same measurement scale was performed.^22^ When studies did not report mean change, these values were calculated as the arithmetic difference between baseline and follow-up.

In this NMA, group-level data were used; the binomial likelihood was used for dichotomous and the normal likelihood for continuous outcomes.^23^ A random-effects model was computed using the Markov chain Monte Carlo (MCMC) methods with Gibbs sampling based on simulations of 50 000 iterations of 3 chains.^24,25^ To avoid the burn-in period, the first 10 000 iterations were rejected.^26^

The restricted maximum likelihood estimation method was used to estimate the heterogeneity, assuming a common estimate for heterogeneity variance among different comparisons for each outcome. Consistency was evaluated by examining the agreement between direct and indirect estimates in all closed loops and by assuming loop-specific heterogeneity using the loop-specific approach.^27^ To assess the consistency of the evidence, a node-splitting analysis was also conducted for each comparison in the treatment network that had both direct and indirect sources of evidence. In this approach, 1 of the treatment comparisons is split into a parameter for both direct and indirect evidence to determine if they agree.^28^

Rank probabilities were summarized using the surface under the cumulative ranking (SUCRA) curve and with a rankogram plot, considering the location and all the relative treatment effects.^29^ The SUCRA value would be 0 when a treatment is certain to be the worst and 1 when it is certain to be the best. A random-effects NMA within a bayesian framework using MCMC was performed using WinBUGS software, version 1.4.3 (MRC Biostatistics Unit).^30^ The statistical evaluation of inconsistency and production of network graphs and summary figures were conducted using network package in the Stata statistical software, version 15.1 (StataCorp).^31^ Data were analyzed from August 2020 to January 2021.

To evaluate whether small studies tended to yield different results, comparison-adjusted funnel plots were evaluated for each outcome.^32^ Sensitivity analyses were conducted in which studies with a sample size of 100 participants or fewer were excluded, to assess the robustness of the findings.^33^

## Results

### Characteristics and Risk of Bias of the Included Studies

**Figure 1.**
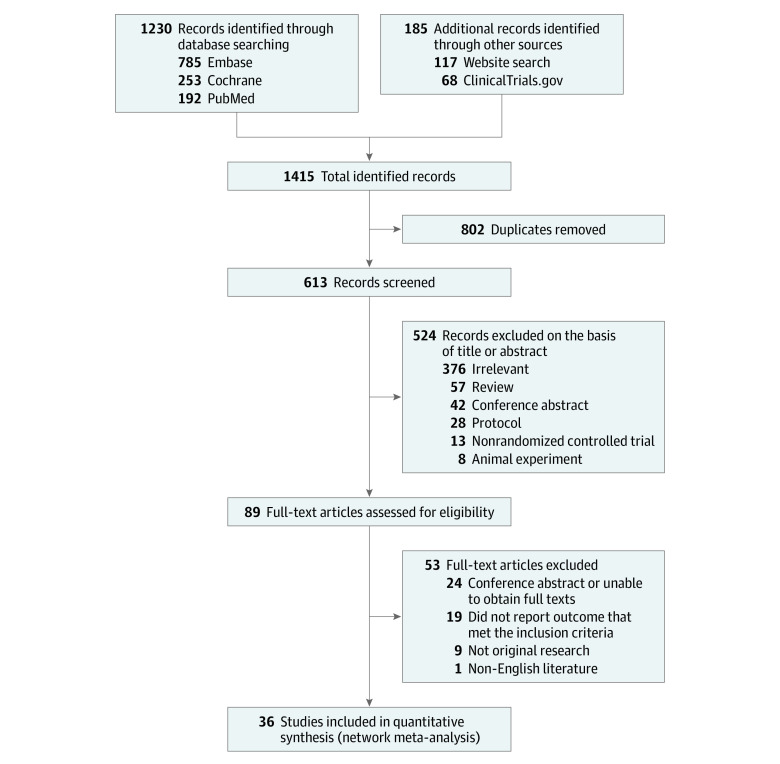
Study Selection Flowchart

**Figure 2.**
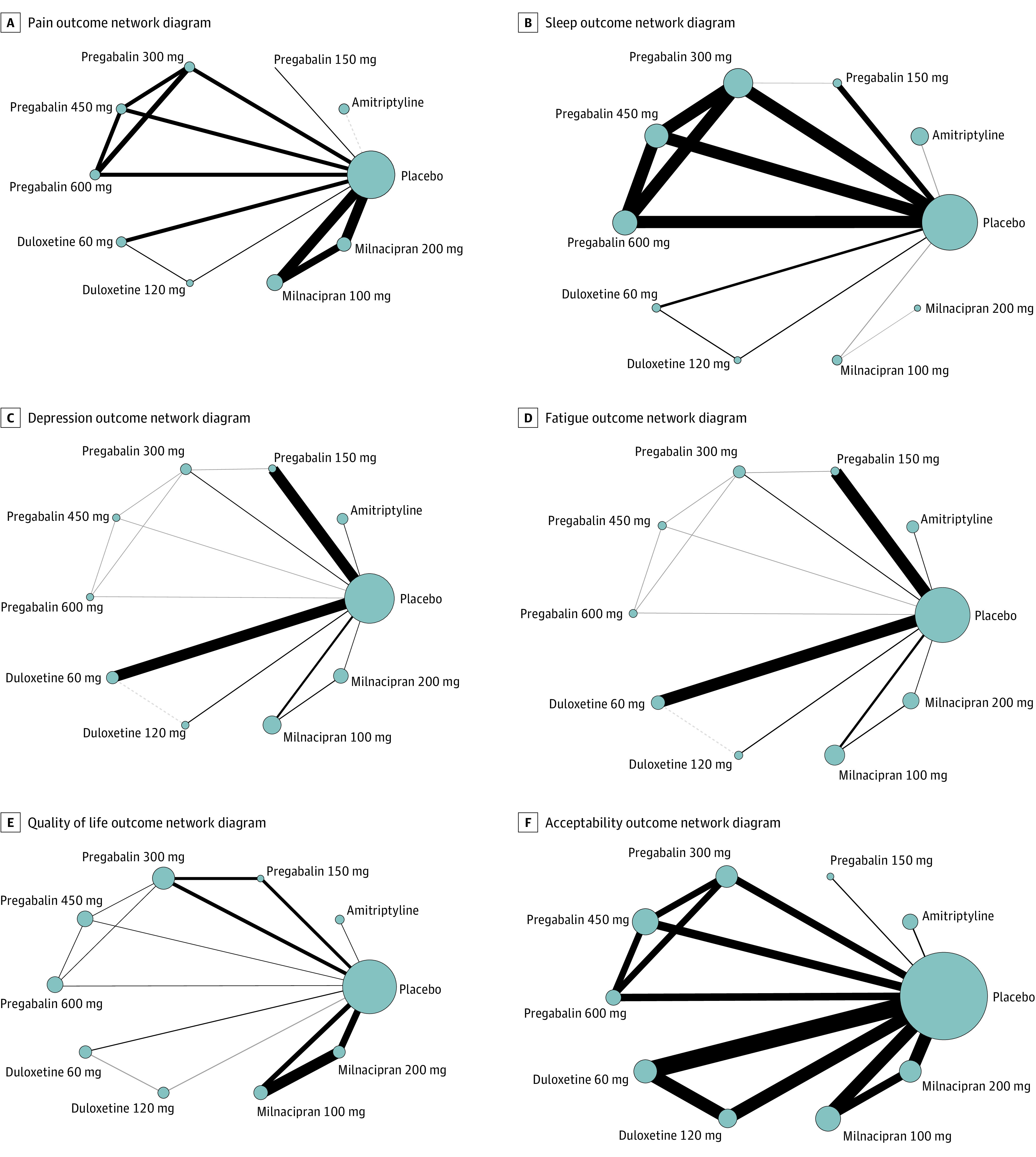
Network Diagrams Network diagrams showing fibromyalgia treatment comparisons in clinical trials with respect to the number of studies and sample sizes. The width of the line is proportional to the number of trials directly comparing each pair of treatments, and the size of each node is proportional to the sample size of randomized participants.

The literature search retrieved 1415 records; of these, 36 RCTs with 11 930 participants were included (Figure 1). The median (range) follow-up was 12 weeks (4-52). A total of 30 studies had a parallel design,^34,35,36,37,38,39,40,41,42,43,44,45,46,47,48,49,50,51,52,53,54,55,56,57,58,59,60,61,62,63,64,65,66^ whereas 3 studies had a crossover design.^67,68,69^ There were 33 studies that used the ACR 1990 criteria for the classification and diagnosis of fibromyalgia,^37,38,39,40,41,42,43,44,45,46,47,48,49,50,51,52,53,54,55,56,57,58,59,60,61,62,63,64,65,66,67,68,69^ 2 studies used Yunus criteria,^35,36^ and 1 study used the Smyth criteria (eTable 2 in the Supplement).^34^ The risk of bias assessment is reported in eTable 3 in the Supplement. Network diagrams for eligible comparisons for the outcomes are shown in Figure 2; eFigure 1 in the Supplement presents the network plots weighted by the risk of bias.

We found 10 clinical trials that evaluated amitriptyline,^34,35,36,37,38,39,40,41,42,67^ 11 trials that evaluated milnacipran,^57,58,59,60,61,62,63,64,65,66,69^ 8 trials that evaluated duloxetine,^43,44,45,46,47,48,49,50^ and 7 trials that evaluated pregabalin (eTable 2 in the Supplement).^51,52,53,54,55,56,68^

### Evaluation of Clinical Assumptions

The mean (SD) age of participants was 48.4 (10.4) years; 11 261 participants were women (94.4%). The distribution of age, sex, and fibromyalgia diagnosis was comparable across studies. Hence, the transitivity assumption was plausible (eTable 2 in the Supplement).

### Evaluation of Statistical Inconsistency

The loop-specific approach did not suggest any inconsistency between closed loops, except in the placebo–milnacipran 100 mg–milnacipran 200 mg loop for acceptability. Furthermore, the node-splitting approach did not suggest the presence of statistical inconsistency for any outcome (eTable 4 in the Supplement).

### Outcomes

#### Pain

A total of 35 trials assessed pain (11 423 patients).^34,35,36,37,38,39,41,42,43,44,45,46,47,48,49,50,51,52,53,54,55,56,57,58,59,60,61,62,63,64,65,66,67,68,69^ Of these, the Visual Analogue Scale (VAS) was used in 18 trials^35,36,38,39,42,51,52,55,57,58,59,60,62,63,64,65,66,67^; Brief Pain Inventory, 9 trials^44,45,46,47,48,49,50,61,69^; Numeric Rating Scale, 7 trials^34,37,41,53,54,56,68^; and Fibromyalgia Impact Questionnaire (FIQ), 1 trial^43^ (eTable 1 and eTable 2 in the Supplement).

Compared with placebo, duloxetine 120 mg was associated with the highest pain reduction (SMD, −0.33; 95% CrI, −0.36 to −0.30), followed by pregabalin 450 mg (SMD, −0.30; 95% CrI, −0.32 to −0.27). Milnacipran 100 mg was associated with the lowest reduction in pain (SMD, −0.17; 95% CrI, −0.20 to −0.15). According to SUCRA, duloxetine 120 mg (99.1%) and pregabalin 450 mg (86.8%) were associated with the highest probability of effectiveness for fibromyalgia pain (eTable 5 and eFigure 2 in the Supplement).

#### Sleep

A total of 16 trials^35,36,38,39,42,44,50,51,53,54,55,56,57,63,67,69^(4452 patients) assessed sleep. Of these, 6^35,36,38,39,42,67^ used VAS, 3^44,50,56^ used Brief Pain Inventory, 3^51,55,63^ used the Medical Outcomes Study Sleep Scale (MOS), 2^53,54^ used Numeric Rating Scale, 1^57^ used the Jenkins Scale, and 1^69^ used the Sleep Quality Scale.

Although all the treatments, except milnacipran 200 mg, were associated with reduced sleep problems, amitriptyline was associated with the highest improvement compared with placebo (SMD, −0.97; 95% CrI, −1.10 to −0.83), followed by pregabalin 600 mg (SMD, −0.60; 95% CrI, −0.67 to −0.54). Duloxetine 60 mg was associated with the least improvement (SMD, −0.21; 95% CrI, −0.30 to −0.13). According to SUCRA, amitriptyline (98.3%) and pregabalin 600 mg (82%) were associated with the highest probability of effectiveness on sleep (eTable 6 and eFigure 3 in the Supplement).

#### Depression

A total of 19 trials^37,42,43,44,45,46,48,49,50,51,52,53,56,59,61,62,63,64,68^ (8138 patients) evaluated depression in fibromyalgia. Of these, 8^37,48,49,50,59,61,62,63^ used Beck Depression Inventory, 5^43,44,45,46,52^ used Hamilton Depression Rating Scale, 3^51,53,68^ used Hospital Anxiety and Depression Scale, 2^42,64^ used VAS, and 1^56^ used FIQ.

Compared with placebo, duloxetine 120 mg (SMD, −0.25; 95% CrI, −0.32 to −0.17), duloxetine 60 mg (SMD, −0.24; 95% CrI, −0.27 to −0.20), pregabalin 600 mg (SMD, −0.23; 95% CrI, −0.28 to −0.17), pregabalin 300 mg (SMD, −0.22; 95% CrI, −0.26 to −0.19), pregabalin 450 mg (SMD, −0.14; 95% CrI, −0.18 to −0.09), milnacipran 100 mg (SMD, −0.10; 95% CrI, −0.12 to −0.07), milnacipran 200 mg (SMD, −0.07; 95% CrI, −0.10 to −0.04), and pregabalin 150 mg (SMD, −0.04; 95% CrI, −0.07 to −0.02) were associated with improved depression. Amitriptyline was not significantly different from placebo. According to SUCRA, duloxetine 120 mg (88.4%), duloxetine 60 mg (85.9%), and pregabalin 600 mg (80.3%) were associated with the highest probability of effectiveness on depression (eTable 7 and eFigure 4 in the Supplement).

#### Fatigue

A total of 21 trials^35,38,39,42,43,45,46,48,50,51,53,56,59,60,61,62,63,64,66,67^ (8172 patients) evaluated fatigue. Of these, 9^45,46,48,59,60,61,62,63,66^ used Multidimensional Fatigue Inventory, 6^35,38,39,42,64,67^ used VAS, 3^43,50,56^ used FIQ, 2^51,53^ used Multidimensional Assessment of Fatigue Global Index, and 1^69^ used Fatigue Severity Scale.

All treatments were associated with improved fatigue; amitriptyline was associated with the greatest improvement (SMD, −0.64; 95% CrI, −0.75 to −0.53), followed by pregabalin 150 mg (SMD, −0.27; 95% CrI, −0.29 to −0.24), and pregabalin 600 mg (SMD, −0.25; 95% CrI, −0.36 to −0.14). Milnacipran 100 mg (SMD, −0.10; 95% CrI, −0.14 to −0.05) and duloxetine 120 mg (SMD, −0.12; 95% CrI, −0.16 to −0.08) were associated with the least improvement in fatigue. According to SUCRA, amitriptyline (100%) and pregabalin 150 mg (83.8%) were associated with the highest probability of effectiveness on fatigue (eTable 8 and eFigure 5 in the Supplement).

#### Quality of Life

A total of 25 trials^37,38,39,40,42,43,44,45,46,47,49,50,51,53,54,55,56,59,60,61,62,63,66,68,69^ (10 219 patients) evaluated QoL. Of these, 18^40,42,43,44,46,47,50,53,54,55,56,60,61,62,63,66,68,69^ used FIQ, 4^45,49,51,59^ used the Short Form 36 Health Survey, 1^37^ used Sickness Impact Profile, 1^38^ used patient global evaluation of fibromyalgia symptoms by VAS, and 1^39^ used the General Health Questionnaire.

Compared with placebo, amitriptyline (SMD, −0.80; 95% CrI, −0.94 to −0.65), duloxetine 120 mg (SMD, −0.39; 95% CrI, −0.55 to −0.23), duloxetine 60 mg (SMD, −0.22; 95% CrI, −0.35 to −0.09), pregabalin 450 mg (SMD, −0.18; 95% CrI, −0.29 to −0.06), pregabalin 300 mg (SMD, −0.14; 95% CrI, −0.23 to −0.06), and pregabalin 150 mg (SMD, −0.12; 95% CrI, −0.23 to −0.02) were associated with improved QoL. Pregabalin 600 mg, milnacipran 100 mg, and milnacipran 200 mg were not associated with improved QoL. According to SUCRA, amitriptyline (100%) and duloxetine 120 mg (88.4%) were associated with the highest probability of effectiveness on QoL (eTable 9 and eFigure 6 in the Supplement).

#### Acceptability

There were 26 trials^35,37,38,39,40,43,44,45,46,49,50,51,53,55,56,57,58,59,60,61,62,63,65,66,68,69^ (9833 patients) that evaluated discontinuations associated with adverse drug reactions. Amitriptyline did not differ from placebo (OR, 0.78; 95% CrI, 0.31-1.66), while all the other treatments were associated with lower acceptability. According to SUCRA, amitriptyline (93.2%) was associated with the highest probability of being the most acceptable (eTable 10, eTable 11, and eFigure 7 in the Supplement).

### Simultaneous Ranking of the Interventions

**Figure 3.**
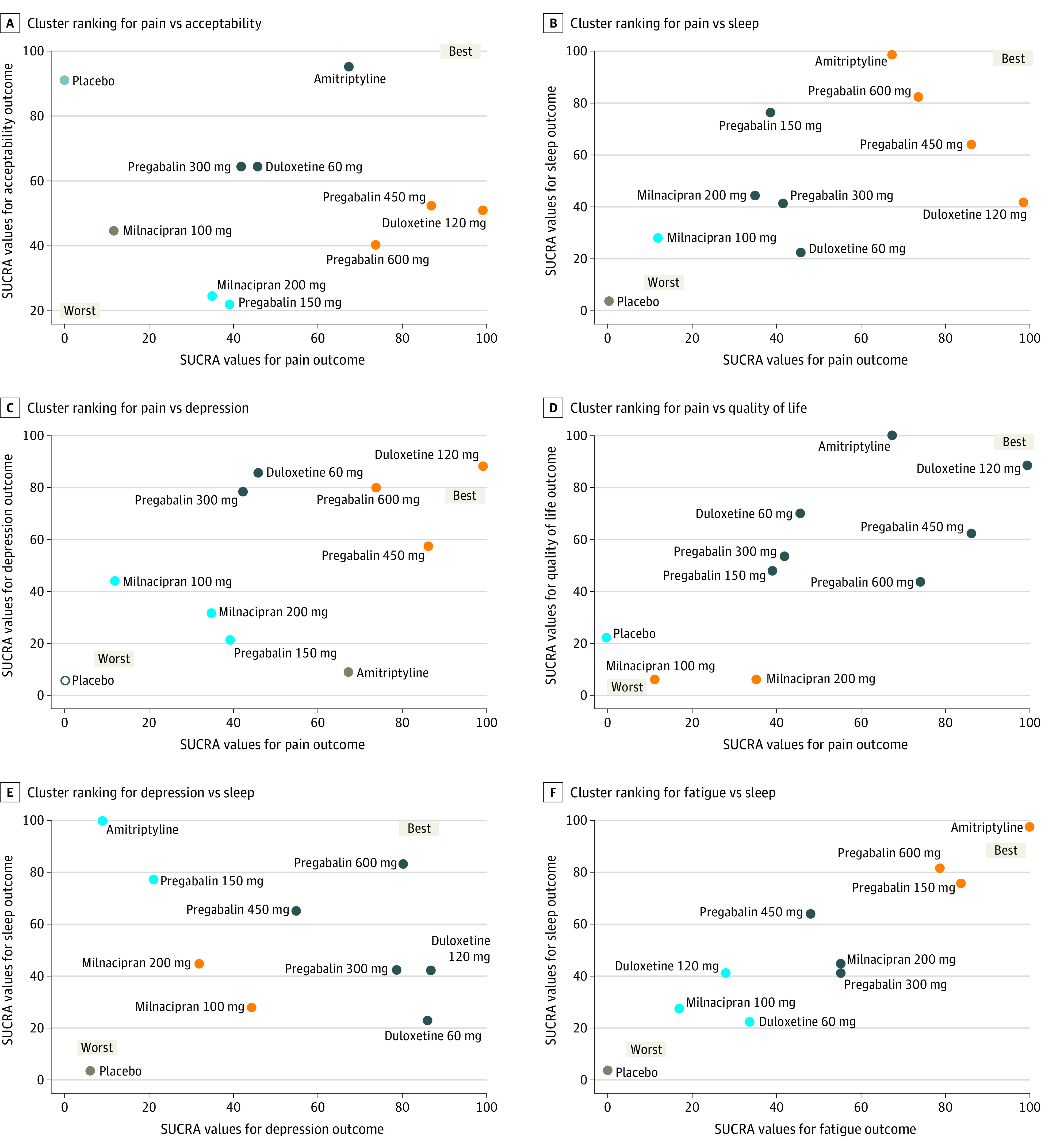
Cluster Ranking Plot for Relative Effectiveness and Acceptability SUCRA indicates surface under the cumulative ranking. Each plot shows SUCRA values on a scale of 0% to 100% for 2 outcomes. Drugs with the same color belong to a similar effectiveness/acceptability profile. The upper right quadrant represents the more favorable interventions on the joint outcomes; lower right quadrant, more favorable on the horizontal axis outcome but less on the vertical axis outcome; lower left quadrant, less favorable on both outcomes; the upper left quadrant, more favorable on the vertical axis outcome but less on the horizontal axis outcome.

Figure 3 presents SUCRA for the following outcome comparisons: pain vs acceptability; pain vs sleep; pain vs depression; pain vs QoL; depression vs sleep; fatigue vs sleep. The rest of the simultaneous ranking of interventions are presented in eFigure 8 in the Supplement.

### Additional Analyses

Comparison-adjusted funnel plots suggested a publication bias for pain (1 trial each for milnacipran 100 mg and 200 mg with biased estimates favoring the drugs) (eFigure 2F in the Supplement), and QoL (1 trial for duloxetine 60 mg and 1 for milnacipran 200 mg with biased outcomes against the drugs) (eFigure 6F in the Supplement). There was no evidence of publication bias for sleep (eFigure 3F in the Supplement), depression (eFigure 4F in the Supplement), fatigue (eFigure 5F in the Supplement), or acceptability (eFigure 7D in the Supplement).

The results of the sensitivity analyses are presented in eFigure 9 and eTable 12 in the Supplement. In the sensitivity analysis, all treatments except amitriptyline and pregabalin 150 mg were associated with improvements in pain (SMD between −0.17 and −0.48) compared with placebo. All pregabalin doses were associated with improved sleep (SMD between −0.55 and −0.80). None of the included treatments were associated with better outcomes than placebo for depression. Pregabalin 150 mg, pregabalin 600 mg, duloxetine 60 mg, milnacipran 100 mg, and milnacipran 200 mg were associated with improved fatigue (SMD between −0.11 and −0.31). Pregabalin 300 mg, pregabalin 450 mg, duloxetine 60 mg, and duloxetine 120 mg were associated with improved QoL (SMD between −0.19 and −0.37).

## Discussion

This systematic review and NMA study of 36 double-blind randomized clinical trials, which included 11 930 patients, assessed the comparative effectiveness and acceptability associated with amitriptyline compared with FDA-approved treatments for reducing the symptoms of fibromyalgia in adults. The NMA found that off-label use of amitriptyline was associated with large improvement in sleep and QoL, a moderate improvement in fatigue, a small improvement in pain, and was not associated with improvement in depression compared with placebo. Duloxetine 120 mg was associated with improvment in all effectiveness outcomes, with the greatest improvements in pain and depression.

We also found that pregabalin 600 mg, 450 mg, and 150 mg were associated with a moderate improvement in sleep symptoms. Although pregabalin 600 mg was associated with improved QoL, pregabalin generally showed only a small improvements in the other measured symptoms. Milnacipran 100 mg was associated with small improvements in all outcomes except QoL; milnacipran 200 mg was associated with small reductions in pain, depression, and fatigue, but did not improve sleep and QoL outcomes. Pregabalin, duloxetine, and milnacipran were associated with worse acceptability than placebo, while the acceptability outcomes associated with amitriptyline did not significantly differ from placebo.

Most of the results from the SUCRA corroborate previous reviews in confirming the therapeutic outcomes associated with pregabalin, duloxetine, and milnacipran in the treatment of fibromyalgia.^70^ However, this NMA’s findings are consistent with a 2011 study by Hauser et al^70^ regarding the greater effectiveness associated with amitriptyline in reducing sleep disturbances, fatigue, and improving QoL compared with duloxetine. In addition, amitriptyline was associated with greater improvements in sleep, fatigue, and QoL than pregabalin. Our results are similar to a 2015 study by Moore et al^3^ in the acceptability of amitriptyline compared with placebo. In contrast to a 2018 study by Cipriani et al^71^ that found amitriptyline to be the antidepressant associated with the most efficacy among patients with major depressive disorder, this NMA found that amitriptyline was not associated with reducing fibromyalgia’s depressive symptoms. This difference may be explained by the pathophysiological causes of depression and fibromyalgia. In fibromyalgia, depression can be a direct result of pain, compounded by various comorbidities.^72^

Our study emphasizes the need for the pharmacological treatments to be selected and tailored to individual symptoms, acceptability, and adverse effect profiles of the drugs.^1^ Considering the dose-dependent adverse effects of all drugs, it is recommended to start at a low dose and increase slowly, if necessary.^70^

Unfortunately, pharmacological treatments will provide a modest effect for most patients. For that reason, nonpharmacological approaches that promote physical activity and coping skills should be recommended to all patients.^1^ Cognitive behavioral therapy, aerobic exercise, tai chi, hydrotherapy, mindfulness-based stress reduction, and multicomponent therapies have been associated with reducing fibromyalgia symptoms and can be recommended either alone or in conjunction with pharmacological treatment.^73^ A 2020 study by Smith et al^74^ showed variations in effect sizes from trials of pharmacological treatments to control chronic pain (including fibromyalgia pain) over time. However, this NMA used random-effects modeling and thus accounted for variations within and between all included studies.

The strength of this NMA includes a comprehensive search of the literature and retrieval of 36 eligible studies with a total of 11 930 participants. Given that off-label use of drugs without strong scientific evidence is associated with adverse health outcomes,^4^ this NMA adds to the literature regarding the evidence of effectiveness and acceptability of amitriptyline vs FDA-approved drugs. With a plethora of FDA-approved and off-label treatment options for patients with fibromyalgia, our NMA provides information that could guide clinicians and patients in making rational, evidence-based decisions while considering the risk-benefit profiles. Future studies may consider including other off-label treatment options that are not as common as amitriptyline.

### Limitations

This NMA has several limitations. First, fewer than 75% of trials included more than 100 patients per group, which may introduce bias due to small-study effects. Second, while this NMA might be used as guide for future drug development, the NMA did not include all the available pharmacological technologies, although the included treatments accounted for more than 70% of the fibromyalgia prescribed treatments.^5^ Third, the SUCRA curve was used to estimate a ranking probability of comparative effectiveness, but it has limitations, and the results should be interpreted with caution.

## Conclusions

The findings of this NMA support the therapeutic effectiveness associated with pregabalin, duloxetine, and milnacipran and suggest that the off-label use of amitriptyline was also associated with favorable efficacy and acceptability in the treatment of fibromyalgia. These findings suggest that for optimal health outcomes in patients with fibromyalgia, pharmacological treatments should be tailored toward individual symptoms. Furthermore, this NMA extends previous research by evaluating the comparative effectiveness and acceptability of amitriptyline vs FDA-approved drugs using a bayesian approach. Future studies are needed to include individual patient data in the NMA to identify specific individual characteristics that may influence the effectiveness and acceptability of fibromyalgia pharmacological drugs.

## Notes

Supplement. **eAppendix 1.** Study Protocol **eAppendix 2.** Search Strategy
eReferences
**eAppendix 3.** Changes Made to the Protocol **eTable 1.** Hierarchy of Tools for Patient-Reported Outcomes Assessment **eTable 2.** Study and Patient Characteristics **eTable 3.** Risk of Bias Assessment **eTable 4.** Assessments of Inconsistencies **eTable 5.** Pain Outcome: League Table **eTable 6.** Sleep Outcome: League Table **eTable 7.** Depression Outcome: League Table **eTable 8.** Fatigue Outcome: League Table **eTable 9.** Quality of Life Outcome: League Table **eTable 10.** Acceptability Outcome: League Table **eFigure 1.** Network Plots **eFigure 2.** Pain Outcome: Results **eFigure 3.** Sleep Outcome: Results **eFigure 4.** Depression Outcome: Results **eFigure 5.** Fatigue Outcome: Results **eFigure 6.** Quality of Life Outcome: Results **eFigure 7.** Acceptability Outcome: Results **eTable 11.** Acceptability Outcome: Direct Pairwise Comparisons (Estimates as Odds Ratios [ORs] and 95% Credible Intervals [95% CrI]) **eFigure 8.** Cluster Ranking Plots for Relative Effectiveness and Acceptability **eFigure 9.** Sensitivity Analysis Removing Studies With Small Sample Size for Each Outcome (Presented as Interval Plots) **eTable 12.** Sensitivity Analysis Removing Studies With Small Sample Size for Each Outcome (Presented as League Tables)
