# Supplementary material for: Comparison of Amitriptyline and US Food and Drug Administration–Approved Treatments for Fibromyalgia: A Systematic Review and Network Meta-analysis
Source: JAMA Netw Open. 2022 May 19;5(5):e2212939. doi: 10.1001/jamanetworkopen.2022.12939 (PMC9121190; doi:10.1001/jamanetworkopen.2022.12939)
Supplement: Supplement. — eAppendix 1. Study Protocol eAppendix 2. Search Strategy eReferences eAppendix 3. Changes Made to the Protocol eTable 1. Hierarchy of Tools for Patient-Reported Outcomes Assessment eTable 2. Study and Patient Characteristics eTable 3. Risk of Bias Assessment eTable 4. Assessments of Inconsistencies eTable 5. Pain Outcome: League Table eTable 6. Sleep Outcome: League Table eTable 7. Depression Outcome: League Table eTable 8. Fatigue Outcome: League Table eTable 9. Quality of Life Outcome: League Table eTable 10. Acceptability Outcome: League Table eFigure 1. Network Plots eFigure 2. Pain Outcome: Results eFigure 3. Sleep Outcome: Results eFigure 4. Depression Outcome: Results eFigure 5. Fatigue Outcome: Results eFigure 6. Quality of Life Outcome: Results eFigure 7. Acceptability Outcome: Results eTable 11. Acceptability Outcome: Direct Pairwise Comparisons (Estimates as Odds Ratios [ORs] and 95% Credible Intervals [95% CrI]) eFigure 8. Cluster Ranking Plots for Relative Effectiveness and Acceptability eFigure 9. Sensitivity Analysis Removing Studies With Small Sample Size for Each Outcome (Presented as Interval Plots) eTable 12. Sensitivity Analysis Removing Studies With Small Sample Size for Each Outcome (Presented as League Tables) [file jamanetwopen-e2212939-s001.pdf]

Supplemental Online Content

Farag HM, Yunusa I, Goswami H, Sultan I, Doucette JA, Eguale T. Comparison of amitriptyline and US Food and Drug Administration–approved treatments for fibromyalgia: a systematic review and network meta-analysis. *JAMA Netw Open*. 2022;5(5):e2212939. doi:10.1001/jamanetworkopen.2022.12939

eAppendix 1. Study Protocol
eAppendix 2. Search Strategy
eReferences
eAppendix 3. Changes Made to the Protocol
eTable 1. Hierarchy of Tools for Patient-Reported Outcomes Assessment
eTable 2. Study and Patient Characteristics
eTable 3. Risk of Bias Assessment
eTable 4. Assessments of Inconsistencies
eTable 5. Pain Outcome: League Table
eTable 6. Sleep Outcome: League Table
eTable 7. Depression Outcome: League Table
eTable 8. Fatigue Outcome: League Table
eTable 9. Quality of Life Outcome: League Table
eTable 10. Acceptability Outcome: League Table
eFigure 1. Network Plots
eFigure 2. Pain Outcome: Results
eFigure 3. Sleep Outcome: Results
eFigure 4. Depression Outcome: Results
eFigure 5. Fatigue Outcome: Results
eFigure 6. Quality of Life Outcome: Results
eFigure 7. Acceptability Outcome: Results
eTable 11. Acceptability Outcome: Direct Pairwise Comparisons (Estimates as Odds Ratios [ORs] and 95% Credible Intervals [95% CrI])

© 2022 Farag HM et al. *JAMA Network Open.*

eFigure 8. Cluster Ranking Plots for Relative Effectiveness and Acceptability
eFigure 9. Sensitivity Analysis Removing Studies With Small Sample Size for Each Outcome (Presented as Interval Plots)
eTable 12. Sensitivity Analysis Removing Studies With Small Sample Size for Each Outcome (Presented as League Tables)

This supplemental material has been provided by the authors to give readers additional information about their work.

© 2022 Farag HM et al. *JAMA Network Open.*

eAppendix 1. Study Protocol

The original study protocol was registered with PROSPERO (http://www.crd.york.ac.uk/PROSPERO/, Registration No. CRD42018116204)

Introduction

Fibromyalgia is chronic condition characterized by widespread pain, fatigue, sleep and mood problems, and its exact etiology is still unknown.[1-3] Its definition went through a drastic change over the last decades.[4] The American College of Rheumatology (ACR) defined fibromyalgia as requiring multiple tender points and chronic widespread pain in 1990.[5] In 2010, the ACR preliminary diagnostic criteria eliminated the tender point examination and relied on patient-reported somatic symptoms and cognitive difficulties.[6]

Over the last three decades, a considerable growth of publications on fibromyalgia covered a multitude of problems, among them the genetic pathophysiology and pain modulation mechanisms, resulting in an improved understanding and leading to improvement in therapy.[2,4,7-9]

In the past decade, guidelines for the management of fibromyalgia have been published by groups in North America, Europe and the Middle East.[10] The United States Food and Drug Administration has approved three pharmacological treatments for fibromyalgia, which are the anticonvulsant pregabalin, and the serotonin-noradrenaline reuptake inhibitors (SNRIs) duloxetine and milnacipran.[11]

Several studies have reported the efficacy of the tricyclic antidepressants compared with placebo in the treatment of FM. Amitriptyline is approved for fibromyalgia in Europe and the Middle East, and is recommended by experts in the context of a multidisciplinary strategy that includes both pharmacological and non-pharmacological therapies in the management of fibromyalgia.[4] However, amitriptyline is not approved by the US FDA for the treatment of fibromyalgia, although its off-label use for fibromyalgia as a first-line therapy is popular.[12]

© 2022 Farag HM et al. *JAMA Network Open.*

In conformance with the PRISMA guidelines for reporting systematic reviews, the purpose of our study is to contribute towards a better understanding, based on a network meta-analysis of double-blind randomized clinical trials of the efficacy of amitriptyline in comparison with FDA approved drugs pregabalin, duloxetine, milnacipran in the treatment of fibromyalgia. [13]

Inclusion/Exclusion criteria

Prospective network meta-analysis literature to be reviewed should include:

1. *Study Design*: Randomized controlled trials with double-blind assessment of fibromyalgia treatments. Observational cohort, case-control, case reports and reviews will be excluded
2. *Population*: Adult patients (³18 years of age) presenting with fibromyalgia as defined by the American College of Rheumatology (ACR) 1990, 2010 or 2016 criteria.[5,6,14] Studies with fewer than 5 participants in any treatment arm will be excluded
3. *Intervention*: Amitriptyline in any dose, by any route other than topical administration for the relief of fibromyalgia
4. *Comparator*: FDA approved doses of pregabalin 150mg, 300mg, and 450mg, and 600mg; duloxetine 60mg, and 120mg, or milnacipran 100 mg, and 200 mg for the treatment of fibromyalgia
5. *Outcomes*: **eTable 3** provides a hierarchy of tools for patient-reported outcomes assessment

Search Strategy

© 2022 Farag HM et al. *JAMA Network Open.*

We will use PubMed/Medline, Embase, and Cochrane databases as sources of our literature screening. Our search will consider literature published about “Amitriptyline”, “Pregabalin”, “Duloxetine” or “Milnacipran” in “Fibromyalgia” from database inception November 20, 2018, and updated on July 29, 2020. Using Medical Subject Headings “MESH” terms, searches will be limited to English and human. Appropriate MESH terms, and title/abstracts keywords relevant to our search topic will be used in the Medline search. Also, synonyms of MESH search terms relevant to our topic will be included. In addition to the articles retrieved from the above databases, we will search manually for extra supplement of literature indicated as references in the retrieved articles. When needed, we will explore and get advice from Dr. Tewodros Eguale as the project supervisor, as well as a team of medical experts at the Brigham and Women’s Hospital in Boston who are knowledgeable on our search topic. Selection Process will be according to the Preferred Reporting Items for Systematic Reviews and Meta-analysis (PRISMA) guidelines (**eFigure 1**). 13

© 2022 Farag HM et al. *JAMA Network Open.*

eAppendix 2. Search strategy

eAppendix 2A. Final search strategy for Pubmed

Our PubMed/MEDLINE search strategy using MeSH terms and text Words:

(Random*[tw] OR RCT [tw]) AND
("Fibromyalgia"[Mesh] OR Fibromyalgia*[tw] OR Muscular Rheumatism[tw] OR Fibrositis[tw] OR Diffuse Myofascial Pain Syndrome[tw])
AND
(("Pregabalin"[Mesh] OR Pregabalin[tw] OR Lyrica[tw] OR "Duloxetine Hydrochloride"[Mesh] OR Duloxetine Hydrochloride[tw] OR Duloxetine HCL[tw] OR Duloxetine[tw] OR Cymbalta[tw] OR Milnacipran[tw] OR midalcipran[tw] OR Ixel[tw] OR Fetzima[tw] OR Savella[tw] OR Levomilnacipran[tw] OR milnacipran hydrochloride[tw]) OR ("Amitriptyline"[Mesh] OR Amitrip[tw] OR Amitriptylin[tw] OR Amitriptyline[tw] OR Amitrol[tw] OR Apo-Amitriptyline[tw] OR Damilen[tw] OR Domical[tw] OR Laroxyl[tw] OR Lentizol[tw] OR Saroten[tw] OR Sarotex[tw] OR Triptafen[tw] OR Endep[tw] OR Tryptizol[tw] OR Elavil[tw] OR Tryptanol[tw]))
AND
("Pain"[Mesh] OR Pain*[tw] OR Ache*[tw] OR Physical Suffering[tw] OR "Chronic Pain" [Mesh] OR Chronic Pain*[tw] OR “Musculoskeletal Pain”[Mesh] OR Musculoskeletal Pain*[tw] OR “Neuralgia”[Mesh] OR Neuralgia*[tw] OR Neuropathic Pain[tw] OR Neurodynia[tw] OR Neuralgia[tw] OR Iliohypogastric Nerve Neuralgia*[tw] OR Ilioinguinal Neuralgia[tw] “Nociceptive Pain”[Mesh] OR Nociceptive Pain*[tw] OR "Sleep"[Mesh] Sleep*[tw] OR "Quality of Life"[Mesh] OR Quality of Life[tw] OR Life Quality[tw] OR Health-Related Quality of life[tw] OR HRQOL[tw] OR “treatment outcome”[mesh] or Treatment Outcome*[tw] OR Clinical Effectiveness[tw] OR Patient-Relevant Outcome*[tw] OR Clinical Efficacy[tw] OR Treatment Effectiveness[tw] OR Treatment Efficacy[tw] OR Rehabilitation Outcome[tw] OR “visual analog scale”[Mesh] OR visual analog scale[tw] OR “Pain Measurement”[Mesh] Or Pain Measurements[tw] OR Pain assessment[tw] OR Analgesia Test[tw] OR McGill Pain scale[tw] OR McGill Pain Questionnaire[tw] OR Complication rates[tw])

Results: 192 hits, November 20, 2018, and updated on July 29, 2020

© 2022 Farag HM et al. *JAMA Network Open.*

eAppendix 2B. Final search strategy for Embase

The following subheading and Emtree keywords will be used in our Embase search:

(exp randomized controlled trial/ or (random* or RCT).tw.)
AND
(exp fibromyalgia/ OR (fibrositic nodule OR fibrositis OR myalgia* OR fibrositides OR diffuse myofascial pain syndrome OR fibromyalgia*).tw.)
AND
(exp pregabalin/ OR exp duloxetine hydrochloride/ OR exp milnacipran/ OR exp amitriptyline/ OR pregabalin.tw. OR lyrica.tw. OR cymbalta.tw. OR midalcipran.tw. OR ixel.tw. OR fetzima.tw. OR savella.tw. OR levomilnacipran.tw. OR tryptine.tw. OR amineurin.tw. OR amitrip.tw. OR amitriptyline.tw. OR amitrol.tw. OR anapsique.tw. OR damilen.tw. OR domical.tw. OR laroxyl.tw. OR lentizol.tw. OR novoprotect.tw. OR saroten.tw. OR sarotex.tw. OR syneudon.tw. OR triptafen.tw. OR endep.tw. OR tryptizol.tw. OR elavil.tw. OR tryptanol.tw.)
AND
(exp pain/ OR pain.tw. OR ache.tw. OR aches.tw. OR allodynia.tw. OR exp sleep/ OR cystalgia.tw. OR hyperalgesia.tw. OR myalgia.tw. OR neuralgia.tw. OR sleep.tw. OR somnolence.tw. OR quality of life.tw. OR life quality.tw. OR health-related quality of life.tw. OR hrqol.tw. OR exp short form 36/ OR short form 36 health survey.tw. OR exp efficacy/ OR exp therapy/ OR therapeutics.tw. OR treatment outcome.tw. OR clinical effectiveness.tw. OR patient-relevant outcome.tw. OR clinical efficacy.tw. OR treatment effectiveness.tw. OR rehabilitation outcome.tw. OR complication rate*.tw.)

Results: 785 hits, November 20, 2018, and updated on July 29, 2020

© 2022 Farag HM et al. *JAMA Network Open.*

eAppendix 2C. Final search strategy for Cochrane Central Register of Controlled Trials

The following search terms will be used in our Cochrane library search:

MeSH descriptor: [Randomized Controlled Trial] explode all trees OR (Random* OR RCT):ti,ab,kw
AND
MeSH descriptor: [Fibromyalgia] explode all trees OR (Fibromyalgia* OR “Muscular Rheumatism” OR Fibrositis OR “Diffuse Myofascial Pain Syndrome”):ti,ab,kw
AND
MeSH descriptor: [Pregabalin] explode all trees OR MeSH descriptor: [Duloxetine Hydrochloride] explode all trees OR MeSH descriptor: [Amitriptyline] explode all trees OR (Pregabalin OR Lyrica OR Duloxetine OR Cymbalta OR Milnacipran OR midalcipran OR Ixel OR Fetzima OR Savella OR Levomilnacipran OR Amitrip OR Amitriptylin OR Amitriptyline OR Amitrol OR Apo-Amitriptyline OR Damilen OR Domical OR Laroxyl OR Lentizol OR Saroten OR Sarotex OR Triptafen OR Endep OR Tryptizol OR Elavil OR Tryptanol):ti,ab,kw
AND
MeSH descriptor: [Pain] explode all trees OR MeSH descriptor: [Chronic Pain] explode all trees OR MeSH descriptor: [Musculoskeletal Pain] explode all trees OR MeSH descriptor: [Neuralgia] explode all trees OR MeSH descriptor: [Nociceptive Pain] explode all trees OR MeSH descriptor: [Sleep] explode all trees OR MeSH descriptor: [Quality of Life] explode all trees OR MeSH descriptor: [Treatment Outcome] explode all trees OR MeSH descriptor: [Visual Analog Scale] explode all trees OR MeSH descriptor: [Pain Measurement] explode all trees OR (Pain* OR Ache* OR “Physical Suffering” OR Neuralgia* OR Neurodynia OR Sleep OR “Quality of Life” OR “Life Quality” OR “Health-Related Quality of life” OR HRQOL OR “Treatment Outcome” OR “Clinical Effectiveness” OR “Patient-Relevant Outcome” OR “Clinical Efficacy” OR “Treatment Effectiveness” OR “Treatment Efficacy” OR “Rehabilitation Outcome” OR “visual analog scale” Or “Pain Measurements” OR “Pain assessment” OR “Analgesia Test” OR “McGill Pain scale” OR “McGill Pain Questionnaire” OR “Complication rates”):ti,ab,kw

Results: 254 hits, November 20, 2018, and updated on July 29, 2020

© 2022 Farag HM et al. *JAMA Network Open.*

With consensus will of all the three reviewers, a systematic documentation will follow outlining criteria agreed upon for inclusion/exclusion and for further review of the literature for meta-analysis. Following collection of the references from the search results and an initial removal of duplicate references, all literature for review will be imported into an Excel sheet by HF and HG for further analysis to exclude any additional duplicates. After removing duplicates, the studies will be first screened, following the inclusion/exclusion criteria, through title and abstract screening (Level 1) and then full text screening (Level 2).

IU, IS, HF, HG, and JD will independently review the literature in pairs to ascertain the appropriateness of each citation. Should a disagreement emerge as a result of cross checking each other's reviewed literature, a consensus will have to be reached by discussion between the independent reviewers or by the intervention of a third reviewer.

A kappa score will be calculated for the level 2 screening. The study selection process will be reflected in the details of Figure 1. The resulting selected full text studies will go on to the data extraction phase.

Data Extraction

A standardized extraction form to be used will be designed using Excel software and will be approved by Dr. Teddy Eguale, the project supervisor. Articles included for potential meta-analysis review, will be divided amongst IU, IS, HF, and HG. The data will be extracted by the 4 independent reviewers, working in pairs. Any disagreements will be resolved by a third investigator. The variables that will be selected and extracted are listed below:

© 2022 Farag HM et al. *JAMA Network Open.*

Data Extraction Form

General Information	Study Characteristics
- Extractors' Initials, date of data extraction<br>- Study ID, lead author, article title, citation, type of publication, publication year, country of origin, source of funding	- Aim/objectives, study design<br>- Inclusion/exclusion criteria<br>- Details of recruitment procedures (i.e. randomization, blinding)<br>- Database used to collect patient's data
**Participant Characteristics**	**Intervention/Exposure and Setting**
- Baseline characteristics (Age, sex, ethnicity, disease characteristics (primary or secondary fibromyalgia, co-morbidities)<br>- Description of intervention<br>- # of patients in treatment and control group<br>- # eligible/enrolled/randomized; mean/median	- Setting intervention delivered<br>- Description of intervention (name, dose, schedule)
**Outcomes Measure**	

© 2022 Farag HM et al. *JAMA Network Open.*

- Definition of outcome in study

- For each intervention and control group

- **Number of participants enrolled in intervention/control grp**
- **Number of participants included in the analysis**
- **Number of withdrawals, exclusions, lost to follow-up**
- **summary of outcome data (see eTable 3 for the hierarchy of patient reported outcomes tools):**

1. Pain: Participant-reported pain relief using Visual analog scale (VAS), Fibromyalgia Impact Questionnaire (FIQ), Revised Fibromyalgia Impact Questionnaire (RFIQ), Numeric Rating Scale (NRS) or Brief Pain Inventory (BPI)
2. Sleep: VAS, FIQ, RFIQ or NRS
3. Depressed mood: VAS, FIQ or RFIQ
4. Health related quality of life (HRQOL): FIQ total score, 36-Item Short Form Survey (SF-36) or EQ-5D form
5. Acceptability: withdrawal due to adverse events (and the reason, if possible)

- Statistical Analysis used in the study (intention to treat, per protocol)

- results of study analysis (SMD, OR, 95% CrI).

© 2022 Farag HM et al. *JAMA Network Open.*

Data Analysis:

A Bayesian multiple treatment network meta-analysis with random effects and uninformative priors will be conducted to examine the overall effect size for the included treatment options for fibromyalgia. Network meta-analysis combines both direct and indirect evidences in all primary trials, which is commonly seen as its main advantage. In the Bayesian approach, the prior probability distribution needs to consider relevant prior information. Vague prior (mean 0, variance 10000) distributions will be used throughout this study to allow data to drive inferences. The Markov Chain Monte Carlo method will be applied to estimate posterior distributions for unknown variables. Then, the random effect model instead of the fixed effect model will be applied as the most conservative and appropriate analysis to deal with differences among trials. For random effects, homogeneous variance will be assumed in this study.

The between-study standard deviation is modeled using a uniform distribution of the 0 to 5 interval. A random effects model will be conducted using Markov chain Monte Carlo approach with Gibbs sampling based on simulation of 50 000 iterations in each of the three chains with different initial values (selected arbitrarily for convergence). To avoid the burn-in period, the first 10,000 iterations will be rejected.

We will estimate the summary odds ratios (ORs) for the acceptability outcomes, using a binomial likelihood model. On the other hand, pain, sleep, depression, fatigue, and quality of life outcomes will be presented as continuous data (mean value or mean changes), which the values are obtained using different patient-reported outcomes (PROs) scales, we will use the standardized mean difference (SMDs), calculated as effect measures. To calculate SMDs, we will use means and their standard deviations. An SMD of -0.20 indicates small differences between groups whereas -0.50 shows moderate and -0.80 large differences. If the standard error is only reported, it will be converted into standard deviation.[15,16] When studies did not report mean change, these values will be calculated as the arithmetic difference between baseline and follow-up. In case the standard error was only reported, it will be converted to standard deviation. [16]

© 2022 Farag HM et al. *JAMA Network Open.*

Loop-specific and side-splitting approaches will be conducted to verify homogeneity and consistency assumptions. In every iteration, the included fibromyalgia treatments will be ranked by their effect relative to arbitrary baseline. The results will be interpreted as associations when the 95% credible interval does not cross the values (zero for the SMDs and one for the odds ratios (OR)). The lower and upper limit of the traditional 95% credible interval (95%CrI) corresponds to the 2.5th and 97.5th percentiles of the posterior distribution respectively. Network meta-analysis is also possible to generate rankings for all concerned treatments on the basis of the level of effectiveness according to posterior probabilities (first best, second best, third best, etc.). The values of probability will be reported as the surface under the cumulative ranking (SUCRA). The best treatment has the SUCRA equal to 100%, while the worst treatment has the SUCRA equal to 0%. Sensitivity analysis will be conducted by excluding studies with a sample size less than hundred patients.

All statistical analyses will be conducting using WinBUGS with DoodleBUGS software (version 1.4.3, Medical Research Council (MRC) Biostatistics Unit, Cambridge, UK), and STATA software (version 15.1 for mac, Stata Corp, College Station, TX). The WinBUGS codes for random effect models of multi-arm trials for each outcome can be found in **eTables 16**.

© 2022 Farag HM et al. *JAMA Network Open.*

eReferences

1. Clauw, D.J., et al., *The science of fibromyalgia.* Mayo Clin Proc, 2011. **86**(9): p. 907-11.
2. Ablin, J.N., H. Cohen, and D. Buskila, *Mechanisms of Disease: genetics of fibromyalgia.* Nat Clin Pract Rheumatol, 2006. **2**(12): p. 671-8.
3. Eich, W., et al., *[Fibromyalgia syndrome. Definition, classification, clinical diagnosis and prognosis].* Schmerz, 2012. **26**(3): p. 247-58.
4. Hauser, W., et al., *Fibromyalgia.* Nat Rev Dis Primers, 2015. **1**: p. 15022.
5. Wolfe, F., et al., *The American College of Rheumatology 1990 Criteria for the Classification of Fibromyalgia. Report of the Multicenter Criteria Committee.* Arthritis Rheum, 1990. **33**(2): p. 160-72.
6. Wolfe, F., et al., *The American College of Rheumatology preliminary diagnostic criteria for fibromyalgia and measurement of symptom severity.* Arthritis Care Res (Hoboken), 2010. **62**(5): p. 600-10.
7. Eyre, S., G. Orozco, and J. Worthington, *The genetics revolution in rheumatology: large scale genomic arrays and genetic mapping.* Nat Rev Rheumatol, 2017. **13**(7): p. 421-432.
8. Ablin, J., L. Neumann, and D. Buskila, *Pathogenesis of fibromyalgia - a review.* Joint Bone Spine, 2008. **75**(3): p. 273-9.
9. Ablin, J.N. and D. Buskila, *Update on the genetics of the fibromyalgia syndrome.* Best Pract Res Clin Rheumatol, 2015. **29**(1): p. 20-8.
10. Fitzcharles, M.A., et al., *Classification and clinical diagnosis of fibromyalgia syndrome: recommendations of recent evidence-based interdisciplinary guidelines.* Evid Based Complement Alternat Med, 2013. **2013**: p. 528952.
11. Forte ML, B.M., Andrade KE, et al. , *Treatments for Fibromyalgia in Adult Subgroups [Internet].* Rockville (MD): Agency for Healthcare Research and Quality (US), 2015.
12. R., K., *Treatment of fibromyalgia.* Australian Prescriber, 2017. **40**(5): p. 179-183.
13. Wolfe, F., et al., *2016 Revisions to the 2010/2011 fibromyalgia diagnostic criteria.* Semin Arthritis Rheum, 2016. **46**(3): p. 319-329.
14. Hauser, W., et al., *Treatment of fibromyalgia syndrome with antidepressants: a meta-analysis.* Jama, 2009. **301**(2): p. 198-209.
15. Moher, D., et al., *Preferred reporting items for systematic reviews and meta-analyses: the PRISMA statement.* PLoS Med, 2009. **6**(7): p. e1000097.
16. Wan, X., et al., *Estimating the sample mean and standard deviation from the sample size, median, range and/or interquartile range.* BMC Med Res Methodol, 2014. **14**: p. 135.
17. Altman DG, B.J., *Standard deviations and standard errors.* BMJ, 2005. **331**(7521): p. 903.
18. Salanti, G., A.E. Ades, and J.P. Ioannidis, *Graphical methods and numerical summaries for presenting results from multiple-treatment meta-analysis: an overview and tutorial.* J Clin Epidemiol, 2011. **64**(2): p. 163-71.

© 2022 Farag HM et al. *JAMA Network Open.*

eAppendix 3. Changes Made to the protocol

We have made some changes in our analysis from the original protocol.

1) We had planned to only compute and present the results of the continuous outcomes (pain, sleep, depression, fatigue and quality of life), while reporting the acceptability outcome, if sufficient number of RCTs has reported the acceptability of the drugs for fibromyalgia patients as a reference for the clustered rankings comparisons versus other outcomes. This has to be changed since the number of randomized clinical trials reporting acceptability is 26 trials. For that, we added in the original protocol the description of the statistical analysis of the dichotomous outcomes, as well as the network meta-analysis model description and WinBUGS model codes
2) Hierarchy of tools for patient-reported outcomes assessment has been modified from the original protocol, and the hierarchy used in this network is reported in **eTable 3**
3) In the original protocol, we selected the following comparator's doses to be included in the analysis: pregabalin 75mg, 150mg, 300mg and 450mg; duloxetine 20mg, 40mg and 60mg or milnacipran 12.5mg, 25mg and 50mg and amitriptyline in any dose. We amended the comparators doses to be as the following: FDA approved doses of pregabalin 150mg, 300mg, and 450mg, and 600mg; duloxetine 60mg, and 120mg, or milnacipran 100 mg, and 200 mg
4) *In the* participants/ population section of the original protocol, an "equal to or more than sign" was missing in the following sentence: Adult patients (18 years of age) presenting with fibromyalgia as defined by the American College of Rheumatology (ACR) 1990, 2010. or 2016 criteria.

© 2022 Farag HM et al. *JAMA Network Open.*

eTable 1. Hierarchy of Tools for Patient-Reported Outcomes Assessment

**Pain outcome**
1. Visual analog scale (VAS)<br>2. Fibromyalgia Impact Questionnaire (FIQ)<br>3. Revised Fibromyalgia Impact Questionnaire (RFIQ)<br>4. Numeric Rating Scale (NRS)<br>5. Brief Pain Inventory (BPI)
**Sleep outcome**
1. VAS<br>2. FIQ<br>3. RFIQ<br>4. NRS
**Depression outcome**
1. Beck Depression Index (BDI)<br>2. VAS<br>3. Hamilton Depression Rating Scale (HDRS)<br>4. FIQ<br>5. RFIQ

© 2022 Farag HM et al. *JAMA Network Open.*

**Fatigue outcome**
1. VAS<br>2. Multidimensional Assessment of Fatigue (MAF)<br>3. VAS FIQ<br>4. RFIQ
**QoL outcome**
1. FIQ total score<br>2. 36-Item Short Form Survey (SF-36)<br>3. EQ-5D form,<br>4. other HRQOL scales

eTable 2. Study and Patient Characteristics

Source (Location)	Age, Mean, y (SD)	Female (%)/ White Race (%)	Diagnostic Criteria; Exclusion Criteria	Study Population		Treatment Group		Placebo Group, completed, No./ Total (%)	Method Quality, Jadad Score	Outcome Measures Used for Meta-analysis	
				Randomized/ Screened, No. (%)	Completed, No./ Total (%)	Design, Treatment, Duration	Completed, No./ Total (%)				
**Tricyclic Antidepressants (TCA): Amitriptyline**											
Carette et al, 1986[17] (Canada)	41.8 (10.4)	92.6 / NA	Smyth, Severe somatic disease, Inflammatory rheumatoid disease	NA	59/70 (84.3)	Parallel Amitriptyline 50mg/day, 9wk	27/34 (79.4)	32/36 (88.9)	4	Pain, VAS; Fatigue, VAS; Sleep, VAS; HRQOL VAS	
Goldenberg et al, 1986[18] (USA)	43.8 (NA)	NA / 87.1	Yunus, history of cardiac arrhythmia, peptic ulcer	NA	56/ 62 (90.3)	Parallel; Amitriptyline; 6 weeks	14/ 15 (93.3)	14/ 15 (93.3)	4	Pain VAS; Fatigue VAS; Sleep VAS; QOL VAS	
Kempenaers et al, 1994[19] (Belgium)	38 (7)	100/ NA	Yunus; Severe somatic disease, inflammatory rheumatoid disease, mental disorder	NA	23/ 36 (63.8)	Parallel; Amitriptyline 50 mg/day; 8 weeks	6/ 12 (50)	8/12 (66.7)	3	Pain VAS; Sleep VAS; Fatigue VAS; QOL VAS	
Carette et al, 1994[20] (Canada)	44 (9.7)	92.9/ NA	ACR 1900, inflammatory rheumatoid diseases, severe somatic diseases	NA	208/184 (88.5)	Parallel; Amitriptyline 10-50 mg/day; 24 weeks	76/84 (95.1)	37/ 42 (85.7)	4	Pain McGill Pain Questionnaire; Fatigue VAS; Sleep VAS	
Carette et al, 1995[21] (Canada)	43.8 (8)	95.5 / NA	ACR 1990; serious medical disease, inflammatory rheumatic disease	22 / 31 (71)	20 / 22 (90.9)	Crossover; Amitriptyline 25 mg/day; 16 weeks	22/ 22 (100)	20 / 22 (90.9)	4	Sleep VAS; Pain VAS; Fatigue VAS	
Ginsberg et al, 1996[22] (Belgium)	46 (11)	83/ 92	ACR 1990, severe somatic diseases, inflammatory rheumatoid diseases, age	NA	46/ 51	Parallel, sustained-release Amitriptyline 25 mg/day; 8 weeks	24/ 26 (92.3)	22 /25 (88)	4	Pain VAS; Fatigue VAS; Sleep VAS; QOL VAS	
Hannonen et al., 1998[23] (Finland)	49.7 (8.6)	100/ NA	ACR 1990; severe somatic diseases, age,	184/ 130 (70.6)	92/ 130 (70.8)	Parallel; Amitriptyline 12.5mg/day;	32/ 42 (76.2)	30/ 45 (66.7)	5	Pain VAS; Fatigue VAS; Sleep VAS; QOL NHP	

© 2022 Farag HM et al. *JAMA Network Open.*

			severe mental disorders			12 weeks				
Heymann et al, 2001[24] (Brazil)	53.4	100/ 65	ACR 1990; severe somatic diseases, age, inflammatory rheumatoid diseases	NA	106/ 118 (89.8)	Parallel; Amitriptyline 25 mg/day; 8 weeks	37/ 40 (92.5)	33/ 40 (82.5)	5	Pain VAS; Fatigue VAS; Sleep VAS; QOL FIQ
Fros et al, 2002[25] (Norway)	45.7 (10)	100 / NA	ACR 1990; Not completing the pain psychiatric test battery	55 / 61 (90.2)	49 / 55 (89.1)	Parallel; Amitriptyline 50 mg/day; 4 weeks	NA	NA	3	Pain VAS; Depression BDI; Negative thoughts ATQ-30; Anxiety sTAI-T
Braz et al, 2013[26] (Brazil)	44.3 (2)	100/ NA	ACR 1990; Inflammatory rheumatic disease, severe medical conditions	52 / 52 (100)	38 / 52 (73.1)	Parallel; Amitriptyline 25 mg/day; 12 weeks	13 / 16 (81.3)	13 / 17 (76.5)	5	Pain VAS; Sleep VAS; Fatigue VAS; QOL & Depression FIQ
**Source (Location)**	**Age, Mean, y (SD)**	**Female (%)/ White Race (%)**	**Diagnostic Criteria; Exclusion Criteria**	**Study Population**		**Treatment Group**		**Placebo Group, completed, No./ Total (%)**	**Jadad Score**	**Outcome Measures Used for Meta-analysis**
				Randomized/ Screened, No. (%)	Completed, No./ Total (%)	Design, Treatment, Duration	Completed, No./ Total (%)			
**Serotonin and Noradrenaline Reuptake Inhibitors: Duloxetine**										
Arnold et al, 2004[27] (USA)	49.9 (12.3)	88.5/ 88.5	ACR 1990; Inflammatory rheumatoid disease, unstable medical or psychiatric illness	207/ 555 (37.3)	124/ 207 (59.9)	Parallel; Duloxetine 120 mg/day; 12 weeks	58/ 104 (55.8)	66/103 (64.1)	4	Pain FIQ; QOL FIQ
Arnold et al, 2005[28] (USA)	49.6 (10.9)	100/ 89.5	ACR 1990; age, severe somatic diseases, inflammatory rheumatoid disease, mental disorders without major depression, disability reviews	354/ 745 (47.5)	215/ 354 (60.7)	Parallel; Duloxetine 60mg/day or 120mg/day; 12 weeks	147/234 (61.5)	68/120 (56.7)	3	Pain, BPI; Sleep, BPI; depression, HAMD; QOL, FIQ total score

© 2022 Farag HM et al. *JAMA Network Open.*

Chappell et al, 2008[29] (USA)	50.5 (10.05)	91.9 / 90.9	ACR 1990; Severe somatic disease; mental disorders except major depression	NA	204 / 330 (61.8)	Parallel; Duloxetine 60 mg/day; 27 weeks	103 / 162 (63.6)	101 / 168 (60.1)	5	Pain BPI; QOL FIQ; Fatigue MFI; Depression (HAMD)	
Russell et al, 2008[30] (USA)	51 (11)	94.8/ 84	ACR 1990; inflammatory rheumatoid disease, refractory to treatment, mental disorders but not depression, severe somatic diseases	520/ 1010 (51.5)	323/ 520 (62.1)	Parallel; Duloxetine 20 mg/day (not included in the analysis), 60mg/day and 120 mg/day; 26 weeks	205/ 376 (54.5)	72/144 (50)	4	Pain BPI; fatigue MFI; QOL FIQ total score	
Chappell et al, 2009 [31] (USA)	49 (11.1)	95.7/ 61.1	ACR 1990; Rheumatic Diseases, psychiatric illness	307/ 350 (87.7)	195/ 307 (63.5)	Parallel; Duloxetine (60 mg/day, 120 mg/day); 52 weeks	124/ 203 (61.1)	71/ 104 (68.3)	4	Pain PBI; Sleep and QOL FIQ; Fatigue MFI	
Arnold et al, 2010[32] (USA)	50.7 (11.3)	92.8 / 77.6	ACR 1990; Psychiatric disorder, diseases have symptoms overlapped with fibromyalgia	530 / 824 (64.3)	363 / 530 (68.5)	Parallel; Duloxetine 60–120 mg/day; 12weeks	176 / 263 (66.9)	187 / 267 (70)	4	Pain PGI; Sleep SF-36; Fatigue MFI; Depression BDI	
Arnold et al, 2012[33] (USA)	51 (12)	94.2 / 85.8	ACR 1990; psychiatric disorder, inflammatory rheumatoid disease	308 / 430 (71.6)	231/ 308 (0.75)	Parallel; Duloxetine 30 mg/day; 12 weeks	121 /155 (78.1)	110 /153 (71.9)	5	Pain BPI; QOL FIQ; Depression BDI	
Murakami et al, 2015 [34] (Japan)	47.8 (12)	82.2/ NA	ACR 1990; severe medical disease, disease with symptoms overlapped with fibromyalgia, Inflammatory rheumatoid disease, psychiatric disorder	748 / 1328 (56.3)	315/ 748	Parallel; Duloxetine 60mg/day; 14 weeks	166/ 191 (86.9)	149 / 195 (76.4)	5	Pain BPI; QOL FIQ; General health SF-36; Depression BDI	

© 2022 Farag HM et al. *JAMA Network Open.*

Source (Location)	Age, Mean, y (SD)	Female (%)/ White Race (%)	Diagnostic Criteria; Exclusion Criteria	Study Population		Treatment Group		Placebo Group, completed, No./ Total (%)	Jadad Score	Outcome Measures Used for Meta-analysis	
				Randomized/ Screened, No. (%)	Completed, No./ Total (%)	Design, Treatment, Duration	Completed, No./ Total (%)				
**Gamma-Aminobutyric Acid (GABA) Inhibitor: Pregabalin**											
Crofford et al, 2005[35] (USA)	48.6 (10.3)	91.5/ 93.2	ACR 1990; clinically significant or unstable medical or psychological condition, creatinine clearance £60 mL/min; applying for disability or engaged in litigation related to FMS	529/ 825 (64.1)	410/ 529 (77.5)	Parallel; Pregabalin (150mg/day, 300 mg/day); 8weeks	313/ 398 (78.6)	97/ 131 (74)	4	Pain NRS; Sleep MOS; Fatigue MAF; Depression HADS; QOL SF-36	
Arnold et al, 2007[36] (USA)	48.6 (10.6)	91/ 93	ACR 1990; Inflammatory rheumatoid disease, psychiatric conditions	529 / 825 (64.1)	410 / 529 (77.5)	Parallel; Pregabalin 450 mg/day; 8 weeks	NA		4	Depression and anxiety HADS; Pain VAS	
Arnold et al, 2008[37] (USA)	50	95.3/ 91	ACR 1990; Inflammatory rheumatoid disease, active infections, sever painful disorder, mental disorders	745/ 1195 (62.3)	486/ 745 (65.2)	Parallel; Pregabalin (300 mg/day, 450 mg/day, 600 mg/day); 14 weeks	262/ 561 (46.7)	125/ 184 (67.9)	5	Pain VAS; Sleep MOS Scale; Depression HADS; Fatigue MAF; QOL SF-36	
Mease et al, 2008[38] (USA)	49 (11)	94/ 90	ACR 1990	748/1328 (56.3)	485/ 748 (64.8)	Parallel; Pregabalin (300 mg/day, 450 mg/day, 600 mg/day); 13 weeks	355/ 558 (63.6)	130/ 190 (68.4)	4	Pain NRS; Fatigue MFI; Sleep MOS; QOL FIQ	
Pauer et al, 2011[39] (USA)	48.5 (11.2)	91 / 76	ACR 1990; High placebo response (>30% decrease on the VAS following the 1-week run-	736 / 986 (74.6)	518 /736 (70.4)	Parallel; Pregabalin (300 mg/day, 450 mg/day, 600 mg/day); 14 weeks	377 / 552 (68.3)	141 / 184 (76.6)	3	Pain NRS, PGIC; Sleep MOS; QOL FIQ; Depression HADS	

© 2022 Farag HM et al. *JAMA Network Open.*

			in period compared with screening							
Ohta et al, 2012[40] (Japan)	47.9 (12.6)	90.4/ NA	ACR 1990	498 / 501 (99.4)	415/ 501 (82.8)	Parallel; Pregabalin (150- 450 mg/day); 17 weeks	207/ 251 (82.5)	208 / 250 (83.2)	5	Pain NRS & PGIC; Depression HADS; QOL FIQ; Sleep MOS
Arnold et al, 2015[41] (USA)	50 (10)	93.3/ 93.8	ACR 1990; severe depression, diseases overlapped with fibromyalgia	197/ 318 (61.9)	149/ 193 (77.2)	Crossover; Pregabalin (300 mg/day, 450 mg/day); 12 weeks	159/ 181 (87.8)	155/177 (87.6)	5	Pain NRS; Depression HADRS; QOL and Fatigue FIQ; Sleep SSQ
**Source (Location)**	**Age, Mean, y (SD)**	**Female (%)/ White Race (%)**	**Diagnostic Criteria; Exclusion Criteria**	**Study Population**		**Treatment Group**		**Placebo Group, completed, No./ Total (%)**	**Jadad Score**	**Outcome Measures Used for Meta-analysis**
				Randomized/ Screened, No. (%)	Completed, No./ Total (%)	Design, Treatment, Duration	Completed, No./ Total (%)			
**Serotonin and Noradrenaline Reuptake Inhibitors: Milnacipran**										
Vitton et al, 2004[42] (USA)	46-48; NA	96-98/ 79-89	ACR 1990; mental disorders, somatic diseases, age	125/ 184 (67.9)	90/ 125 (71.1)	Parallel; Milnacipran 25-200 mg/day; 12 weeks	69/ 97 (71.1)	21/ 28 (75)	3	Pain VAS; Sleep Jenkins scale
Gendreau et al, 2005[43] (USA)	47 (11.1)	98 / 84	ACR 1990; Psychosis, liver or kidney dysfunction	125 / 184 (67.9)	90 / 125 (72)	Parallel; Milnacipran (100 mg/day, 200 mg/day); 12 weeks	69 / 97 (71.1)	21 / 28 (75)	5	Pain VAS; QOL FIQ; Fatigue SF-36; Sleep Jenkins Questionnaire
Clauw et al, 2008[44] (USA)	50 (10.9)	97/ 93.5	ACR 1990; somatic diseases, severe mental disorder including major depression	1207/ 2270 (53.2)	811/ 1196 (67.6)	Parallel; Milnacipran (100 mg/day or 200 mg/day); 15 weeks	521/ 802 (65)	290/ 405 (71.6)	5	Pain VAS; Fatigue MFI; QOL FIQ; Sleep SF-36
Mease et al, 2009[45] (USA)	49.9 (10.6)	95 / 93.4	ACR 1990; Severe psychiatric illness; serious	888 / 1639 (54.2)	512 / 888 (57.7)	Parallel; Milnacipran (100 mg/day, 200 mg/day); 27 weeks	367 / 665 (55.2)	145 / 223 (65)	4	2-measure composite (>30% VAS pain improvement & PGIC);

© 2022 Farag HM et al. *JAMA Network Open.*

			medical conditions							Sleep MOS; Fatigue MFI; Function SF-36; QOL FIQ	
Arnold et al, 2010[46] (USA)	49.1 (10.8)	96.9/ 91.9	ACR 1990; disorders with symptoms similar to fibromyalgia, mental disorders (BDI >25)	1025/ 1947 (52.6)	716/ 1025 (70)	Parallel; Milnacipran 100 mg/day; 12 weeks	357/ 516 (69.2)	359/ 509 (70.5)	5	2 composite responder indices:1) >30% improvement in BPI, 2) SF-36 (much or very much improved) ; Fatigue (MFI), Depression (BDI)	
Branco et al, 2010[47] (France)	48.3 (9.3)	95.1 / NA	ACR 1990; Severe psychiatric disorder, serious medical condition	884 / 1406 (62.9)	678 / 884 (76.7)	Parallel; Milnacipran 200 mg/day; 16 weeks	308 / 430 (70.8)	370 / 446 (82.4)	4	2-measure composite responder rate (pain >30% VAS & PGIC); QOL FIQ; Pain VAS, Fatigue VAS; Sleep VAS	
Goldenberg et al, 2010[48] (USA)	49.7 (10.11)	96.9 / 93.8	ACR 1990; severe psychiatric illness, cardiovascular, pulmonary, hepatic, renal, gastrointestinal, endocrine diseases and cancer	449/ 512 (87.7)	301/ 449 (67)	Parallel, Milnacipran (100 mg/day, 200 mg/day); 28 weeks	217 / 320 (67.8)	NA	3	Pain VAS; QOL FIQ; Fatigue MFI; Depression BDI; Sleep MOS	
Branco et al, 2011[49] (USA)	50 (9.9)	91.2 / NA	ACR 1990; severe psychiatric illness	468 / 678 (69)	NA	Parallel, Milnacipran (100 mg/day, 200 mg/day); 52 weeks	283 / 468 (60.5)	NA	3	2-measure composite (pain VAS & PGIC); QOL & sleep FIQ	
Staud et al, 2015[50] (USA)	46.9 (11.5)	91.3 / 95.6	ACR 1990; relevant medical condition, major depression, analgesics, antipsychotics and antidepressant use	46 / 61 (75.4)	35 / 46 (76.1)	Parallel; Milnacipran 50 mg/day; 6 weeks	17 / 31 (54.8)	18 / 30 (60)	3	Pain VAS; Fatigue VAS; Depression VAS	

© 2022 Farag HM et al. *JAMA Network Open.*

Ahmed et al, 2016[51] (USA)	49.2 (11)	89.5 / 89.5	ACR 1990; severe mental disorder, sleep apnea	19 / 45 (42.2)	15 / 19 (78.9)	Cross-over, Per-protocol; Milnacipran 100 mg/day; 12 weeks	15 / 19 (78.9)	17 / 19 (89.5)	5	Polysomnographic measures of sleep; Fatigue FSS; Pain BPI, subject-rated sleep; QOL FIQ	
Pickering et al, 2018[52] (France)	46.7 (10.6)	100/ NA	ACR 2010; milnacipran contraindication, hypertension or heart disease, renal impairment, mental disorder, diabetes, taking alcohol, hypnotics, analgesics, opioids, diuretics, NSAIDs, oral anticoagulants, aspirin, SNRIs, digitalis, CYP1A2 inhibitor, pregnant or breastfeeding women	54/ 54 (100)	48/ 54 (88.9)	Parallel, per-protocol; Milnacipran; 50-100mg/day; 4 weeks	24/ 24 (100)	24/ 24 (100)	3	Pain NRS	

Abbreviations: ACR 1990, The American College of Rheumatology 1990 Criteria For the Classification of Fibromyalgia; ; BDI, Beck Depression Inventory; BPI, Brief Pain Inventory; FIQ, Fibromyalgia Impact Questionnaire; FSS, Fatigue Severity Scale; HDRS, Hamilton Depression Rating Scale; QOL, Health related quality of life; MOS, Medical Outcomes study Sleep Scale; MFI, Multidimensional Fatigue Inventory; MFA, Multidimensional Assessment of Fatigue Score Global Index; NA, Not Available; NHP, Nottingham Health Profile; NR, not reported; NRS, Numeric Rating Scale; PGIC, Patient Global Impression of Change; SDS, Sheehan Disability scale; SF-36, Short Form Health Survey; SSQ, The Subject Sleep Questionnaire; VAS, Visual Analogue Scale.
NOTE. The order of the presented studies is arranged according to the year of publication.

References

1. Clauw DJ, Arnold LM, McCarberg BH, FibroCollaborative. The science of fibromyalgia. *Mayo Clin Proc.* 2011;86(9):907-911.
2. Ablin JN, Cohen H, Buskila D. Mechanisms of Disease: genetics of fibromyalgia. *Nat Clin Pract Rheumatol.* 2006;2(12):671-678.
3. Eich W, Hauser W, Arnold B, et al. [Fibromyalgia syndrome. Definition, classification, clinical diagnosis and prognosis]. *Schmerz (Berlin, Germany).* 2012;26(3):247-258.
4. Hauser W, Ablin J, Fitzcharles MA, et al. Fibromyalgia. *Nat Rev Dis Primers.* 2015;1:15022.

© 2022 Farag HM et al. *JAMA Network Open.*

5. Wolfe F, Smythe HA, Yunus MB, et al. The American College of Rheumatology 1990 Criteria for the Classification of Fibromyalgia. Report of the Multicenter Criteria Committee. *Arthritis Rheum.* 1990;33(2):160-172.
6. Wolfe F, Clauw DJ, Fitzcharles MA, et al. The American College of Rheumatology preliminary diagnostic criteria for fibromyalgia and measurement of symptom severity. *Arthritis care & research.* 2010;62(5):600-610.
7. Ablin J, Neumann L, Buskila D. Pathogenesis of fibromyalgia - a review. *Joint Bone Spine.* 2008;75(3):273-279.
8. Ablin JN, Buskila D. Update on the genetics of the fibromyalgia syndrome. *Best Pract Res Clin Rheumatol.* 2015;29(1):20-28.
9. Eyre S, Orozco G, Worthington J. The genetics revolution in rheumatology: large scale genomic arrays and genetic mapping. *Nat Rev Rheumatol.* 2017;13(7):421-432.
10. Fitzcharles MA, Shir Y, Ablin JN, et al. Classification and clinical diagnosis of fibromyalgia syndrome: recommendations of recent evidence-based interdisciplinary guidelines. *Evid Based Complement Alternat Med.* 2013;2013:528952.
11. Forte ML BM, Andrade KE, et al. . Treatments for Fibromyalgia in Adult Subgroups [Internet]. *Rockville (MD): Agency for Healthcare Research and Quality (US).* 2015.
12. Kwiatek R. Treatment of fibromyalgia. *Australian Prescriber.* 2017;40(5):179-183.
13. Moher D, Liberati A, Tetzlaff J, Altman DG, Group P. Preferred reporting items for systematic reviews and meta-analyses: the PRISMA statement. *PLoS Med.* 2009;6(7):e1000097.
14. Wolfe F, Clauw DJ, Fitzcharles MA, et al. 2016 Revisions to the 2010/2011 fibromyalgia diagnostic criteria. *Semin Arthritis Rheum.* 2016;46(3):319-329.
15. Wan X, Wang W, Liu J, Tong T. Estimating the sample mean and standard deviation from the sample size, median, range and/or interquartile range. *BMC medical research methodology.* 2014;14:135.
16. Altman DG BJ. Standard deviations and standard errors. *BMJ.* 2005;331(7521):903.
17. Carette SM, G. A.; Bell, D. A.; Fam, A. G. Evaluation of amitriptyline in primary fibrositis. A double-blind, placebo-controlled study. *Arthritis and rheumatism.* 1986;29(5):655-659.
18. Goldenberg DLF, D. T.; Dinerman, H. A randomized, controlled trial of amitriptyline and naproxen in the treatment of patients with fibromyalgia. *Arthritis and rheumatism.* 1986;29(11):1371-1377.
19. Kempenaers CS, G.; Vander Elst, M.; Fransolet, L.; Mingard, P.; de Maertelaer, V.; Appelboom, T.; Mendlewicz, J. Effect of an antidiencephalon immune serum on pain and sleep in primary fibromyalgia. *Neuropsychobiology.* 1994;30(2-3):66-72.
20. Carette SB, M. J.; Reynolds, W. J.; Haraoui, B.; McCain, G. A.; Bykerk, V. P.; Edworthy, S. M.; Baron, M.; Koehler, B. E.; Fam, A. G.; et al.,. Comparison of amitriptyline, cyclobenzaprine, and placebo in the treatment of fibromyalgia. A randomized, double-blind clinical trial. *Arthritis Rheum.* 1994;37(1):32-40.
21. Carette SO, G.; Guimont, C.; Steriade, M. Sleep electroencephalography and the clinical response to amitriptyline in patients with fibromyalgia. *Arthritis Rheum.* 1995;38(9):1211-1217.

© 2022 Farag HM et al. *JAMA Network Open.*

22. Ginsberg FM, A.; Joos, E.; Vanhove, P.; Famaey, J. P. A randomized placebo-controlled trial of sustained-release amitriptyline in primary fibromyalgia. *Journal of Musculoskeletal Pain.* 1996;4(3):37-47.
23. Hannonen PM, K.; Yli-Kerttula, U.; Isomeri, R.; Roponen, P. A randomized, double-blind, placebo-controlled study of moclobemide and amitriptyline in the treatment of fibromyalgia in females without psychiatric disorder. *Br J Rheumatol.* 1998;37(12):1279-1286.
24. Heymann REH, M.; Feldman, D. A double-blind, randomized, controlled study of amitriptyline, nortriptyline and placebo in patients with fibromyalgia. An analysis of outcome measures. *Clinical and experimental rheumatology.* 2001;19(6):697-702.
25. Fors EAS, H.; Gotestam, K. G. The effect of guided imagery and amitriptyline on daily fibromyalgia pain: a prospective, randomized, controlled trial. *J Psychiatr Res.* 2002;36(3):179-187.
26. Braz ASM, L. C.; Paula, A. P.; Diniz, M. F.; Almeida, R. N. Effects of Panax ginseng extract in patients with fibromyalgia: a 12-week, randomized, double-blind, placebo-controlled trial. *Braz J Psychiatr.* 2013;35(1):21-28.
27. Arnold LML, Y.; Crofford, L. J.; Wohlreich, M.; Detke, M. J.; Iyengar, S.; Goldstein, D. J. A double-blind, multicenter trial comparing duloxetine with placebo in the treatment of fibromyalgia patients with or without major depressive disorder. *Arthritis Rheum.* 2004;50(9):2974-2984.
28. Arnold LMR, A.; Pritchett, Y. L.; D'Souza, D. N.; Goldstein, D. J.; Iyengar, S.; Wernicke, J. F. A randomized, double-blind, placebo-controlled trial of duloxetine in the treatment of women with fibromyalgia with or without major depressive disorder. *Pain.* 2005;119(1-3):5-15.
29. Chappell AS, Bradley LA, Wiltse C, Detke MJ, D'Souza DN, Spaeth M. A six-month double-blind, placebo-controlled, randomized clinical trial of duloxetine for the treatment of fibromyalgia. *Int J Gen Med.* 2008;1:91-102.
30. Russell IJM, P. J.; Smith, T. R.; Kajdasz, D. K.; Wohlreich, M. M.; Detke, M. J.; Walker, D. J.; Chappell, A. S.; Arnold, L. M. Efficacy and safety of duloxetine for treatment of fibromyalgia in patients with or without major depressive disorder: Results from a 6-month, randomized, double-blind, placebo-controlled, fixed-dose trial. *Pain.* 2008;136(3):432-444.
31. Chappell ASL, G.; Kajdasz, D. K.; Scheinberg, M.; D'Souza, D. N.; Moldofsky, H. A 1-year safety and efficacy study of duloxetine in patients with fibromyalgia. *Clin J Pain.* 2009;25(5):365-375.
32. Arnold LMC, D.; Wang, F.; Ahl, J.; Gaynor, P. J.; Wohlreich, M. M. Flexible dosed duloxetine in the treatment of fibromyalgia: a randomized, double-blind, placebo-controlled trial. *The Journal of rheumatology.* 2010;37(12):2578-2586.
33. Arnold LMZ, S.; Pangallo, B. A. Efficacy and safety of duloxetine 30 mg/d in patients with fibromyalgia: A randomized, double-blind, placebo-controlled study. *Clinical Journal of Pain.* 2012;28(9):775-781.
34. Murakami M, Osada K, Mizuno H, Ochiai T, Alev L, Nishioka K. A randomized, double-blind, placebo-controlled phase III trial of duloxetine in Japanese fibromyalgia patients. *Arthritis Res Ther.* 2015;17:224.

© 2022 Farag HM et al. *JAMA Network Open.*

35. Crofford LJR, M. C.; Mease, P. J.; Russell, I. J.; Dworkin, R. H.; Corbin, A. E.; Young, J. P., Jr.; LaMoreaux, L. K.; Martin, S. A.; Sharma, U. Pregabalin for the treatment of fibromyalgia syndrome: results of a randomized, double-blind, placebo-controlled trial. *Arthritis Rheum.* 2005;52(4):1264-1273.
36. Arnold LMC, L. J.; Martin, S. A.; Young, J. P.; Sharma, U. The effect of anxiety and depression on improvements in pain in a randomized, controlled trial of pregabalin for treatment of fibromyalgia. *Pain Medicine.* 2007;8(8):633-638.
37. Arnold LMR, I. J.; Diri, E. W.; Duan, W. R.; Young, J. P., Jr.; Sharma, U.; Martin, S. A.; Barrett, J. A.; Haig, G. A 14-week, randomized, double-blinded, placebo-controlled monotherapy trial of pregabalin in patients with fibromyalgia. *J Pain.* 2008;9(9):792-805.
38. Mease PJR, I. J.; Arnold, L. M.; Florian, H.; Young, J. P., Jr.; Martin, S. A.; Sharma, U. A randomized, double-blind, placebo-controlled, phase III trial of pregabalin in the treatment of patients with fibromyalgia. *The Journal of rheumatology.* 2008;35(3):502-514.
39. Pauer LW, A.; Arsenault, P.; Jespersen, A.; Whelan, L.; Atkinson, G.; Leon, T.; Zeiher, B. An international, randomized, double-blind, placebo-controlled, phase III trial of pregabalin monotherapy in treatment of patients with fibromyalgia. *The Journal of rheumatology.* 2011;38(12):2643-2652.
40. Ohta H, Oka H, Usui C, Ohkura M, Suzuki M, Nishioka K. A randomized, double-blind, multicenter, placebo-controlled phase III trial to evaluate the efficacy and safety of pregabalin in Japanese patients with fibromyalgia. *Arthritis Res Ther.* 2012;14(5):R217.
41. Arnold LMS-P, P.; Arsenault, P.; Khan, T.; Bhadra Brown, P.; Clair, A.; Scavone, J. M.; Driscoll, J.; Landen, J.; Pauer, L. Efficacy and Safety of Pregabalin in Patients with Fibromyalgia and Comorbid Depression Taking Concurrent Antidepressant Medication: A Randomized, Placebo-controlled Study. *The Journal of rheumatology.* 2015;42(7):1237-1244.
42. Vitton OG, M.; Gendreau, J.; Kranzler, J.; Rao, S. G. A double-blind placebo-controlled trial of milnacipran in the treatment of fibromyalgia. *Human Psychopharmacology.* 2004;19(SUPPL. 1):S27-S35.
43. Gendreau RMT, M. D.; Gendreau, J. F.; Kranzler, J. D.; Ribeiro, S.; Gracely, R. H.; Williams, D. A.; Mease, P. J.; McLean, S. A.; Clauw, D. J. Efficacy of milnacipran in patients with fibromyalgia. *The Journal of rheumatology.* 2005;32(10):1975-1985.
44. Clauw DJM, P.; Palmer, R. H.; Gendreau, R. M.; Wang, Y. Milnacipran for the treatment of fibromyalgia in adults: a 15-week, multicenter, randomized, double-blind, placebo-controlled, multiple-dose clinical trial. *Clin Ther.* 2008;30(11):1988-2004.
45. Mease PJC, D. J.; Gendreau, R. M.; Rao, S. G.; Kranzler, J.; Chen, W.; Palmer, R. H. The efficacy and safety of milnacipran for treatment of fibromyalgia. a randomized, double-blind, placebo-controlled trial. *The Journal of rheumatology.* 2009;36(2):398-409.
46. Arnold LMG, R. M.; Palmer, R. H.; Gendreau, J. F.; Wang, Y. Efficacy and safety of milnacipran 100 mg/day in patients with fibromyalgia: results of a randomized, double-blind, placebo-controlled trial. *Arthritis Rheum.* 2010;62(9):2745-2756.

© 2022 Farag HM et al. *JAMA Network Open.*

47. Branco JCZ, O.; Perrot, S.; Mainguy, Y. A European multicenter randomized double-blind placebo-controlled monotherapy clinical trial of milnacipran in treatment of fibromyalgia. *The Journal of rheumatology.* 2010;37(4):851-859.
48. Goldenberg DLC, D. J.; Palmer, R. H.; Mease, P.; Chen, W.; Gendreau, R. M. Durability of therapeutic response to milnacipran treatment for fibromyalgia. Results of a randomized, double-blind, monotherapy 6-month extension study. *Pain Med.* 2010;11(2):180-194.
49. Branco JCC, P.; Montagne, A.; Bouroubi, A. Longterm therapeutic response to milnacipran treatment for fibromyalgia. A European 1-year extension study following a 3-month study. *The Journal of rheumatology.* 2011;38(7):1403-1412.
50. Staud R, Lucas YE, Price DD, Robinson ME. Effects of milnacipran on clinical pain and hyperalgesia of patients with fibromyalgia: results of a 6-week randomized controlled trial. *J Pain.* 2015;16(8):750-759.
51. Ahmed M, Aamir R, Jishi Z, Scharf MB. The Effects of Milnacipran on Sleep Disturbance in Fibromyalgia: A Randomized, Double-Blind, Placebo-Controlled, Two-Way Crossover Study. *J Clin Sleep Med.* 2016;12(1):79-86.
52. Pickering G, Macian N, Delage N, et al. Milnacipran poorly modulates pain in patients suffering from fibromyalgia: a randomized double-blind controlled study. *Drug design, development and therapy.* 2018;12:2485-2496.
53. Chaimani A, Higgins JP, Mavridis D, Spyridonos P, Salanti G. Graphical tools for network meta-analysis in Stata. *PLoS One.* 2013;8(10):e76654.

© 2022 Farag HM et al. *JAMA Network Open.*

eTable 3. Risk of Bias Assessment

eTable 3A. Risk of bias assessment for the included randomized clinical trials

Ahmed et al. 2016	Risk of Bias	Reason/Quotation
Random sequence generation (selection bias)	Low risk	"Computer-generated random numbers were used for enrollment and allocation to sequence (1:1): milnacipran → placebo or placebo → milnacipran"
Allocation concealment (selection bias)	Low risk	"Computer-generated random numbers were used for enrollment and allocation to sequence (1:1): milnacipran → placebo or placebo → milnacipran. A non-involved staff member generated the random allocation sequence and kept an electronic copy in a secure location."
Blinding of participants and personnel (performance bias)	Low risk	"The investigator, clinical staff, subjects, and the study sponsor were blinded to sequence allocation".
Blinding of outcome assessment (detection bias)	Low risk	"The investigator, clinical staff, subjects, and the study sponsor were blinded to sequence allocation".
Incomplete outcome data (attrition bias)	Low risk	"Only 19 subjects were enrolled with 15 completers, reducing the power of the study to 80%. For missing data, the last-observation-carried-forward approach (LOCF) while receiving the study medication or placebo was used for the sleep diary quality of sleep scale."
Selective reporting (reporting bias)	Low risk	All registered outcomes on the protocol (NCT01234675) were reported.
Other bias	Low risk	No other sources of bias, such as cross over design concerns or baseline imbalance could be detected.
**Arnold et al. 2004**	**Risk of Bias**	**Reason/Quotation**

© 2022 Farag HM et al. *JAMA Network Open.*

Random sequence generation (selection bias)	Low risk	"At visit 3, subjects were randomized to 1 of the following 2 treatment groups: duloxetine or placebo in a 1:1 ratio. Assignment to treatment groups was determined by a computer-generated random sequence using an interactive voice response system".
Allocation concealment (selection bias)	Low risk	"Assignment to treatment groups was determined by a computer-generated random sequence using an interactive voice response system".
Blinding of participants and personnel (performance bias)	Low risk	"The 3–30-day screening phase was followed by a 1-week, single-blind, placebo lead-in phase at visit 2. Then, Treatment was double-blind for 12 weeks".
Blinding of outcome assessment (detection bias)	Low risk	All patients entered the initial placebo lead-in phase. During the actual study treatment, physicians were also blinded.
Incomplete outcome data (attrition bias)	Low risk	A large number of patients discontinued the study. However, the authors performed adequate intention to treat analysis.
Selective reporting (reporting bias)	Low risk	Although the protocol was not found, relevant outcomes, commonly reported in other trials, were reported.
Other bias	Low risk	To reduce baseline imbalance, subjects received placebo for 1 week in an attempt to obtain an unbiased evaluation of the baseline variables, including safety measures. No concerns on study design were noted.
**Arnold et al. 2005**	**Risk of Bias**	**Reason/Quotation**
Random sequence generation (selection bias)	Unclear risk	The method of random sequence generation was not reported.
Allocation concealment (selection bias)	Unclear risk	The method of allocation concealment was not reported.
Blinding of participants and personnel (performance bias)	Low risk	The study is double-blind and there is no indication that the blinding was broken.

© 2022 Farag HM et al. *JAMA Network Open.*

Blinding of outcome assessment (detection bias)	Low risk	The study is double-blind and there is no indication that the blinding was broken.
Incomplete outcome data (attrition bias)	Low risk	A large number of patients discontinued the study. However, the authors performed adequate intention to treat analysis.
Selective reporting (reporting bias)	Low risk	Although the protocol was not found, relevant outcomes, commonly reported in other trials, were reported.
Other bias	Low risk	No other sources of bias could be detected.
**Arnold et al. 2007/Crofford et al. 2005**	**Risk of Bias**	**Reason/Quotation**
Random sequence generation (selection bias)	Low risk	Randomization was by computer-generated code using a block size of 8.
Allocation concealment (selection bias)	Unclear risk	The method of allocation concealment was not mentioned.
Blinding of participants and personnel (performance bias)	Low risk	The study is double-blind and there is no indication that the blinding was broken.
Blinding of outcome assessment (detection bias)	Low risk	The study is double-blind and there is no indication that the blinding was broken.
Incomplete outcome data (attrition bias)	Low risk	A large number of patients discontinued the study. However, the authors performed adequate intention to treat analysis.
Selective reporting (reporting bias)	High risk	Several relevant outcomes were not adequately reported in both articles.
Other bias	High risk	Apparent marked differences in baseline characteristics between the study groups are noted.
**Arnold et al. 2008**	**Risk of Bias**	**Reason/Quotation**
Random sequence generation (selection bias)	Low risk	"Random assignment to treatment regimens used a 1:1:1:1 ratio according to a computer-generated pseudorandom code using the method of random permuted blocks (i.e., block size of 4). Random assignment was managed by a telerandomization system".

© 2022 Farag HM et al. *JAMA Network Open.*

Allocation concealment (selection bias)	Low risk	"Random assignment was managed by a telerandomization system".
Blinding of participants and personnel (performance bias)	Low risk	"This was a 14-week, randomized, double-blinded, multiple-dose, placebo-controlled, parallel-group, multicenter trial with a 1-week, single-blinded, placebo run-in phase followed by a 2-week, double-blinded, dose-escalation phase and then a 12-week, fixed-dose phase".
Blinding of outcome assessment (detection bias)	Low risk	All patients entered the initial placebo lead-in phase. During the actual study treatment, physicians were also blinded.
Incomplete outcome data (attrition bias)	Low risk	A large number of patients discontinued the study. However, the authors performed adequate intention to treat analysis.
Selective reporting (reporting bias)	Low risk	Relevant outcomes were adequately reported.
Other bias	Low risk	To reduce baseline imbalance, subjects received placebo for 1 week to obtain an unbiased evaluation of baseline variables. No concerns on study design were noted.
**Arnold et al. 2010a**	**Risk of Bias**	**Reason/Quotation**
Random sequence generation (selection bias)	Low risk	"Patients were randomly assigned 1:1 in a double-blind fashion to duloxetine 60 mg once daily (QD) or placebo by a computer-generated random sequence using an interactive voice response system (IVRS)".
Allocation concealment (selection bias)	Low risk	
Blinding of participants and personnel (performance bias)	Low risk	"The protocol employed a variable transition to active treatment strategy, whereby investigators and patients were told that active study drug would be initiated sometime between randomization and Week 4, thereby blinding the onset of active treatment to reduce the patient's expectations of experiencing side effects or improved symptoms".
Blinding of outcome assessment (detection bias)	Low risk	

© 2022 Farag HM et al. *JAMA Network Open.*

Incomplete outcome data (attrition bias)	Low risk	Following randomization of 540 subjects, only 23 (< 10%) were excluded from the analysis.
Selective reporting (reporting bias)	Low risk	All registered outcomes on the protocol (NCT00673452) were reported.
Other bias	Unclear risk	No sufficient information to assess whether other sources of bias existed.
**Arnold et al. 2010b**	**Risk of Bias**	**Reason/Quotation**
Random sequence generation (selection bias)	Low risk	"Assignment to treatment groups was conducted centrally (i.e., at the study level) using an interactive voice response system generated and maintained by Premier Research and securely kept by Forest Research Institute, Inc. The randomization assignments were generated in blocks of 4 so that each center would have a balanced distribution of patient assignments".
Allocation concealment (selection bias)	Low risk	"Assignment to treatment groups was conducted centrally (i.e., at the study level) using an interactive voice response system".
Blinding of participants and personnel (performance bias)	Low risk	"Clinical staff, investigators, patients, and the study sponsor were blinded to treatment allocation".
Blinding of outcome assessment (detection bias)	Low risk	"Clinical staff, investigators, patients, and the study sponsor were blinded to treatment allocation".
Incomplete outcome data (attrition bias)	Low risk	Although a large number of the study participants withdraw from the study, adequate intention to treat analysis was conducted by the authors.
Selective reporting (reporting bias)	Low risk	All registered outcomes on the protocol (NCT00314249) were reported.
Other bias	Low risk	No other sources of bias could be detected.
**Arnold et al. 2012**	**Risk of Bias**	**Reason/Quotation**
Random sequence generation (selection bias)	Low risk	Patients were randomized by a computer-generated random sequence using an interactive voice response system

© 2022 Farag HM et al. *JAMA Network Open.*

Allocation concealment (selection bias)	Low risk	Patients were randomized by a computer-generated random sequence using an interactive voice response system
Blinding of participants and personnel (performance bias)	Low risk	The duloxetine and placebo capsules were identical in appearance to maintain the blinding.
Blinding of outcome assessment (detection bias)	Low risk	Double-blind "Patients and investigators were kept blinded to the rescue criteria and dose increase"
Incomplete outcome data (attrition bias)	Low risk	More than 10% of subjects discontinued the study; however, the authors performed intention to treat analysis.
Selective reporting (reporting bias)	Low risk	Adequate reporting of efficacy and safety outcomes was conducted.
Other bias	Low risk	No other sources of bias could be detected.
**Arnold et al. 2015**	**Risk of Bias**	**Reason/Quotation**
Random sequence generation (selection bias)	Unclear risk	The method of random sequence generation was not mentioned.
Allocation concealment (selection bias)	Unclear risk	The method of allocation concealment was not mentioned.
Blinding of participants and personnel (performance bias)	Low risk	"A randomized, 2-way crossover, double-blind, placebo-controlled study conducted at 34 centers"
Blinding of outcome assessment (detection bias)	Unclear risk	It was unclear whether the individuals involved in outcome assessment were blinded.
Incomplete outcome data (attrition bias)	Low risk	12.4% of subjects discontinued the study; however, all randomized patients were analyzed.
Selective reporting (reporting bias)	Unclear risk	No sufficient information to assess whether reporting bias existed.
Other bias	Unclear risk	No sufficient information to assess whether other sources of bias existed.
**Branco et al. 2010**	**Risk of Bias**	**Reason/Quotation**
Random sequence generation (selection bias)	Unclear risk	The method of random sequence generation was not mentioned.

© 2022 Farag HM et al. *JAMA Network Open.*

Allocation concealment (selection bias)	Unclear risk	The method of allocation concealment was not mentioned.
Blinding of participants and personnel (performance bias)	Low risk	"In patients receiving placebo, twice daily sham dosing was used to maintain blinding".
Blinding of outcome assessment (detection bias)	Low risk	The study is double-blind and there is no indication that the blinding was broken.
Incomplete outcome data (attrition bias)	Low risk	Adequate intention to treat analysis was performed.
Selective reporting (reporting bias)	Low risk	All registered outcomes on the protocol (NCT00436033) were reported.
Other bias	Unclear risk	No sufficient information to assess whether other sources of bias existed.
**Branco et al. 2011**	**Risk of Bias**	**Reason/Quotation**
Random sequence generation (selection bias)	Unclear risk	The method of random sequence generation was not mentioned.
Allocation concealment (selection bias)	Unclear risk	The method of allocation concealment was not mentioned.
Blinding of participants and personnel (performance bias)	Low risk	"Patients initially randomized to the milnacipran 200 mg/day group in the lead-in study were blindly maintained on milnacipran 200 mg/day".
Blinding of outcome assessment (detection bias)	Low risk	The study is double-blind and there is no indication that the blinding was broken.
Incomplete outcome data (attrition bias)	Low risk	"Changes in efficacy scores were calculated from the lead-in study and extension study baselines using both the last observation carried forward".
Selective reporting (reporting bias)	Low risk	All registered outcomes on the protocol (NCT00757731) were reported.
Other bias	Low risk	No other sources of bias could be detected.
**Braz et al. 2013**	**Risk of Bias**	**Reason/Quotation**
Random sequence generation (selection bias)	Unclear risk	The method of random sequence generation was not mentioned.
Allocation concealment (selection bias)	Unclear risk	The method of allocation concealment was not mentioned.

© 2022 Farag HM et al. *JAMA Network Open.*

Blinding of participants and personnel (performance bias)	Low risk	"The study consisted of a randomized, double-blind, controlled, 12-week clinical trial".
Blinding of outcome assessment (detection bias)	Low risk	"Staff were blind with respect to the medication".
Incomplete outcome data (attrition bias)	High risk	A large number of subjects dropped the study that was not managed by appropriate analysis.
Selective reporting (reporting bias)	High risk	The study protocol could not be found and several relevant outcomes are not reported.
Other bias	High risk	No sample size calculation was performed and the employed sample size is small.
**Carette 1986**	**Risk of Bias**	**Reason/Quotation**
Random sequence generation (selection bias)	Unclear risk	The method of random sequence generation was not mentioned.
Allocation concealment (selection bias)	Unclear risk	The method of allocation concealment was not mentioned.
Blinding of participants and personnel (performance bias)	High risk	"The first problem was that of maintaining the double-blind nature of the trial, since such a high proportion (70%) of the amitriptyline patients developed anticholinergic side effects, which in some cases unblinded both patients and observers".
Blinding of outcome assessment (detection bias)	High risk	
Incomplete outcome data (attrition bias)	Low risk	More than the estimated sample size at the start was enrolled. Therefore, the original power was preserved throughout.
Selective reporting (reporting bias)	High risk	The study protocol could not be found and several relevant outcomes are not reported.
Other bias	Unclear risk	No sufficient information to assess whether other sources of bias existed.
**Carette 1995a**	**Risk of Bias**	**Reason/Quotation**
Random sequence generation (selection bias)	Low risk	The order of treatment (amitriptyline first or placebo first) was generated using a table of random numbers.
Allocation concealment (selection bias)	Unclear risk	The method of allocation concealment was not mentioned.

© 2022 Farag HM et al. *JAMA Network Open.*

Blinding of participants and personnel (performance bias)	Low risk	"We used a double-blind, crossover trial design of 8 weeks' duration".
Blinding of outcome assessment (detection bias)	Low risk	"Paper speed was 15 mm/second, and all records were scored blindly in 40-second epochs according to standard criteria".
Incomplete outcome data (attrition bias)	Low risk	No significant patient attrition occurred during the study.
Selective reporting (reporting bias)	Low risk	Relevant outcomes were adequately reported.
Other bias	High risk	No washout period was instituted between the two 8-week treatment periods. Moreover, no sample size calculation was performed and the employed sample size is small.
**Carette 1995b**	**Risk of Bias**	**Reason/Quotation**
Random sequence generation (selection bias)	Low risk	"The assignment scheme was generated using a table of random numbers. Patients were assigned to each participating clinic in blocks of 5"
Allocation concealment (selection bias)	Unclear risk	The method of allocation concealment was not mentioned.
Blinding of participants and personnel (performance bias)	Low risk	"were entered into a 6-month prospective, double-blind, multicenter trial"
Blinding of outcome assessment (detection bias)	Low risk	"were entered into a 6-month prospective, double-blind, multicenter trial"
Incomplete outcome data (attrition bias)	Low risk	Two types of analysis were performed: an analysis based on the results for all patients entered into the trial (intent-to-treat)
Selective reporting (reporting bias)	Low risk	Relevant outcomes were adequately reported.
Other bias	Unclear risk	No sufficient information to assess whether other sources of bias existed.
**Chappell et al. 2008**	**Risk of Bias**	**Reason/Quotation**

© 2022 Farag HM et al. *JAMA Network Open.*

Random sequence generation (selection bias)	Low risk	"Assignment to treatment groups was determined by a computer-generated random sequence within each study center, stratified by MDD status (yes, no)"
Allocation concealment (selection bias)	Unclear risk	The method of allocation concealment was not mentioned.
Blinding of participants and personnel (performance bias)	Low risk	"Following the one-week screening phase, patients were treated in a double-blind manner for 27 weeks".
Blinding of outcome assessment (detection bias)	Low risk	"The CGI-Severity is administered by a study physician (blinded to study treatment) in the presence of the patient".
Incomplete outcome data (attrition bias)	Low risk	"All analyses were conducted on an intent-to-treat (ITT) basis".
Selective reporting (reporting bias)	Low risk	Relevant outcomes were adequately reported.
Other bias	Low risk	No other sources of bias could be detected.
**Chappell et al. 2009**	**Risk of Bias**	**Reason/Quotation**
Random sequence generation (selection bias)	Low risk	"Stratification and randomization of patients was conducted using an interactive voice response system that was accessed via telephone by each investigator".
Allocation concealment (selection bias)	High risk	
Blinding of participants and personnel (performance bias)	Low risk	During the second phase of the study, both patients and assessing physicians were blinded to the treatment group.
Blinding of outcome assessment (detection bias)	Low risk	During the second phase of the study, both patients and assessing physicians were blinded to the treatment group.
Incomplete outcome data (attrition bias)	Low risk	"All analyses were conducted using the principles of intent-to-treat. All enrolled (randomized) patients were included in safety assessments for applicable study periods, whereas all enrolled (randomized) patients with at least 1 post-baseline observation were included in efficacy and some safety assessments".
Selective reporting (reporting bias)	Low risk	Relevant outcomes were adequately reported.
Other bias	High risk	"The secondary (efficacy) findings should be viewed as exploratory, as the power to demonstrate persistence of

© 2022 Farag HM et al. *JAMA Network Open.*

		efficacy or a clinically meaningful gain in efficacy associated with a dose increase is likely to be low, particularly given the large improvements seen during the open-label study phase. In addition, there were differences in the levels of respective individual measures at the start of the double-blind phase".
**Clauw et al. 2008**	**Risk of Bias**	**Reason/Quotation**
Random sequence generation (selection bias)	Low risk	"Randomization lists for each site were generated by a computer program (SCIREX Corporation, Hartford, Connecticut)".
Allocation concealment (selection bias)	Low risk	"Randomization assignments were made via an interactive voice response system, with electronic copies of the randomization assignments kept in a secured location".
Blinding of participants and personnel (performance bias)	Low risk	"Clinical staff, investigators, patients, and the study sponsor were blinded to treatment allocation".
Blinding of outcome assessment (detection bias)	Low risk	"Clinical staff, investigators, patients, and the study sponsor were blinded to treatment allocation".
Incomplete outcome data (attrition bias)	Low risk	"Assuming a binary response rate for placebo of 19% for the composite end point of pain associated with FM and response rates for both doses of milnacipran of ~28% to 29% (intent-to treat [ITT] population)".
Selective reporting (reporting bias)	Low risk	Relevant outcomes were adequately reported.
Other bias	Low risk	No other sources of bias could be detected.
**Fors et al. 2002**	**Risk of Bias**	**Reason/Quotation**
Random sequence generation (selection bias)	Low risk	Although patients chose their preferred medication, they were not informed with the nature of either medication.
Allocation concealment (selection bias)	High risk	"They then blindly chose either amitriptyline or placebo, which had been prepared in identical capsules by the hospital pharmacist".
Blinding of participants and personnel (performance bias)	Low risk	"They then blindly chose either amitriptyline or placebo, which had been prepared in identical capsules by the hospital pharmacist".

© 2022 Farag HM et al. *JAMA Network Open.*

Blinding of outcome assessment (detection bias)	Low risk	"Assignment was thus double blind".
Incomplete outcome data (attrition bias)	Unclear risk	No sufficient information to assess whether attrition bias existed.
Selective reporting (reporting bias)	High risk	Several relevant outcomes were not adequately reported in this study.
Other bias	High risk	No sample size calculation was performed and the employed sample size is small.
**Gendreau et al. 2005/Vitton et al. 2004**	**Risk of Bias**	**Reason/Quotation**
Random sequence generation (selection bias)	Low risk	"Randomization was performed by an independent contract research organization that generated randomization assignments and packaged drug in a block size of 8, in a ratio of 3:3:2 for QD: BID: placebo".
Allocation concealment (selection bias)	Low risk	"An automated telephone response system operated by the same firm performed the patient treatment assignments using the previously generated randomization table".
Blinding of participants and personnel (performance bias)	Low risk	"All capsules were visually identical, and patients and investigators remained blinded to patients' treatment allocation".
Blinding of outcome assessment (detection bias)	Low risk	
Incomplete outcome data (attrition bias)	Low risk	"Last observation carried forward coupled with an intent-to-treat approach was used in all analyses other than the completer analyses".
Selective reporting (reporting bias)	High risk	Several relevant outcomes were not adequately reported in this study.
Other bias	Low risk	No other sources of bias could be detected.
**Ginsberg et al. 1996**	**Risk of Bias**	**Reason/Quotation**
Random sequence generation (selection bias)	Unclear risk	The method of random sequence generation was not mentioned.

© 2022 Farag HM et al. *JAMA Network Open.*

Allocation concealment (selection bias)	Unclear risk	The method of allocation concealment was not mentioned.
Blinding of participants and personnel (performance bias)	Low risk	Both drug capsules were identical
Blinding of outcome assessment (detection bias)	Unclear risk	It is unclear whether physicians were blinded during outcome evaluation.
Incomplete outcome data (attrition bias)	Low risk	Adequate intention to treat analysis was performed.
Selective reporting (reporting bias)	High risk	Only one relevant outcome was reported in the study.
Other bias	Unclear risk	No sufficient information to assess whether other sources of bias existed.
**Goldenberg et al. 1986**	**Risk of Bias**	**Reason/Quotation**
Random sequence generation (selection bias)	Unclear risk	"Each patient was randomly assigned to 1 of 4 treatment groups using a method that assured balanced assignment".
Allocation concealment (selection bias)	Unclear risk	The method of allocation concealment was not mentioned.
Blinding of participants and personnel (performance bias)	Low risk	"Patients, as well as the examining physician, were “blinded” as to specific treatment".
Blinding of outcome assessment (detection bias)	Low risk	"Patients, as well as the examining physician, were “blinded” as to specific treatment".
Incomplete outcome data (attrition bias)	Low risk	All randomized patients' data were considered for the analysis.
Selective reporting (reporting bias)	Low risk	Several relevant outcomes were reported in the published article.
Other bias	Unclear risk	No sufficient information to assess whether other sources of bias existed.
**Goldenberg et al. 2010/Mease et al. 2009**	**Risk of Bias**	**Reason/Quotation**
Random sequence generation (selection bias)	Unclear risk	The method of random sequence generation was not mentioned.
Allocation concealment (selection bias)	Unclear risk	The method of allocation concealment was not mentioned.

© 2022 Farag HM et al. *JAMA Network Open.*

Blinding of participants and personnel (performance bias)	Low risk (at least in the initial main study)	The nature of the blinding was such that patients were not informed of their treatment assignments during the 6 months of the lead-in study. During the 6-month extension period (this study), patients were informed that they would receive milnacipran but remained blinded to the dose they would receive.
Blinding of outcome assessment (detection bias)	Low risk	This study was a 27-week, randomized, double-blind, placebo-controlled comparison of 2 doses of milnacipran
Incomplete outcome data (attrition bias)	Low risk	"All patients who received at least 1 dose of study medication were included in the intent-to-treat (ITT) analysis".
Selective reporting (reporting bias)	Low risk	Several relevant outcomes were reported in the published original and extension studies.
Other bias	Low risk	No other sources of bias could be detected.
**Hannonen et al. 1986**	**Risk of Bias**	**Reason/Quotation**
Random sequence generation (selection bias)	Low risk	"The randomization was organized centrally with sequentially numbered envelopes consisting of blocks of six".
Allocation concealment (selection bias)	Low risk	
Blinding of participants and personnel (performance bias)	Low risk	The study was double-blind. "The placebo capsule was identical to the active drugs".
Blinding of outcome assessment (detection bias)	Low risk	The physicians involved in outcome assessment were blinded to the treatment assignments.
Incomplete outcome data (attrition bias)	High risk	A substantial portion of patients withdrawn he study and were not managed by appropriate analysis.
Selective reporting (reporting bias)	Low risk	Relevant efficacy and safety outcomes were adequately reported.
Other bias	Unclear risk	No sufficient information to assess whether other sources of bias existed.
**Heymann et al. 2001**	**Risk of Bias**	**Reason/Quotation**

© 2022 Farag HM et al. *JAMA Network Open.*

Random sequence generation (selection bias)	Low risk	"The randomization tables were supplied by the same laboratory".
Allocation concealment (selection bias)	Unclear risk	The method of allocation concealment was not mentioned.
Blinding of participants and personnel (performance bias)	Low risk	Amitriptyline, nortriptyline and placebo were all prepared by Sandoz Laboratory in standard packages for all groups, thus guaranteeing that the study remained blinded both for the investigator and the patients.
Blinding of outcome assessment (detection bias)	Low risk	
Incomplete outcome data (attrition bias)	High risk	About 10% of patients were lost; were not handled by appropriate analysis and the placebo group size became lower than that recommended statistically to reach significance.
Selective reporting (reporting bias)	High risk	Several relevant outcomes were not adequately reported in this study.
Other bias	High risk	The short treatment period and small size of placebo group could have abolished the significant difference compared to other groups.
**Kempenaers et al. 1994**	**Risk of Bias**	**Reason/Quotation**
Random sequence generation (selection bias)	Unclear risk	The method of random sequence generation was not reported.
Allocation concealment (selection bias)	Unclear risk	The method of allocation concealment was not reported.
Blinding of participants and personnel (performance bias)	Low risk	The study is double-blind and there is no indication that the blinding was broken.
Blinding of outcome assessment (detection bias)	Low risk	The study is double-blind and there is no indication that the blinding was broken.
Incomplete outcome data (attrition bias)	High risk	13/36 patients withdrew the study and were not managed by appropriate analysis.
Selective reporting (reporting bias)	High risk	Several relevant outcomes were not adequately reported in this study.
Other bias	High risk	Small sample size/ No adequate sample size calculation was performed.

© 2022 Farag HM et al. *JAMA Network Open.*

Mease et al. 2008	Risk of Bias	Reason/Quotation
Random sequence generation (selection bias)	Unclear risk	The method of random sequence generation was not reported.
Allocation concealment (selection bias)	Unclear risk	The method of allocation concealment was not reported.
Blinding of participants and personnel (performance bias)	Low risk	"This randomized, double-blind, placebo controlled trial was conducted at 79 research sites in the US".
Blinding of outcome assessment (detection bias)	Low risk	
Incomplete outcome data (attrition bias)	Low risk	All randomized patients' data were entered in the safety and efficacy analyses
Selective reporting (reporting bias)	Low risk	Relevant efficacy and safety outcomes were adequately reported.
Other bias	Low risk	No other sources of bias could be detected.
**Murakami et al. 2015**	**Risk of Bias**	**Reason/Quotation**
Random sequence generation (selection bias)	Low risk	"After the screening phase, patients were assigned randomly to receive duloxetine or placebo in a 1:1 ratio, using a web-based patient registration system (ACRONET Corp., Tokyo, Japan) with a stochastic minimization procedure".
Allocation concealment (selection bias)	High risk	"Only the drug allocation controller was aware of the type of drugs being dispensed".
Blinding of participants and personnel (performance bias)	Low risk	"Duloxetine or placebo was orally administered once daily after breakfast on a double-blind basis. The drug allocation controller confirmed the study drugs were undiscernible in terms of appearance, packaging, and labeling, and mock titration of placebo pills was also performed to maintain blinding".
Blinding of outcome assessment (detection bias)	Low risk	
Incomplete outcome data (attrition bias)	Low risk	All randomized patients' data were entered in the safety and efficacy analyses
Selective reporting (reporting bias)	Low risk	All registered outcomes on the protocol (NCT01552057) were reported.
Other bias	Low risk	No other sources of bias could be detected.

© 2022 Farag HM et al. *JAMA Network Open.*

Ohta et al. 2012	Risk of Bias	Reason/Quotation
Random sequence generation (selection bias)	Low risk	"Patients were registered to the randomization control system (IMPALA), which provided subject randomization numbers".
Allocation concealment (selection bias)	Unclear risk	The method of allocation concealment was not reported.
Blinding of participants and personnel (performance bias)	Low risk	This was a randomized, double-blind, multicenter, placebo-controlled trial to compare the efficacy and safety of pregabalin vs. placebo in patients with FM. Pregabalin and identical placebo capsules were prescribed by the investigator using blinded drug numbers issued by IMPALA.
Blinding of outcome assessment (detection bias)	Low risk	
Incomplete outcome data (attrition bias)	Low risk	All randomized patients' data were entered in the safety and efficacy analyses
Selective reporting (reporting bias)	Low risk	All registered outcomes on the protocol (NCT00830167) were reported.
Other bias	Low risk	No other sources of bias could be detected.

Pauer et al. 2011	Risk of Bias	Reason/Quotation
Random sequence generation (selection bias)	Unclear risk	The method of random sequence generation was not reported.
Allocation concealment (selection bias)	Unclear risk	The method of allocation concealment was not reported.
Blinding of participants and personnel (performance bias)	Low risk	"This randomized, double-blind, placebo-controlled monotherapy trial was conducted at 73 centers in Europe".
Blinding of outcome assessment (detection bias)	Low risk	
Incomplete outcome data (attrition bias)	Low risk	All randomized patients' data were entered in the safety and efficacy analyses
Selective reporting (reporting bias)	Low risk	All registered outcomes on the protocol (NCT00333866) were reported.
Other bias	Unclear risk	No sufficient information to assess whether other sources of bias existed.

© 2022 Farag HM et al. *JAMA Network Open.*

Pickering et al. 2018	Risk of Bias	Reason/Quotation
Random sequence generation (selection bias)	Low risk	"Treatment allocation followed a predefined randomization plan and was conducted by a person independent from the protocol. The randomization sequence was generated using random blocks".
Allocation concealment (selection bias)	Low risk	
Blinding of participants and personnel (performance bias)	Low risk	"This study is a prospective, randomized, controlled double-blind clinical trial with 2 parallel groups".
Blinding of outcome assessment (detection bias)	Low risk	
Incomplete outcome data (attrition bias)	Low risk	"No missing data occurred in this work".
Selective reporting (reporting bias)	Low risk	All registered outcomes on the protocol (NCT00333866) were reported.
Other bias	Low risk	No other sources of bias could be detected.
**Russel et al. 2008**	**Risk of Bias**	**Reason/Quotation**
Random sequence generation (selection bias)	Low risk	"Assignment to treatment groups was determined by a computer-generated random sequence and each stratum (depressed and non-depressed) was randomly assigned within sites to achieve a relative balance across treatments".
Allocation concealment (selection bias)	Unclear risk	The method of allocation concealment was not reported.
Blinding of participants and personnel (performance bias)	Low risk	"There was a 1-week screening phase, which was followed by 3-month (15 weeks) double-blind, randomized treatment with duloxetine (20 mg/day, 60 mg/day, or 120 mg/day) or placebo once daily, for evaluation of the primary endpoint. All remaining patients continued for an additional 13 weeks of double-blind, placebo-controlled treatment".
Blinding of outcome assessment (detection bias)	Low risk	
Incomplete outcome data (attrition bias)	Low risk	"All analyses were conducted on an intent-to-treat basis unless otherwise specified".
Selective reporting (reporting bias)	Low risk	Relevant efficacy and safety outcomes were adequately reported.

© 2022 Farag HM et al. *JAMA Network Open.*

Other bias	High risk	"The lack of significance between treatment groups in some safety measures could be attributed to the study not being sufficiently powered to detect these differences".
**Staud et al. 2015**	**Risk of Bias**	**Reason/Quotation**
Random sequence generation (selection bias)	Low risk	"Subjects who fulfilled the 1990 ACR Criteria for FM were randomized to either 50 mg milnacipran or identically looking placebo tablets twice daily using Research Randomizer (http://www.randomizer.org/)".
Allocation concealment (selection bias)	Low risk	
Blinding of participants and personnel (performance bias)	Low risk	"Double-blind, randomized, placebo controlled over six weeks"
Blinding of outcome assessment (detection bias)	Low risk	Investigators and research subjects remained unaware of study drug allocation during the whole trial.
Incomplete outcome data (attrition bias)	Low risk	All patients allocated to the treatment arms were analyzed.
Selective reporting (reporting bias)	Low risk	Both registered outcomes in the protocol were reported in the published study (NCT01294059).
Other bias	Unclear risk	No sufficient information to assess whether other sources of bias existed.

© 2022 Farag HM et al. *JAMA Network Open.*

eTable 3B. Risk of bias summary

We used the Cochrane Collaboration's tool for assessing risk of bias.

Risk of bias summary: it is a summary table of review author's judgments for each risk of bias item for each study

	Staud et al. 2015	Russel et al. 2008	Pickering et al. 2018	Pauer et al. 2011	Ohta et al. 2012	Murakami et al. 2015	Mease et al. 2008	Kempenaers et al. 1994	Heymann et al. 2001	Hannonen et al. 1986	Goldenberg et al. 2010/Mease et al. 2009	Goldenberg et al. 1986	Ginsberg et al. 1996	Gendreau et al. 2005/Vitton et al. 2004	Fors et al. 2002	Crofford et al. 2005/Arnold et al. 2007	Clauw et al. 2008	Chappell et al. 2009	Chappell et al. 2008	Carette et al. 1995b	Carette et al. 1995a	Carette et al. 1986	Braz et al. 2013	Branco et al. 2011	Branco et al. 2010	Arnold et al. 2015	Arnold et al. 2012	Arnold et al. 2010b	Arnold et al. 2010a	Arnold et al. 2008	Arnold et al. 2005	Arnold et al. 2004	Ahmed et al. 2016
Random sequence generation (selection bias)	+	+	+	?	+	+	?	?	+	+	?	?	?	+	+	+	+	+	+	+	+	?	?	?	?	?	+	+	+	+	?	+	+
Allocation concealment (selection bias)	+	?	+	?	?	−	?	?	?	+	?	?	?	+	−	?	+	−	?	?	?	?	?	?	?	?	+	+	+	+	?	+	+
Blinding of participants and personnel (performance bias)	+	+	+	+	+	+	+	+	+	+	+	+	+	+	+	+	+	+	+	+	+	−	+	+	+	+	+	+	+	+	+	+	+
Blinding of outcome assessment (detection bias)	+	+	+	+	+	+	+	+	+	+	+	+	?	+	+	+	+	+	+	+	+	−	+	+	+	?	+	+	+	+	+	+	+
Incomplete outcome data (attrition bias)	+	+	+	+	+	+	+	−	−	−	+	+	+	+	?	+	+	+	+	+	+	+	−	+	+	+	+	+	+	+	+	+	+
Selective reporting (reporting bias)	+	+	+	+	+	+	+	−	−	+	+	+	−	?	−	−	+	+	+	+	+	−	−	+	+	?	+	+	+	+	+	+	+
Other bias	?	−	+	?	+	+	+	−	−	?	+	?	?	+	−	−	+	−	+	?	−	?	−	+	?	?	+	+	?	+	+	+	+

+ Low risk of bias

? Unclear risk of bias

− High risk of bias

© 2022 Farag HM et al. *JAMA Network Open.*

eTable 3C. Risk of bias graph

Risk of bias graph: it is a plot of the distribution of judgments (Yes, No, Unclear) across studies for each risk of bias item

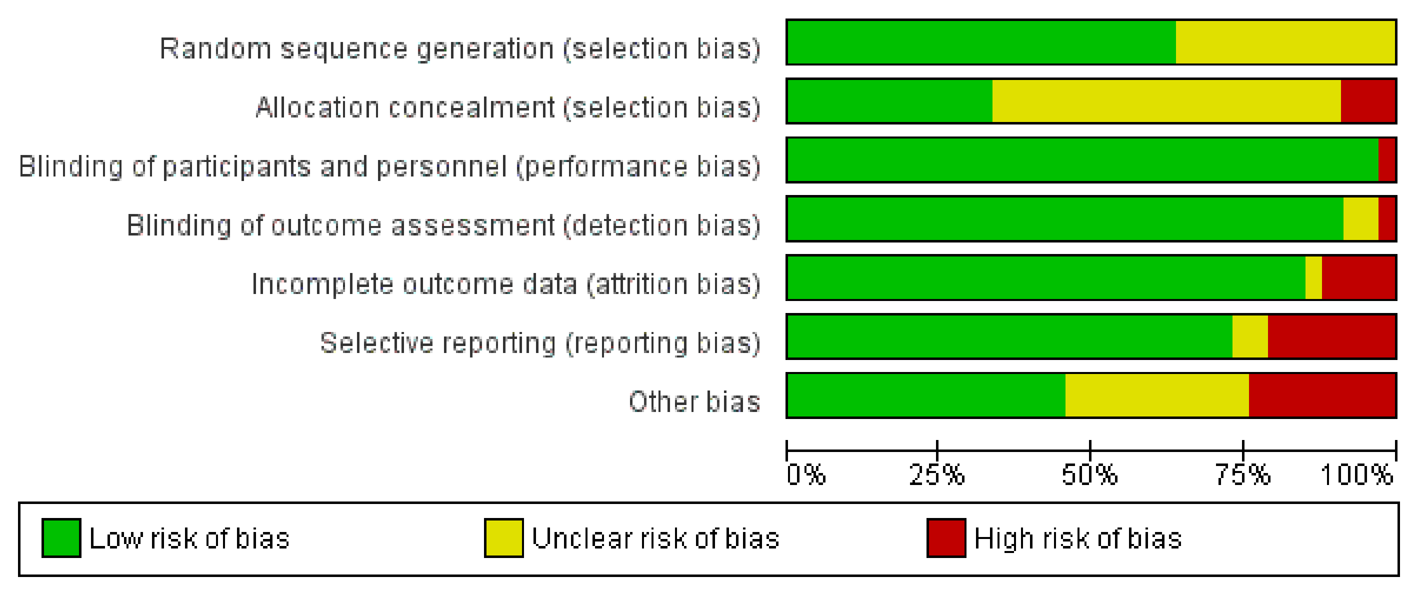

We found the overall quality of studies to be good, although around two thirds of the studies did not report the details about the method of allocation concealment.

© 2022 Farag HM et al. *JAMA Network Open.*

eTable 4. Assessments of Inconsistencies

eTable 4A. Assessment of inconsistency for the pain outcome

eTable 4A1. Inconsistency Test Based on Loop-Specific Approach

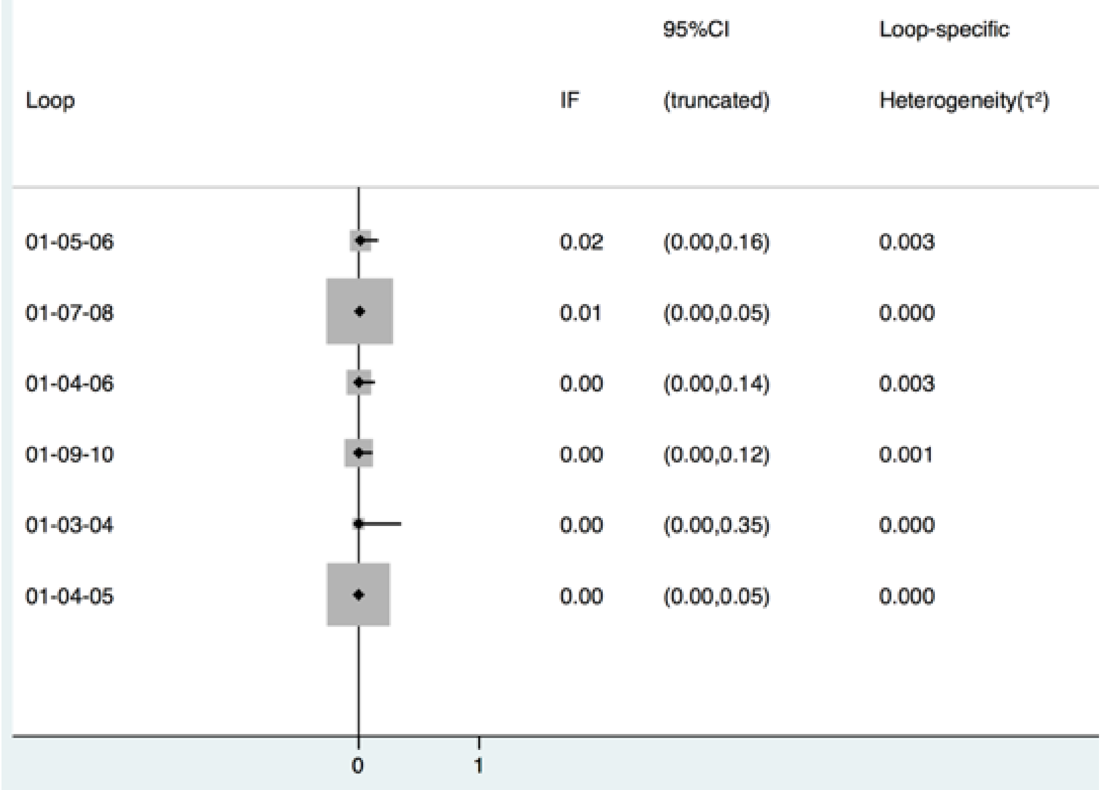

Inconsistency plot for the fibromyalgia treatments network showing for each loop the ratio of standardized mean differences between direct and indirect estimates. The within-loop heterogeneities have been estimated using the method of moments estimator. A p-value more than >0.1; 1: **Placebo** 2: **Amitriptyline** 3: **Pregabalin 150mg** 4: **Pregabalin 300mg** 5: **Pregabalin 450mg** 6: **Pregabalin 600mg** 7: **Duloxetine 60mg** 8: **Duloxetine 120mg** 9: **Milnacipran 100mg** 10: **Milnacipran 200mg.**

© 2022 Farag HM et al. *JAMA Network Open.*

eTable 4A1. Inconsistency Test Based on Loop-Specific Approach (continued)

Loop	IF	seIF	z_value	p_value	CI_95	Loop_Heterog_tau2
01-05-06	0.016	0.075	0.217	0.828	(0.00,0.16)	0.003
01-07-08	0.011	0.018	0.606	0.544	(0.00,0.05)	0.000
01-04-06	0.004	0.068	0.054	0.957	(0.00,0.14)	0.003
01-09-10	0.002	0.058	0.042	0.967	(0.00,0.12)	0.001
01-03-04	0.001	0.180	0.007	0.995	(0.00,0.35)	0.000
01-04-05	0.000	0.025	0.011	0.991	(0.00,0.05)	0.000
04-05-06	.	.	.	.		0.001

1: **Placebo** 2: **Amitriptyline** 3: **Pregabalin 150mg** 4: **Pregabalin 300mg** 5: **Pregabalin 450mg** 6: **Pregabalin 600mg** 7: **Duloxetine 60mg** 8: **Duloxetine 120mg** 9: **Milnacipran 100mg** 10: **Milnacipran 200mg.**

eTable 4A1. Inconsistency Test Based on Loop-Specific Approach with tau= 0.02 (continued)

© 2022 Farag HM et al. *JAMA Network Open.*

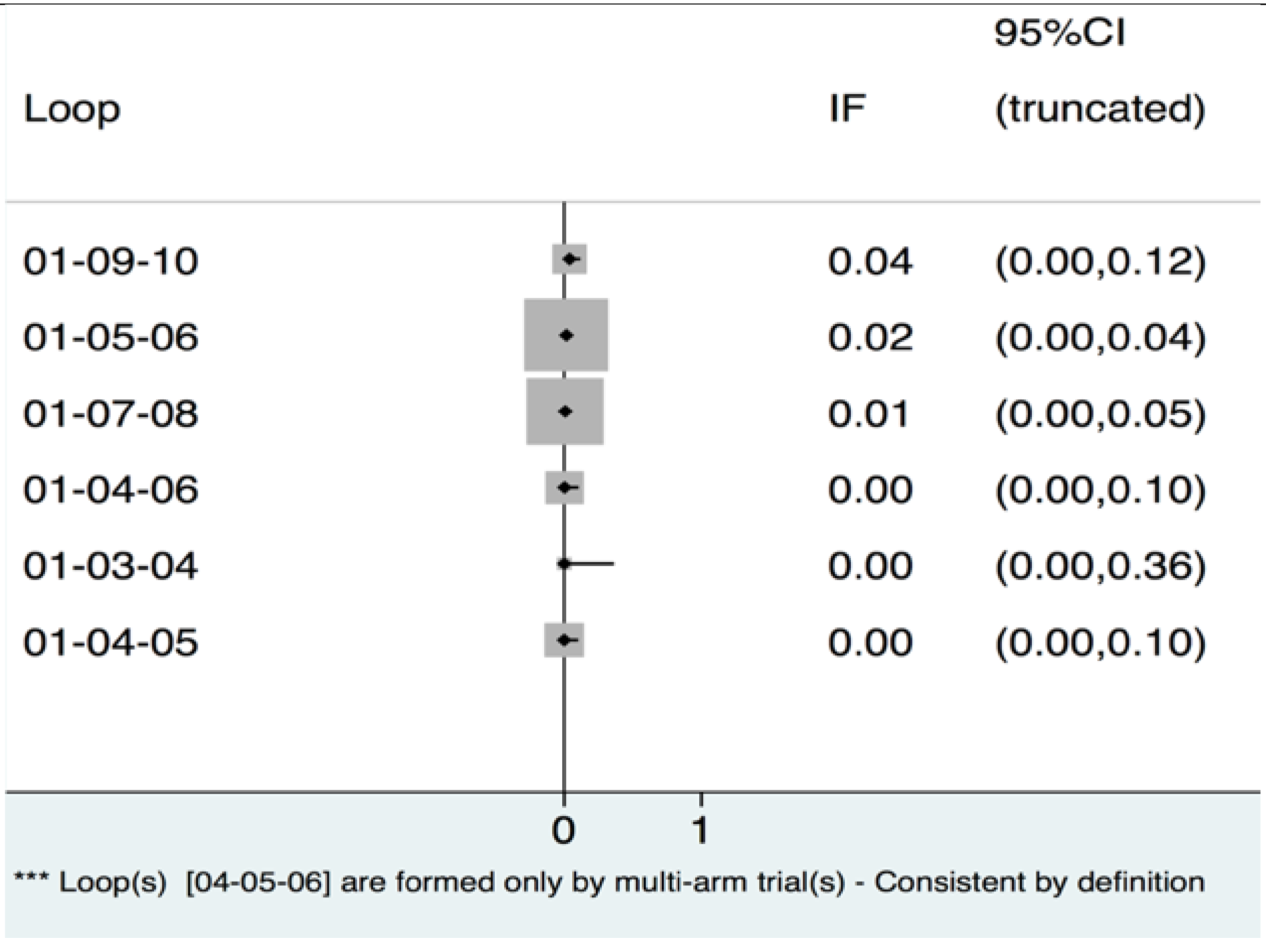

Inconsistency plot for the fibromyalgia treatments network showing for each loop the ratio of standardized mean differences between direct and indirect estimates. The within-loop heterogeneities have been estimated using the method of moments estimator. 1: **Placebo** 2: **Amitriptyline** 3: **Pregabalin 150mg** 4: **Pregabalin 300mg** 5: **Pregabalin 450mg** 6: **Pregabalin 600mg** 7: **Duloxetine 60mg** 8: **Duloxetine 120mg** 9: **Milnacipran 100mg** 10: **Milnacipran 200mg.**

eTable 4A1. Inconsistency Test Based on Loop-Specific Approach with tau= 0.02 (continued)

© 2022 Farag HM et al. *JAMA Network Open.*

Loop	IF	seIF	z_value	p_value	CI_95
01-09-10	0.039	0.040	0.980	0.327	(0.00,0.12)
01-05-06	0.016	0.014	1.158	0.247	(0.00,0.04)
01-07-08	0.008	0.020	0.414	0.679	(0.00,0.05)
01-04-06	0.003	0.051	0.065	0.948	(0.00,0.10)
01-03-04	0.001	0.185	0.006	0.995	(0.00,0.36)
01-04-05	0.001	0.051	0.014	0.989	(0.00,0.10)
04-05-06	.	.	.	.	

1: **Placebo** 2: **Amitriptyline** 3: **Pregabalin 150mg** 4: **Pregabalin 300mg** 5: **Pregabalin 450mg** 6: **Pregabalin 600mg** 7: **Duloxetine 60mg** 8: **Duloxetine 120mg** 9: **Milnacipran 100mg** 10: **Milnacipran 200mg.**

© 2022 Farag HM et al. *JAMA Network Open.*

eTable 4A2. Inconsistency Test Based on Side-Splitting Approach

Side		Direct Coef.	Std. Err.	Indirect Coef.	Std. Err.	Difference Coef.	Std. Err.	P>|z|
01 02		.	.	.	.	.	.	.
01 03	*	-.2266246	.0271626	-.2271754	.258155	.0005507	.2595936	0.998
01 04	*	-.2299068	.0131216	-.2607733	.0593956	.0308665	.060888	0.612
01 05	*	-.2951535	.0132123	-.2988991	.0601056	.0037456	.0615271	0.951
01 06	*	-.2727579	.0151094	-.2926708	.04384	.0199129	.0462952	0.667
01 07		-.2352929	.011594	-.2405991	.0534653	.0053062	.0547075	0.923
01 08		-.3334982	.0196588	-.3202179	.0311062	-.0132803	.0366377	0.717
01 09		-.1764225	.0136794	-.1537252	.050022	-.0226973	.0518665	0.662
01 10		-.2232316	.015551	-.2303428	.040257	.0071112	.04346	0.870
03 04		-.004403	.1620843	-.0047443	.0300663	.0003413	.1641597	0.998
04 05	*	-.0622958	.0150987	-.0746494	.0383683	.0123535	.0412322	0.764
04 06	*	-.0436897	.0152076	-.0399441	.0596571	-.0037456	.0615272	0.951
05 06	*	.0185886	.0151186	.0501967	.0597892	-.0316081	.0616335	0.608
07 08		-.0917519	.0181375	-.1038814	.0357449	.0121295	.0398784	0.761
09 10		-.0526535	.0157368	-.0306164	.0368515	-.0220371	.0400309	0.582

1: **Placebo** 2: **Amitriptyline** 3: **Pregabalin 150mg** 4: **Pregabalin 300mg** 5: **Pregabalin 450mg** 6: **Pregabalin 600mg** 7: **Duloxetine 60mg** 8: **Duloxetine 120mg** 9: **Milnacipran 100mg** 10: **Milnacipran 200mg.**

eTables 4B. Assessment of inconsistency for the sleep outcome

eTable 4B1. Inconsistency Test Based on Loop-Specific Approach (results when tau=0.02 are the same)

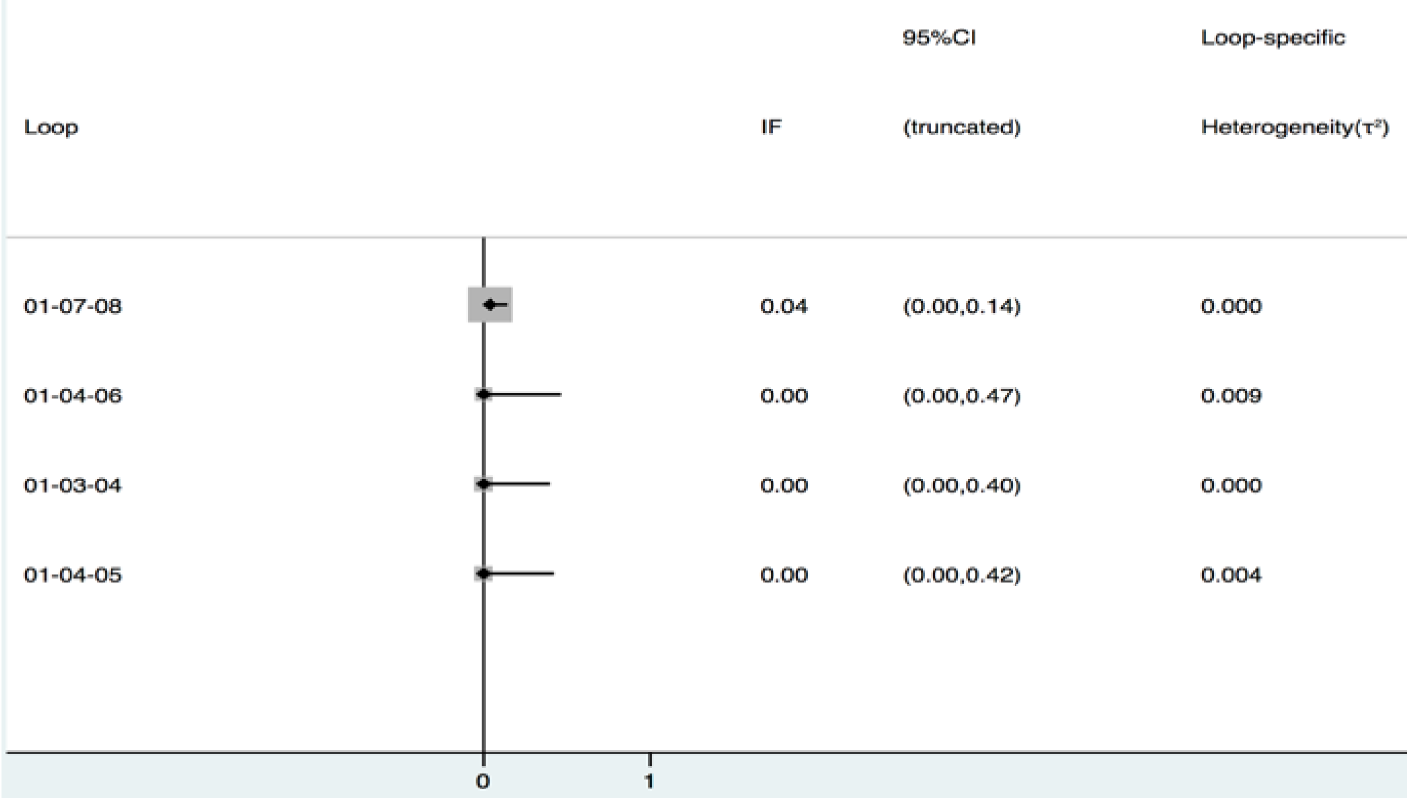

Inconsistency plot for the fibromyalgia treatments network showing for each loop the ratio of standardized mean differences between direct and indirect estimates. The within-loop heterogeneities have been estimated using the method of moments estimator. 1: **Placebo** 2: **Amitriptyline** 3: **Pregabalin 150mg** 4: **Pregabalin 300mg** 5: **Pregabalin 450mg** 6: **Pregabalin 600mg** 7: **Duloxetine 60mg** 8: **Duloxetine 120mg** 9: **Milnacipran 100mg** 10: **Milnacipran 200mg.**

eTable 4B1. Inconsistency Test Based on Loop-Specific Approach (continued)

© 2022 Farag HM et al. *JAMA Network Open.*

Loop	IF	seIF	z_value	p_value	CI_95	Loop_Heterog_tau2
01-07-08	0.039	0.053	0.734	0.463	(0.00,0.14)	0.000
01-04-06	0.000	0.237	0.000	1.000	(0.00,0.47)	0.009
01-03-04	0.000	0.204	0.000	1.000	(0.00,0.40)	0.000
04-05-06	.	.	.	.		0.005
01-05-06	.	.	.	.		0.005
01-04-05	0.000	0.216	0.000	1.000	(0.00,0.42)	0.004

1: **Placebo** 2: **Amitriptyline** 3: **Pregabalin 150mg** 4: **Pregabalin 300mg** 5: **Pregabalin 450mg** 6: **Pregabalin 600mg** 7: **Duloxetine 60mg** 8: **Duloxetine 120mg** 9: **Milnacipran 100mg** 10: **Milnacipran 200mg.**

© 2022 Farag HM et al. *JAMA Network Open.*

eTable 4B2. Inconsistency Test Based on Side-Splitting Approach

Side		Direct Coef.	Std. Err.	Indirect Coef.	Std. Err.	Difference Coef.	Std. Err.	P>|z|
01 02		.	.	.	.	.	.	.
01 03	*	-.5717752	.0564391	-.5711246	.3692102	-.0006506	.3735387	0.999
01 04	*	-.3022641	.0336626	-.3035402	.3818083	.0012761	.3833083	0.997
01 05	*	-.4984223	.0340026	-.4990729	.3719892	.0006506	.3735424	0.999
01 06	*	-.6039403	.034021	-.604591	.371989	.0006506	.3735405	0.999
01 07		.	.	.	.	.	.	.
01 08	*	-.31727	.0694075	-.23881	.17285	-.07846	.1848764	0.671
01 09	*	-.2296359	.0848137	-.0000652	307.182	-.2295707	307.182	0.999
03 04		.2689003	.2094483	.269547	.0676161	-.0006467	.2200862	0.998
04 05	*	-.1961586	.0340036	-.195508	.3719857	-.0006506	.3735392	0.999
04 06	*	-.3016767	.0340219	-.301026	.3719888	-.0006506	.3735405	0.999
05 06		.	.	.	.	.	.	.
07 08	*	-.084	.0695996	-.16246	.1726182	.07846	.1848764	0.671
09 10	*	-.081	.6318398	.3782824	626.9011	-.4592824	626.9007	0.999

1: **Placebo** 2: **Amitriptyline** 3: **Pregabalin 150mg** 4: **Pregabalin 300mg** 5: **Pregabalin 450mg** 6: **Pregabalin 600mg** 7: **Duloxetine 60mg** 8: **Duloxetine 120mg** 9: **Milnacipran 100mg** 10: **Milnacipran 200mg.**

eTables 4C. Assessment of inconsistency for the depression outcome

© 2022 Farag HM et al. *JAMA Network Open.*

eTable 4C1. Inconsistency Test Based on Loop-Specific Approach (results when tau=0.02 are the same)

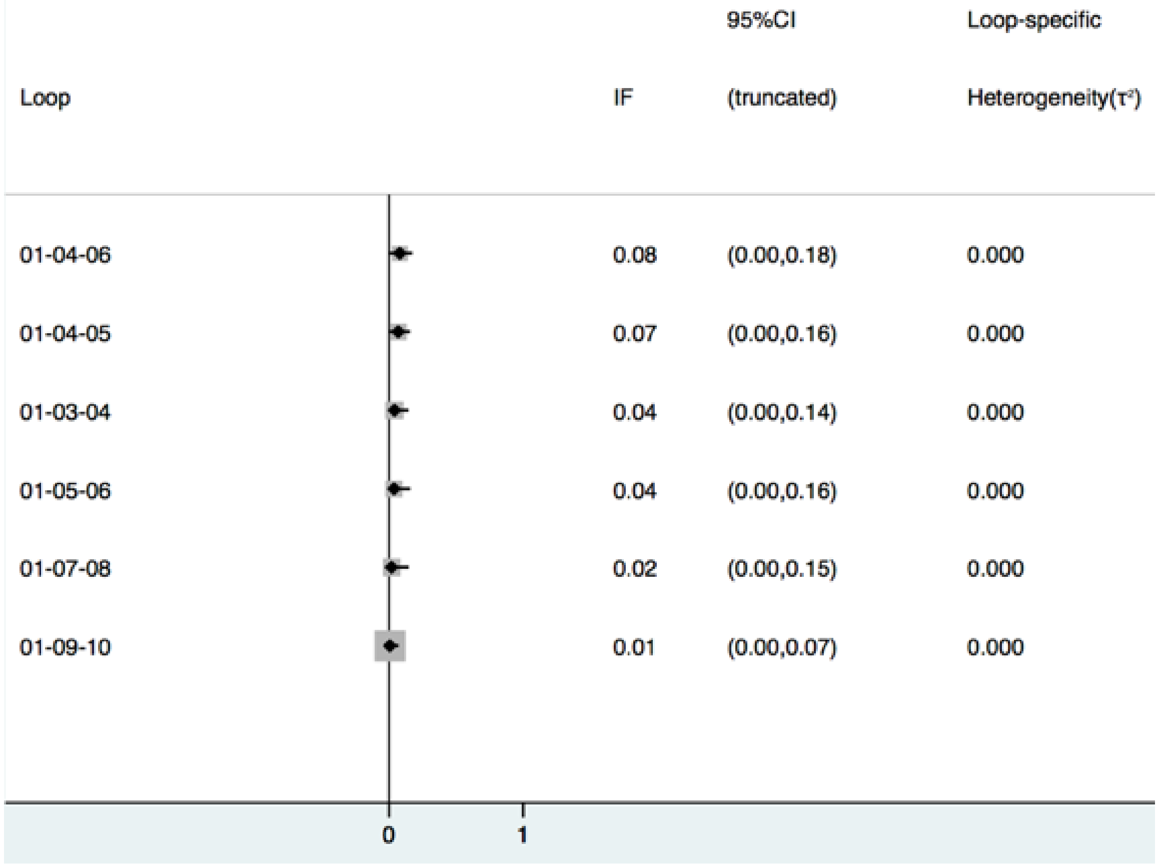

Loop	IF	95%CI (truncated)	Loop-specific Heterogeneity(τ^2)
01-04-06	0.08	(0.00,0.18)	0.000
01-04-05	0.07	(0.00,0.16)	0.000
01-03-04	0.04	(0.00,0.14)	0.000
01-05-06	0.04	(0.00,0.16)	0.000
01-07-08	0.02	(0.00,0.15)	0.000
01-09-10	0.01	(0.00,0.07)	0.000

Inconsistency plot for the fibromyalgia treatments network showing for each loop the ratio of standardized mean differences between direct and indirect estimates. The within-loop heterogeneities have been estimated using the method of moments estimator. 1: **Placebo** 2: **Amitriptyline** 3: **Pregabalin 150mg** 4: **Pregabalin 300mg** 5: **Pregabalin 450mg** 6: **Pregabalin 600mg** 7: **Duloxetine 60mg** 8: **Duloxetine 120mg** 9: **Milnacipran 100mg** 10: **Milnacipran 200mg.**

eTable 4C1. Inconsistency Test Based on Loop-Specific Approach (continued)

© 2022 Farag HM et al. *JAMA Network Open.*

Loop	IF	seIF	z_value	p_value	CI_95	Loop_Heterog_tau2
01-04-06	0.079	0.049	1.626	0.104	(0.00,0.18)	0.000
01-04-05	0.068	0.046	1.458	0.145	(0.00,0.16)	0.000
01-03-04	0.041	0.053	0.767	0.443	(0.00,0.14)	0.000
01-05-06	0.037	0.063	0.588	0.557	(0.00,0.16)	0.000
01-07-08	0.019	0.065	0.293	0.769	(0.00,0.15)	0.000
01-09-10	0.006	0.033	0.194	0.846	(0.00,0.07)	0.000
04-05-06	.	.	.	.		0.000

1: **Placebo** 2: **Amitriptyline** 3: **Pregabalin 150mg** 4: **Pregabalin 300mg** 5: **Pregabalin 450mg** 6: **Pregabalin 600mg** 7: **Duloxetine 60mg** 8: **Duloxetine 120mg** 9: **Milnacipran 100mg** 10: **Milnacipran 200mg.**

© 2022 Farag HM et al. *JAMA Network Open.*

eTable 4C2. Inconsistency Test Based on Side-Splitting Approach

Side		Direct		Indirect		Difference		
		Coef.	Std. Err.	Coef.	Std. Err.	Coef.	Std. Err.	P>|z|
01 02		.	.	.	.	.	.	.
01 03	*	-.0426396	.0130554	-.0964373	.0898472	.0537977	.0906643	0.553
01 04	*	-.2266146	.0183631	-.19545	.0659193	-.0311646	.0681996	0.648
01 05	*	-.122043	.0252456	-.2567812	.0686949	.1347382	.0720051	0.061
01 06	*	-.20317	.0300361	-.3286784	.0604911	.1255084	.0661515	0.058
01 07	*	-.2365427	.0185924	-.2780468	.1946386	.0415041	.195265	0.832
01 08	*	-.2504391	.0393659	-.2164726	.0860527	-.0339665	.0899642	0.706
01 09		-.0981524	.0134946	-.0842261	.0565832	-.0139264	.0579655	0.810
01 10		-.0679025	.0155495	-.0801338	.0504843	.0122313	.0522916	0.815
03 04		-.145461	.0470795	-.1898254	.0238329	.0443644	.0527585	0.400
04 05	*	.0671197	.0305822	.1366758	.0460574	-.069556	.0546443	0.203
04 06	*	-.02311	.0300828	.1116282	.0667792	-.1347382	.0720051	0.061
05 06	*	-.09287	.0320639	-.0625495	.1008088	-.0303205	.1060993	0.775
07 08		.0016863	.0465288	-.0298808	.0643398	.0315671	.0772829	0.683
09 10		.0251388	.0234619	.0331158	.0268671	-.007977	.0356088	0.823

1: **Placebo** 2: **Amitriptyline** 3: **Pregabalin 150mg** 4: **Pregabalin 300mg** 5: **Pregabalin 450mg** 6: **Pregabalin 600mg** 7: **Duloxetine 60mg** 8: **Duloxetine 120mg** 9: **Milnacipran 100mg** 10: **Milnacipran 200mg.**

eTables 4D. Assessment of inconsistency for the fatigue outcome

© 2022 Farag HM et al. *JAMA Network Open.*

eTable 4D1. Inconsistency Test Based on Loop-Specific Approach

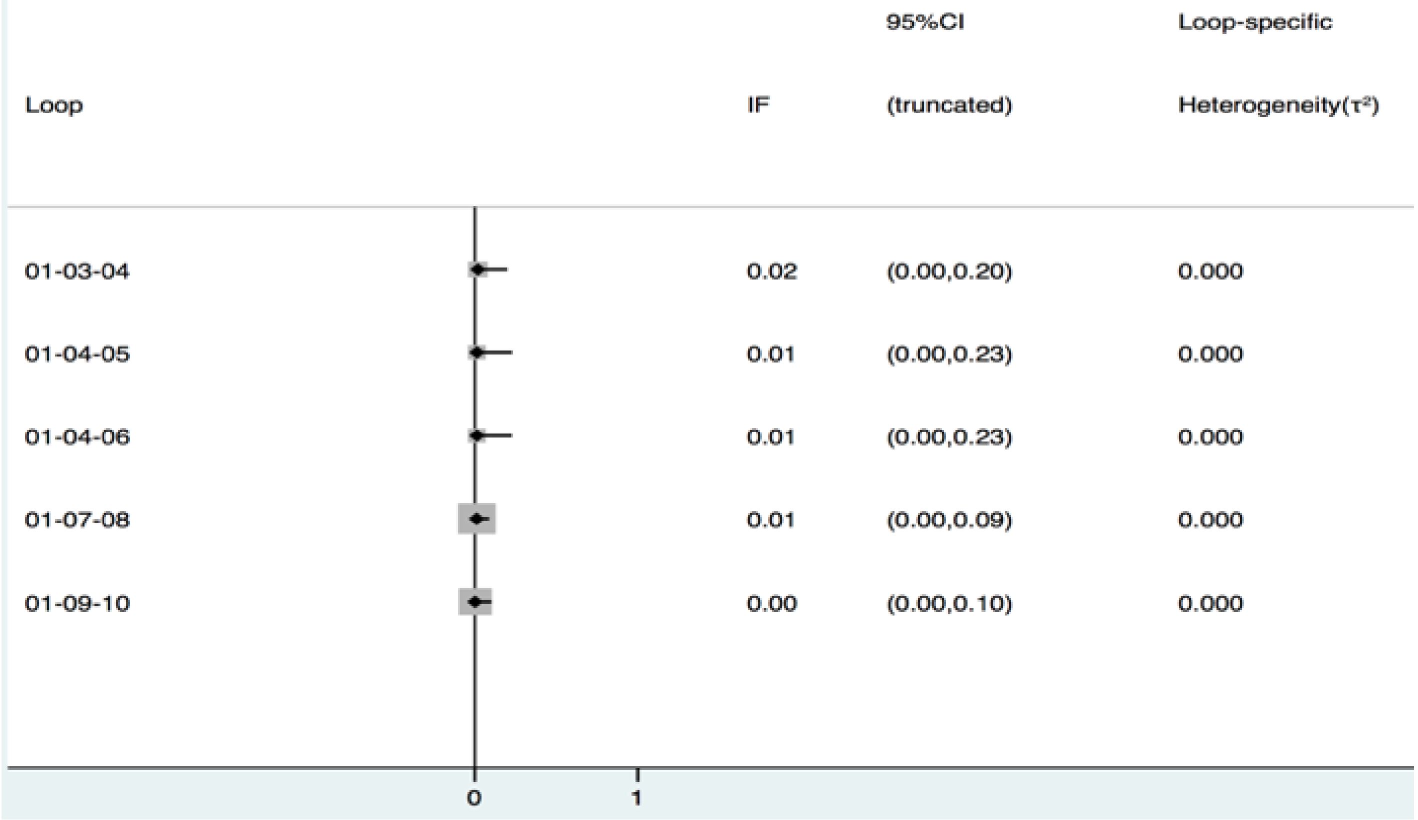

Inconsistency plot for the fibromyalgia treatments network showing for each loop the ratio of standardized mean differences between direct and indirect estimates. The within-loop heterogeneities have been estimated using the method of moments estimator. 1: **Placebo** 2: **Amitriptyline** 3: **Pregabalin 150mg** 4: **Pregabalin 300mg** 5: **Pregabalin 450mg** 6: **Pregabalin 600mg** 7: **Duloxetine 60mg** 8: **Duloxetine 120mg** 9: **Milnacipran 100mg** 10: **Milnacipran 200mg.**

eTable 4D1. Inconsistency Test Based on Loop-Specific Approach (continued)

© 2022 Farag HM et al. *JAMA Network Open.*

Loop	IF	seIF	z_value	p_value	CI_95	Loop_Heterog_tau2
01-03-04	0.018	0.093	0.198	0.843	(0.00,0.20)	0.000
01-04-05	0.013	0.111	0.113	0.910	(0.00,0.23)	0.000
01-04-06	0.013	0.110	0.114	0.909	(0.00,0.23)	0.000
01-07-08	0.011	0.039	0.279	0.781	(0.00,0.09)	0.000
01-09-10	0.001	0.051	0.020	0.984	(0.00,0.10)	0.000
01-05-06	.	.	.	.		0.000
04-05-06	.	.	.	.		0.000

1: **Placebo** 2: **Amitriptyline** 3: **Pregabalin 150mg** 4: **Pregabalin 300mg** 5: **Pregabalin 450mg** 6: **Pregabalin 600mg** 7: **Duloxetine 60mg** 8: **Duloxetine 120mg** 9: **Milnacipran 100mg** 10: **Milnacipran 200mg.**

eTable 4D1. Inconsistency Test Based on Loop-Specific Approach with tau= 0.02 (continued)

© 2022 Farag HM et al. *JAMA Network Open.*

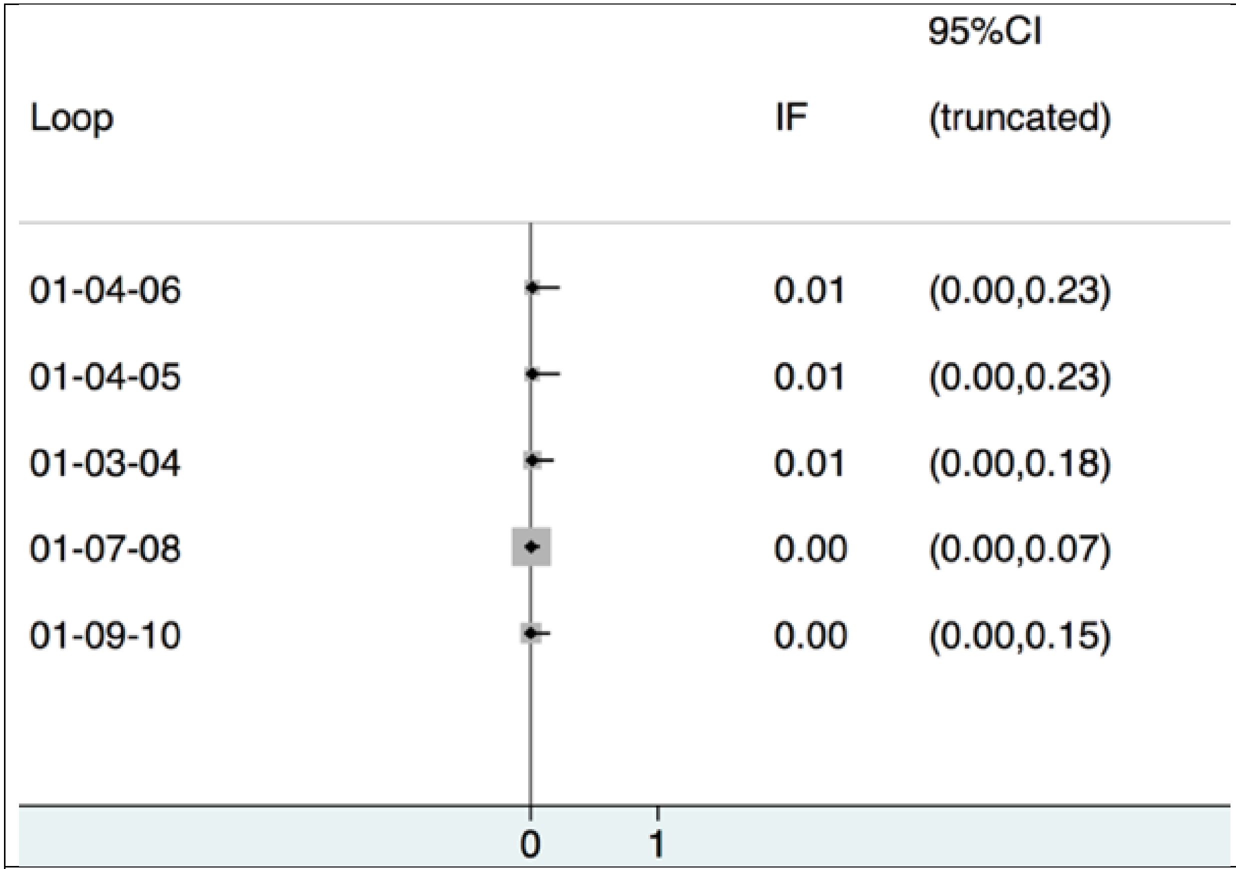

Inconsistency plot for the fibromyalgia treatments network showing for each loop the ratio of standardized mean differences between direct and indirect estimates. The within-loop heterogeneities have been estimated using the method of moments estimator. 1: **Placebo** 2: **Amitriptyline** 3: **Pregabalin 150mg** 4: **Pregabalin 300mg** 5: **Pregabalin 450mg** 6: **Pregabalin 600mg** 7: **Duloxetine 60mg** 8: **Duloxetine 120mg** 9: **Milnacipran 100mg** 10: **Milnacipran 200mg.**

eTable 4D1. Inconsistency Test Based on Loop-Specific Approach with tau= 0.02 (continued)

© 2022 Farag HM et al. *JAMA Network Open.*

Loop	IF	seIF	z_value	p_value	CI_95
01-04-06	0.013	0.110	0.114	0.909	(0.00,0.23)
01-04-05	0.013	0.111	0.113	0.910	(0.00,0.23)
01-03-04	0.012	0.086	0.145	0.885	(0.00,0.18)
01-07-08	0.004	0.034	0.113	0.910	(0.00,0.07)
01-09-10	0.001	0.077	0.012	0.991	(0.00,0.15)
04-05-06	.	.	.	.	
01-05-06	.	.	.	.	

1: **Placebo** 2: **Amitriptyline** 3: **Pregabalin 150mg** 4: **Pregabalin 300mg** 5: **Pregabalin 450mg** 6: **Pregabalin 600mg** 7: **Duloxetine 60mg** 8: **Duloxetine 120mg** 9: **Milnacipran 100mg** 10: **Milnacipran 200mg.**

© 2022 Farag HM et al. *JAMA Network Open.*

eTable 4D2. Inconsistency Test Based on Side-Splitting Approach

Side		Direct		Indirect		Difference		
		Coef.	Std. Err.	Coef.	Std. Err.	Coef.	Std. Err.	P>|z|
01 02		.	.	.	.	.	.	.
01 03	*	-.2653244	.0153235	-.2346052	.173157	-.0307192	.1738228	0.860
01 04	*	-.1724174	.0450097	-.1907157	.123054	.0182982	.1282881	0.887
01 05	*	-.15769	.0598227	-.1884092	.1656356	.0307192	.1738226	0.860
01 06	*	-.24477	.0590358	-.2754892	.1653531	.0307192	.1738227	0.860
01 07	*	-.1254229	.0111487	-.1490229	.0837518	.0236	.0844651	0.780
01 08	*	-.1141561	.0211759	-.1431102	.0554261	.0289542	.0616468	0.639
01 09		-.0955955	.0232704	-.097454	.0867754	.0018585	.0896363	0.983
01 10		-.1682127	.0230158	-.1708427	.0841639	.00263	.0875227	0.976
03 04		.079837	.072855	.0968754	.0548193	-.0170384	.0894762	0.849
04 05	*	.00994	.0605604	.0406592	.1648294	-.0307192	.1738226	0.860
04 06	*	-.07714	.0597832	-.0464208	.1645455	-.0307192	.1738227	0.860
05 06		.	.	.	.	.	.	.
07 08		.003933	.0265238	.0120014	.0331485	-.0080684	.0438981	0.854
09 10		-.0726803	.0281207	-.0727103	.0553105	.00003	.0620932	1.000

1: **Placebo** 2: **Amitriptyline** 3: **Pregabalin 150mg** 4: **Pregabalin 300mg** 5: **Pregabalin 450mg** 6: **Pregabalin 600mg** 7: **Duloxetine 60mg** 8: **Duloxetine 120mg** 9: **Milnacipran 100mg** 10: **Milnacipran 200mg.**

eTables 4E. Assessment of inconsistency for the quality of life outcome.

© 2022 Farag HM et al. *JAMA Network Open.*

eTable 4E1. Inconsistency Test Based on Loop-Specific Approach

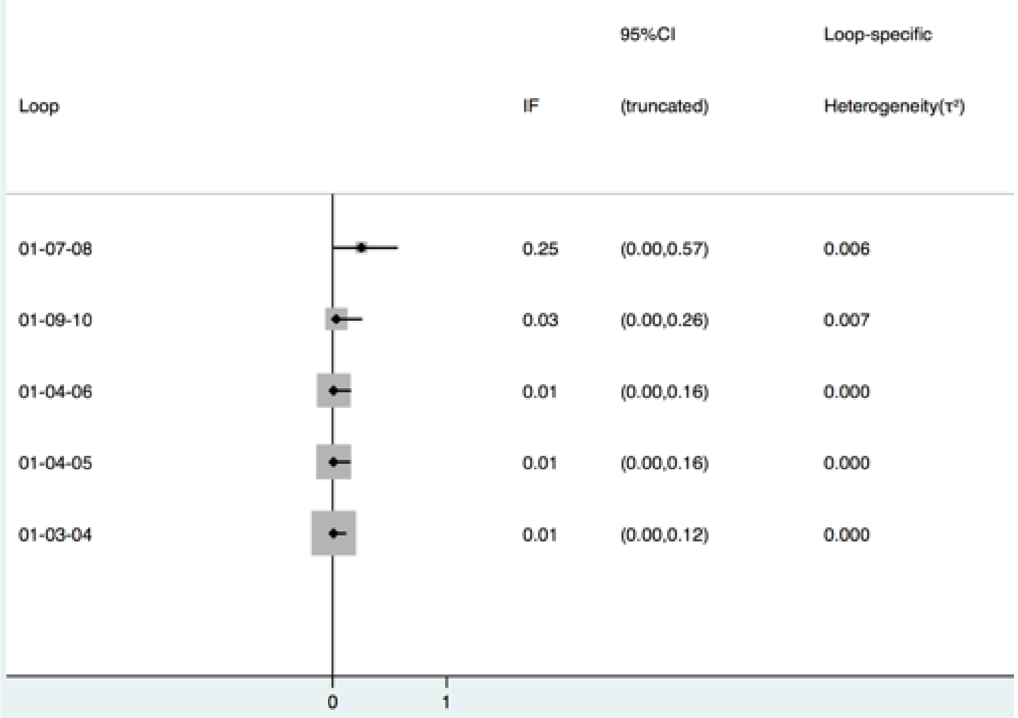

Inconsistency plot for the fibromyalgia treatments network showing for each loop the ratio of standardized mean differences between direct and indirect estimates. The within-loop heterogeneities have been estimated using the method of moments estimator. 1: **Placebo** 2: **Amitriptyline** 3: **Pregabalin 150mg** 4: **Pregabalin 300mg** 5: **Pregabalin 450mg** 6: **Pregabalin 600mg** 7: **Duloxetine 60mg** 8: **Duloxetine 120mg** 9: **Milnacipran 100mg** 10: **Milnacipran 200mg.**

eTable 4E1. Inconsistency Test Based on Loop-Specific Approach (continued)

© 2022 Farag HM et al. *JAMA Network Open.*

Loop	IF	seIF	z_value	p_value	CI_95	Loop_Heterog_tau2
01-07-08	0.251	0.164	1.527	0.127	(0.00,0.57)	0.006
01-09-10	0.031	0.116	0.265	0.791	(0.00,0.26)	0.007
01-04-06	0.007	0.079	0.090	0.928	(0.00,0.16)	0.000
01-04-05	0.007	0.076	0.093	0.926	(0.00,0.16)	0.000
01-03-04	0.007	0.057	0.118	0.906	(0.00,0.12)	0.000
04-05-06	.	.	.	.		0.000
01-05-06	.	.	.	.		0.000

1: **Placebo** 2: **Amitriptyline** 3: **Pregabalin 150mg** 4: **Pregabalin 300mg** 5: **Pregabalin 450mg** 6: **Pregabalin 600mg** 7: **Duloxetine 60mg** 8: **Duloxetine 120mg** 9: **Milnacipran 100mg** 10: **Milnacipran 200mg.**

eTable 4E1. Inconsistency Test Based on Loop-Specific Approach with tau= 0.02 (continued)

© 2022 Farag HM et al. *JAMA Network Open.*

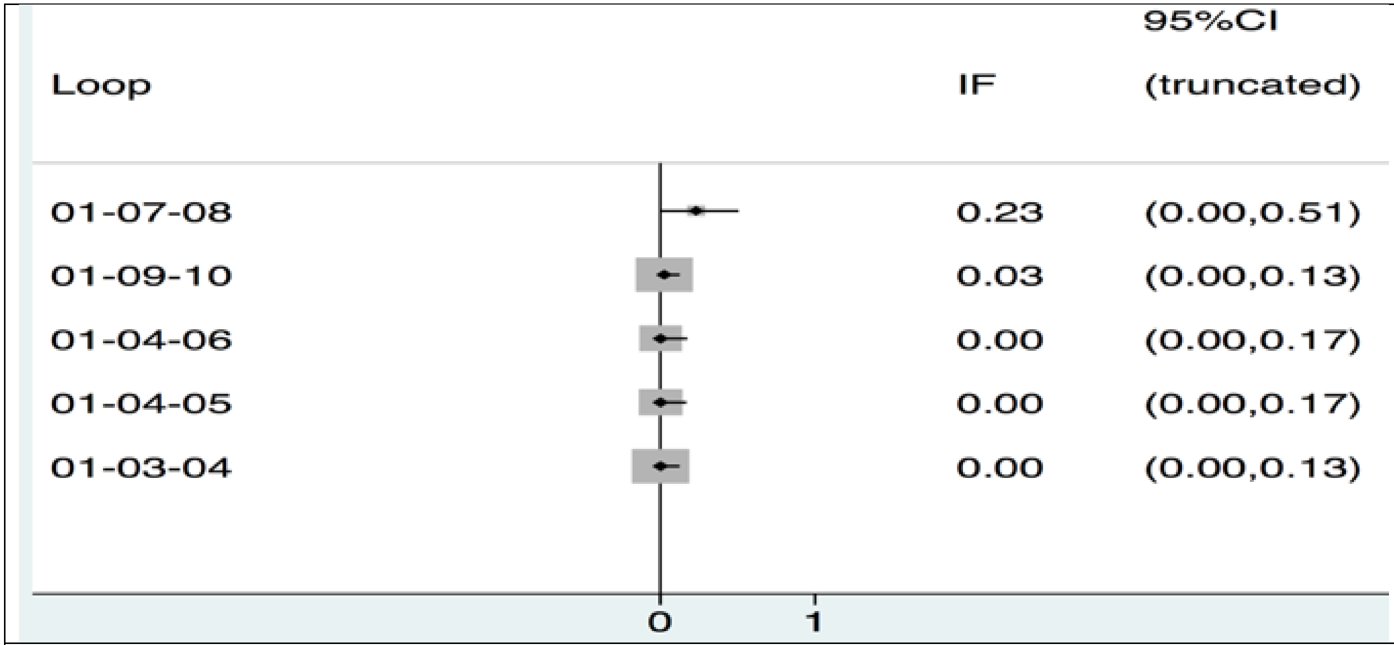

Inconsistency plot for the fibromyalgia treatments network showing for each loop the ratio of standardized mean differences between direct and indirect estimates. The within-loop heterogeneities have been estimated using the method of moments estimator. 1: **Placebo** 2: **Amitriptyline** 3: **Pregabalin 150mg** 4: **Pregabalin 300mg** 5: **Pregabalin 450mg** 6: **Pregabalin 600mg** 7: **Duloxetine 60mg** 8: **Duloxetine 120mg** 9: **Milnacipran 100mg** 10: **Milnacipran 200mg.**

eTable 4E1. Inconsistency Test Based on Loop-Specific Approach with tau= 0.02 (continued)

Loop	IF	seIF	z_value	p_value	CI_95
01-07-08	0.232	0.141	1.644	0.100	(0.00,0.51)
01-09-10	0.025	0.052	0.489	0.625	(0.00,0.13)
01-04-06	0.002	0.088	0.026	0.979	(0.00,0.17)
01-04-05	0.002	0.085	0.026	0.979	(0.00,0.17)
01-03-04	0.002	0.063	0.033	0.973	(0.00,0.13)
04-05-06	.	.	.	.	
01-05-06	.	.	.	.	

1: **Placebo** 2: **Amitriptyline** 3: **Pregabalin 150mg** 4: **Pregabalin 300mg** 5: **Pregabalin 450mg** 6: **Pregabalin 600mg** 7: **Duloxetine 60mg** 8: **Duloxetine 120mg** 9: **Milnacipran 100mg** 10: **Milnacipran 200mg.**

© 2022 Farag HM et al. *JAMA Network Open.*

eTable 4E2. Inconsistency Test Based on Side-Splitting Approach

Side	Direct Coef.	Std. Err.	Indirect Coef.	Std. Err.	Difference Coef.	Std. Err.	P>|z|
01 02	.	.	.	.	.	.	.
01 03 *	-.1232347	.0591225	-.1354339	.1691428	.0121992	.178998	0.946
01 04 *	-.1440536	.0440092	-.1683211	.2339125	.0242675	.2379857	0.919
01 05 *	-.1777092	.0639168	-.1725463	.1627494	-.005163	.1727927	0.976
01 06 *	-.1115263	.0657172	-.1063636	.1634613	-.0051627	.1727974	0.976
01 07	-.2092237	.067473	-.3410644	.256885	.1318407	.2655514	0.620
01 08	-.3269505	.0912691	-.6286991	.1695262	.3017486	.1922544	0.117
01 09	.0488127	.0416565	.2055629	.0982837	-.1567502	.1067089	0.142
01 10	.0661086	.0455029	.1268703	.1073004	-.0607617	.116285	0.601
03 04	-.0205753	.0733499	-.0198922	.1058447	-.0006831	.1287226	0.996
04 05 *	-.0315435	.0639317	-.0367062	.1627326	.0051628	.1727941	0.976
04 06 *	.0346395	.0657324	.0294766	.1634563	.0051628	.1727945	0.976
05 06	.	.	.	.	.	.	.
07 08	-.2307919	.0855617	.0865942	.1870423	-.3173861	.2049326	0.121
09 10	-.0058112	.041502	.0631742	.1184096	-.0689853	.1254613	0.582

1: **Placebo** 2: **Amitriptyline** 3: **Pregabalin 150mg** 4: **Pregabalin 300mg** 5: **Pregabalin 450mg** 6: **Pregabalin 600mg** 7: **Duloxetine 60mg** 8: **Duloxetine 120mg** 9: **Milnacipran 100mg** 10: **Milnacipran 200mg.**

eTables 4F. Assessment of inconsistency for the acceptability outcome

© 2022 Farag HM et al. *JAMA Network Open.*

eTable 4F1. Inconsistency Test Based on Loop-Specific Approach

Loop	ROR	95%CI (truncated)	Loop-specific Heterogeneity(τ^2)
01 09 10	1.748	(1.02,3.00)	0.000
01 05 06	1.620	(1.00,3.81)	0.000
01 07 08	1.355	(1.00,2.40)	0.000
*01 04 06	1.081	(1.00,2.06)	0.000
*04 05 06	1.045	(1.00,1.89)	0.000
01 04 05	1.008	(1.00,3.13)	0.000

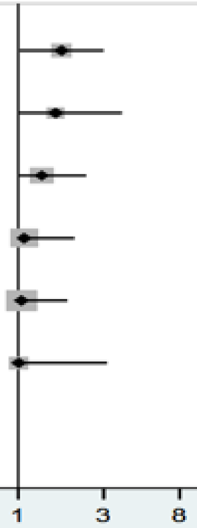

Note: * These loops are formed only by multi-arm trial(s)

For the dichotomous outcomes, inconsistency factors (IF) are the ratios of odds ratios (RORs). CI_95 = 95% confidence interval. Loop_Heterog_tau2 = loop-specific heterogeneity. All loops were consistent (p > 0.1) indicating lack of evidence of inconsistency in the network, except the milnacipran 100mg and 200mg loop. 1: **Placebo** 2: **Amitriptyline** 3: **Pregabalin 150mg** 4: **Pregabalin 300mg** 5: **Pregabalin 450mg** 6: **Pregabalin 600mg** 7: **Duloxetine 60mg** 8: **Duloxetine 120mg** 9: **Milnacipran 100mg** 10: **Milnacipran 200mg.**

eTable 4F1. Inconsistency Test Based on Loop-Specific Approach (continued)

© 2022 Farag HM et al. *JAMA Network Open.*

01 09 10	1.748	2.026	0.043	(1.02,3.00)	0.000
01 05 06	1.620	1.106	0.269	(1.00,3.81)	0.000
01 07 08	1.355	1.039	0.299	(1.00,2.40)	0.000
*01 04 06	1.081	0.235	0.814	(1.00,2.06)	0.000
*04 05 06	1.045	0.146	0.884	(1.00,1.89)	0.000
01 04 05	1.008	0.013	0.989	(1.00,3.13)	0.000

1: **Placebo** 2: **Amitriptyline** 3: **Pregabalin 150mg** 4: **Pregabalin 300mg** 5: **Pregabalin 450mg** 6: **Pregabalin 600mg** 7: **Duloxetine 60mg** 8: **Duloxetine 120mg** 9: **Milnacipran 100mg** 10: **Milnacipran 200mg.**

eTable 4F2. Inconsistency Test Based on Side-Splitting Approach

© 2022 Farag HM et al. *JAMA Network Open.*

Side	Direct Coef.	Std. Err.	Indirect Coef.	Std. Err.	Difference Coef.	Std. Err.	P>|z|
01 02	.	.	.	.	.	.	.
01 03	.	.	.	.	.	.	.
01 04	.	.	.	.	.	.	.
01 05	.	.	.	.	.	.	.
01 06	.	.	.	.	.	.	.
01 07	.	.	.	.	.	.	.
01 08	.	.	.	.	.	.	.
01 09	.4979884	.1329159	1.009211	.3275944	-.5112223	.3335779	0.125
01 10	.9572248	.1355619	.4459829	.3265118	.5112419	.3335809	0.125
04 05 *	.1560925	.1859674	.1606497	.5700644	-.0045571	.5691973	0.994
04 06	.	.	.	.	.	.	.
05 06	.	.	.	.	.	.	.
07 08	.3791826	.1484603	-.0090316	.4490545	.3882143	.4546414	0.393
09 10	.2666263	.1839341	.4555541	.2951959	-.1889278	.3098821	0.542

1: **Placebo** 2: **Amitriptyline** 3: **Pregabalin 150mg** 4: **Pregabalin 300mg** 5: **Pregabalin 450mg** 6: **Pregabalin 600mg** 7: **Duloxetine 60mg** 8: **Duloxetine 120mg** 9: **Milnacipran 100mg** 10: **Milnacipran 200mg.**

Illustrated legends were adapted from "Graphical Tools for Network Meta-Analysis in STATA", by Chaimani et al.[53]

eTable 5. Pain Outcome: League Table

© 2022 Farag HM et al. *JAMA Network Open.*

Treatment	DLX120	DLX60	MLN100	MLN200	PLB	PRG150	PRG300	PRG450	PRG600
AMT	-0.06 (0.00, 0.12)	0.03 (-0.03, 0.09)	0.09 (0.03, 0.15)	0.04 (-0.02, 0.10)	0.27 (0.21, 0.32)	0.04 (-0.03, 0.11)	0.03 (-0.02, 0.09)	-0.03 (-0.09, 0.03)	-0.01 (-0.07, 0.05)
DLX120		0.09 (0.06, 0.13)	0.15 (0.11, 0.20)	0.11 (0.06, 0.15)	0.33 (0.30, 0.36)	0.10 (0.04, 0.17)	0.10 (0.06, 0.14)	0.03 (-0.01, 0.08)	0.05 (0.01, 0.10)
DLX60			0.06 (0.03, 0.09)	0.01 (-0.02, 0.05)	0.24 (0.21, 0.26)	0.01 (-0.05, 0.07)	0.00 (-0.03, 0.04)	-0.06 (-0.09, -0.03)	-0.04 (-0.07, -0.00)
MLN100				-0.05 (-0.08, -0.02)	0.17 (0.015, 0.20)	-0.05 (-0.11, 0.01)	-0.06 (-0.09, -0.02)	-0.12 (-0.16, -0.09)	-0.10 (-0.14, -0.06)
MLN200					0.22 (0.20, 0.25)	0.00 (-0.06, 0.06)	-0.01 (-0.04, 0.03)	-0.07 (-0.11, -0.03)	-0.05 (-0.09, -0.01)
PLB						-0.23 (-0.28, -0.17)	-0.23 (-0.26, -0.21)	-0.30 (-0.32, -0.27)	-0.27 (-0.30, -0.25)
PRG150							0.00 (-0.06, 0.05)	-0.07 (-0.13, -0.01)	-0.05 (-0.11, 0.01)
PRG300								-0.06 (-0.09, -0.04)	-0.04 (-0.07, -0.02)
PRG450									0.02 (-0.01, 0.05)

Comparisons between treatments should be read from left to right, and the estimates are in the cell in common between the column-defining treatment and the row-defining treatment. Data are standardized mean differences (SMDs) and credible intervals (95% CrI) in the column-defining treatment compared with the row-defining treatment. SMDs lower than 0 favor the column-defining treatment. To obtain SMDs for comparisons in the opposite direction, reciprocals should be taken.

eTable 6. Sleep Outcome: League Table

Treatment	DLX120	DLX60	MLN100	MLN200	PLB	PRG150	PRG300	PRG450	PRG600
AMT	0.66	0.76	0.74	0.66	0.97	0.40	0.67	0.47	0.36

© 2022 Farag HM et al. *JAMA Network Open.*

	(0.48, 0.84)	(0.60, 0.92)	(0.52, 0.95)	(-0.60, 1.91)	(0.83, 1.10)	(0.22, 0.57)	(0.52, 0.81)	(0.32, 0.81)	(0.21, 0.51)
**DLX120**		0.09 (-0.03,0.22)	0.08 (-0.13, 0.28)	0.00 (-1.26, 1.25)	0.31 (0.18, 0.43)	-0.27 (-0.43, -0.10)	0.00 (-0.13, 0.14)	-0.19 (-0.33, -0.05)	-0.30 (-0.44, -0.16)
**DLX60**			-0.02 (-0.21, 0.17)	-0.10 (-1.35, 1.15)	0.21 (0.13, 0.30)	-0.36 (-0.50, -0.22)	-0.09 (-0.20, 0.02)	-0.29 (-0.40, -0.18)	-0.39 (-0.50, -0.28)
**MLN100**				-0.08 (-1.32, 1.16)	0.23 (0.06, 0.40)	-0.34 (-0.54, -0.14)	-0.07 (-0.25, 0.11)	-0.27 (-0.45, -0.09)	-0.37 (-0.55, -0.28)
**MLN200**					0.31 (-0.94, 1.56)	-0.26 (-1.52, 0.99)	0.01 (-1.24, 1.26)	-0.19 (-1.44, 1.06)	-0.29 (-1.54, 0.96)
**PLB**						-0.57 (-0.68, -0.46)	-0.30 (-0.37, -0.24)	-0.50 (-0.56, -0.43)	-0.60 (-0.67, -0.54)
**PRG150**							0.27 (0.14, 0.39)	0.07 (-0.05, 0.20)	-0.03 (-0.16, 0.09)
**PRG300**								-0.20 (-0.26, -0.13)	-0.30 (-0.37, -0.24)
**PRG450**									-0.11 (-0.17, -0.04)

Comparisons between treatments should be read from left to right, and the estimates are in the cell in common between the column-defining treatment and the row-defining treatment. Data are standardized mean differences (SMDs) and credible intervals (95% CrI) in the column-defining treatment compared with the row-defining treatment. SMDs lower than 0 favor the column-defining treatment. To obtain SMDs for comparisons in the opposite direction, reciprocals should be taken.

eTable 7. Depression Outcome: League Table

Treatment	DLX120	DLX60	MLN100	MLN200	PLB	PRG150	PRG300	PRG450	PRG600
**AMT**	-0.25 (-0.37, -0.12)	-0.24 (-0.34, -0.12)	-0.10 (-0.20, 0.00)	-0.07 (-0.17, 0.03)	0.00 (-0.10, 0.10)	-0.05 (-0.15, 0.06)	-0.23 (-0.33, -0.12)	-0.14 (-0.25, -0.03)	-0.23 (-0.34, -0.12)

© 2022 Farag HM et al. *JAMA Network Open.*

**DLX120**		0.01 (-0.07, 0.08)	0.15 (0.07, 0.23)	0.18 (0.10, 0.26)	0.25 (0.17, 0.32)	0.20 (0.12, 0.28)	0.02 (-0.06, 0.10)	0.11 (0.02, 0.20)	0.02 (-0.07, 0.11)
**DLX60**			0.14 (0.09, 0.18)	0.17 (0.12, 0.21)	0.24 (0.20, 0.27)	0.19 (0.15, 0.24)	0.01 (-0.04, 0.06)	0.10 (0.04, 0.16)	0.01 (-0.05, 0.88)
**MLN100**				0.03 (-0.01, 0.06)	0.10 (0.07, 0.12)	0.05 (0.02, 0.09)	-0.13 (-0.17, -0.08)	-0.04 (-0.09, 0.01)	-0.13 (-0.19, -0.07)
**MLN200**					0.07 (0.04, 0.10)	0.03 (-0.01,0.06)	-0.16 (-0.20, -0.11)	-0.07 (-0.12, -0.01)	-0.16 (-0.22, -0.10)
**PLB**						-0.04 (-0.07,-0.02)	-0.22 (-0.26, -0.19)	-0.14 (-0.18, -0.04)	-0.23 (-0.28, -0.17)
**PRG150**							-0.18 (-0.22, -0.14)	-0.09 (-0.15, -0.04)	-0.18 (-0.24, -0.12)
**PRG300**								0.09 (0.04, 0.14)	0.00 (-0.06, 0.05)
**PRG450**									-0.09 (-0.15, -0.03)

Comparisons between treatments should be read from left to right, and the estimates are in the cell in common between the column-defining treatment and the row-defining treatment. Data are standardized mean differences (SMDs) and credible intervals (95% CrI) in the column-defining treatment compared with the row-defining treatment. SMDs lower than 0 favor the column-defining treatment. To obtain SMDs for comparisons in the opposite direction, reciprocals should be taken.

eTable 8. Fatigue Outcome: League Table

Treatment	DLX120	DLX60	MLN100	MLN200	PLB	PRG150	PRG300	PRG450	PRG600
**AMT**	0.52 (0.41, 0.64)	0.51 (0.40, 0.62)	0.54 (0.43, 0.66)	0.47 (0.36, 0.59)	0.64 (0.53, 0.75)	0.38 (0.26, 0.49)	0.47 (0.33, 0.60)	0.48 (0.32, 0.63)	0.39 (0.24, 0.55)

© 2022 Farag HM et al. *JAMA Network Open.*

**DLX120**		-0.01 (-0.05, 0.03)	0.02 (-0.03, 0.08)	-0.05 (-0.11, 0.01)	0.12 (0.08, 0.16)	-0.15 (-0.19, -0.10)	-0.06 (-0.15, 0.04)	-0.04 (-0.16, 0.88)	-0.13 (-0.25, -0.01)
**DLX60**			0.03 (-0.02, 0.08)	-0.04 (-0.09, 0.01)	0.13 (0.11, 0.15)	-0.14 (-0.18, -0.10)	-0.05 (-0.14, 0.04)	-0.03 (-0.15, 0.08)	-0.12 (-0.23, -0.01)
**MLN100**				-0.07 (-0.12, -0.02)	0.10 (0.05, 0.14)	-0.17 (-0.22, -0.12)	-0.08 (-0.17, 0.02)	-0.07 (-0.19, 0.05)	-0.15 (-0.27, -0.03)
**MLN200**					0.17 (0.13, 0.21)	-0.10 (-0.15, -0.04)	-0.01 (-0.10, 0.09)	0.01 (-0.11, 0.13)	-0.08 (-0.20, 0.04)
**PLB**						-0.27 (-0.29, -0.24)	-0.17 (-0.26, -0.09)	-0.16 (-0.27, -0.05)	-0.25 (-0.36, -0.16)
**PRG150**							0.09 (0.00, 0.18)	0.10 (-0.01, 0.22)	0.02 (-0.10, 0.13)
**PRG300**								0.01 (-0.10, 0.13)	-0.07 (-0.18, 0.04)
**PRG450**									-0.09 (-0.21, 0.04)

Comparisons between treatments should be read from left to right, and the estimates are in the cell in common between the column-defining treatment and the row-defining treatment. Data are standardized mean differences (SMDs) and credible intervals (95% CrI) in the column-defining treatment compared with the row-defining treatment. SMDs lower than 0 favor the column-defining treatment. To obtain SMDs for comparisons in the opposite direction, reciprocals should be taken.

eTable 9. Quality of Life Outcome: League Table

Treatment	DLX120	DLX60	MLN100	MLN200	PLB	PRG150	PRG300	PRG450	PRG600
**AMT**	0.40 (0.19, 0.62)	0.58 (0.39, 0.77)	0.87 (0.71,1.03)	0.87 (0.71, 1.04)	0.80 (0.65, 0.94)	0.67 (0.50, 0.85)	0.65 (0.49, 0.82)	0.62 (0.44, 0.80)	0.69 (0.50, 0.87)
**DLX120**		0.18 (0.02, 0.33)	0.47 (0.29, 0.65)	0.47 (0.29, 0.65)	0.39 (0.23, 0.55)	0.27 (0.08, 0.46)	0.25 (0.07, 0.43)	0.22 (0.02, 0.41)	0.28 (0.08, 0.48)
**DLX60**			0.29 (0.14, 0.44)	0.29 (0.14, 0.44)	0.22 (0.09, 0.35)	0.09 (-0.07,0.26)	0.07 (-0.08, 0.22)	0.04 (-0.13, 0.21)	0.11 (-0.07, 0.28)
**MLN100**				0.00	-0.07	-0.20 (-0.33, -0.07)	-0.22 (-0.33, -0.11)	-0.25 (-0.39, -0.11)	-0.18 (-0.33, -0.04)

© 2022 Farag HM et al. *JAMA Network Open.*

				(-0.07, 0.07)	(-0.15, 0.01)				
**MLN200**					-0.07<br>(-0.15, 0.01)	-0.20<br>(-0.33, -0.07)	-0.22<br>(-0.33, -0.11)	-0.25<br>(-0.39, -0.11)	-0.19<br>(-0.33, -0.04)
**PLB**						-0.12<br>(-0.23, -0.02)	-0.14<br>(-0.23, 0.09)	-0.18<br>(-0.29, -0.06)	-0.11<br>(-0.23, 0.01)
**PRG150**							-0.02<br>(-0.13, 0.09)	-0.05<br>(-0.20, 0.09)	0.01<br>(-0.14, 0.16)
**PRG300**								-0.03<br>(-0.15, 0.08)	0.03<br>(-0.08, 0.15)
**PRG450**									0.07<br>(-0.06, 0.20)

Comparisons between treatments should be read from left to right, and the estimates are in the cell in common between the column-defining treatment and the row-defining treatment. Data are standardized mean differences (SMDs) and credible intervals (95% CrI) in the column-defining treatment compared with the row-defining treatment. SMDs lower than 0 favor the column-defining treatment. To obtain SMDs for comparisons in the opposite direction, reciprocals should be taken.

eTable 10. Acceptability Outcome: League Table

Treatment	DLX120	DLX60	MLN100	MLN200	PLB	PRG150	PRG300	PRG450	PRG600
**AMT**	3.83<br>(1.42, 8.36)	2.67<br>(1.002, 5.82)	2.94<br>(1.05, 6.53)	3.96<br>(1.44, 8.73)	0.78<br>(0.31, 1.66)	6.35<br>(1.41, 19.1)	2.49<br>(0.87, 5.62)	2.89<br>(1.02, 6.46)	3.38<br>(1.13, 7.72)
**DLX120**		1.45<br>(1.05, 1.98)	0.79<br>(0.49, 1.28)	1.06<br>(0.66, 1.66)	2.51<br>(1.78, 3.41)	0.79<br>(0.23, 1.95)	1.61<br>(0.92, 2.64)	1.39<br>(0.8, 2.25)	1.2<br>(0.65, 2.01)
**DLX60**			1.13<br>(0.71, 1.81)	1.52<br>(0.96, 2.35)	1.74<br>(1.28, 2.32)	0.55<br>(0.16, 1.34)	1.12<br>(0.65, 1.8)	0.96<br>(0.57, 1.53)	0.84<br>(0.46, 1.39)
**MLN100**				1.37<br>(0.93, 1.86)	1.91<br>(1.38, 2.76)	0.61<br>(0.17, 1.53)	1.24<br>(0.71, 2.11)	1.06<br>(0.62,1.79)	0.92<br>(0.52, 1.59)

© 2022 Farag HM et al. *JAMA Network Open.*

**MLN200**					2.58 (1.86, 3.67)	0.82 (0.23, 2.02)	1.67 (0.96, 2.78)	1.43 (0.84, 2.35)	1.24 (0.69, 2.1)
**PLB**						4.16 (1.34, 10.5)	1.62 (1.04, 2.39)	1.88 (1.24, 2.74)	2.2 (1.36, 3.37)
**PRG150**							0.51 (0.14, 1.29)	0.59 (0.16, 1.49)	0.69 (0.19, 1.78)
**PRG300**								1.19 (0.79, 1.71)	1.42 (0.73, 2.52)
**PRG450**									1.22 (0.64, 2.15)

Comparisons between treatments should be read from left to right, and the estimates are in the cell in common between the column-defining treatment and the row-defining treatment. Data are odds ratios ORs and credible intervals (95% CrI) in the column-defining treatment compared with the row-defining treatment. ORs lower than 1 favor the column-defining treatment. To obtain ORs for comparisons in the opposite direction, reciprocals should be taken.

eFigure 1. Network Plots

eFigure 1A. Pain outcome: Network plot

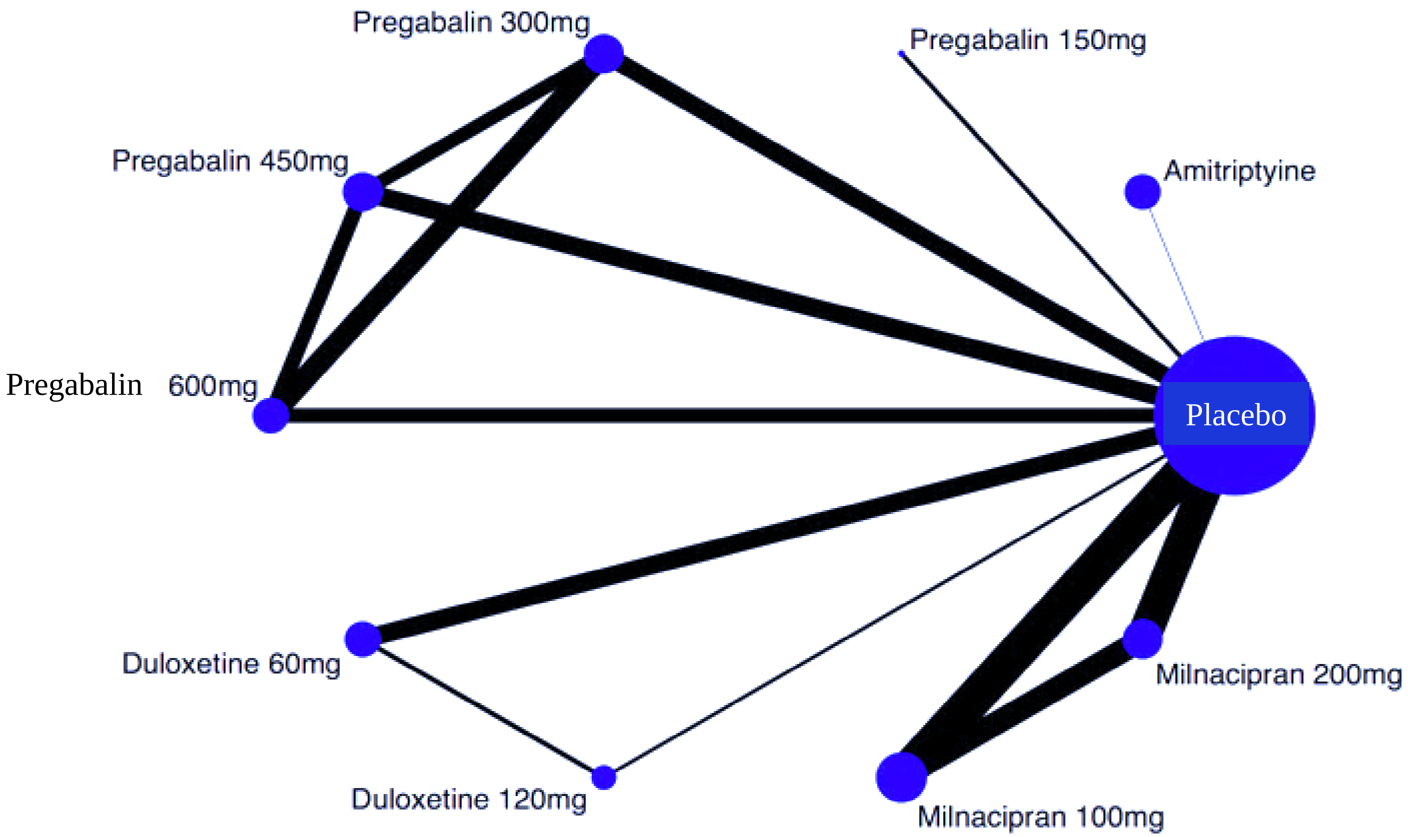

The width of the lines is proportional to the number of trials comparing every pair of treatments, and the size of every circle is proportional to the number of randomized participants (sample size).

eFigure 1A. Pain outcome: Network plot (continued)

© 2022 Farag HM et al. *JAMA Network Open.*

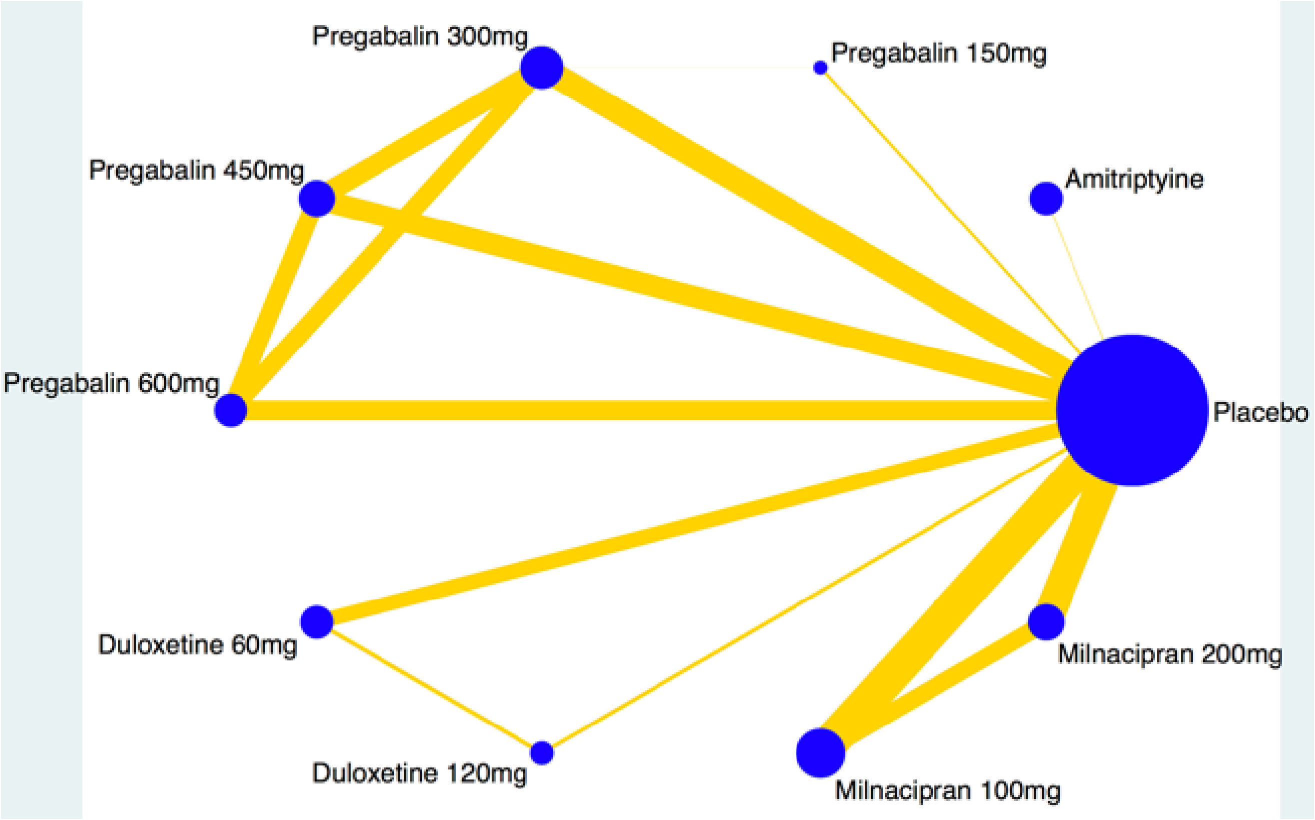

The width of the lines is proportional to the number of trials comparing every pair of treatment, and the size of every node is proportional to the number of randomized participants (sample size). The edges are colored according to adequacy of allocation concealment estimated as the level of bias in the majority of the trials and weighted according to the number of studies in each comparison.

© 2022 Farag HM et al. *JAMA Network Open.*

eFigure 1B. Sleep outcome: Network plot

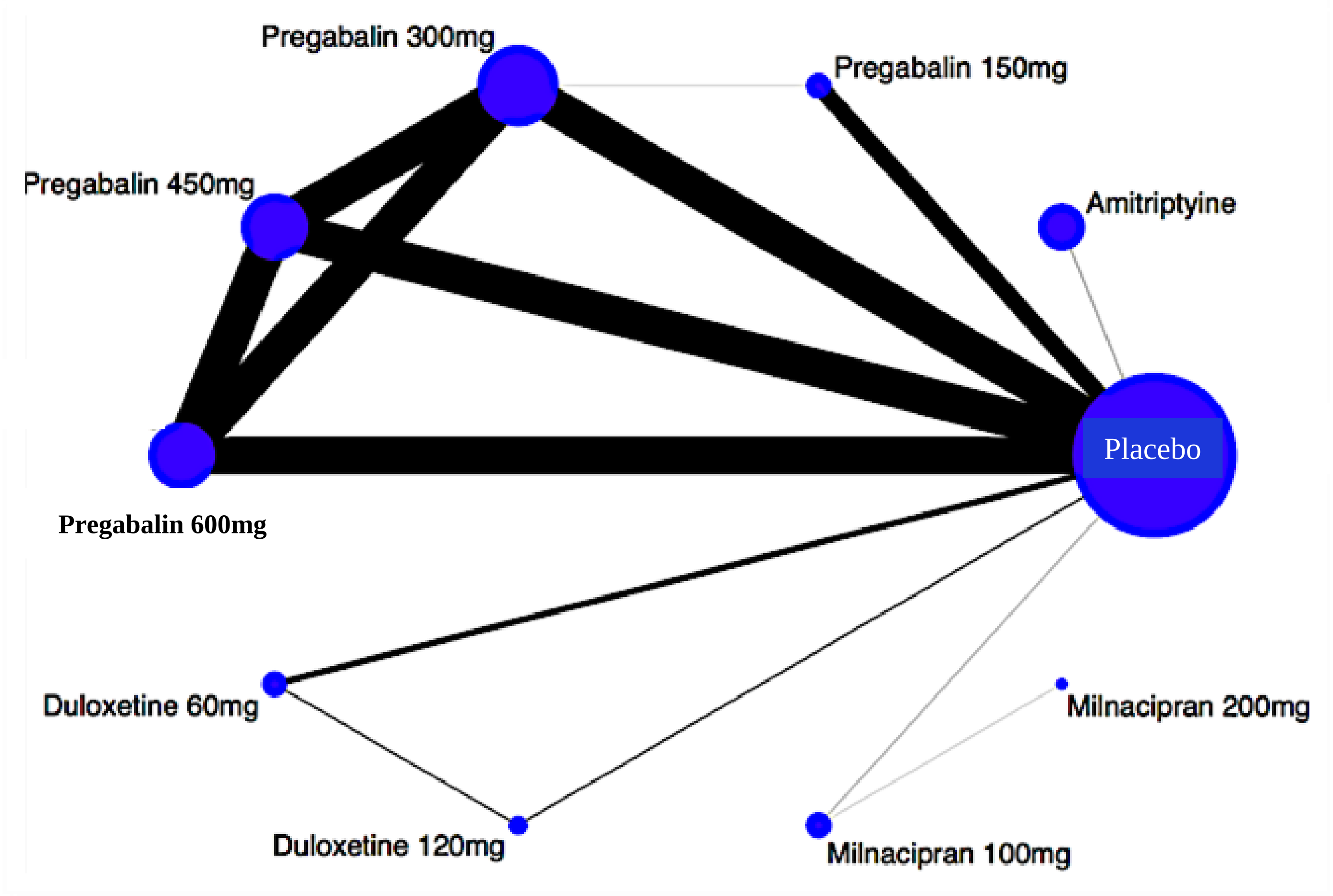

The width of the lines is proportional to the number of trials comparing every pair of treatments, and the size of every circle is proportional to the number of randomized participants (sample size).

eFigure 1B – Sleep outcome: Network plot (continued)

© 2022 Farag HM et al. *JAMA Network Open.*

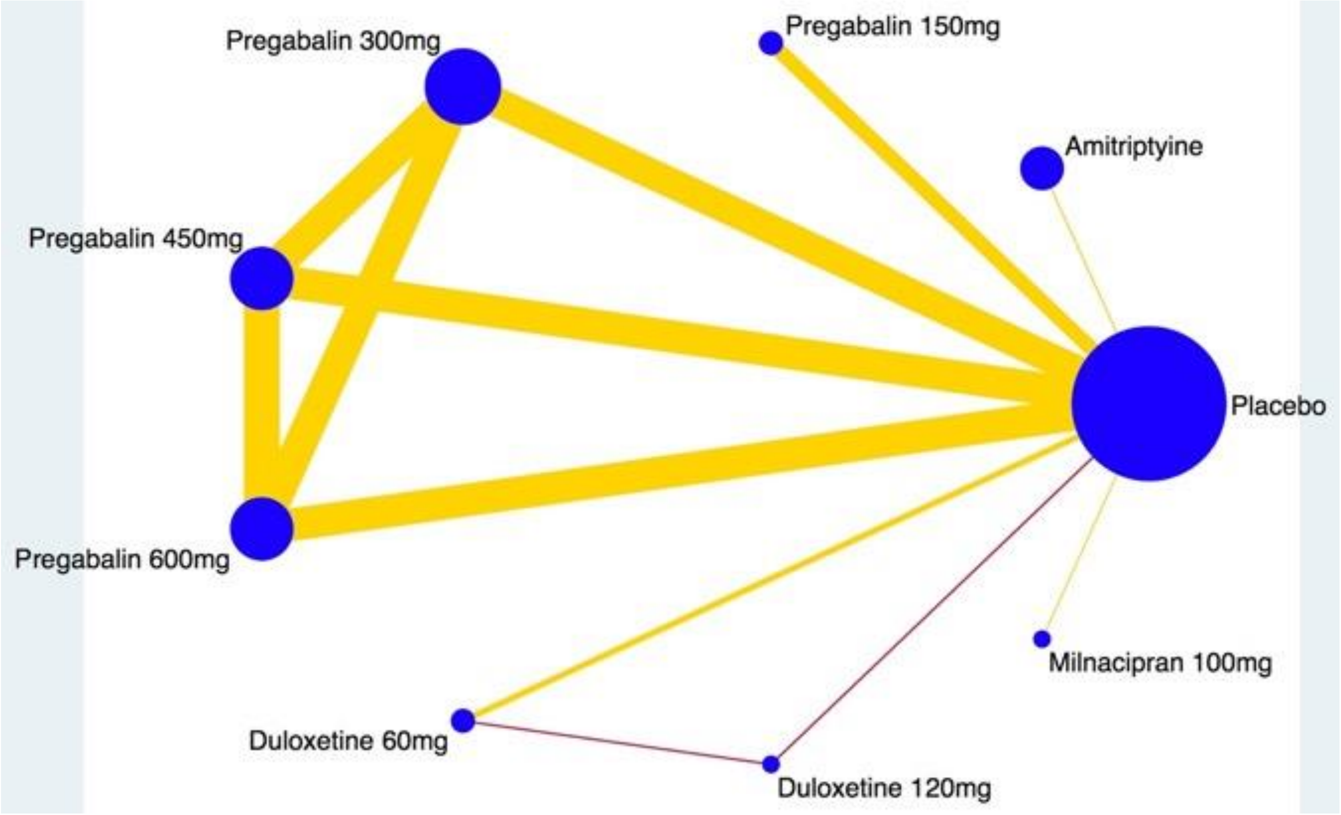

The width of the lines is proportional to the number of trials comparing every pair of treatment, and the size of every node is proportional to the number of randomized participants (sample size). The edges are colored according to adequacy of allocation concealment estimated as the level of bias in the majority of the trials and weighted according to the number of studies in each comparison.

© 2022 Farag HM et al. *JAMA Network Open.*

eFigure 1C. Depression outcome: Network plot

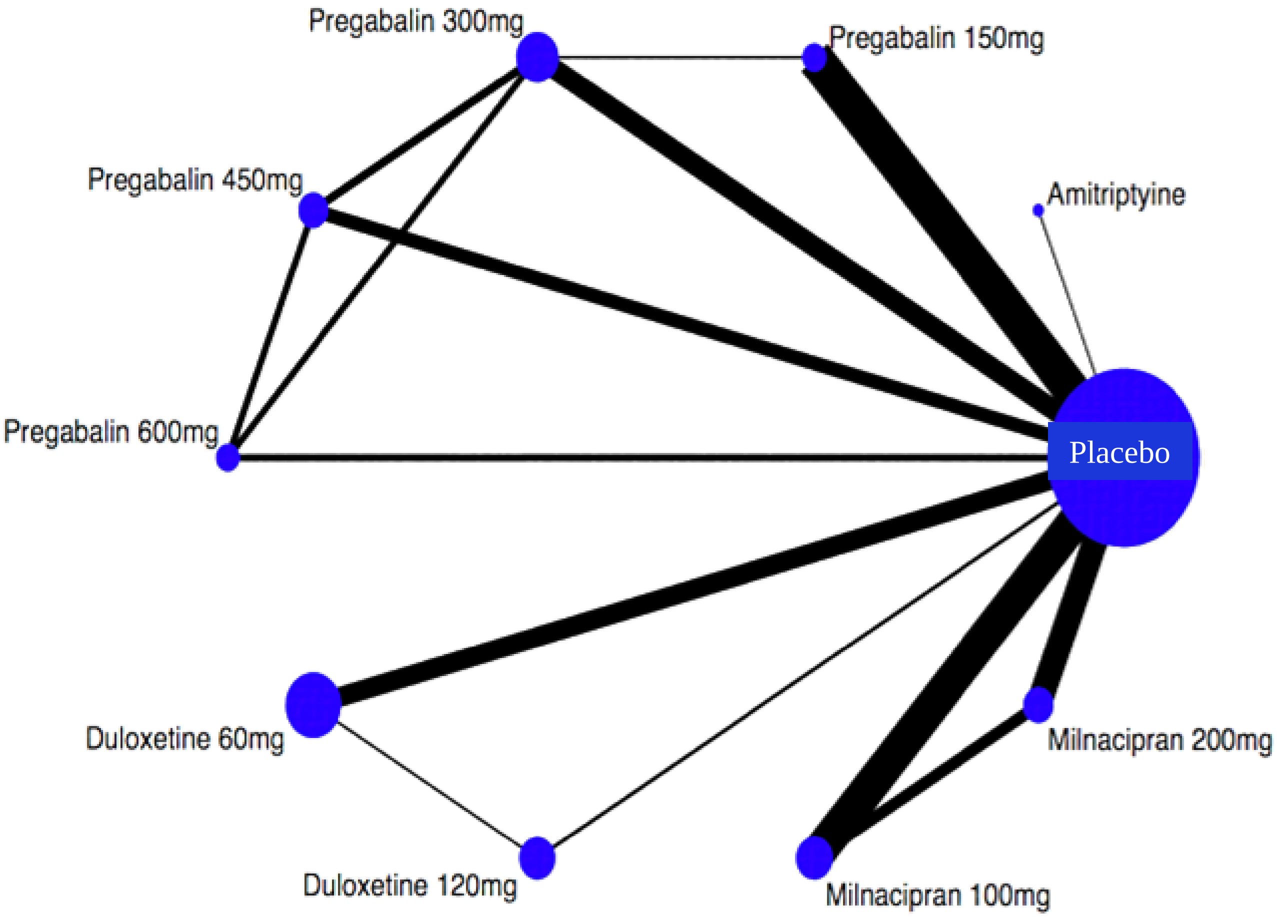

The width of the lines is proportional to the number of trials comparing every pair of treatments, and the size of every circle is proportional to the number of randomized participants (sample size).

eFigure 1C. Depression outcome: Network plot (continued)‹

© 2022 Farag HM et al. *JAMA Network Open.*

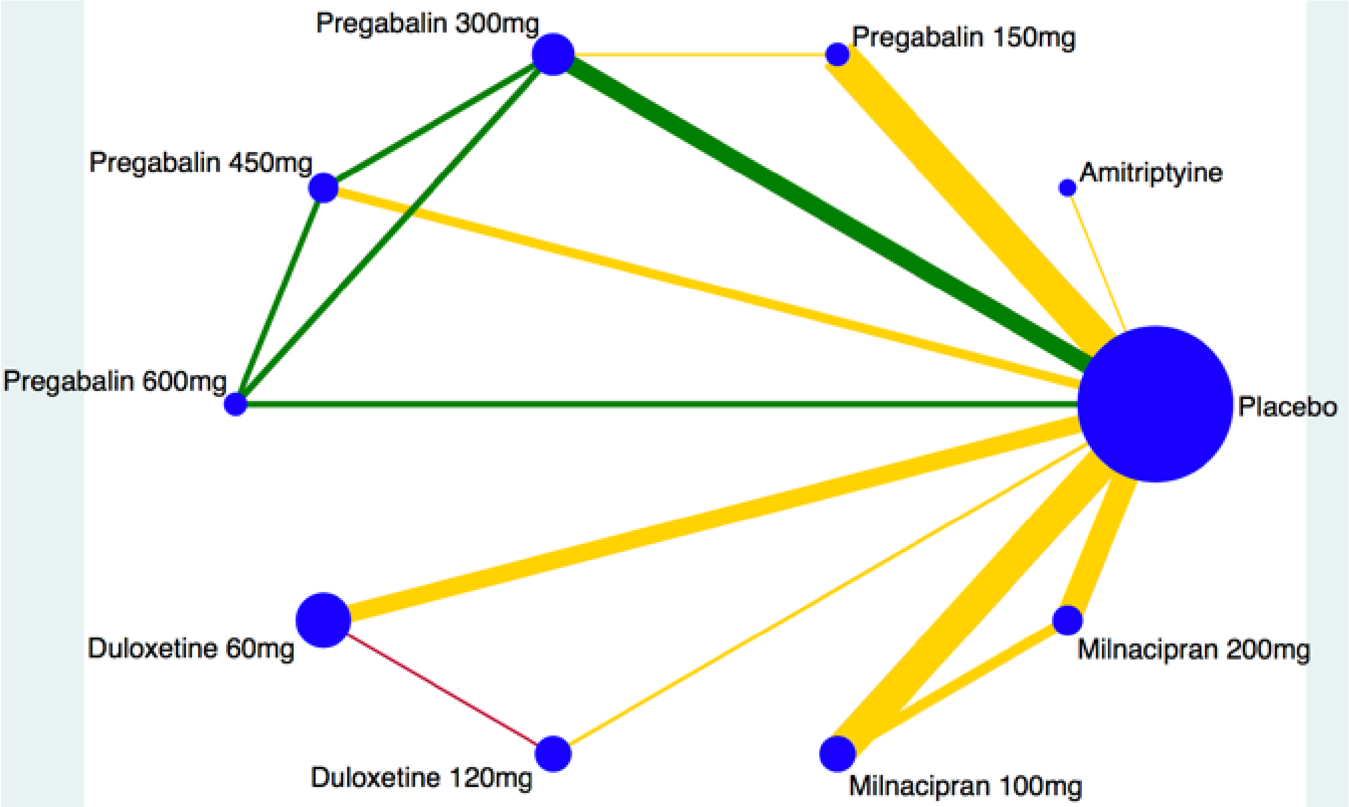

The width of the lines is proportional to the number of trials comparing every pair of treatment, and the size of every node is proportional to the number of randomized participants (sample size). The edges are colored according to adequacy of allocation concealment estimated as the level of bias in the majority of the trials and weighted according to the number of studies in each comparison.

© 2022 Farag HM et al. *JAMA Network Open.*

eFigure 1D. Fatigue outcome: Network plot

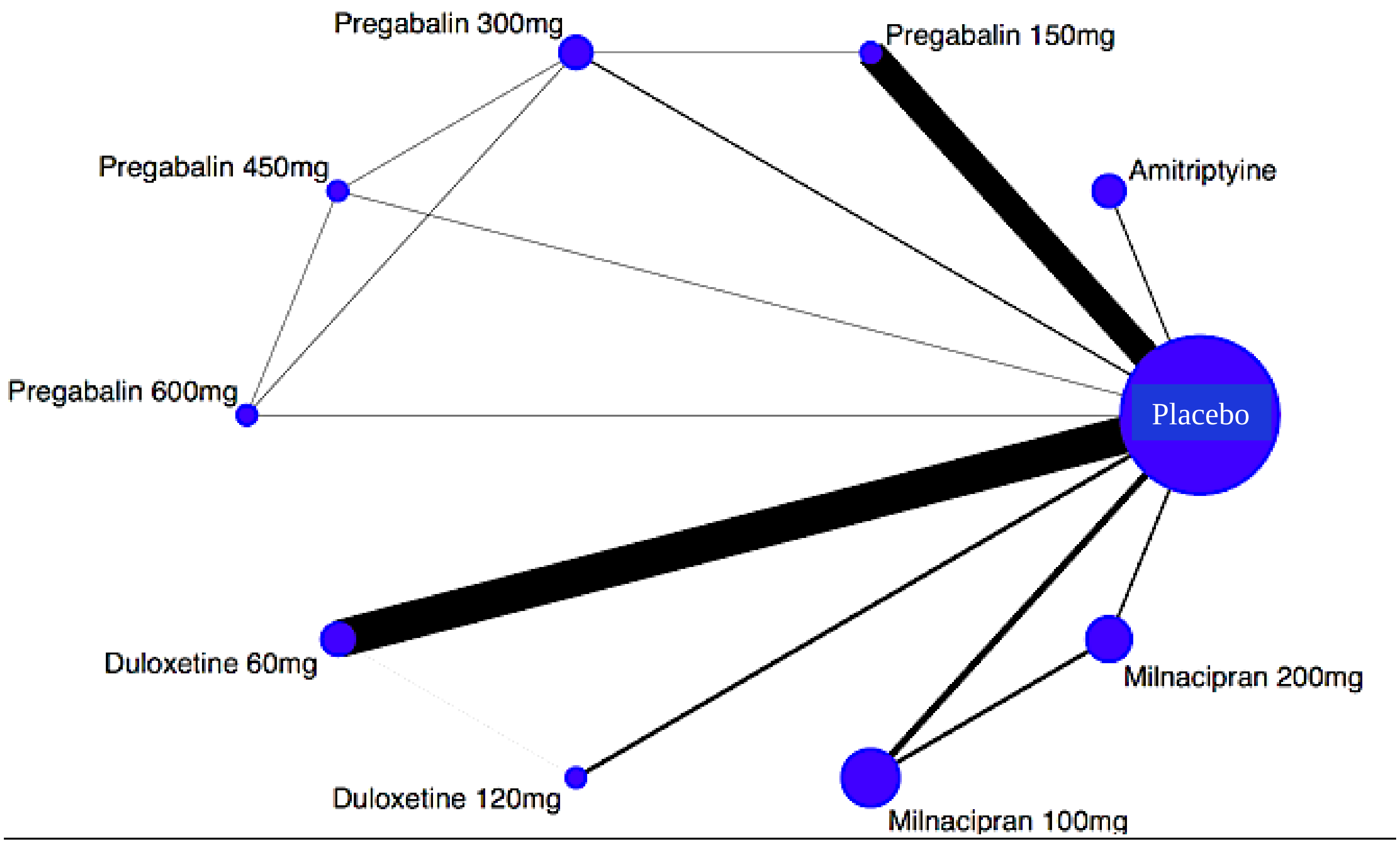

The width of the lines is proportional to the number of trials comparing every pair of treatments, and the size of every circle is proportional to the number of randomized participants (sample size).

eFigure 1D. Fatigue outcome: Network plot (continued)

© 2022 Farag HM et al. *JAMA Network Open.*

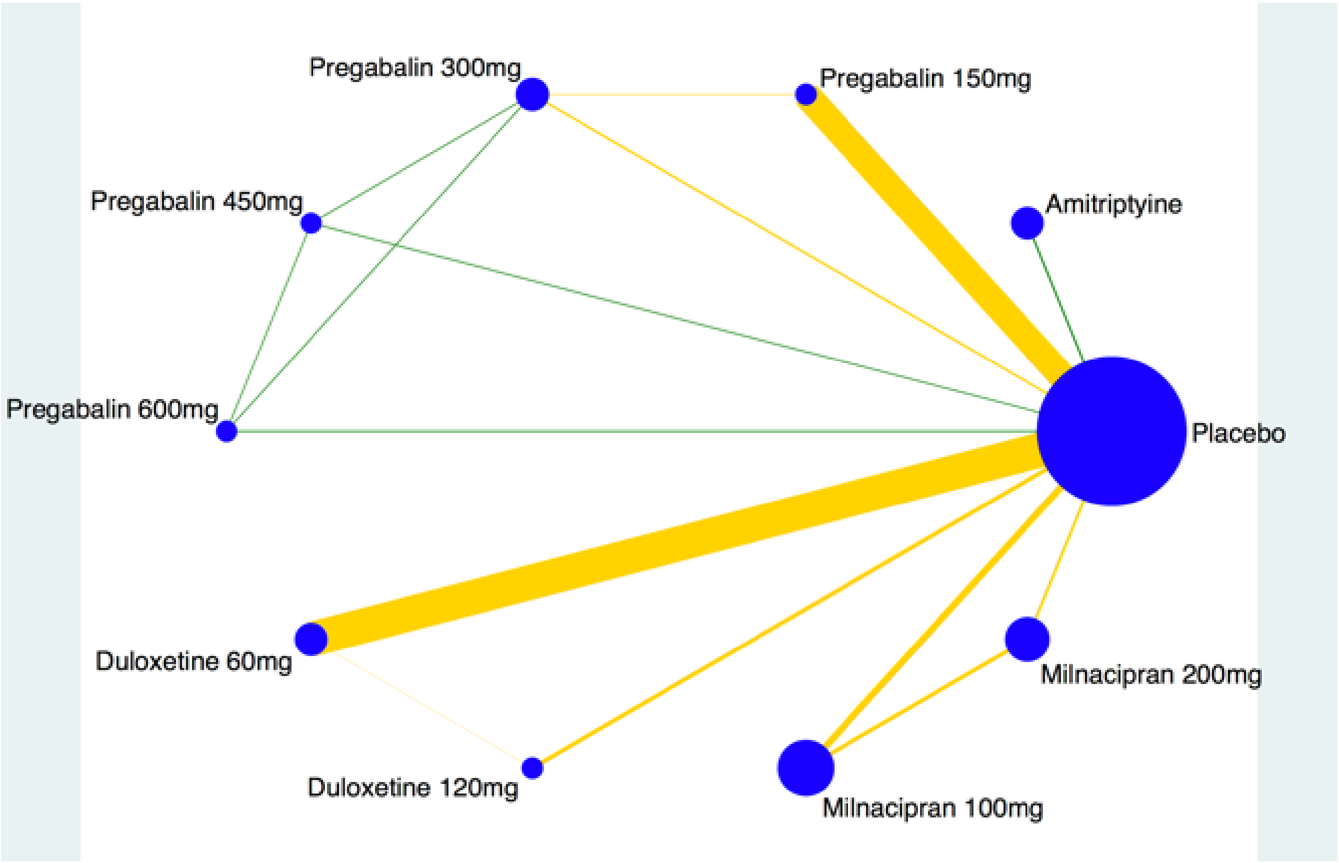

The width of the lines is proportional to the number of trials comparing every pair of treatment, and the size of every node is proportional to the number of randomized participants (sample size). The edges are coloured according to adequacy of allocation concealment estimated as the level of bias in the majority of the trials and weighted according to the number of studies in each comparison.

© 2022 Farag HM et al. *JAMA Network Open.*

eFigure 1E. Quality of life outcome: Network plot

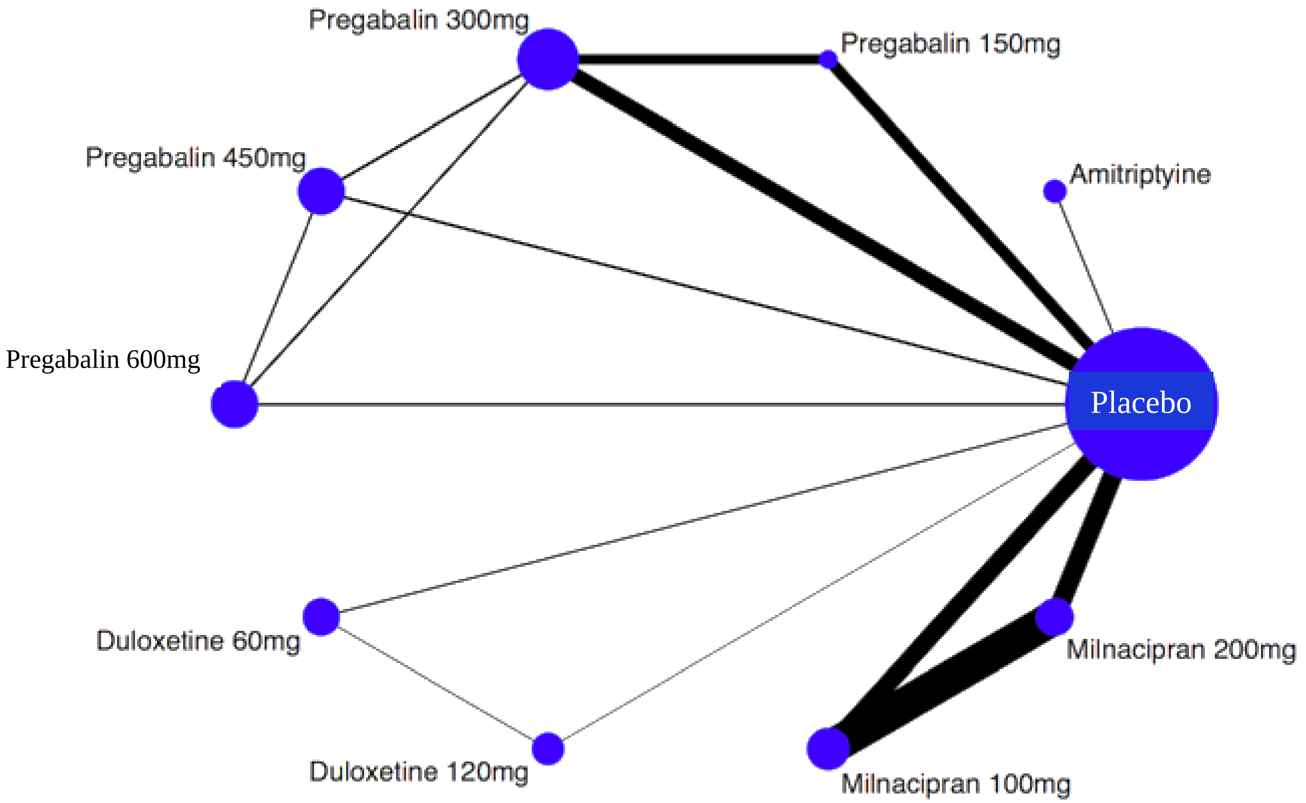

The width of the lines is proportional to the number of trials comparing every pair of treatments, and the size of every circle is proportional to the number of randomized participants (sample size).

eFigure 1E. Quality of life outcome: Network plot (continued)

© 2022 Farag HM et al. *JAMA Network Open.*

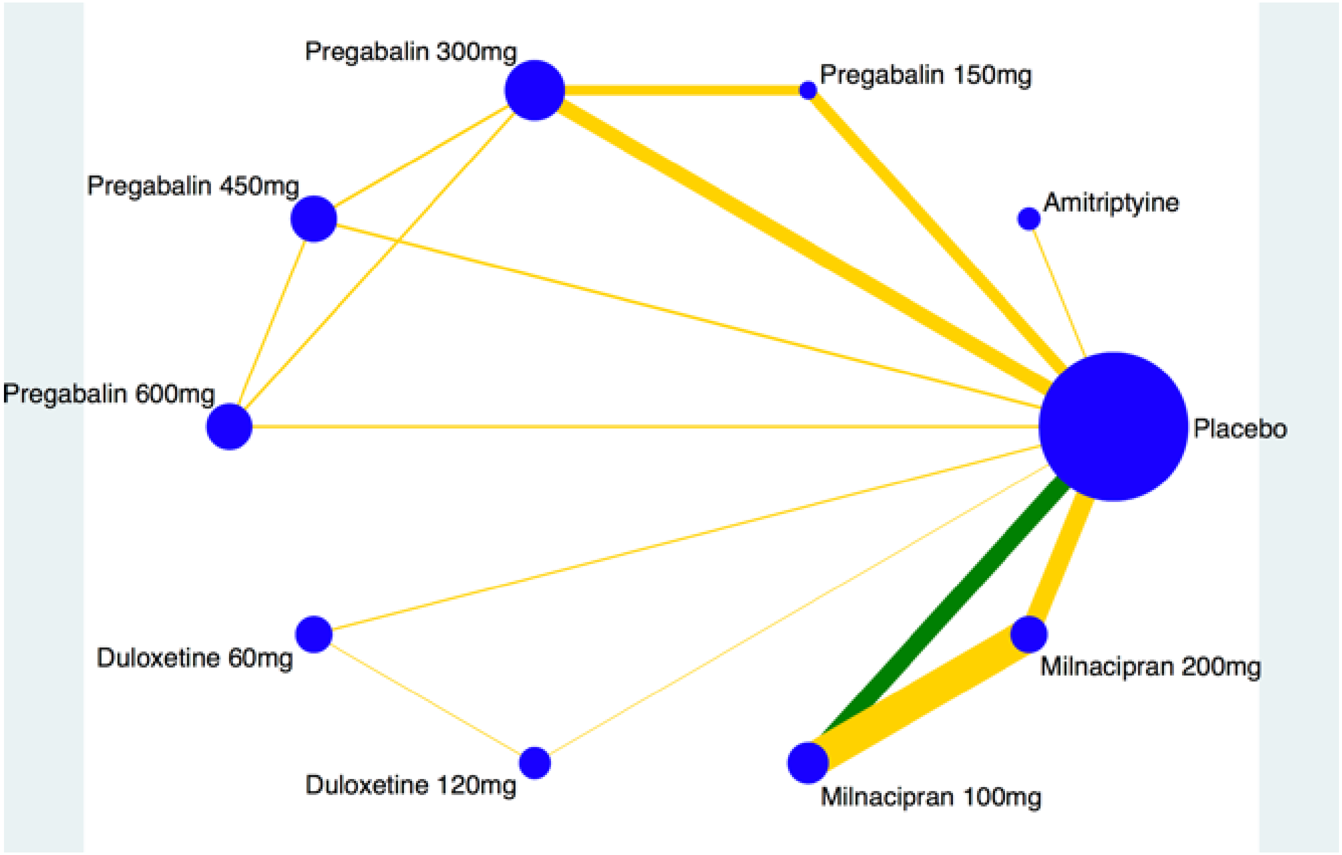

The width of the lines is proportional to the number of trials comparing every pair of treatment, and the size of every node is proportional to the number of randomized participants (sample size). The edges are coloured according to adequacy of allocation concealment estimated as the level of bias in the majority of the trials and weighted according to the number of studies in each comparison.

eFigure 1F. Acceptability outcome: Network plot

© 2022 Farag HM et al. *JAMA Network Open.*

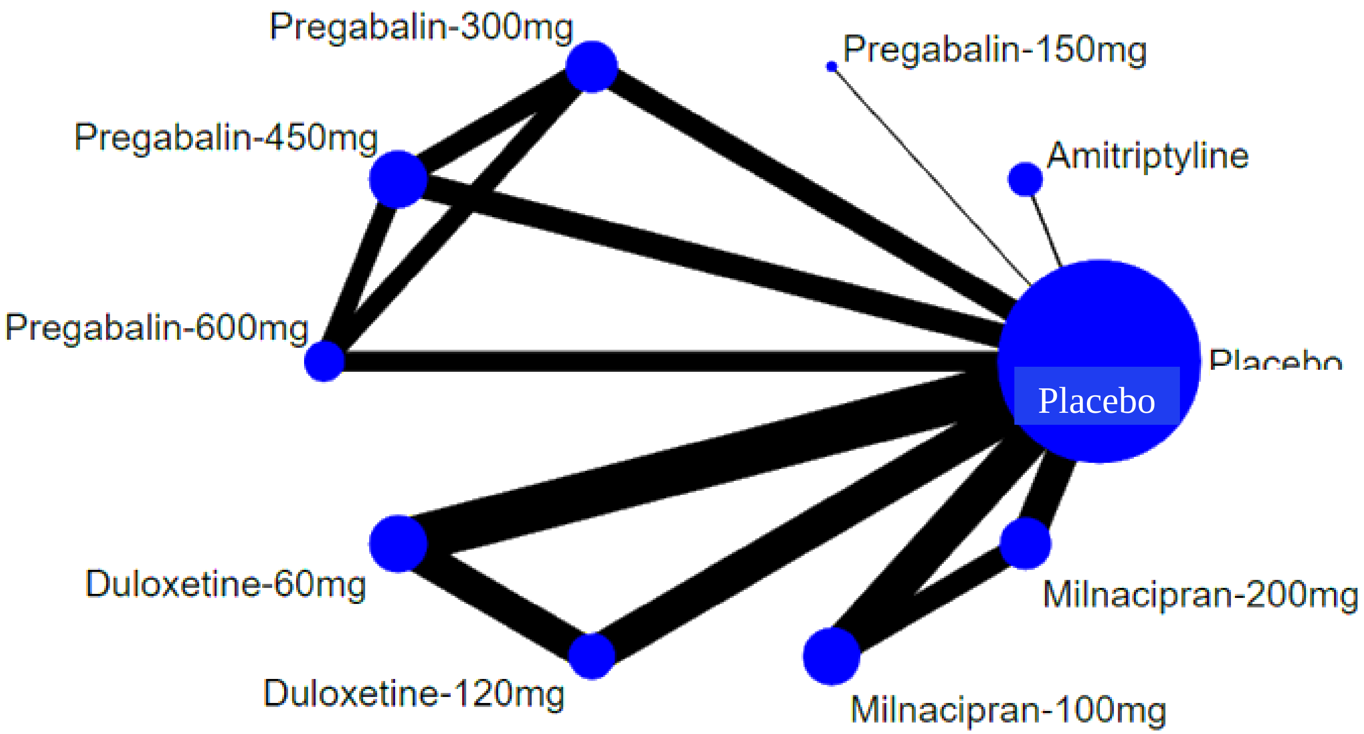

eFigure 2. Pain Outcome: Results

© 2022 Farag HM et al. *JAMA Network Open.*

eFigure 2A. Pain outcome: network forest

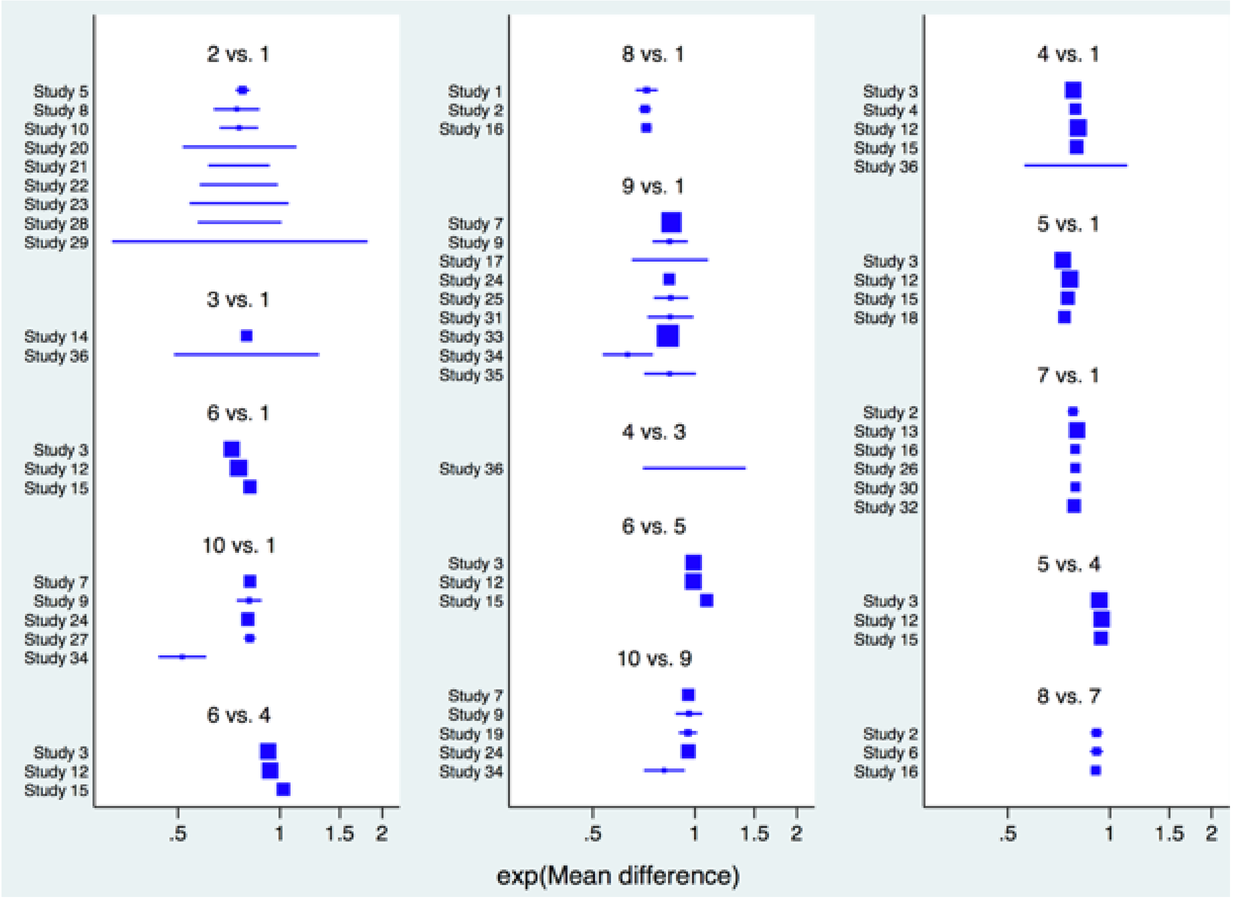

1: **Placebo** 2: **Amitriptyline** 3: **Pregabalin 150mg** 4: **Pregabalin 300mg** 5: **Pregabalin 450mg** 6: **Pregabalin 600mg** 7: **Duloxetine 60mg** 8: **Duloxetine 120mg** 9: **Milnacipran 100mg** 10: **Milnacipran 200mg.**

© 2022 Farag HM et al. *JAMA Network Open.*

eFigure 2B. Pain outcome: Contribution plot

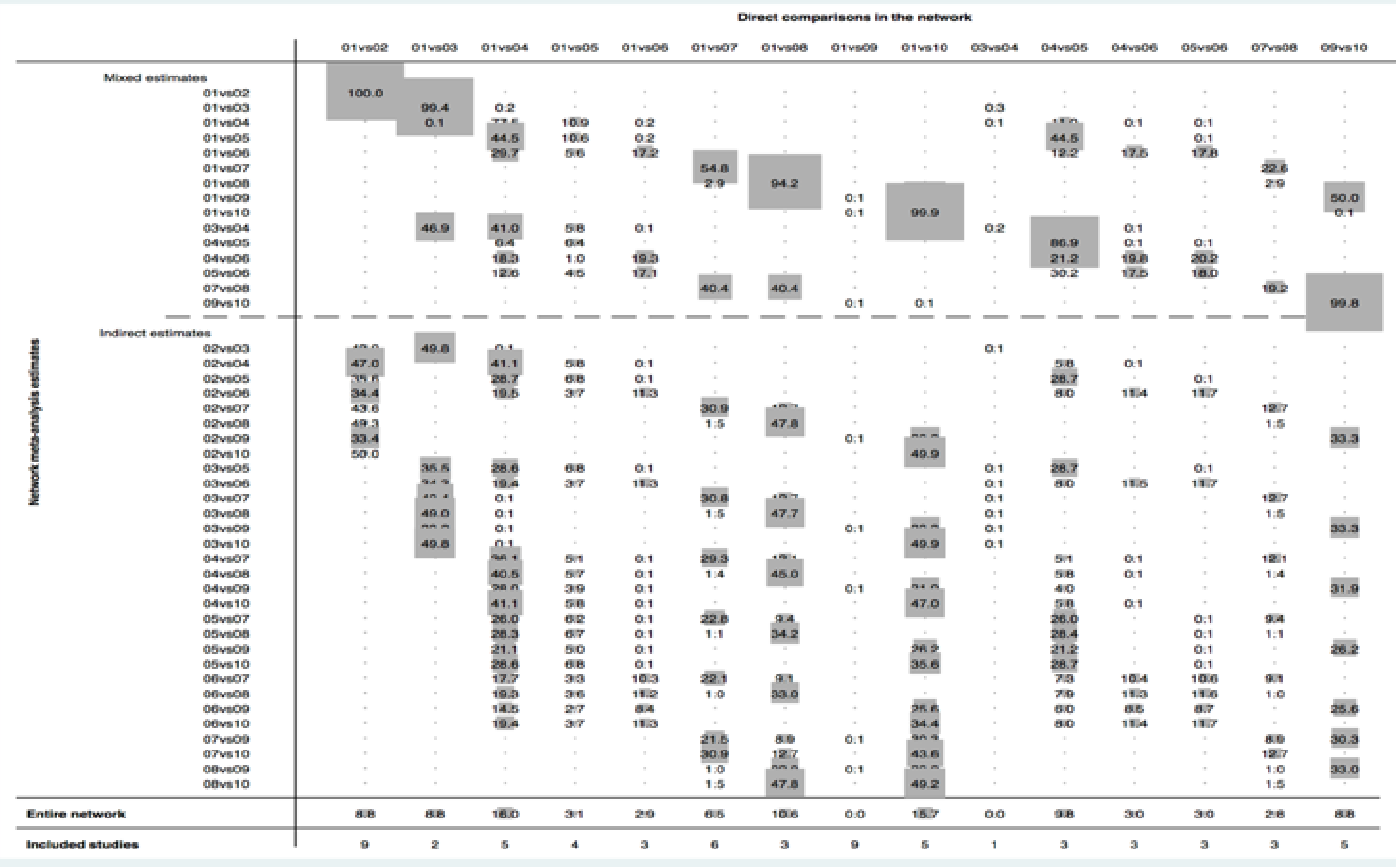

The size of each square is proportional to the weight attached to each direct summary effect (horizontal axis) for the estimation of each network summary effects (vertical axis). The numbers re-express the weights as percentages. 1: **Placebo** 2: **Amitriptyline** 3: **Pregabalin 150mg** 4: **Pregabalin 300mg** 5: **Pregabalin 450mg** 6: **Pregabalin 600mg** 7: **Duloxetine 60mg** 8: **Duloxetine 120mg** 9: **Milnacipran 100mg** 10: **Milnacipran 200mg.**

© 2022 Farag HM et al. *JAMA Network Open.*

eFigure 2C. Pain outcome: Interval plot (estimates as Standardized Mean Difference)

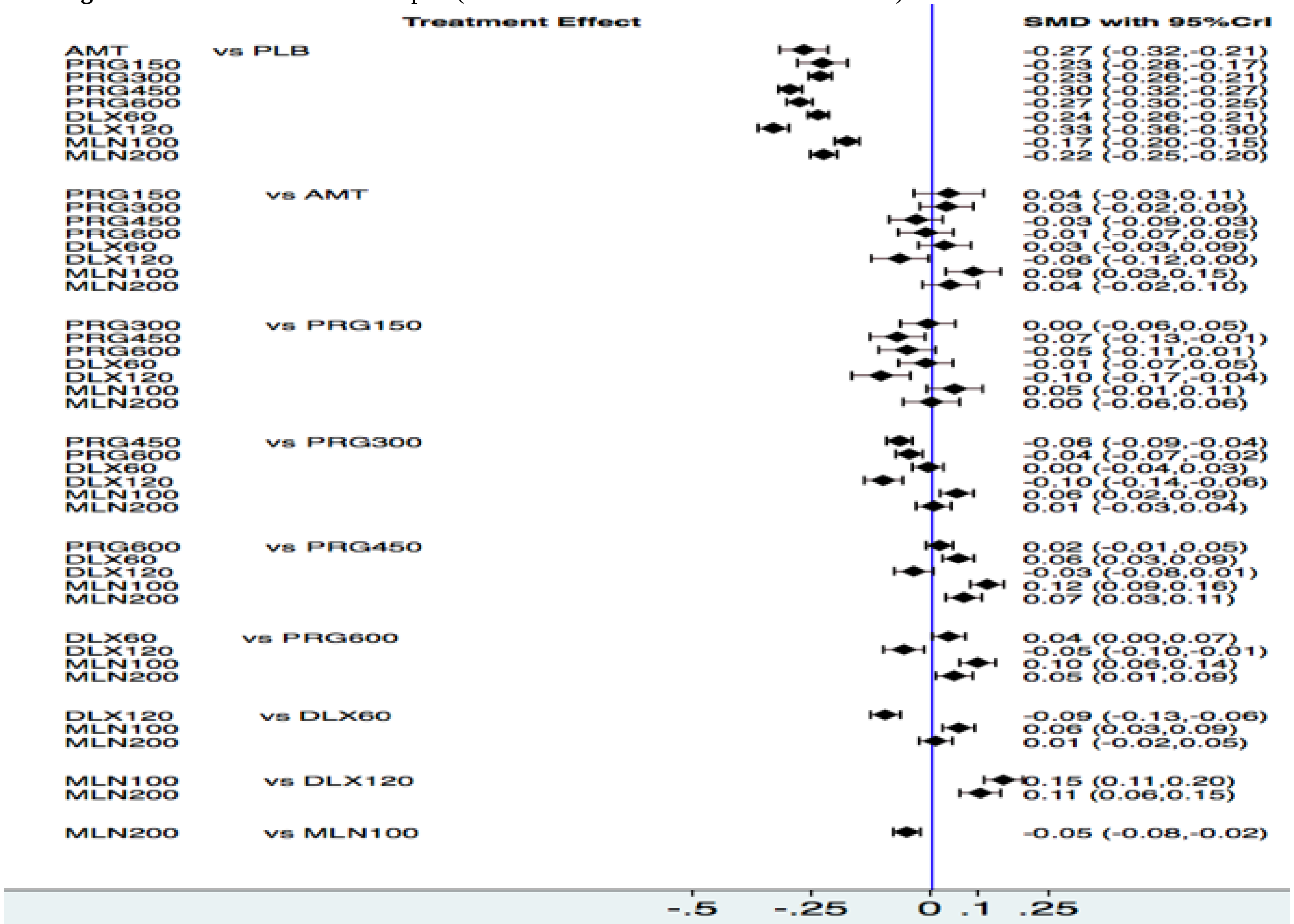

The black solid lines represent the credible intervals for summary standardized mean differences (SMD) for each comparison, and the blue line is the line of no effect (SMD equal to 0). PLB: **Placebo;** AMT: **Amitriptyline;** PRG150: **Pregabalin 150mg;** PRG300: **Pregabalin 300mg;** PRG450: **Pregabalin 450mg;** PRG600: **Pregabalin 600mg;** DLX60: **Duloxetine 60mg;** DLX120: **Duloxetine 120mg;** MLN100: **Milnacipran 100mg;** MLN200: **Milnacipran 200mg.**

eFigure 2C - Pain outcome: interval plot (continued)

© 2022 Farag HM et al. *JAMA Network Open.*

_Comparison	_Effect_Size	_Standard_Error	_LCI	_UCI
AMT vs PLB	-.2661101	.0261206	-.3173055	-.2149147
PRG150 vs PLB	-.2266307	.0269796	-.2795097	-.1737517
PRG300 vs PLB	-.2313645	.0125541	-.2559701	-.2067589
PRG450 vs PLB	-.2953143	.0125744	-.3199598	-.2706689
PRG600 vs PLB	-.2749066	.0139939	-.3023342	-.247479
DLX60 vs PLB	-.2355141	.0111893	-.2574446	-.2135835
DLX120 vs PLB	-.329725	.0164934	-.3620513	-.2973986
MLN100 vs PLB	-.1747631	.0130024	-.2002474	-.1492788
MLN200 vs PLB	-.223886	.0142119	-.2517408	-.1960312
PRG150 vs AMT	.0394794	.0375523	-.0341218	.1130806
PRG300 vs AMT	.0347456	.0289789	-.022052	.0915432
PRG450 vs AMT	-.0292042	.0289871	-.0860179	.0276094
PRG600 vs AMT	-.0087965	.0296494	-.0669081	.0493152
DLX60 vs AMT	.030596	.0284092	-.0250849	.086277
DLX120 vs AMT	-.0636149	.0308894	-.1241571	-.0030727
MLN100 vs AMT	.091347	.0291432	.0342273	.1484667
MLN200 vs AMT	.0422241	.0296469	-.0158827	.1003309
PRG300 vs PRG150	-.0047338	.0296398	-.0628268	.0533591
PRG450 vs PRG150	-.0686837	.0297184	-.1269307	-.0104366
PRG600 vs PRG150	-.0482759	.0303392	-.1077397	.0111879
DLX60 vs PRG150	-.0088834	.0292078	-.0661297	.0483628
DLX120 vs PRG150	-.1030943	.0316216	-.1650715	-.041117
MLN100 vs PRG150	.0518676	.0299492	-.0068318	.1105669
MLN200 vs PRG150	.0027447	.0304936	-.0570217	.0625111
PRG450 vs PRG300	-.0639498	.013714	-.0908288	-.0370709
PRG600 vs PRG300	-.0435421	.0143647	-.0716963	-.0153879
DLX60 vs PRG300	-.0041496	.0168162	-.0371087	.0288095
DLX120 vs PRG300	-.0983605	.0207275	-.1389855	-.0577354
MLN100 vs PRG300	.0566014	.0180711	.0211827	.0920201
MLN200 vs PRG300	.0074785	.0189554	-.0296735	.0446305
PRG600 vs PRG450	.0204078	.0143725	-.0077618	.0485774
DLX60 vs PRG450	.0598003	.0168312	.0268118	.0927888
DLX120 vs PRG450	-.0344106	.0207397	-.0750597	.0062385
MLN100 vs PRG450	.1205512	.0180843	.0851067	.1559958
MLN200 vs PRG450	.0714284	.0189665	.0342546	.1086021
DLX60 vs PRG600	.0393925	.0179222	.0042656	.0745194
DLX120 vs PRG600	-.0548184	.0216317	-.0972157	-.0124211
MLN100 vs PRG600	.1001435	.0191253	.0626586	.1376283
MLN200 vs PRG600	.0510206	.0200034	.0118147	.0902265
DLX120 vs DLX60	-.0942109	.0160762	-.1257197	-.0627021
MLN100 vs DLX60	.060751	.0171434	.0271506	.0943513
MLN200 vs DLX60	.0116281	.0180612	-.0237712	.0470274
MLN100 vs DLX120	.1549619	.0209988	.1138049	.1961188
MLN200 vs DLX120	.105839	.021763	.0631842	.1484937
MLN200 vs MLN100	-.0491229	.0143425	-.0772336	-.0210122

PLB: **Placebo;** AMT: **Amitriptyline;** PRG150: **Pregabalin 150mg;** PRG300: **Pregabalin 300mg;** PRG450: **Pregabalin 450mg;** PRG600: **Pregabalin 600mg;** DLX60: **Duloxetine 60mg;** DLX120: **Duloxetine 120mg;** MLN100: **Milnacipran 100mg;** MLN200: **Milnacipran 200mg.**

© 2022 Farag HM et al. *JAMA Network Open.*

eFigure 2D - Pain outcome: Surface under the cumulative ranking curves (SUCRAs) plots

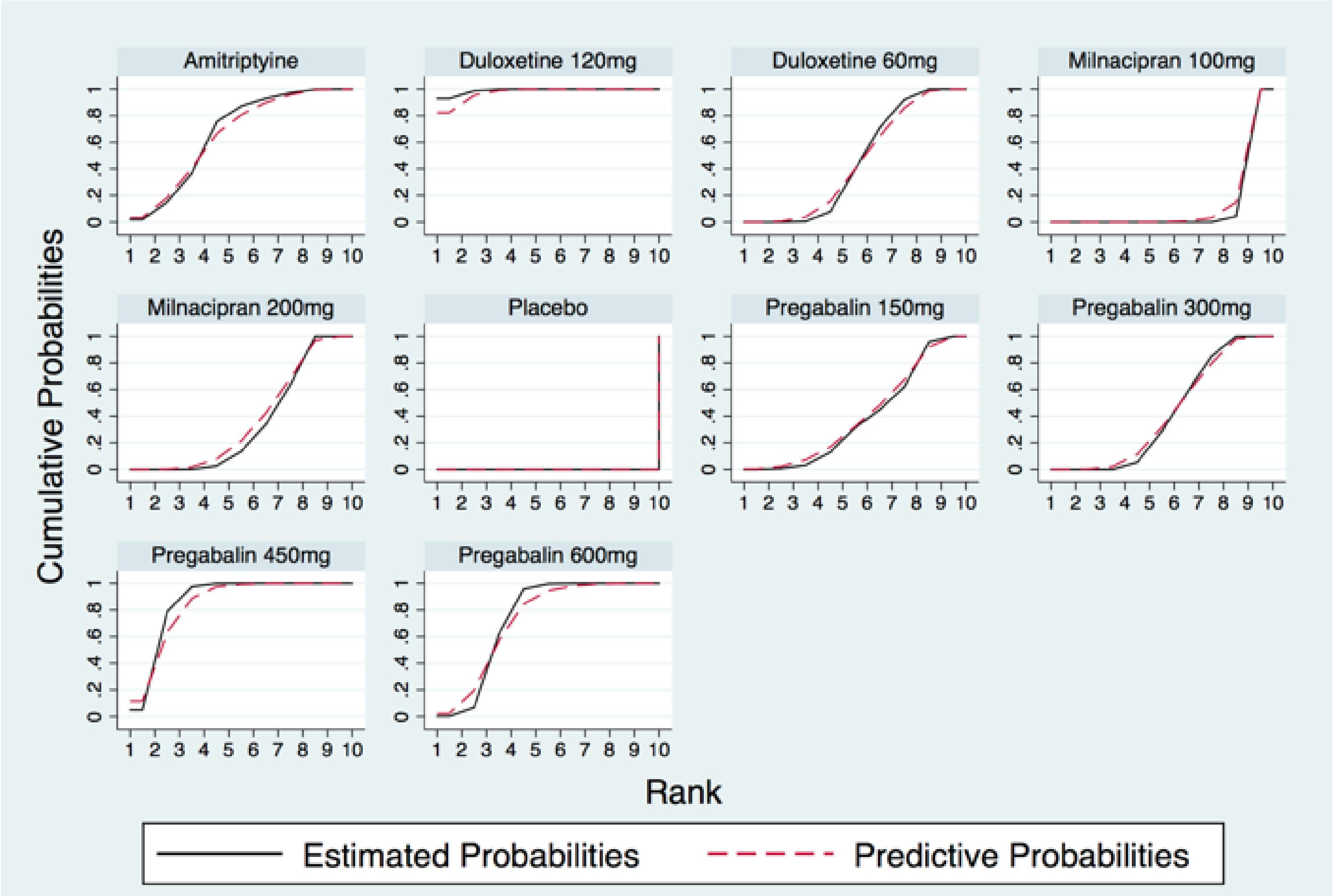

Cumulative probability curves for the fibromyalgia treatments network showing the estimated and predictive probabilities for each treatment being up to a specific rank.

eFigure 2D - Pain outcome: Surface under the cumulative ranking curves (SUCRAs) plots (continued)

© 2022 Farag HM et al. *JAMA Network Open.*

Treatment	SUCRA	PrBest	MeanRank
Placebo	0.0	0.0	10.0
Amitriptyine	67.4	1.9	3.9
Pregabalin 150mg	39.0	0.0	6.5
Pregabalin 300mg	41.8	0.0	6.2
Pregabalin 450mg	86.8	4.9	2.2
Pregabalin 600mg	73.8	0.3	3.4
Duloxetine 60mg	45.7	0.0	5.9
Duloxetine 120mg	99.1	92.9	1.1
Milnacipran 100mg	11.6	0.0	9.0
Milnacipran 200mg	34.9	0.0	6.9

© 2022 Farag HM et al. *JAMA Network Open.*

eFigure 2E - Pain outcome: rankogram plots

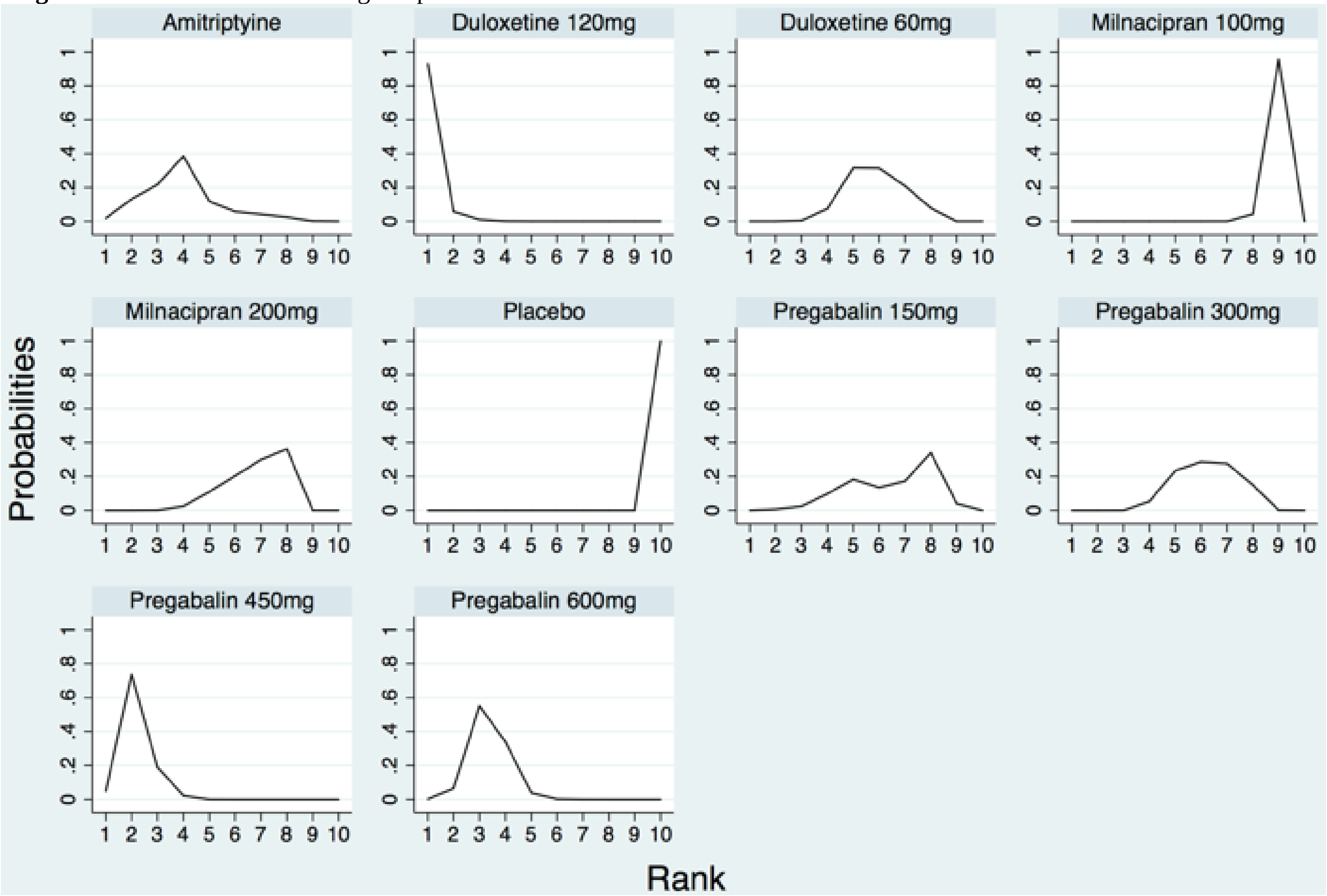

Rankogram plots for the fibromyalgia treatments network showing the probability for every treatment being at particular order.

eFigure 2E - Pain outcome: rankogram plots (continued)

© 2022 Farag HM et al. *JAMA Network Open.*

id and Rank	Treatment 1	2	3	4	5	6	7	8	9	10
1										
Best	**0.0**	**3.5**	**0.3**	**0.0**	**11.6**	**2.3**	**0.0**	**82.3**	**0.0**	**0.0**
2nd	**0.0**	**14.9**	**1.9**	**0.3**	**51.6**	**17.4**	**0.6**	**13.1**	**0.0**	**0.2**
3rd	**0.0**	**22.2**	**5.0**	**1.8**	**25.5**	**37.0**	**3.3**	**3.5**	**0.0**	**1.5**
4th	**0.0**	**25.8**	**9.8**	**9.2**	**8.9**	**27.8**	**11.7**	**0.9**	**0.0**	**5.9**
5th	**0.0**	**14.3**	**15.3**	**20.6**	**1.6**	**10.1**	**24.2**	**0.1**	**0.2**	**13.7**
6th	**0.0**	**9.2**	**16.2**	**24.1**	**0.6**	**3.6**	**24.9**	**0.0**	**0.5**	**21.0**
7th	**0.0**	**6.0**	**19.0**	**23.7**	**0.1**	**1.4**	**21.3**	**0.0**	**2.4**	**26.1**
8th	**0.0**	**3.7**	**24.7**	**18.4**	**0.0**	**0.4**	**12.7**	**0.0**	**11.6**	**28.6**
9th	**0.0**	**0.5**	**8.0**	**1.9**	**0.0**	**0.0**	**1.3**	**0.0**	**85.2**	**3.0**
Worst	**100.0**	**0.0**	**0.0**	**0.0**	**0.0**	**0.0**	**0.0**	**0.0**	**0.0**	**0.0**

1: **Placebo** 2: **Amitriptyline** 3: **Pregabalin 150mg** 4: **Pregabalin 300mg** 5: **Pregabalin 450mg** 6: **Pregabalin 600mg** 7: **Duloxetine 60mg** 8: **Duloxetine 120mg** 9: **Milnacipran 100mg** 10: **Milnacipran 200mg.**

© 2022 Farag HM et al. *JAMA Network Open.*

eFigure 2F. Pain outcome: comparison adjusted funnel plot

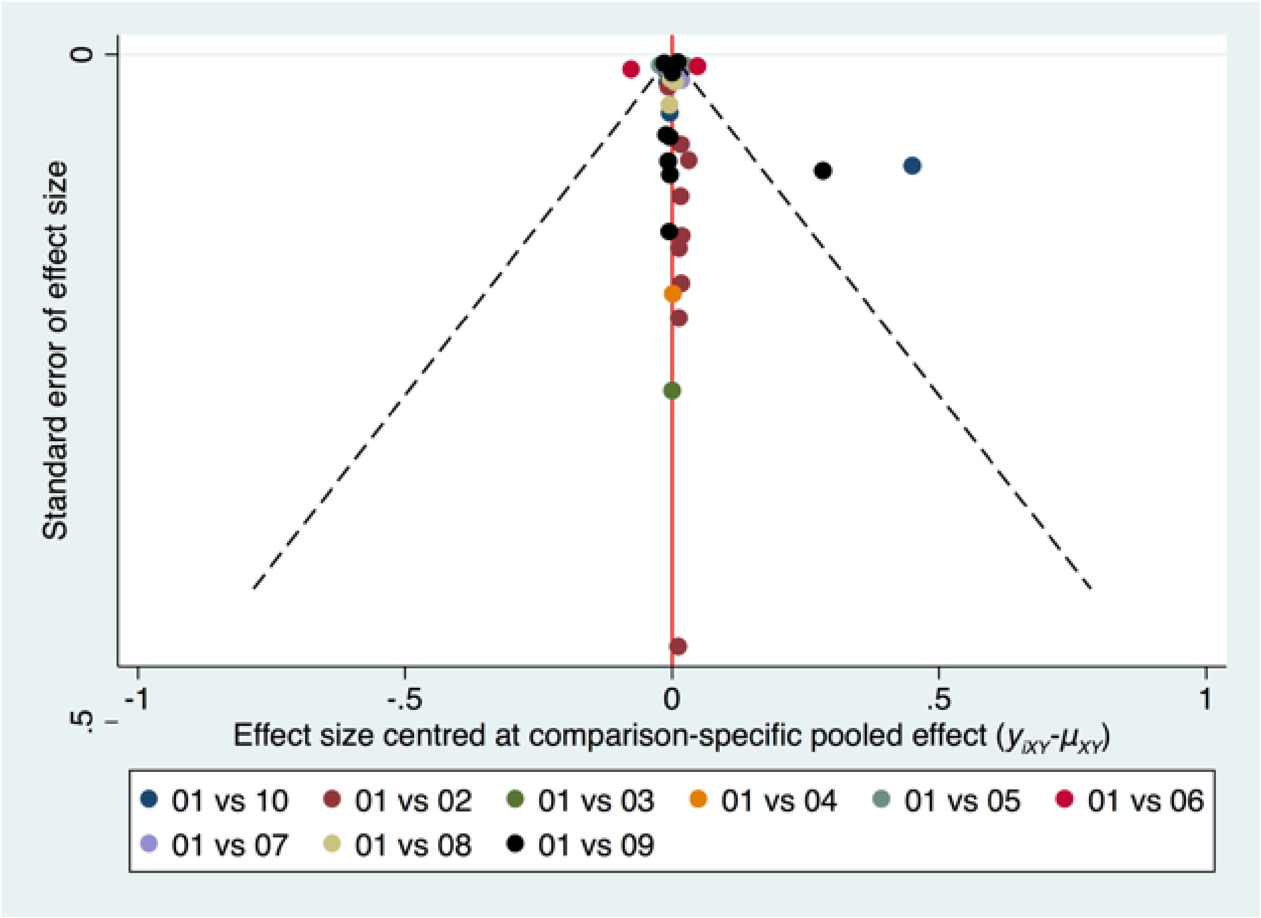

The red line represents the null hypothesis that the study-specific effect sizes do not differ from the respective comparison-specific pooled effect estimates. Different colors correspond to different comparisons. 1: **Placebo** 2: **Amitriptyline** 3: **Pregabalin 150mg** 4: **Pregabalin 300mg** 5: **Pregabalin 450mg** 6: **Pregabalin 600mg** 7: **Duloxetine 60mg** 8: **Duloxetine 120mg** 9: **Milnacipran 100mg** 10: **Milnacipran 200mg.**

© 2022 Farag HM et al. *JAMA Network Open.*

eFigure 2G. Pain outcome network pattern

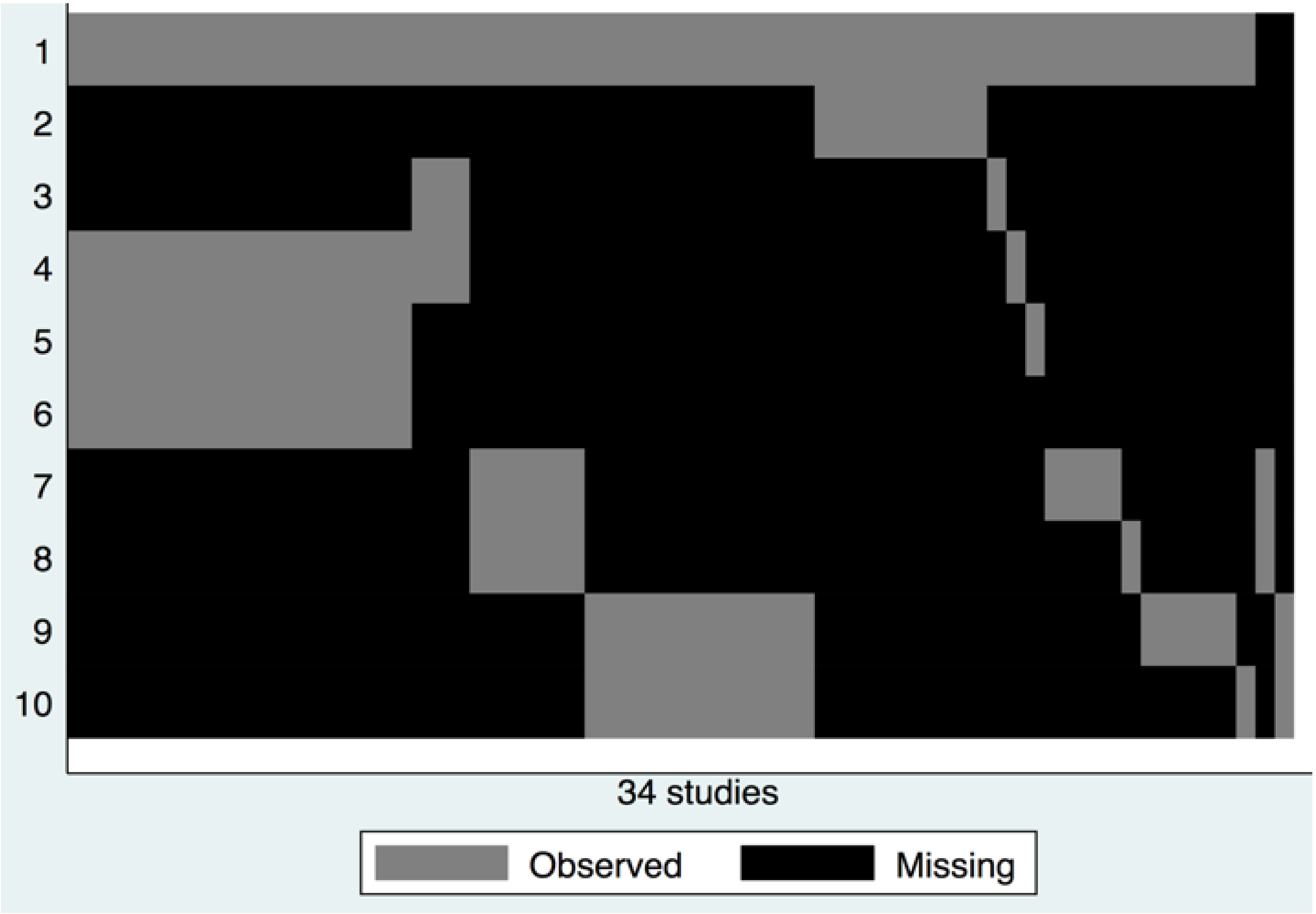

eFigure 3. Sleep Outcome: Results

© 2022 Farag HM et al. *JAMA Network Open.*

eFigure 3A. Sleep outcome: network forest

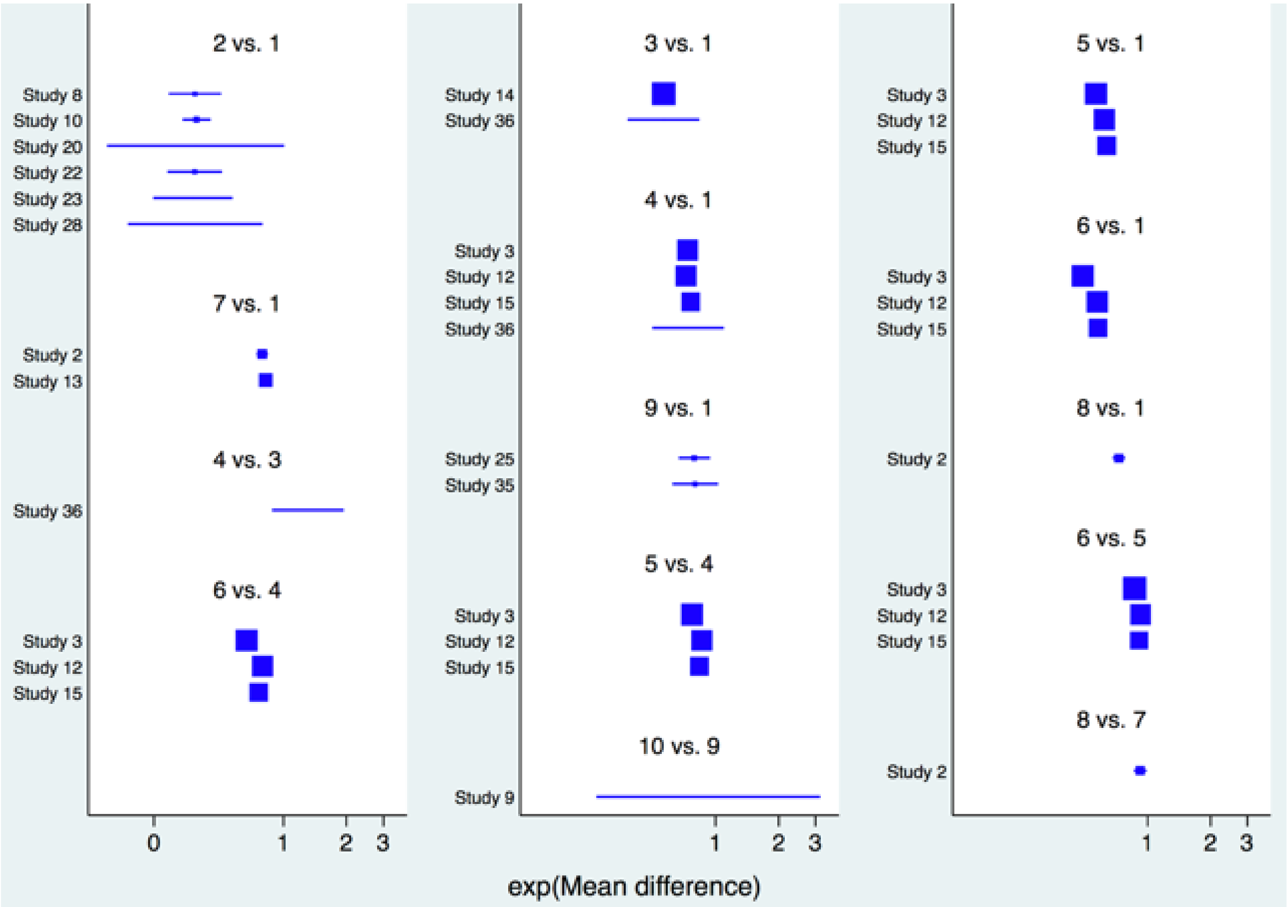

1: **Placebo** 2: **Amitriptyline** 3: **Pregabalin 150mg** 4: **Pregabalin 300mg** 5: **Pregabalin 450mg** 6: **Pregabalin 600mg** 7: **Duloxetine 60mg** 8: **Duloxetine 120mg** 9: **Milnacipran 100mg** 10: **Milnacipran 200mg.**

© 2022 Farag HM et al. *JAMA Network Open.*

eFigure 3B. Sleep outcome: contribution plot

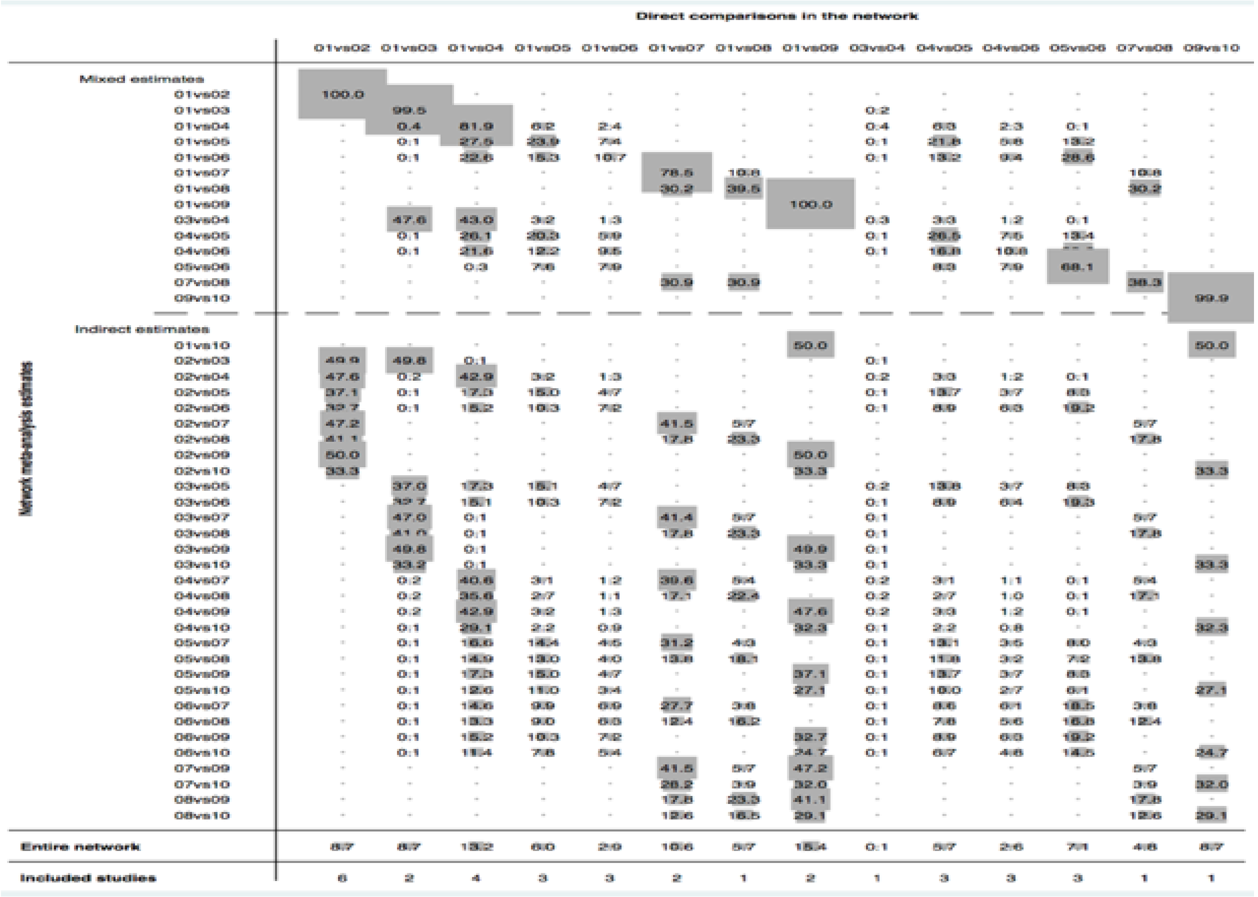

The size of each square is proportional to the weight attached to each direct summary effect (horizontal axis) for the estimation of each network summary effects (vertical axis). The numbers re-express the weights as percentages. 1: **Placebo** 2: **Amitriptyline** 3: **Pregabalin 150mg** 4: **Pregabalin 300mg** 5: **Pregabalin 450mg** 6: **Pregabalin 600mg** 7: **Duloxetine 60mg** 8: **Duloxetine 120mg** 9: **Milnacipran 100mg** 10: **Milnacipran 200mg.**

© 2022 Farag HM et al. *JAMA Network Open.*

eFigure 3C. Sleep outcome: interval plot (estimates as Standardized Mean Difference)

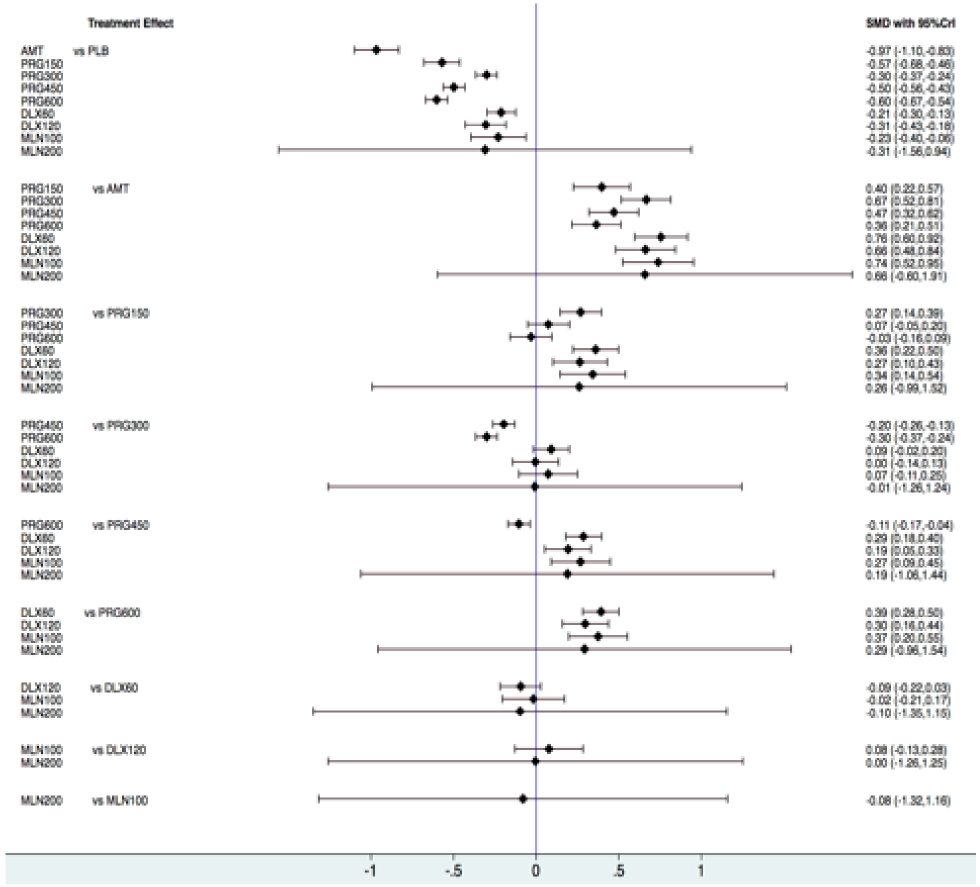

The black solid lines represent the credible intervals for summary standardized mean differences (SMD) for each comparison, and the blue line is the line of no effect (SMD equal to 0). CrI indicates credible interval; SMD, standardized mean difference. PLB: **Placebo;** AMT: **Amitriptyline;** PRG150: **Pregabalin 150mg;** PRG300: **Pregabalin 300mg;** PRG450: **Pregabalin 450mg;** PRG600: **Pregabalin 600mg;** DLX60: **Duloxetine 60mg;** DLX120: **Duloxetine 120mg;** MLN100: **Milnacipran 100mg;** MLN200: **Milnacipran 200mg.**

eFigure 3C. Sleep outcome: interval plot (estimates as Standardized Mean Difference)

© 2022 Farag HM et al. *JAMA Network Open.*

_Comparison	_Effect_Size	_Standard_Error	_LCI	_UCI
AMT vs PLB	−.9680143	.0684899	−1.102252	−.8337766
PRG150 vs PLB	−.5717611	.0554033	−.6803496	−.4631727
PRG300 vs PLB	−.3022757	.0332076	−.3673614	−.2371901
PRG450 vs PLB	−.4984311	.0336124	−.5643102	−.432552
PRG600 vs PLB	−.6039517	.033631	−.6698672	−.5380362
DLX60 vs PLB	−.212104	.0444286	−.2991824	−.1250256
DLX120 vs PLB	−.3067529	.062817	−.429872	−.1836339
MLN100 vs PLB	−.2296359	.0848137	−.3958677	−.0634042
MLN200 vs PLB	−.3106359	.6375068	−1.560126	.9388544
PRG150 vs AMT	.3962532	.0880933	.2235935	.5689128
PRG300 vs AMT	.6657386	.0761161	.5165538	.8149234
PRG450 vs AMT	.4695832	.0762942	.3200493	.6191171
PRG600 vs AMT	.3640626	.0763031	.2145112	.513614
DLX60 vs AMT	.7559103	.0816327	.5959131	.9159074
DLX120 vs AMT	.6612613	.0929325	.479117	.8434056
MLN100 vs AMT	.7383783	.1090157	.5247115	.9520451
MLN200 vs AMT	.6573784	.6411754	−.5993024	1.914059
PRG300 vs PRG150	.2694854	.0637882	.1444628	.394508
PRG450 vs PRG150	.07333	.0644023	−.0528961	.1995561
PRG600 vs PRG150	−.0321906	.0644119	−.1584356	.0940545
DLX60 vs PRG150	.3596571	.0710173	.2204658	.4988485
DLX120 vs PRG150	.2650082	.0837587	.1008442	.4291722
MLN100 vs PRG150	.3421252	.1013059	.1435693	.540681
MLN200 vs PRG150	.2611252	.6399097	−.9930747	1.515325
PRG450 vs PRG300	−.1961554	.0336133	−.2620363	−.1302744
PRG600 vs PRG300	−.301676	.0336318	−.3675932	−.2357588
DLX60 vs PRG300	.0901717	.055468	−.0185435	.1988869
DLX120 vs PRG300	−.0044772	.0710545	−.1437415	.1347871
MLN100 vs PRG300	.0726398	.0910829	−.1058794	.251159
MLN200 vs PRG300	−.0083602	.6383711	−1.259544	1.242824
PRG600 vs PRG450	−.1055206	.0337557	−.1716807	−.0393606
DLX60 vs PRG450	.2863271	.0557121	.1771335	.3955207
DLX120 vs PRG450	.1916781	.0712449	.0520407	.3313156
MLN100 vs PRG450	.2687952	.0912312	.0899854	.4476049
MLN200 vs PRG450	.1877952	.6383922	−1.063431	1.439021
DLX60 vs PRG600	.3918477	.0557242	.2826303	.5010652
DLX120 vs PRG600	.2971988	.071254	.1575435	.4368541
MLN100 vs PRG600	.3743158	.0912379	.1954928	.5531387
MLN200 vs PRG600	.2933158	.6383932	−.9579119	1.544543
DLX120 vs DLX60	−.094649	.0629151	−.2179603	.0286623
MLN100 vs DLX60	−.017532	.0957468	−.2051922	.1701283
MLN200 vs DLX60	−.0985319	.6390532	−1.351053	1.153989
MLN100 vs DLX120	.077117	.1055435	−.1297444	.2839784
MLN200 vs DLX120	−.003883	.6405942	−1.259425	1.251659
MLN200 vs MLN100	−.081	.6318398	−1.319383	1.157383

PLB: **Placebo;** AMT: **Amitriptyline;** PRG150: **Pregabalin 150mg;** PRG300: **Pregabalin 300mg;** PRG450: **Pregabalin 450mg;** PRG600: **Pregabalin 600mg;** DLX60: **Duloxetine 60mg;** DLX120: **Duloxetine 120mg;** MLN100: **Milnacipran 100mg;** MLN200: **Milnacipran 200mg.**

© 2022 Farag HM et al. *JAMA Network Open.*

eFigure 3D. Sleep outcome: SUCRA plots

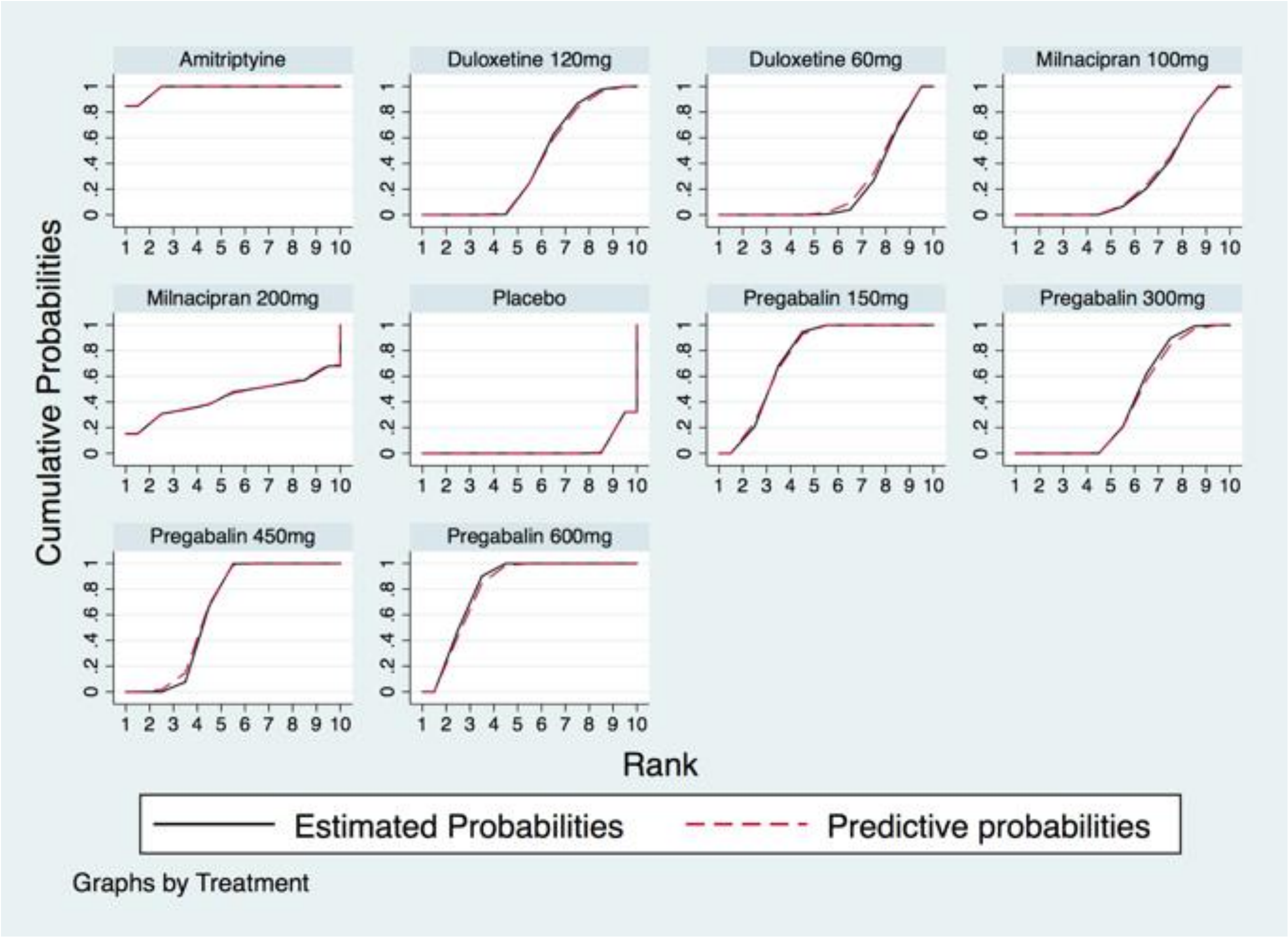

Cumulative probability curves for the fibromyalgia treatments network showing the estimated and predictive probabilities for each treatment being up to a specific rank.

eFigure 3D. Sleep outcome: SUCRA plots (continued)

© 2022 Farag HM et al. *JAMA Network Open.*

Treatment	SUCRA	PrBest	MeanRank
Placebo	3.6	0.0	9.7
Amitriptyine	98.3	84.6	1.2
Pregabalin 150mg	76.0	0.0	3.2
Pregabalin 300mg	41.3	0.0	6.3
Pregabalin 450mg	63.8	0.0	4.3
Pregabalin 600mg	82.0	0.0	2.6
Duloxetine 60mg	22.2	0.0	8.0
Duloxetine 120mg	41.4	0.0	6.3
Milnacipran 100mg	27.5	0.0	7.5
Milnacipran 200mg	44.0	15.4	6.0

© 2022 Farag HM et al. *JAMA Network Open.*

eFigure 3E. Sleep outcome: rankogram plots

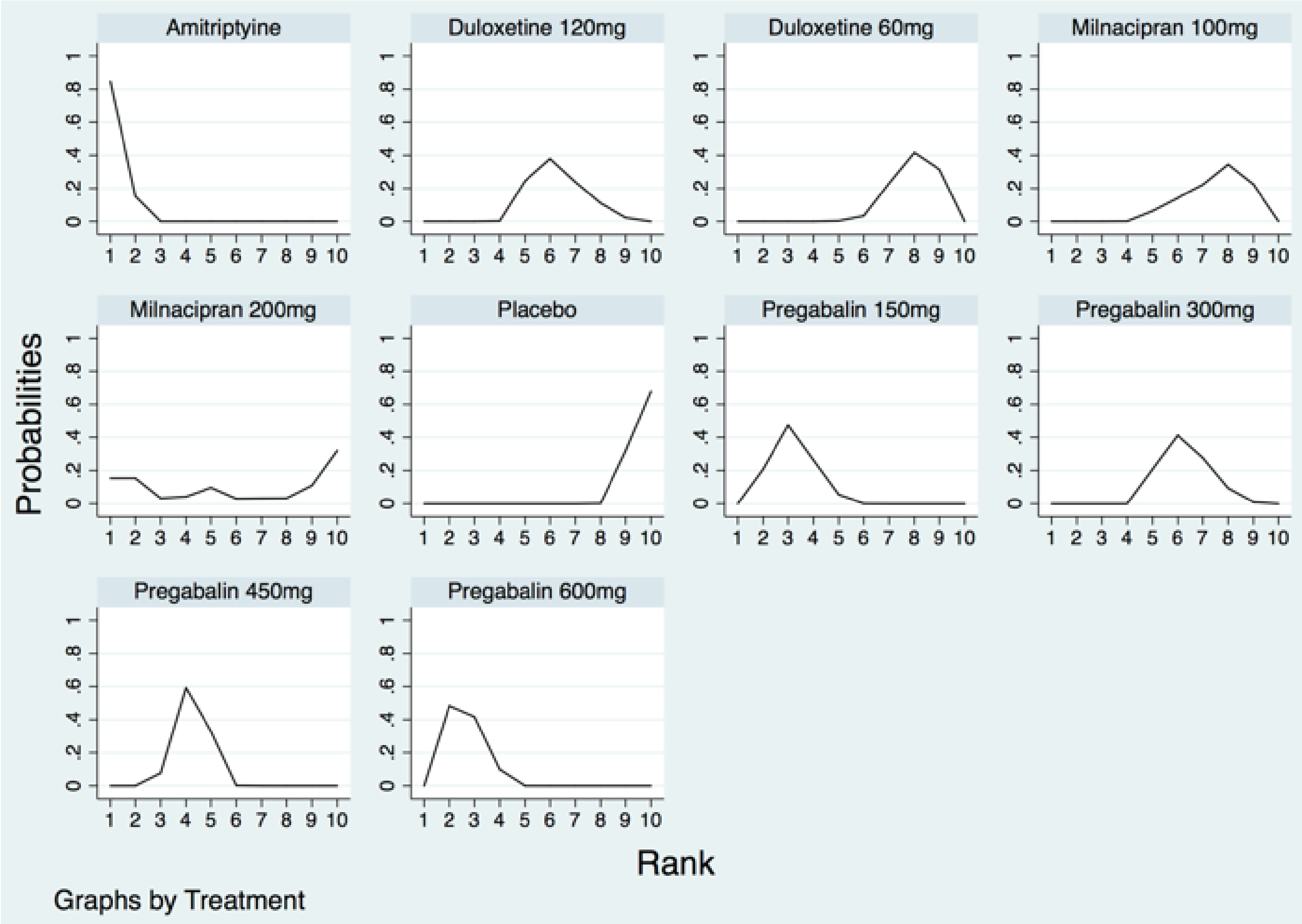

Rankogram plots for the fibromyalgia treatments network showing the probability for every treatment being at particular order.

eFigure 3E. Sleep outcome: rankogram plots (continued)

© 2022 Farag HM et al. *JAMA Network Open.*

id and Rank	Treatment 1	2	3	4	5	6	7	8	9	10
2										
Best	**0.0**	**84.5**	**0.0**	**0.0**	**0.0**	**0.0**	**0.0**	**0.0**	**0.0**	**15.5**
2nd	**0.0**	**15.5**	**20.9**	**0.0**	**0.0**	**46.5**	**0.0**	**0.0**	**0.0**	**17.0**
3rd	**0.0**	**0.0**	**45.9**	**0.0**	**8.0**	**43.0**	**0.0**	**0.0**	**0.0**	**3.0**
4th	**0.0**	**0.0**	**27.7**	**0.0**	**57.7**	**10.4**	**0.0**	**0.2**	**0.1**	**4.0**
5th	**0.0**	**0.0**	**5.5**	**19.8**	**34.1**	**0.0**	**0.2**	**25.0**	**6.0**	**9.4**
6th	**0.0**	**0.0**	**0.0**	**41.3**	**0.2**	**0.0**	**3.4**	**36.8**	**15.6**	**2.8**
7th	**0.0**	**0.0**	**0.0**	**28.9**	**0.0**	**0.0**	**21.8**	**24.9**	**21.6**	**2.9**
8th	**0.1**	**0.0**	**0.0**	**9.1**	**0.0**	**0.0**	**42.2**	**11.0**	**34.1**	**3.5**
9th	**32.0**	**0.0**	**0.0**	**1.0**	**0.0**	**0.0**	**32.4**	**2.2**	**22.5**	**10.0**
Worst	**67.9**	**0.0**	**0.0**	**0.0**	**0.0**	**0.0**	**0.0**	**0.0**	**0.2**	**31.9**

1: **Placebo** 2: **Amitriptyline** 3: **Pregabalin 150mg** 4: **Pregabalin 300mg** 5: **Pregabalin 450mg** 6: **Pregabalin 600mg** 7: **Duloxetine 60mg** 8: **Duloxetine 120mg** 9: **Milnacipran 100mg** 10: **Milnacipran 200mg.**

© 2022 Farag HM et al. *JAMA Network Open.*

eFigure 3F. Sleep outcome: comparison adjusted funnel plot

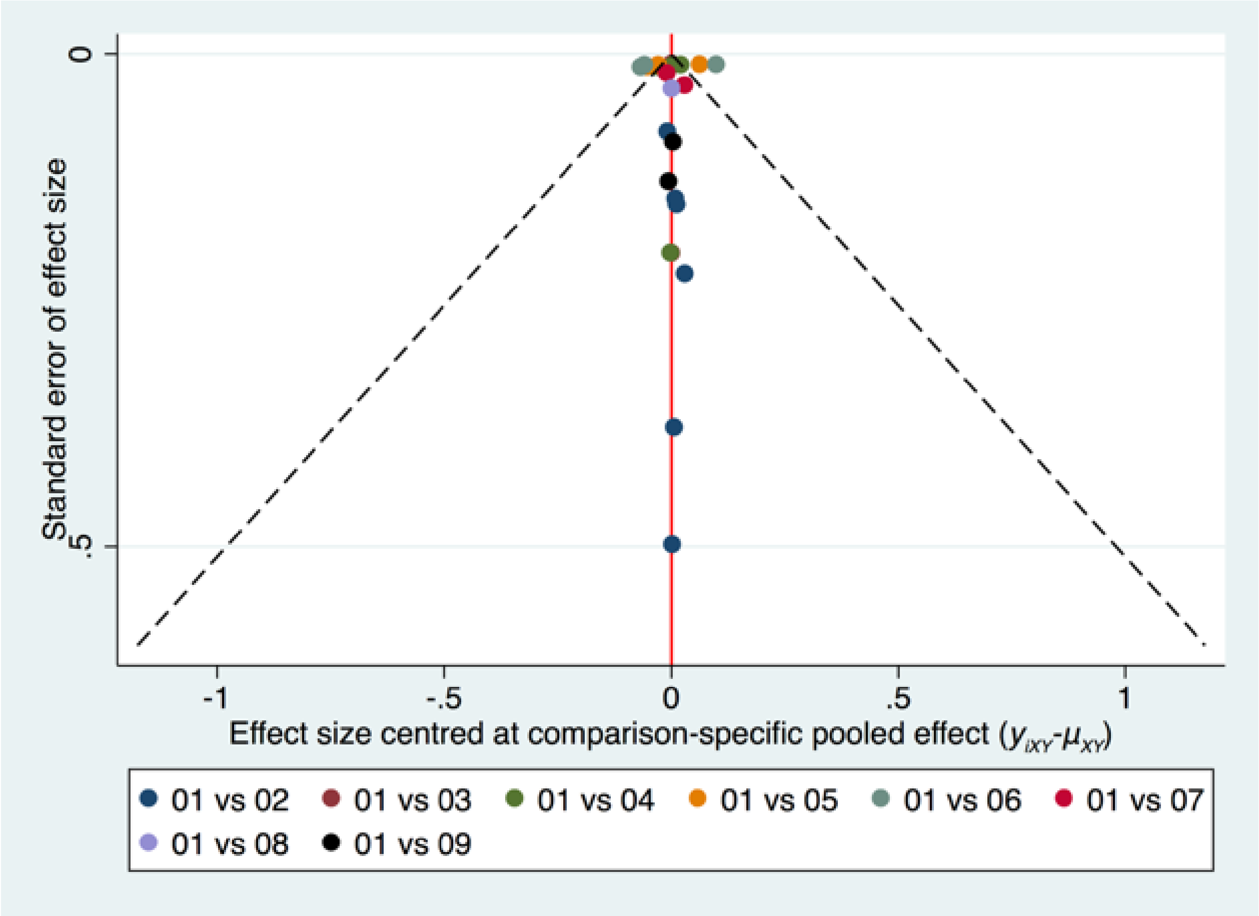

The red line represents the null hypothesis that the study-specific effect sizes do not differ from the respective comparison-specific pooled effect estimates. Different colors correspond to different comparisons. 1: **Placebo** 2: **Amitriptyline** 3: **Pregabalin 150mg** 4: **Pregabalin 300mg** 5: **Pregabalin 450mg** 6: **Pregabalin 600mg** 7: **Duloxetine 60mg** 8: **Duloxetine 120mg** 9: **Milnacipran 100mg** 10: **Milnacipran 200mg.**

© 2022 Farag HM et al. *JAMA Network Open.*

eFigure 3G. Sleep outcome network pattern

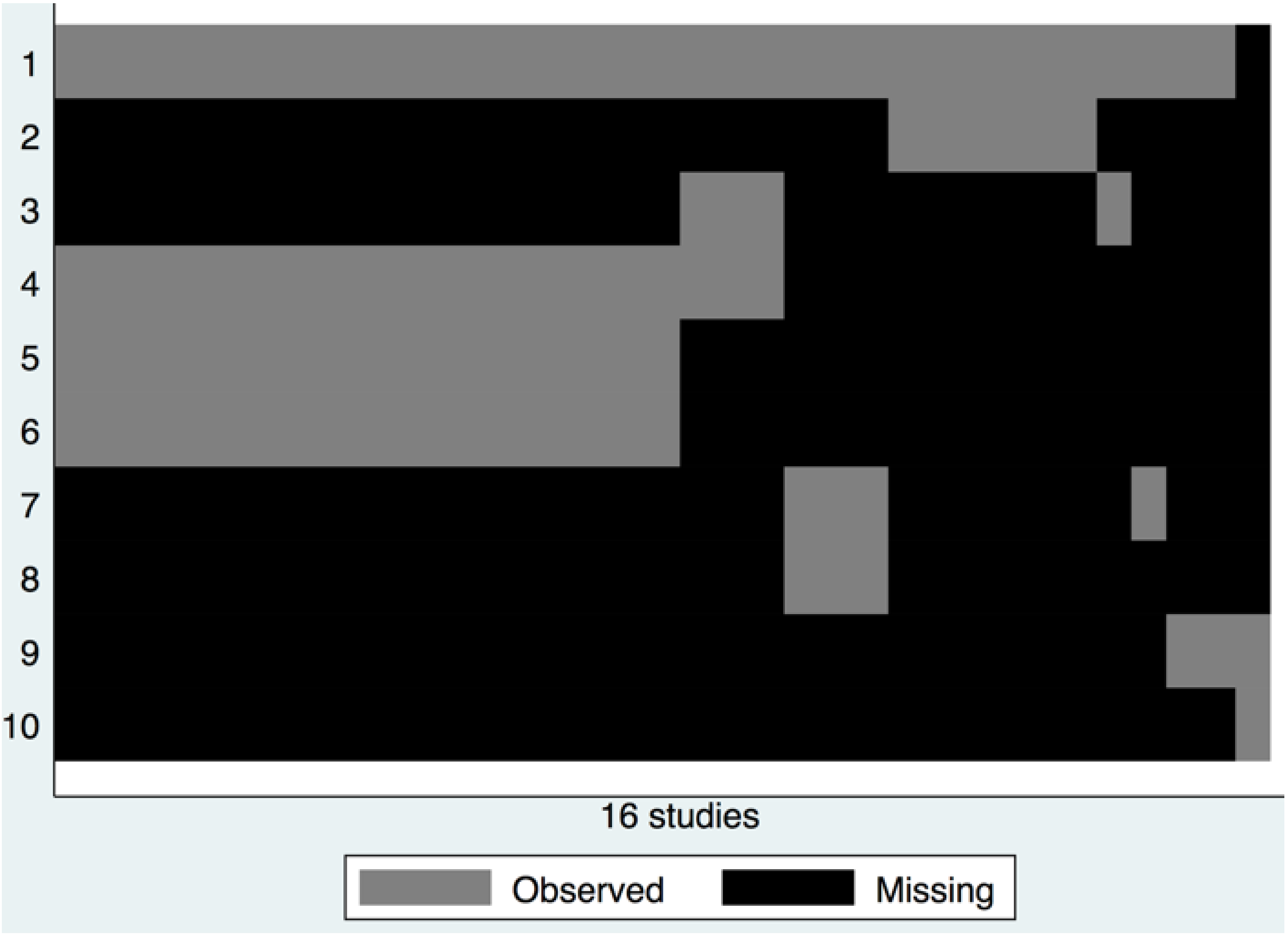

eFigure 4. Depression Outcome: Results

© 2022 Farag HM et al. *JAMA Network Open.*

eFigure 4A. Depression outcome: network forest

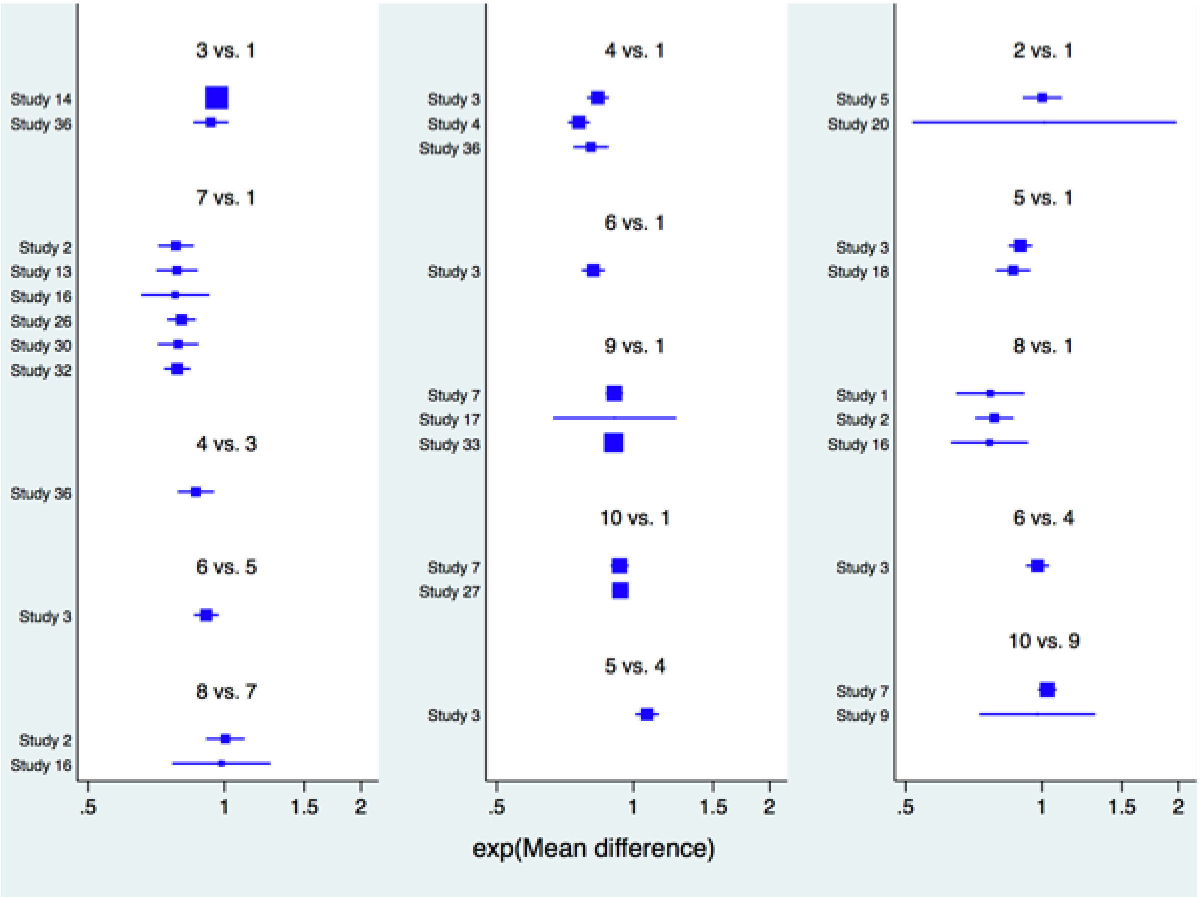

1: **Placebo** 2: **Amitriptyline** 3: **Pregabalin 150mg** 4: **Pregabalin 300mg** 5: **Pregabalin 450mg** 6: **Pregabalin 600mg** 7: **Duloxetine 60mg** 8: **Duloxetine 120mg** 9: **Milnacipran 100mg** 10: **Milnacipran 200mg.**

© 2022 Farag HM et al. *JAMA Network Open.*

eFigure 4B. Depression outcome: contribution plot

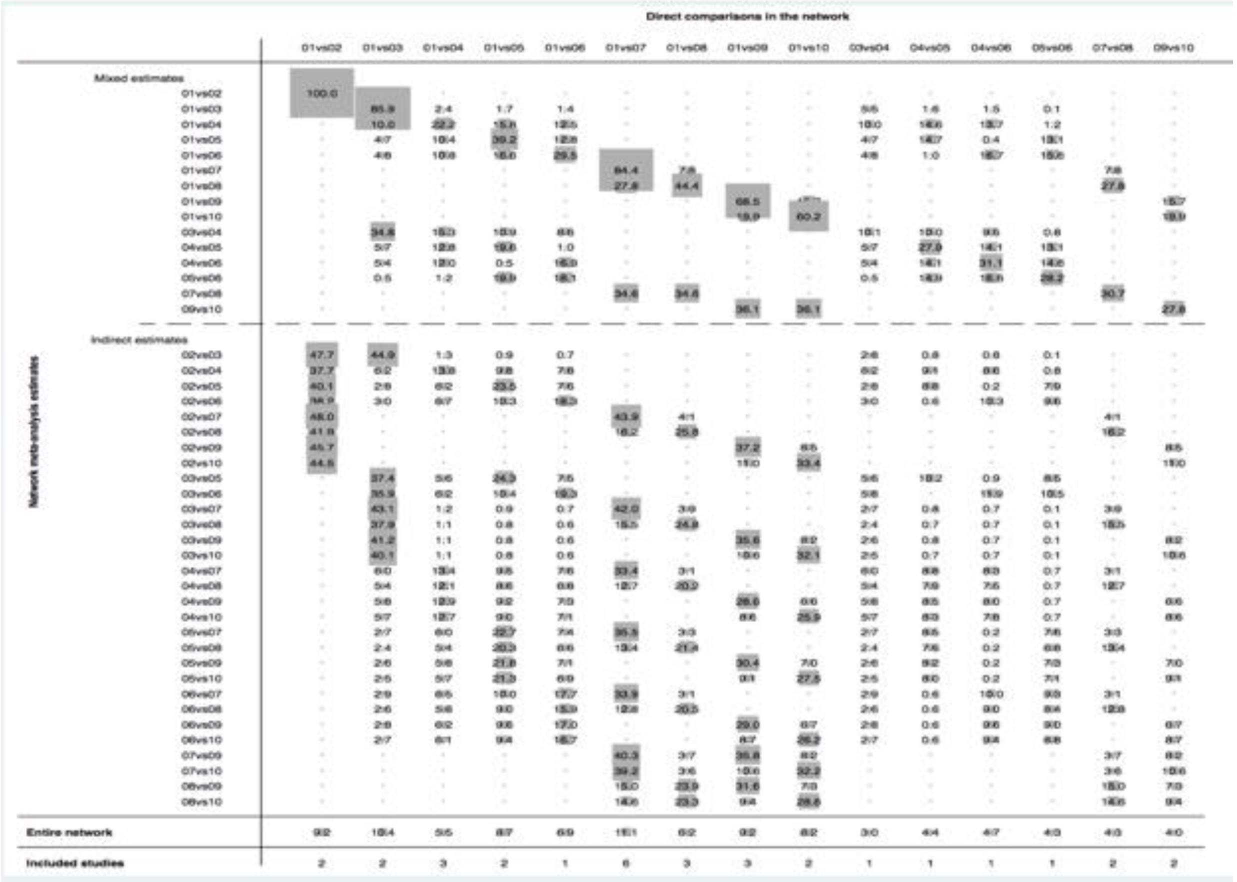

The size of each square is proportional to the weight attached to each direct summary effect (horizontal axis) for the estimation of each network summary effects (vertical axis). The numbers re-express the weights as percentages. 1: **Placebo** 2: **Amitriptyline** 3: **Pregabalin 150mg** 4: **Pregabalin 300mg** 5: **Pregabalin 450mg** 6: **Pregabalin 600mg** 7: **Duloxetine 60mg** 8: **Duloxetine 120mg** 9: **Milnacipran 100mg** 10: **Milnacipran 200mg.**

© 2022 Farag HM et al. *JAMA Network Open.*

eFigure 4C. Depression outcome: interval plot (estimates as Standardized Mean Difference)

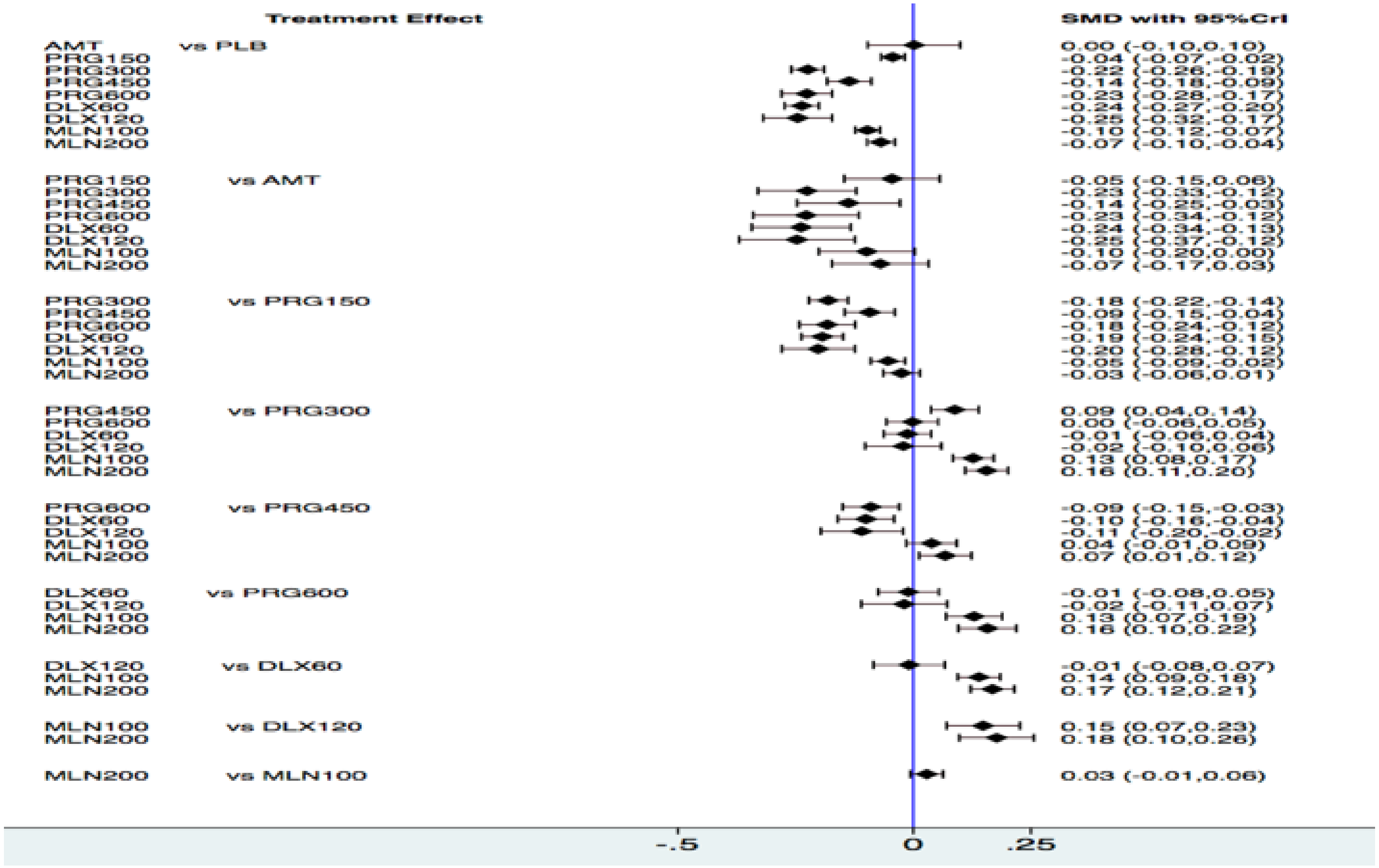

The black solid lines represent the credible intervals for summary standardized mean differences (SMD) for each comparison, and the blue line is the line of no effect (SMD equal to 0). CrI indicates credible interval; SMD, standardized mean difference. PLB: **Placebo;** AMT: **Amitriptyline;** PRG150: **Pregabalin 150mg;** PRG300: **Pregabalin 300mg;** PRG450: **Pregabalin 450mg;** PRG600: **Pregabalin 600mg;** DLX60: **Duloxetine 60mg;** DLX120: **Duloxetine 120mg;** MLN100: **Milnacipran 100mg;** MLN200: **Milnacipran 200mg.**

eFigure 4C. Depression outcome: interval plot (continued)

© 2022 Farag HM et al. *JAMA Network Open.*

_Comparison	_Effect_Size	_Standard_Error	_LCI	_UCI	_LPrI	_UPrI
AMT vs PLB	.0014892	.0502141	-.0969286	.099907	-.1583145	.1612928
PRG150 vs PLB	-.04368	.0129372	-.0690363	-.0183236	-.0848518	-.0025081
PRG300 vs PLB	-.2244603	.0177476	-.259245	-.1896756	-.2809412	-.1679794
PRG450 vs PLB	-.1363761	.0240555	-.1835241	-.0892282	-.2129315	-.0598207
PRG600 vs PLB	-.2263874	.0274302	-.2801496	-.1726251	-.3136826	-.1390921
DLX60 vs PLB	-.2368637	.018531	-.2731838	-.2005437	-.2958376	-.1778899
DLX120 vs PLB	-.2457426	.0373489	-.3189451	-.1725401	-.3646035	-.1268818
MLN100 vs PLB	-.0974469	.0131712	-.123262	-.0716318	-.1393635	-.0555303
MLN200 vs PLB	-.0688587	.0150025	-.0982632	-.0394543	-.1166035	-.0211139
PRG150 vs AMT	-.0451691	.0518539	-.1468009	.0564626	-.2101913	.119853
PRG300 vs AMT	-.2259495	.0532582	-.3303336	-.1215654	-.3954407	-.0564582
PRG450 vs AMT	-.1378653	.0556787	-.2469936	-.0287369	-.3150599	.0393293
PRG600 vs AMT	-.2278765	.0572178	-.3400213	-.1157318	-.409969	-.0457841
DLX60 vs AMT	-.2383529	.0535243	-.3432586	-.1334472	-.4086911	-.0680147
DLX120 vs AMT	-.2472318	.0625811	-.3698885	-.1245751	-.4463928	-.0480708
MLN100 vs AMT	-.0989361	.0519128	-.2006832	.0028111	-.2641456	.0662735
MLN200 vs AMT	-.0703479	.0524074	-.1730644	.0323686	-.2371315	.0964357
PRG300 vs PRG150	-.1807803	.0212675	-.2224638	-.1390968	-.2484629	-.1130977
PRG450 vs PRG150	-.0926961	.0271125	-.1458356	-.0395566	-.1789802	-.0064121
PRG600 vs PRG150	-.1827074	.0300987	-.2416997	-.1237151	-.2784948	-.08692
DLX60 vs PRG150	-.1931838	.0226002	-.2374793	-.1488883	-.2651076	-.12126
DLX120 vs PRG150	-.2020627	.0395261	-.2795323	-.124593	-.3278523	-.076273
MLN100 vs PRG150	-.0537669	.0184621	-.0899521	-.0175818	-.1125217	.0049878
MLN200 vs PRG150	-.0251787	.0198103	-.0640062	.0136487	-.0882239	.0378664
PRG450 vs PRG300	.0880842	.0257683	.0375792	.1385892	.0060779	.1700905
PRG600 vs PRG300	-.001927	.0278716	-.0565543	.0527002	-.0906268	.0867727
DLX60 vs PRG300	-.0124034	.0256588	-.0626938	.037887	-.0940613	.0692544
DLX120 vs PRG300	-.0212823	.0413512	-.1023291	.0597645	-.1528802	.1103155
MLN100 vs PRG300	.1270134	.0221011	.083696	.1703308	.0566778	.197349
MLN200 vs PRG300	.1556016	.0232391	.1100538	.2011494	.0816445	.2295587
PRG600 vs PRG450	-.0900112	.0304635	-.1497186	-.0303039	-.1869597	.0069372
DLX60 vs PRG450	-.1004876	.0303655	-.160003	-.0409723	-.1971243	-.003851
DLX120 vs PRG450	-.1093665	.0444253	-.1964386	-.0222945	-.2507477	.0320147
MLN100 vs PRG450	.0389292	.0274253	-.0148235	.0926819	-.0483504	.1262088
MLN200 vs PRG450	.0675174	.0283504	.0119516	.1230831	-.0227062	.157741
DLX60 vs PRG600	-.0104764	.0331031	-.0753573	.0544045	-.1158252	.0948724
DLX120 vs PRG600	-.0193553	.0463396	-.1101792	.0714687	-.1668286	.128118
MLN100 vs PRG600	.1289404	.0304286	.0693015	.1885794	.0321031	.2257777
MLN200 vs PRG600	.1575286	.0312649	.0962506	.2188067	.0580298	.2570275
DLX120 vs DLX60	-.0088789	.0386767	-.0846839	.066926	-.1319654	.1142076
MLN100 vs DLX60	.1394168	.0227349	.0948572	.1839765	.0670641	.2117696
MLN200 vs DLX60	.168005	.0238427	.1212742	.2147358	.092127	.2438831
MLN100 vs DLX120	.1482957	.0396033	.0706747	.2259168	.0222604	.2743311
MLN200 vs DLX120	.1768839	.0402494	.0979965	.2557714	.0487923	.3049756
MLN200 vs MLN100	.0285882	.017702	-.0061072	.0632836	-.0277476	.084924

PLB: **Placebo;** AMT: **Amitriptyline;** PRG150: **Pregabalin 150mg;** PRG300: **Pregabalin 300mg;** PRG450: **Pregabalin 450mg;** PRG600: **Pregabalin 600mg;** DLX60: **Duloxetine 60mg;** DLX120: **Duloxetine 120mg;** MLN100: **Milnacipran 100mg;** MLN200: **Milnacipran 200mg.**

© 2022 Farag HM et al. *JAMA Network Open.*

eFigure 4D. Depression outcome: SUCRA plots

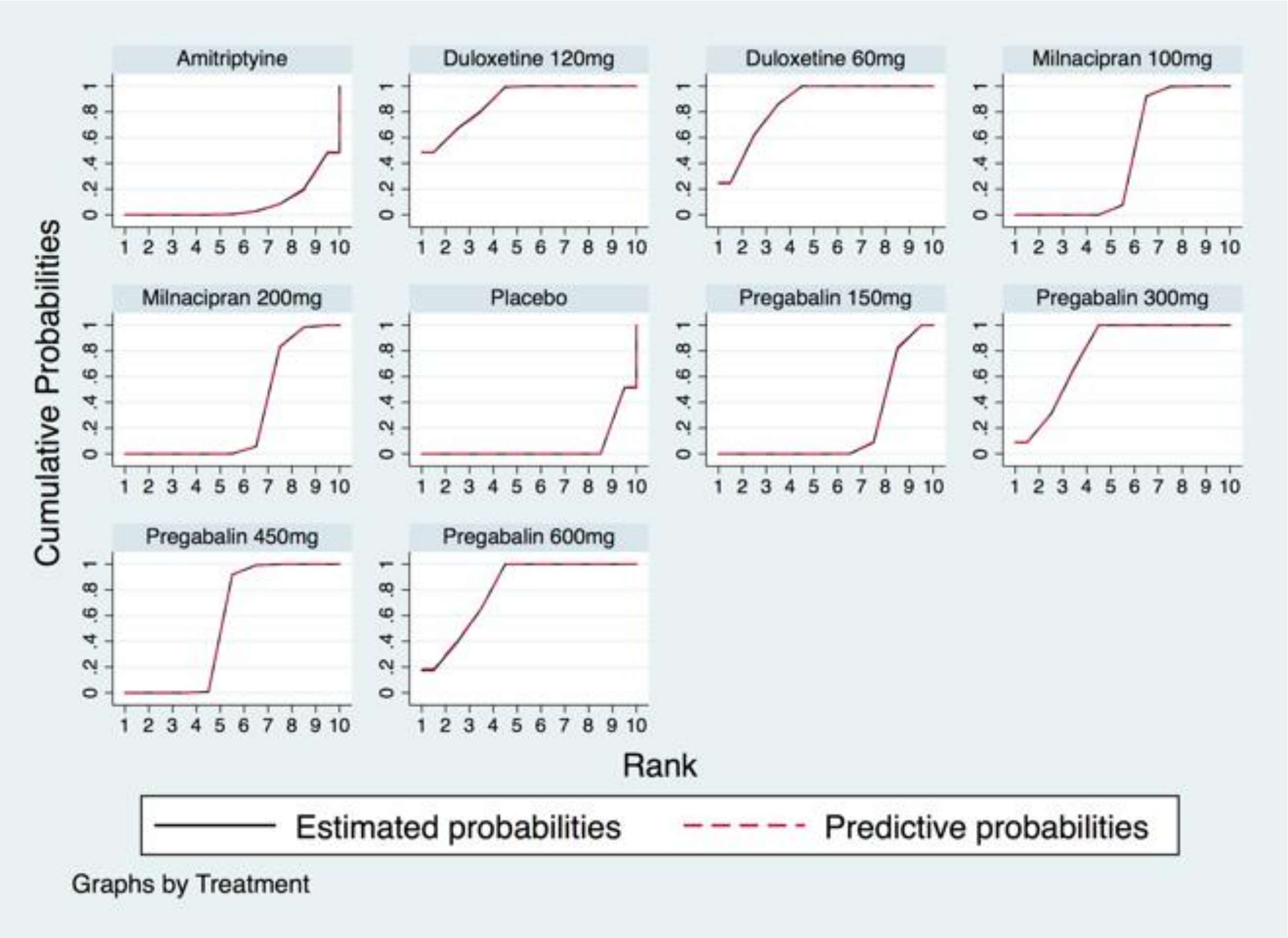

Cumulative probability curves for the fibromyalgia treatments network showing the estimated and predictive probabilities for each treatment being up to a specific rank.

eFigure 4D. Depression outcome: SUCRA plots (continued)

© 2022 Farag HM et al. *JAMA Network Open.*

Treatment	SUCRA	PrBest	MeanRank
Placebo	5.7	0.0	9.5
Amitriptyine	8.9	0.0	9.2
Pregabalin 150mg	21.1	0.0	8.1
Pregabalin 300mg	78.6	9.0	2.9
Pregabalin 450mg	54.6	0.0	5.1
Pregabalin 600mg	80.3	17.4	2.8
Duloxetine 60mg	85.9	24.9	2.3
Duloxetine 120mg	88.4	48.7	2.0
Milnacipran 100mg	44.4	0.0	6.0
Milnacipran 200mg	31.9	0.0	7.1

© 2022 Farag HM et al. *JAMA Network Open.*

eFigure 4E. Depression outcome: rankogram plots

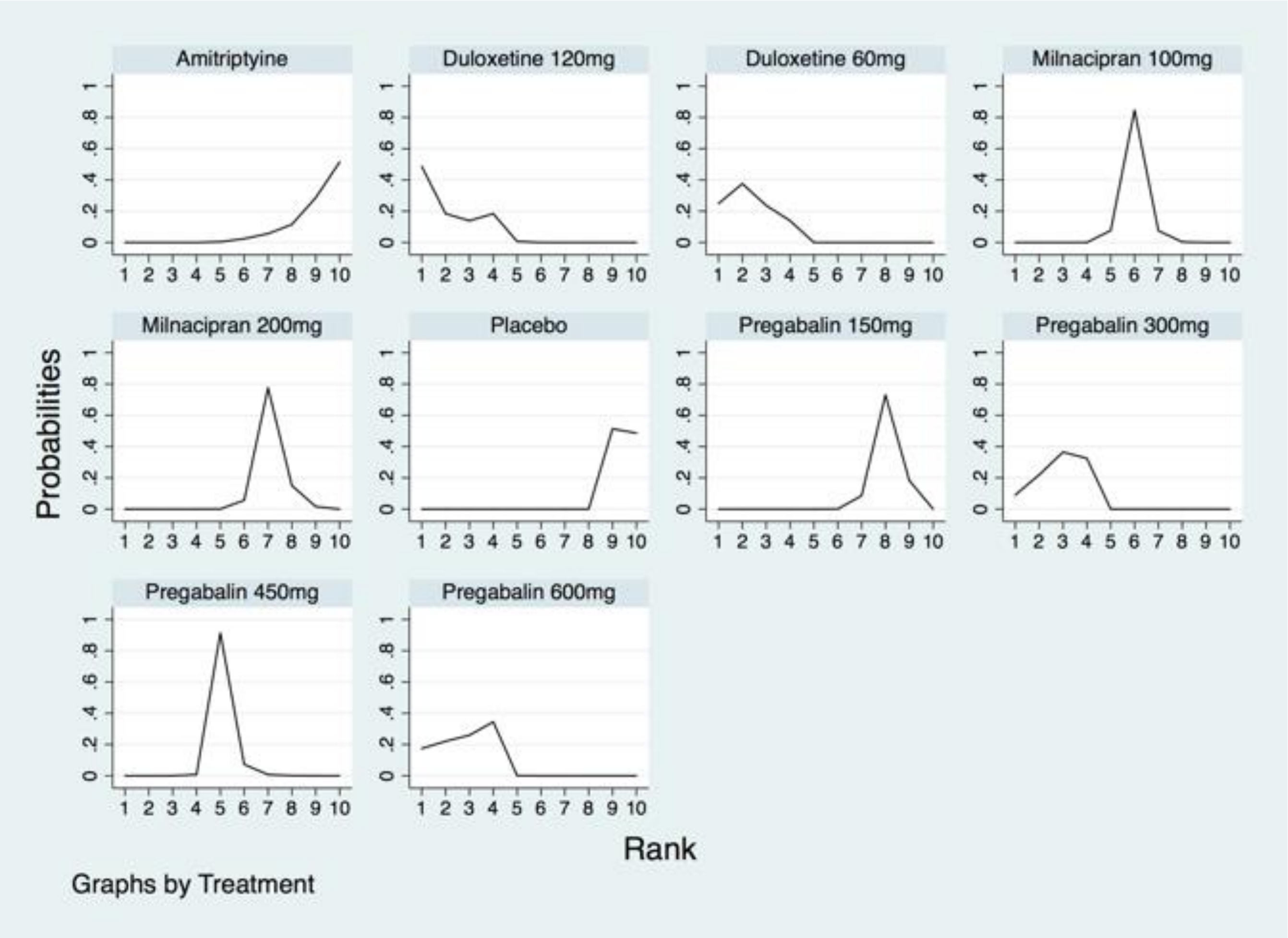

Rankogram plots for the fibromyalgia treatments network showing the probability for every treatment being at particular order.

eFigure 4E. Depression outcome: rankogram plots (continued)

© 2022 Farag HM et al. *JAMA Network Open.*

id and Rank	Treatment									
	1	2	3	4	5	6	7	8	9	10
1										
Best	**0.0**	**0.0**	**0.0**	**9.0**	**0.0**	**17.4**	**24.9**	**48.7**	**0.0**	**0.0**
2nd	**0.0**	**0.0**	**0.0**	**22.0**	**0.0**	**22.1**	**37.6**	**18.4**	**0.0**	**0.0**
3rd	**0.0**	**0.0**	**0.0**	**36.5**	**0.0**	**25.9**	**23.7**	**13.9**	**0.0**	**0.0**
4th	**0.0**	**0.0**	**0.0**	**32.5**	**0.7**	**34.5**	**13.9**	**18.4**	**0.0**	**0.0**
5th	**0.0**	**0.4**	**0.0**	**0.0**	**91.2**	**0.1**	**0.0**	**0.6**	**7.6**	**0.1**
6th	**0.0**	**2.4**	**0.1**	**0.0**	**7.3**	**0.0**	**0.0**	**0.0**	**84.6**	**5.7**
7th	**0.0**	**5.6**	**8.6**	**0.0**	**0.7**	**0.0**	**0.0**	**0.0**	**7.5**	**77.6**
8th	**0.0**	**11.5**	**73.0**	**0.0**	**0.1**	**0.0**	**0.0**	**0.0**	**0.3**	**15.1**
9th	**51.4**	**28.7**	**18.3**	**0.0**	**0.0**	**0.0**	**0.0**	**0.0**	**0.0**	**1.6**
Worst	**48.6**	**51.4**	**0.0**	**0.0**	**0.0**	**0.0**	**0.0**	**0.0**	**0.0**	**0.0**

1: **Placebo** 2: **Amitriptyline** 3: **Pregabalin 150mg** 4: **Pregabalin 300mg** 5: **Pregabalin 450mg** 6: **Pregabalin 600mg** 7: **Duloxetine 60mg** 8: **Duloxetine 120mg** 9: **Milnacipran 100mg** 10: **Milnacipran 200mg.**

© 2022 Farag HM et al. *JAMA Network Open.*

eFigure 4F. Depression outcome: comparison adjusted funnel plot

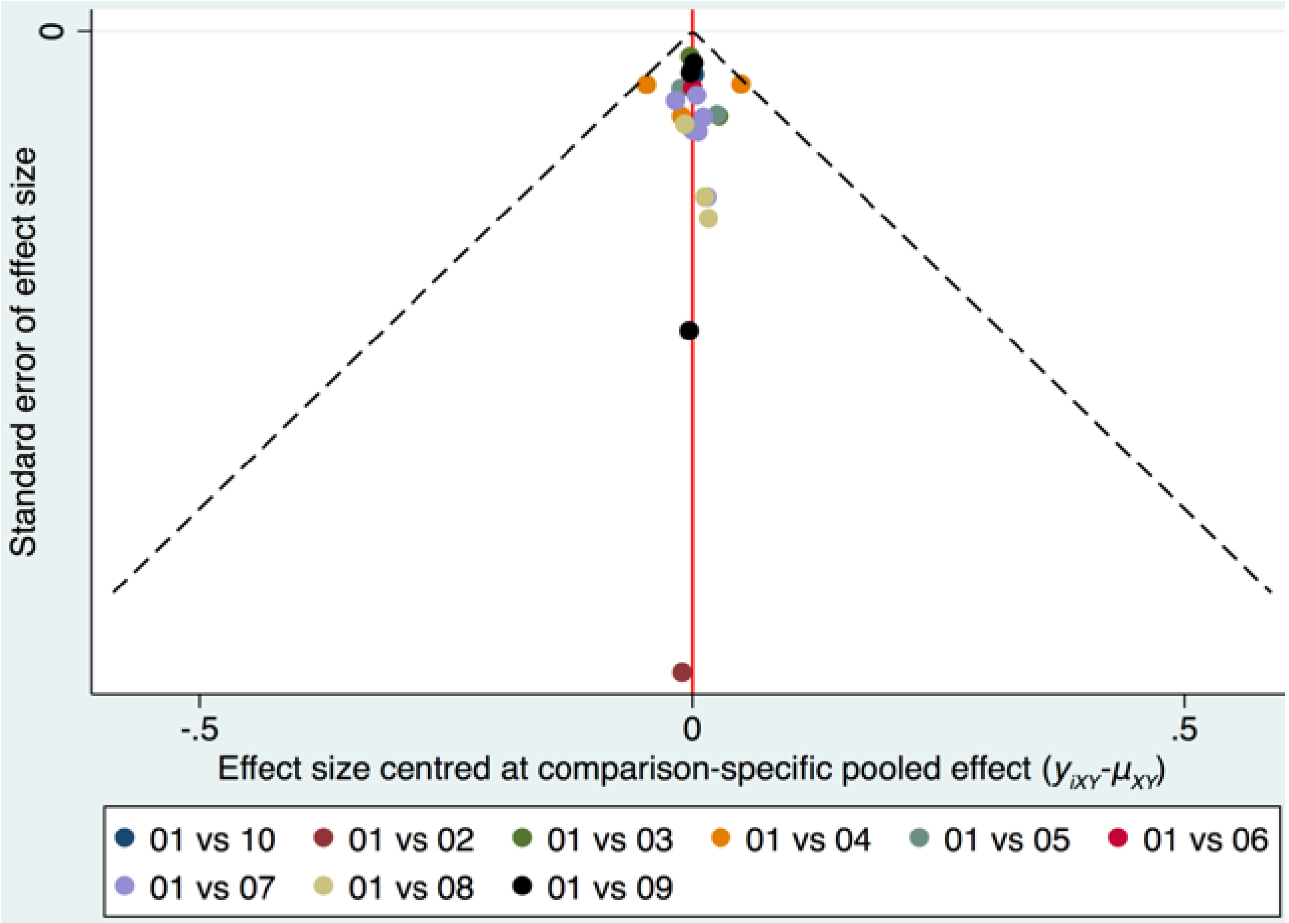

The red line represents the null hypothesis that the study-specific effect sizes do not differ from the respective comparison-specific pooled effect estimates. Different colors correspond to different comparisons. 1: **Placebo** 2: **Amitriptyline** 3: **Pregabalin 150mg** 4: **Pregabalin 300mg** 5: **Pregabalin 450mg** 6: **Pregabalin 600mg** 7: **Duloxetine 60mg** 8: **Duloxetine 120mg** 9: **Milnacipran 100mg** 10: **Milnacipran 200mg.**

© 2022 Farag HM et al. *JAMA Network Open.*

eFigure 4G. Depression outcome: network pattern

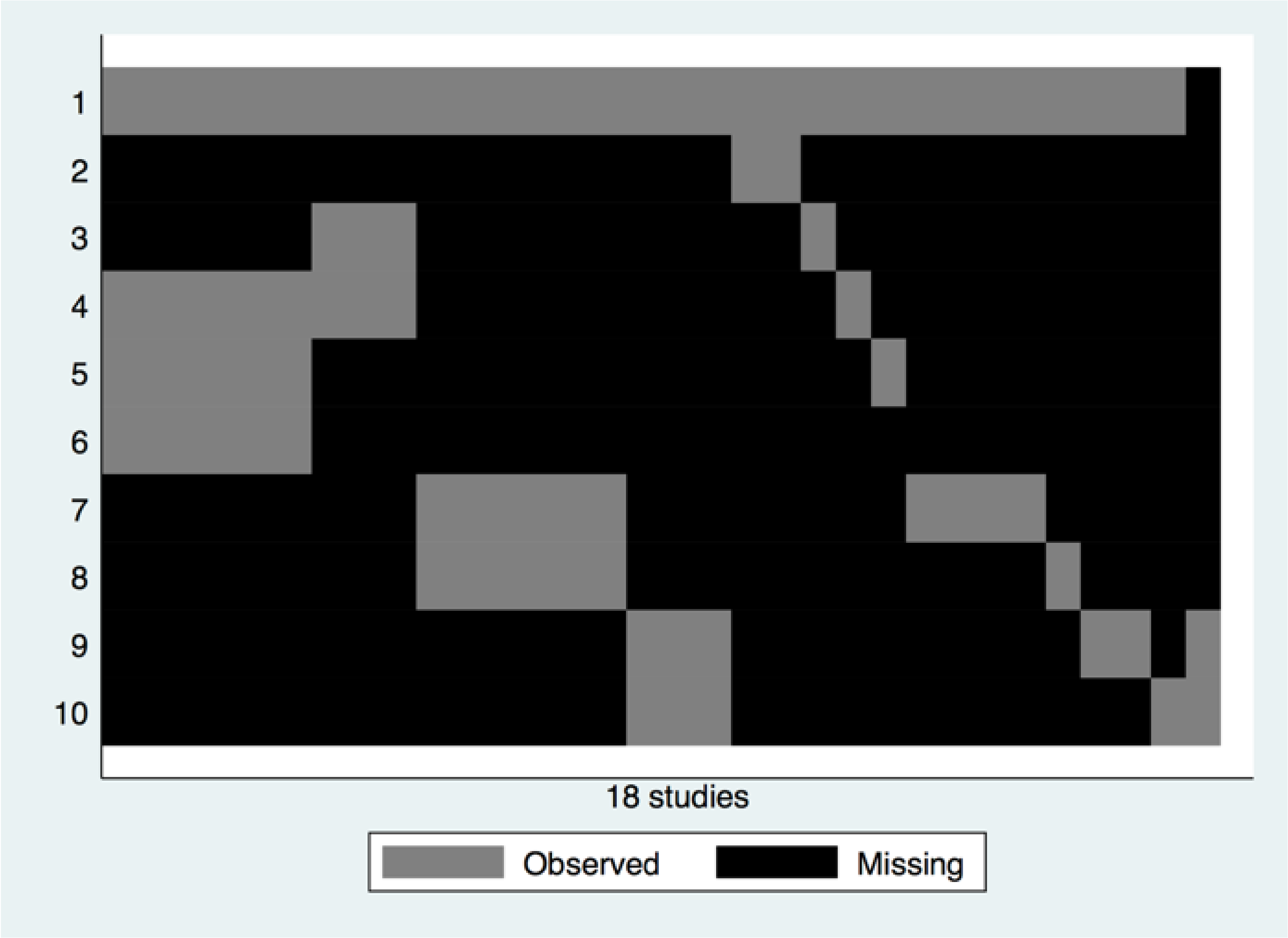

eFigure 5. Fatigue Outcome: Results

© 2022 Farag HM et al. *JAMA Network Open.*

eFigure 5A. Fatigue outcome: network forest

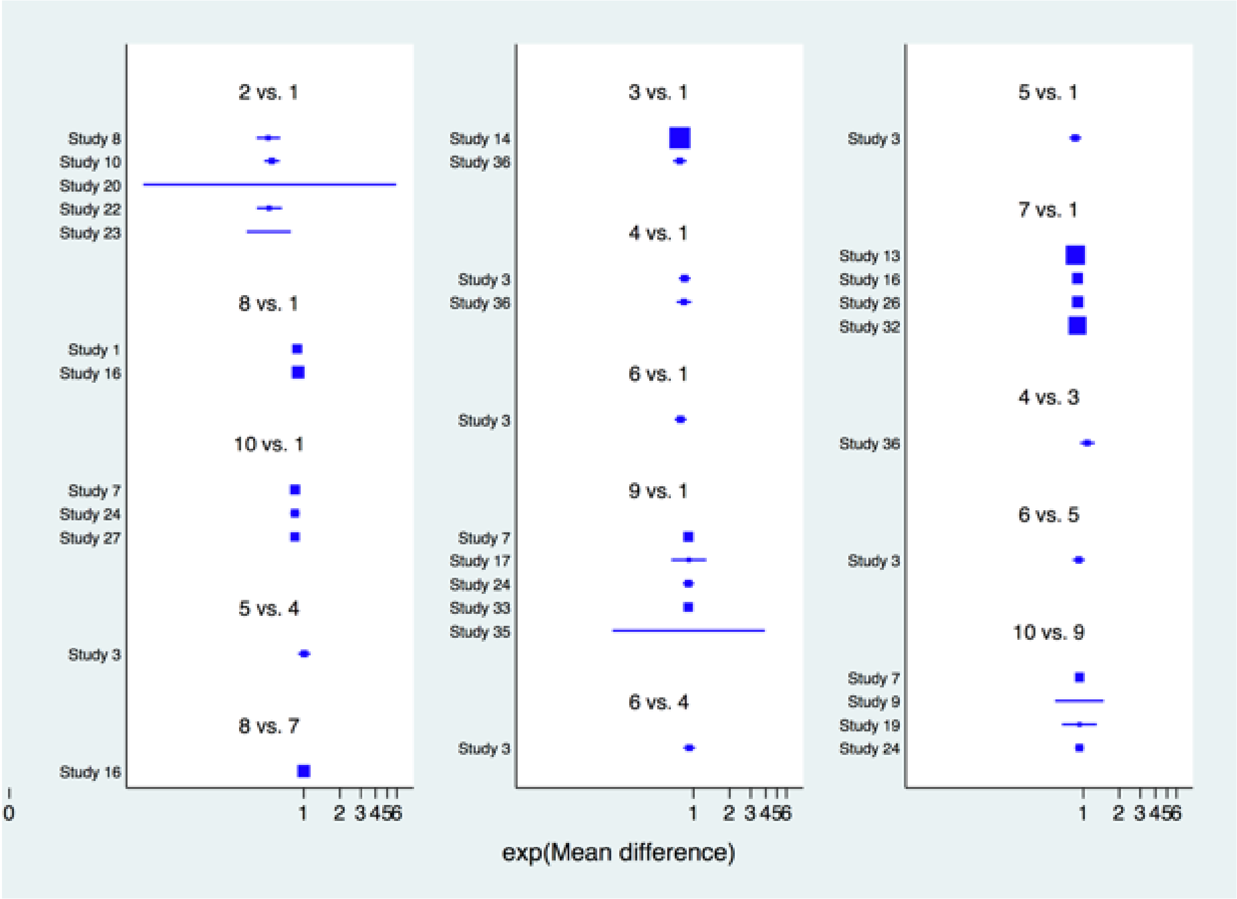

1: **Placebo** 2: **Amitriptyline** 3: **Pregabalin 150mg** 4: **Pregabalin 300mg** 5: **Pregabalin 450mg** 6: **Pregabalin 600mg** 7: **Duloxetine 60mg** 8: **Duloxetine 120mg** 9: **Milnacipran 100mg** 10: **Milnacipran 200mg.**

© 2022 Farag HM et al. *JAMA Network Open.*

eFigure 5B. Fatigue outcome: contribution plot

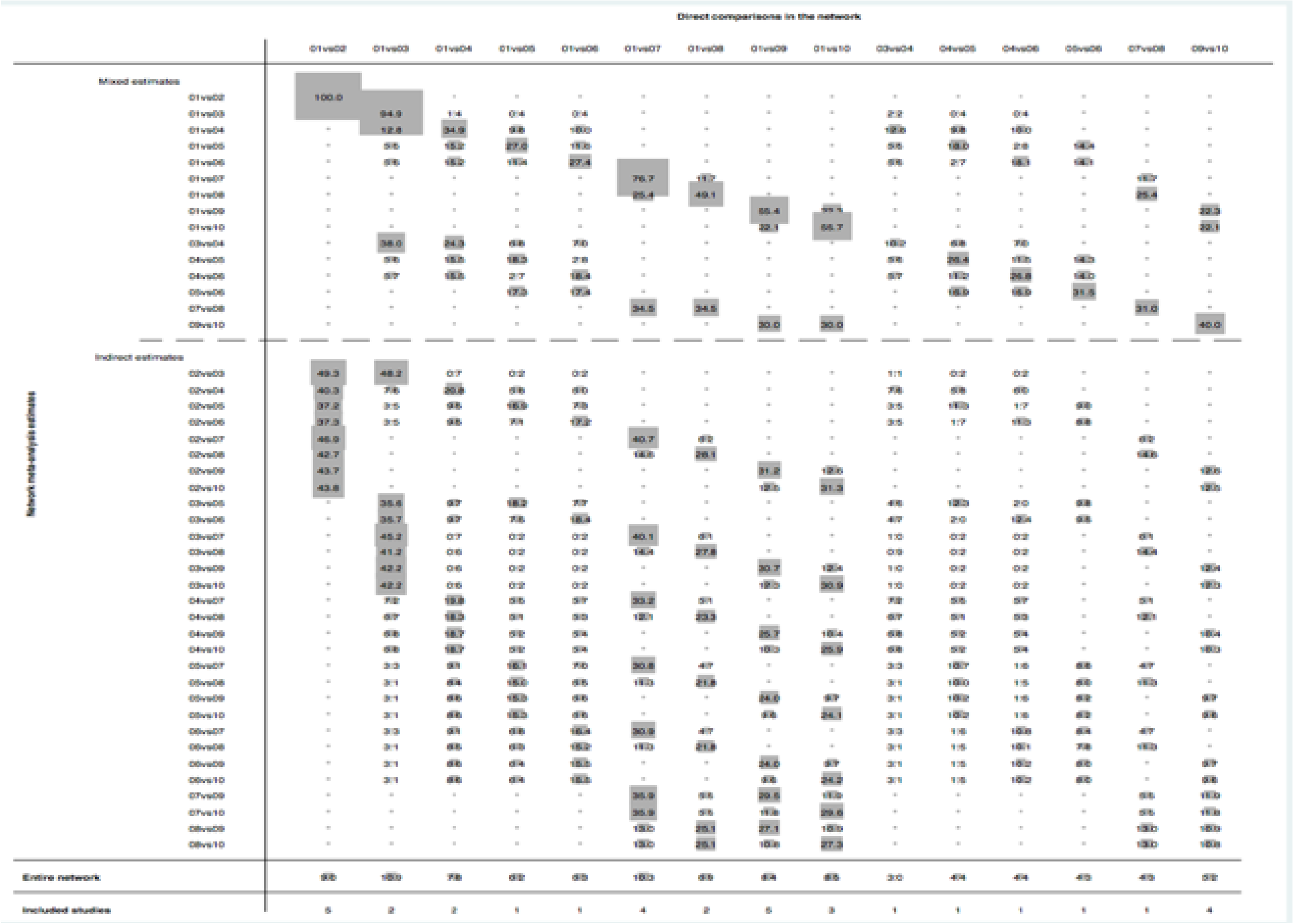

The size of each square is proportional to the weight attached to each direct summary effect (horizontal axis) for the estimation of each network summary effects (vertical axis). The numbers re-express the weights as percentages. 1: **Placebo** 2: **Amitriptyline** 3: **Pregabalin 150mg** 4: **Pregabalin 300mg** 5: **Pregabalin 450mg** 6: **Pregabalin 600mg** 7: **Duloxetine 60mg** 8: **Duloxetine 120mg** 9: **Milnacipran 100mg** 10: **Milnacipran 200mg.**

© 2022 Farag HM et al. *JAMA Network Open.*

eFigure 5C. Fatigue outcome: interval plot (estimates as Standardized Mean Difference)

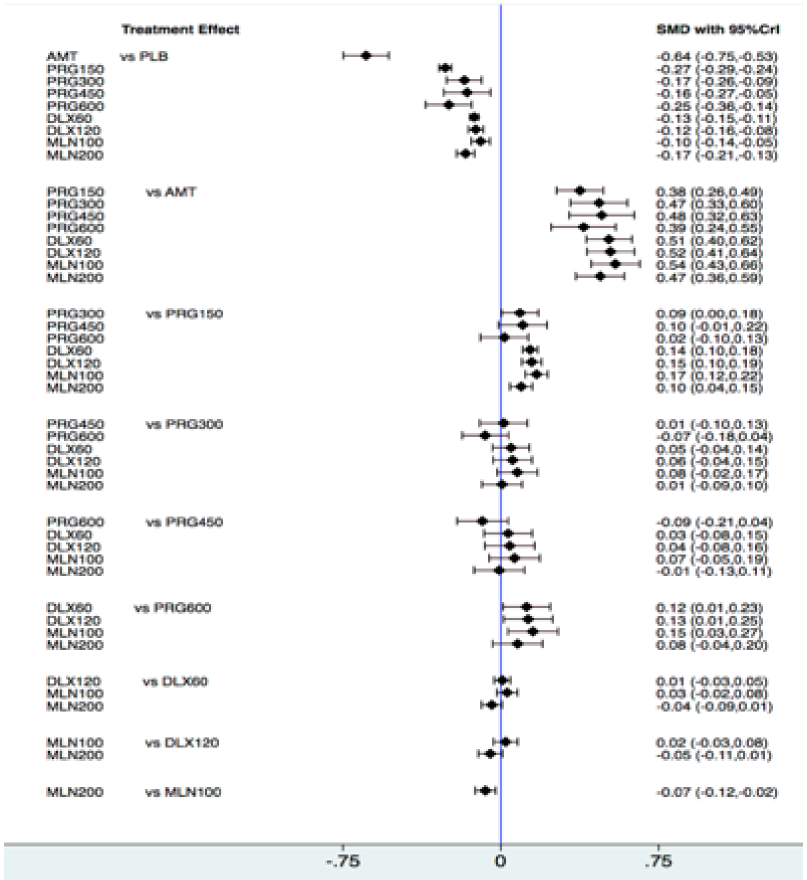

The black solid lines represent the credible intervals for summary standardized mean differences (SMD) for each comparison, and the blue line is the line of no effect (SMD equal to 0). CrI indicates credible interval; SMD, standardized mean difference. PLB: **Placebo;** AMT: **Amitriptyline;** PRG150: **Pregabalin 150mg;** PRG300: **Pregabalin 300mg;** PRG450: **Pregabalin 450mg;** PRG600: **Pregabalin 600mg;** DLX60: **Duloxetine 60mg;** DLX120: **Duloxetine 120mg;** MLN100: **Milnacipran 100mg;** MLN200: **Milnacipran 200mg.**

eFigure 5C. Fatigue outcome: interval plot (continued)

© 2022 Farag HM et al. *JAMA Network Open.*

_Comparison	_Effect_Size	_Standard_Error	_LCI	_UCI
AMT vs PLB	−.6402875	.0547952	−.7476841	−.5328908
PRG150 vs PLB	−.265098	.015115	−.2947228	−.2354732
PRG300 vs PLB	−.1742744	.0430284	−.2586086	−.0899403
PRG450 vs PLB	−.1609218	.0569182	−.2724794	−.0493642
PRG600 vs PLB	−.2480018	.0560906	−.3579373	−.1380662
DLX60 vs PLB	−.1261049	.0106811	−.1470394	−.1051704
DLX120 vs PLB	−.1186845	.0187063	−.1553483	−.0820207
MLN100 vs PLB	−.0957179	.0224398	−.1396991	−.0517367
MLN200 vs PLB	−.1684015	.0220443	−.2116075	−.1251955
PRG150 vs AMT	.3751894	.0568442	.2637768	.486602
PRG300 vs AMT	.466013	.0696703	.3294617	.6025643
PRG450 vs AMT	.4793656	.0790075	.3245137	.6342176
PRG600 vs AMT	.3922856	.0784134	.2385982	.5459731
DLX60 vs AMT	.5141825	.055844	.4047303	.6236346
DLX120 vs AMT	.5216029	.0579039	.4081135	.6350924
MLN100 vs AMT	.5445696	.0592121	.4285159	.6606232
MLN200 vs AMT	.4718859	.0590631	.3561244	.5876476
PRG300 vs PRG150	.0908236	.044511	.0035837	.1780635
PRG450 vs PRG150	.1041762	.0584821	−.0104465	.2187989
PRG600 vs PRG150	.0170962	.0576769	−.0959484	.1301408
DLX60 vs PRG150	.1389931	.0184494	.1028329	.1751532
DLX120 vs PRG150	.1464135	.0240402	.0992957	.1935313
MLN100 vs PRG150	.1693801	.0270553	.1163528	.2224075
MLN200 vs PRG150	.0966965	.0267288	.0443091	.1490839
PRG450 vs PRG300	.0133527	.0573592	−.0990693	.1257746
PRG600 vs PRG300	−.0737273	.0565381	−.1845399	.0370852
DLX60 vs PRG300	.0481695	.044335	−.0387255	.1350645
DLX120 vs PRG300	.0555899	.0469189	−.0363695	.1475493
MLN100 vs PRG300	.0785566	.0485282	−.016557	.1736701
MLN200 vs PRG300	.005873	.0483466	−.0888847	.1006306
PRG600 vs PRG450	−.08708	.0622879	−.209162	.035002
DLX60 vs PRG450	.0348169	.0579122	−.078689	.1483228
DLX120 vs PRG450	.0422373	.0599135	−.0751909	.1596655
MLN100 vs PRG450	.0652039	.0611819	−.0547104	.1851183
MLN200 vs PRG450	−.0074797	.061038	−.1271119	.1121525
DLX60 vs PRG600	.1218969	.057099	.0099848	.2338089
DLX120 vs PRG600	.1293173	.0591278	.013429	.2452056
MLN100 vs PRG600	.1522839	.0604127	.0338771	.2706907
MLN200 vs PRG600	.0796003	.0602669	−.0385207	.1977213
DLX120 vs DLX60	.0074204	.0195967	−.0309884	.0458292
MLN100 vs DLX60	.030387	.0248497	−.0183174	.0790915
MLN200 vs DLX60	−.0422966	.0244977	−.0903113	.0057181
MLN100 vs DLX120	.0229666	.0292138	−.0342913	.0802246
MLN200 vs DLX120	−.049717	.0289119	−.1063833	.0069493
MLN200 vs MLN100	−.0726836	.0249431	−.1215712	−.023796

PLB: **Placebo;** AMT: **Amitriptyline;** PRG150: **Pregabalin 150mg;** PRG300: **Pregabalin 300mg;** PRG450: **Pregabalin 450mg;** PRG600: **Pregabalin 600mg;** DLX60: **Duloxetine 60mg;** DLX120: **Duloxetine 120mg;** MLN100: **Milnacipran 100mg;** MLN200: **Milnacipran 200mg.**

© 2022 Farag HM et al. *JAMA Network Open.*

eFigure 5D. Fatigue outcome: SUCRA plots

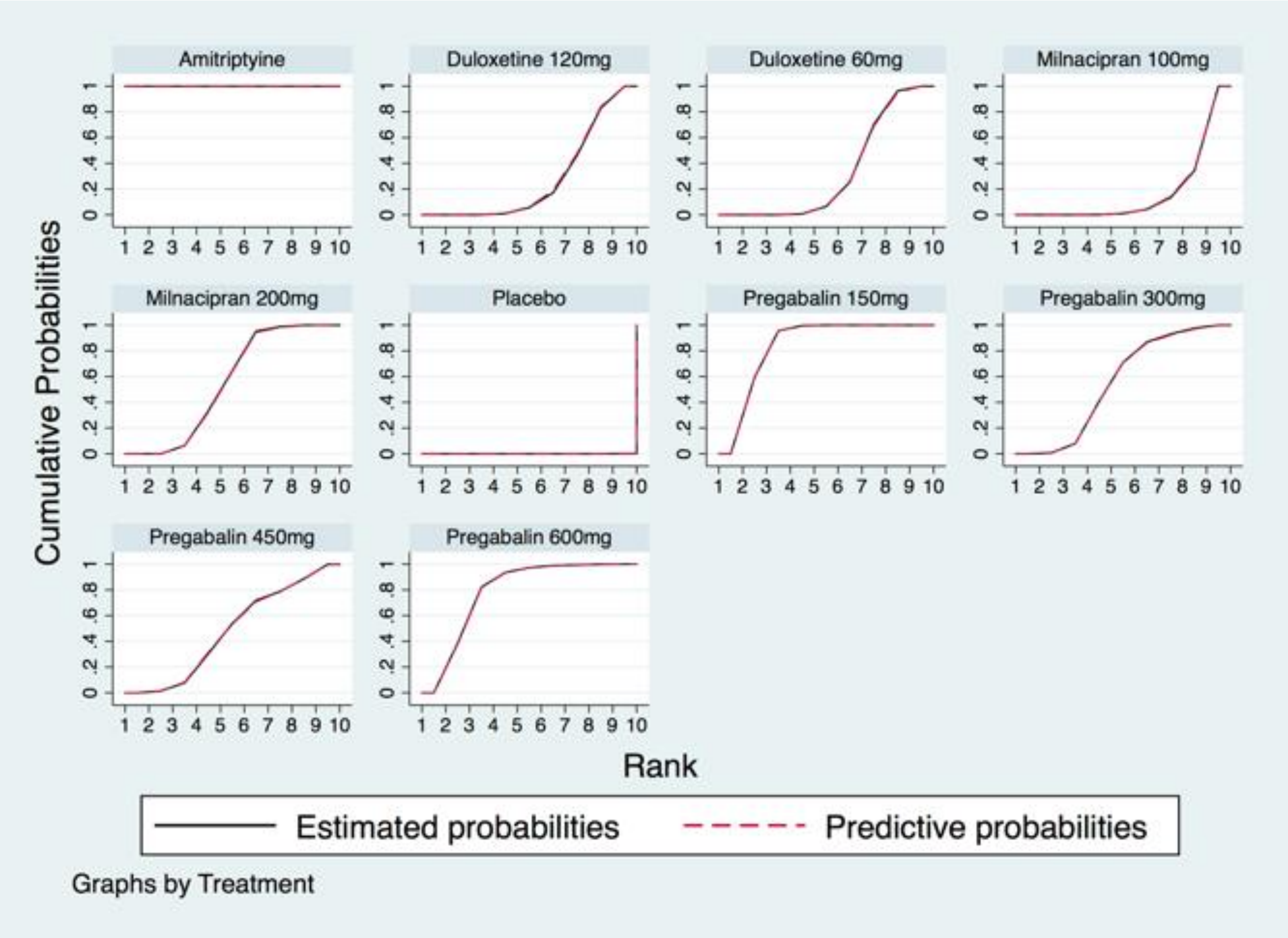

Cumulative probability curves for the fibromyalgia treatments network showing the estimated and predictive probabilities for each treatment being up to a specific rank.

eFigure 5D. Fatigue outcome: SUCRA plots (continued)

© 2022 Farag HM et al. *JAMA Network Open.*

Treatment	SUCRA	PrBest	MeanRank
Placebo	0.0	0.0	10.0
Amitriptyine	100.0	100.0	1.0
Pregabalin 150mg	83.8	0.0	2.5
Pregabalin 300mg	55.4	0.0	5.0
Pregabalin 450mg	48.1	0.0	5.7
Pregabalin 600mg	78.9	0.0	2.9
Duloxetine 60mg	33.3	0.0	7.0
Duloxetine 120mg	28.1	0.0	7.5
Milnacipran 100mg	17.1	0.0	8.5
Milnacipran 200mg	55.4	0.0	5.0

© 2022 Farag HM et al. *JAMA Network Open.*

eFigure 5E. Fatigue outcome: rankogram plots

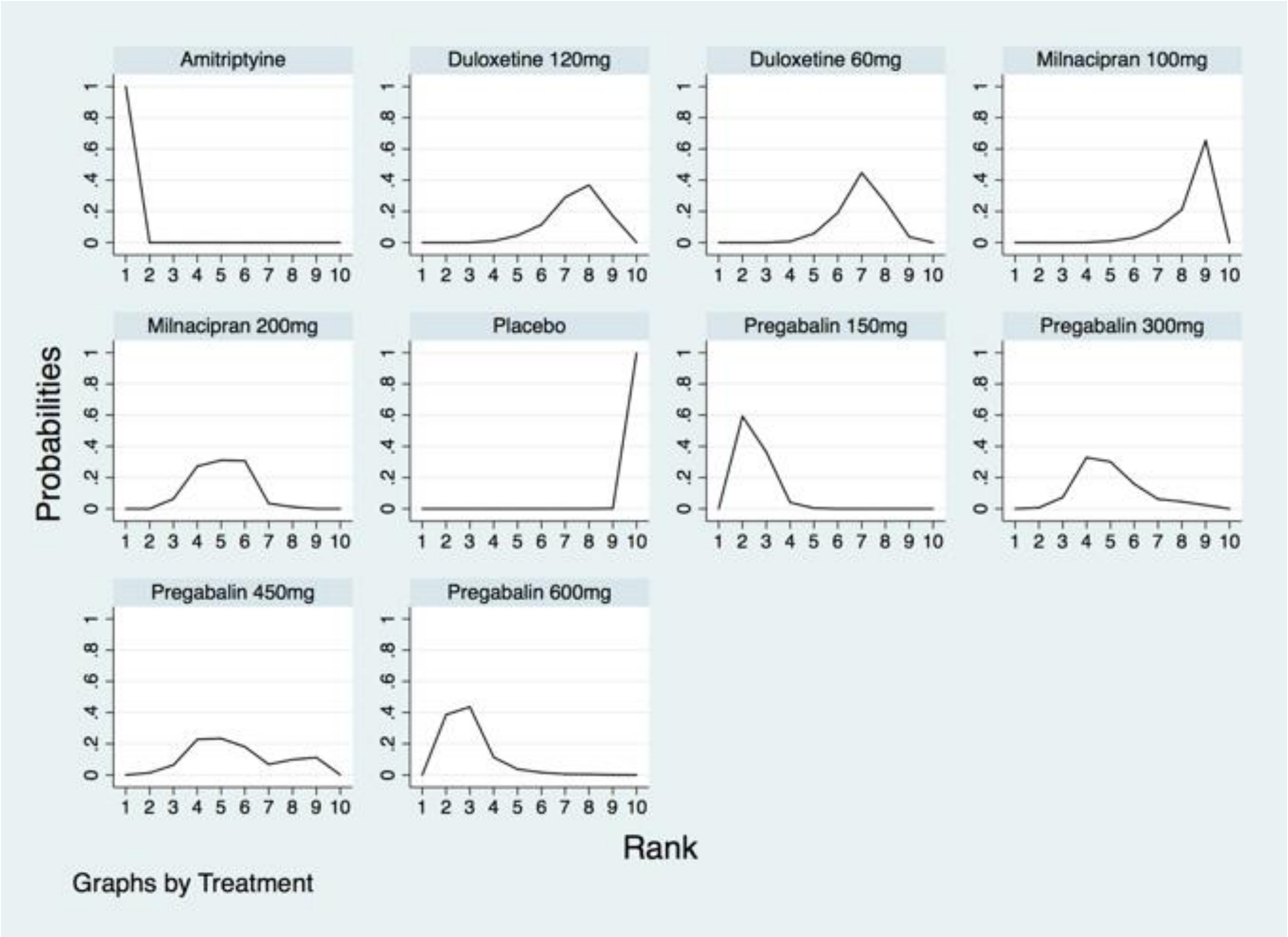

Rankogram plots for the fibromyalgia treatments network showing the probability for every treatment being at particular order.

eFigure 5E. Fatigue outcome: rankogram plots (continued)

© 2022 Farag HM et al. *JAMA Network Open.*

id and Rank	Treatment 1	2	3	4	5	6	7	8	9	10
1										
Best	**0.0**	**100.0**	**0.0**	**0.0**	**0.0**	**0.0**	**0.0**	**0.0**	**0.0**	**0.0**
2nd	**0.0**	**0.0**	**59.3**	**0.7**	**1.4**	**38.6**	**0.0**	**0.0**	**0.0**	**0.0**
3rd	**0.0**	**0.0**	**36.3**	**7.3**	**6.3**	**43.6**	**0.0**	**0.1**	**0.0**	**6.3**
4th	**0.0**	**0.0**	**4.0**	**32.8**	**22.8**	**11.3**	**0.7**	**1.0**	**0.1**	**27.2**
5th	**0.0**	**0.0**	**0.4**	**30.1**	**23.4**	**3.7**	**5.8**	**4.6**	**0.9**	**31.1**
6th	**0.0**	**0.0**	**0.0**	**15.9**	**18.0**	**1.6**	**19.1**	**11.4**	**3.2**	**30.7**
7th	**0.0**	**0.0**	**0.0**	**6.2**	**6.8**	**0.6**	**44.7**	**29.0**	**9.3**	**3.4**
8th	**0.0**	**0.0**	**0.0**	**4.6**	**9.9**	**0.4**	**26.0**	**36.8**	**21.1**	**1.2**
9th	**0.2**	**0.0**	**0.0**	**2.3**	**11.2**	**0.1**	**3.7**	**17.1**	**65.4**	**0.0**
Worst	**99.8**	**0.0**	**0.0**	**0.0**	**0.2**	**0.0**	**0.0**	**0.0**	**0.0**	**0.0**

1: **Placebo** 2: **Amitriptyline** 3: **Pregabalin 150mg** 4: **Pregabalin 300mg** 5: **Pregabalin 450mg** 6: **Pregabalin 600mg** 7: **Duloxetine 60mg** 8: **Duloxetine 120mg** 9: **Milnacipran 100mg** 10: **Milnacipran 200mg.**

© 2022 Farag HM et al. *JAMA Network Open.*

eFigure 5F. Fatigue outcome: comparison adjusted funnel plot

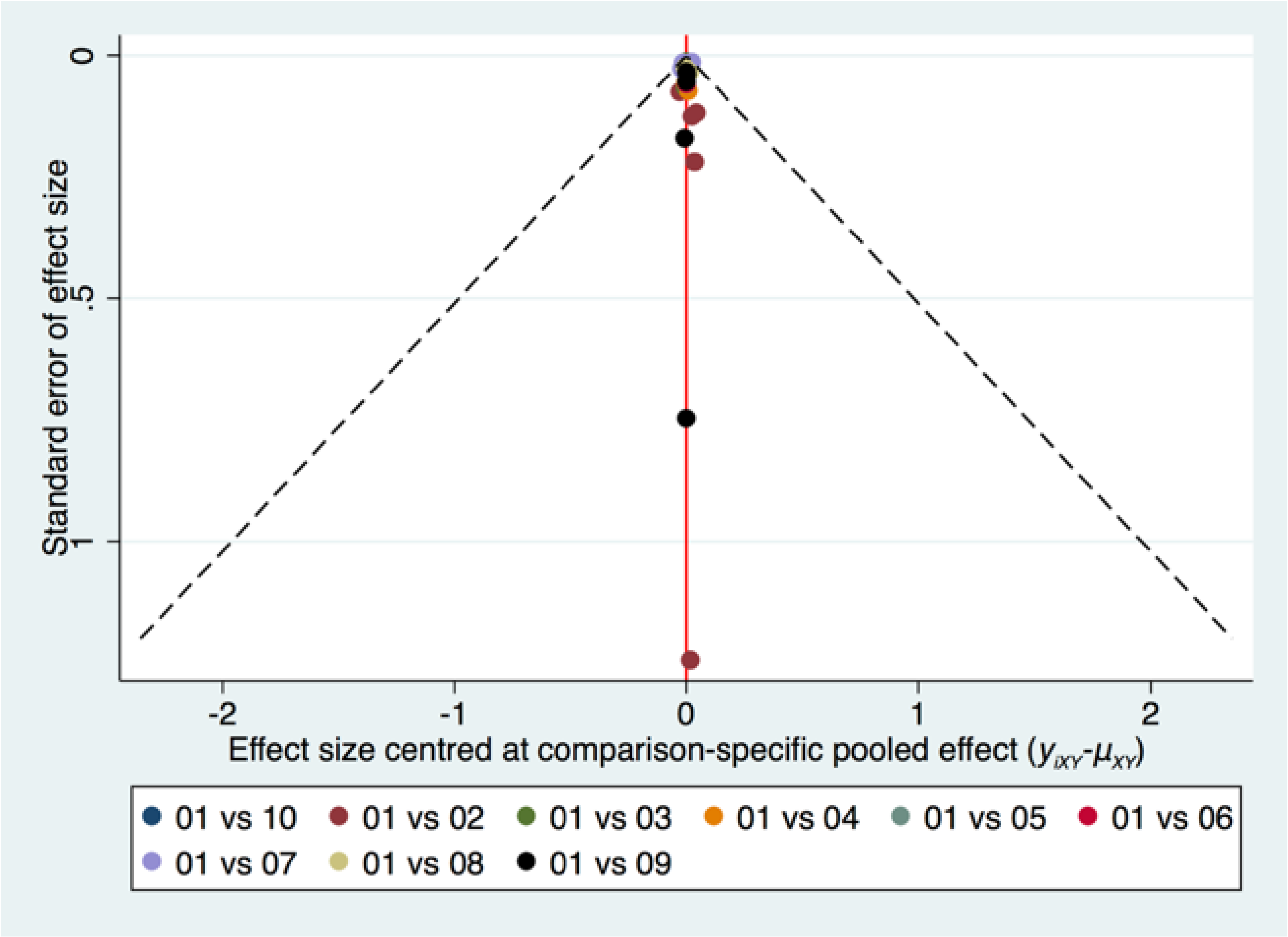

The red line represents the null hypothesis that the study-specific effect sizes do not differ from the respective comparison-specific pooled effect estimates. Different colors correspond to different comparisons. 1: **Placebo** 2: **Amitriptyline** 3: **Pregabalin 150mg** 4: **Pregabalin 300mg** 5: **Pregabalin 450mg** 6: **Pregabalin 600mg** 7: **Duloxetine 60mg** 8: **Duloxetine 120mg** 9: **Milnacipran 100mg** 10: **Milnacipran 200mg.**

© 2022 Farag HM et al. *JAMA Network Open.*

eFigure 5G. Fatigue outcome: network pattern

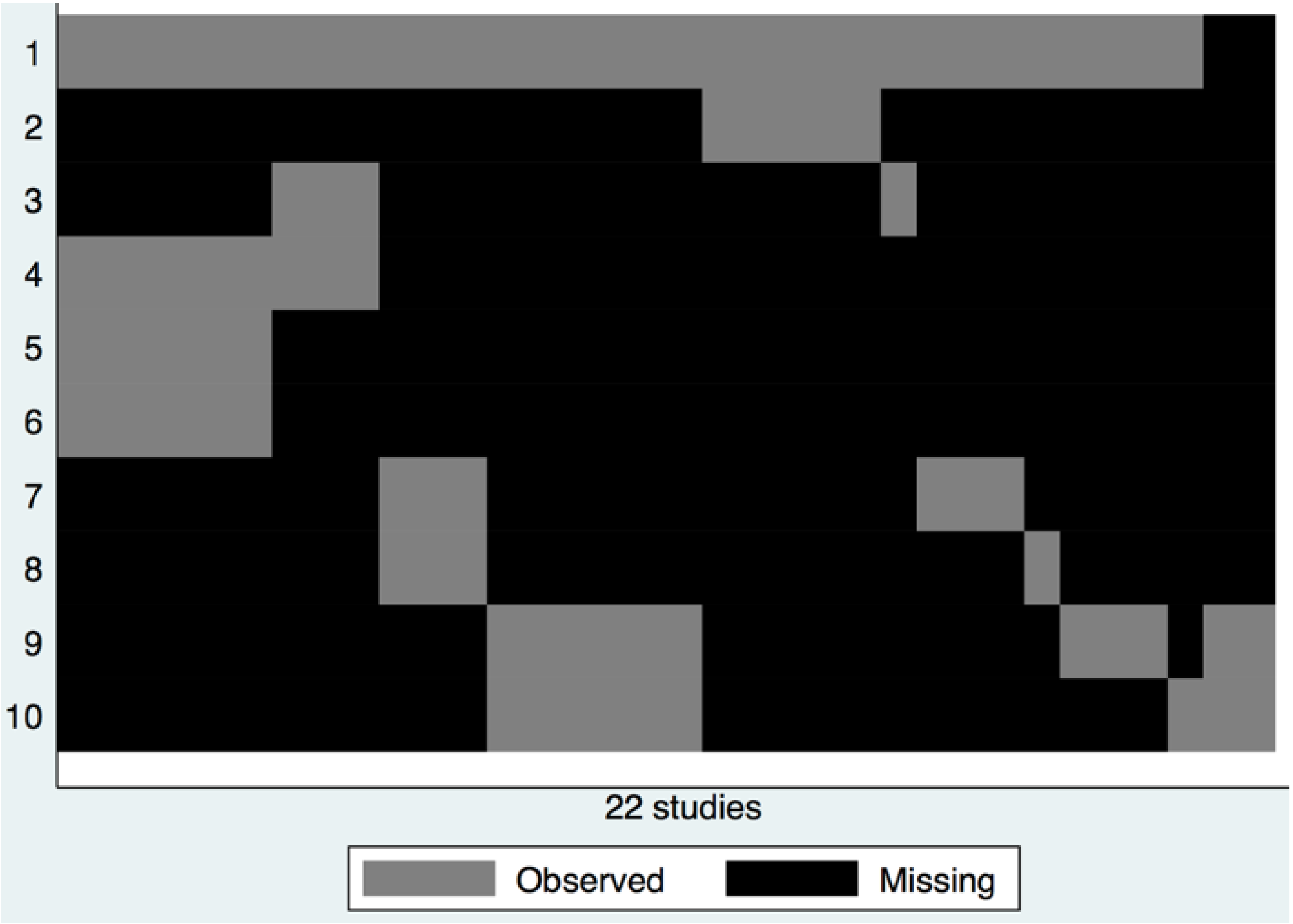

eFigure 6. Quality of Life Outcome: Results

© 2022 Farag HM et al. *JAMA Network Open.*

eFigure 6A. Quality of life outcome: network forest

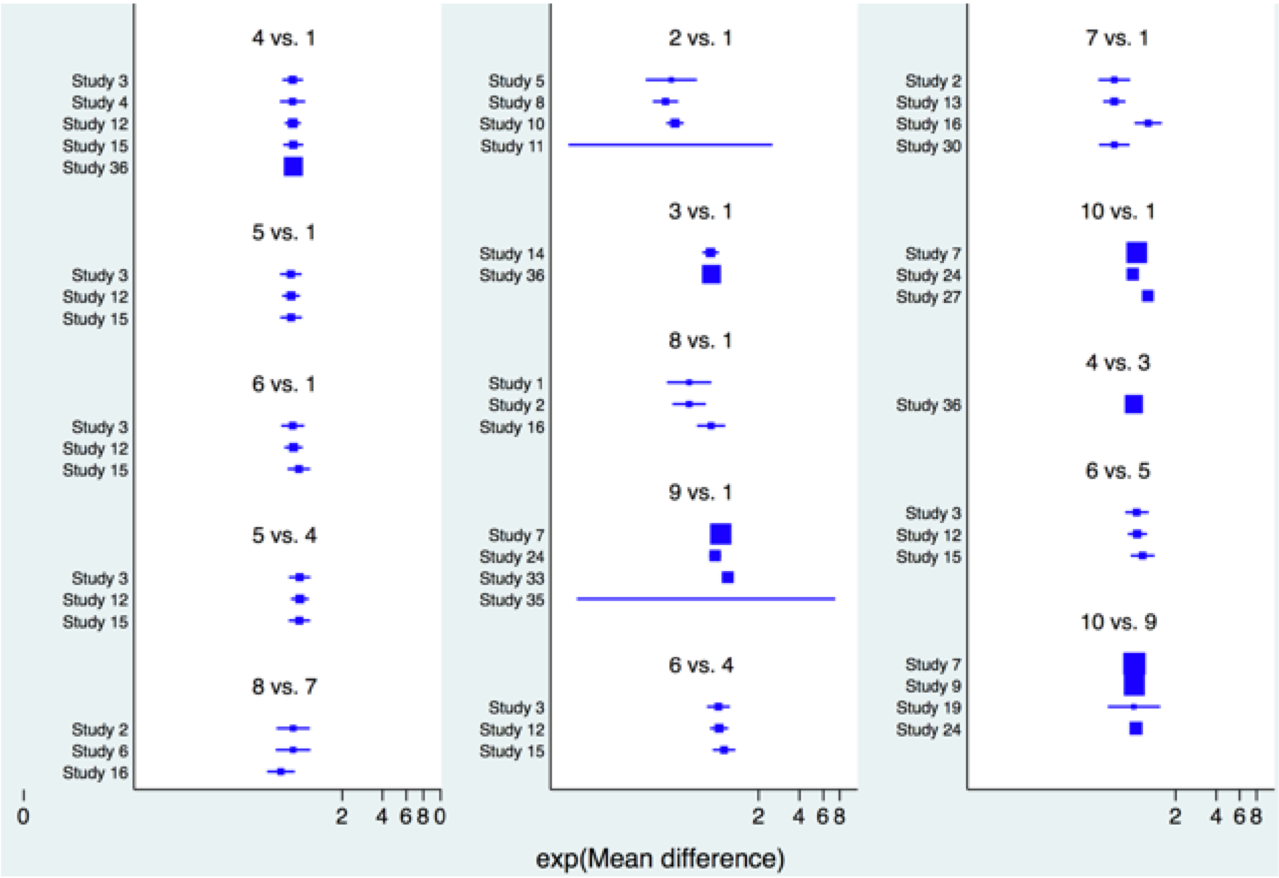

<u>1:</u> **Placebo** <u>2:</u> **Amitriptyline** <u>3:</u> **Pregabalin 150mg** <u>4:</u> **Pregabalin 300mg** <u>5:</u> **Pregabalin 450mg** <u>6:</u> **Pregabalin 600mg** <u>7:</u> **Duloxetine 60mg** <u>8:</u> **Duloxetine 120mg** <u>9:</u> **Milnacipran 100mg** <u>10:</u> **Milnacipran 200mg.**

© 2022 Farag HM et al. *JAMA Network Open.*

eFigure 6B. Quality of life outcome: contribution plot

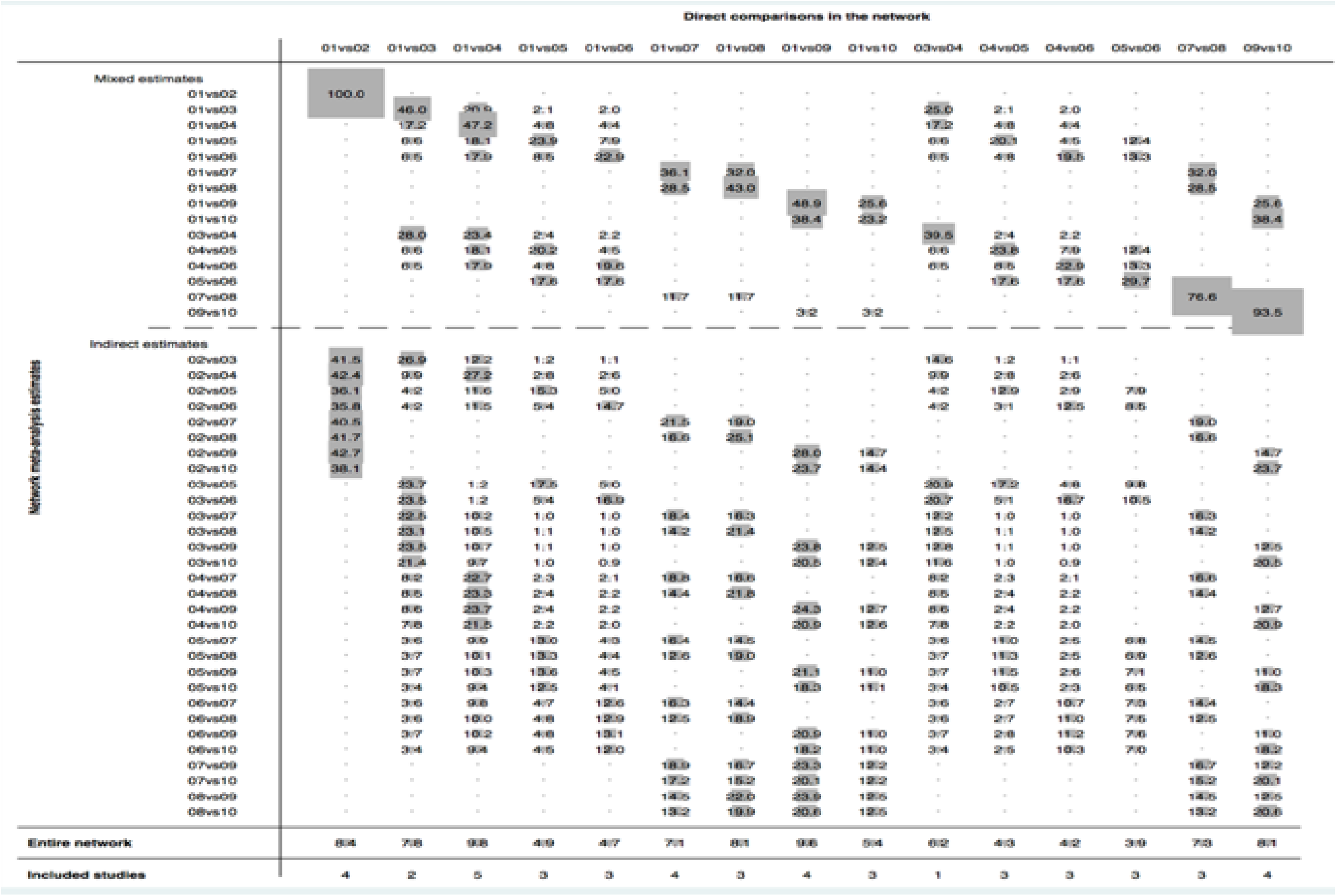

The size of each square is proportional to the weight attached to each direct summary effect (horizontal axis) for the estimation of each network summary effects (vertical axis). The numbers re-express the weights as percentages. 1: **Placebo** 2: **Amitriptyline** 3: **Pregabalin 150mg** 4: **Pregabalin 300mg** 5: **Pregabalin 450mg** 6: **Pregabalin 600mg** 7: **Duloxetine 60mg** 8: **Duloxetine 120mg** 9: **Milnacipran 100mg** 10: **Milnacipran 200mg.**

© 2022 Farag HM et al. *JAMA Network Open.*

eFigure 6C. Quality of life outcome: interval plot (estimates as Standardized Mean Difference)

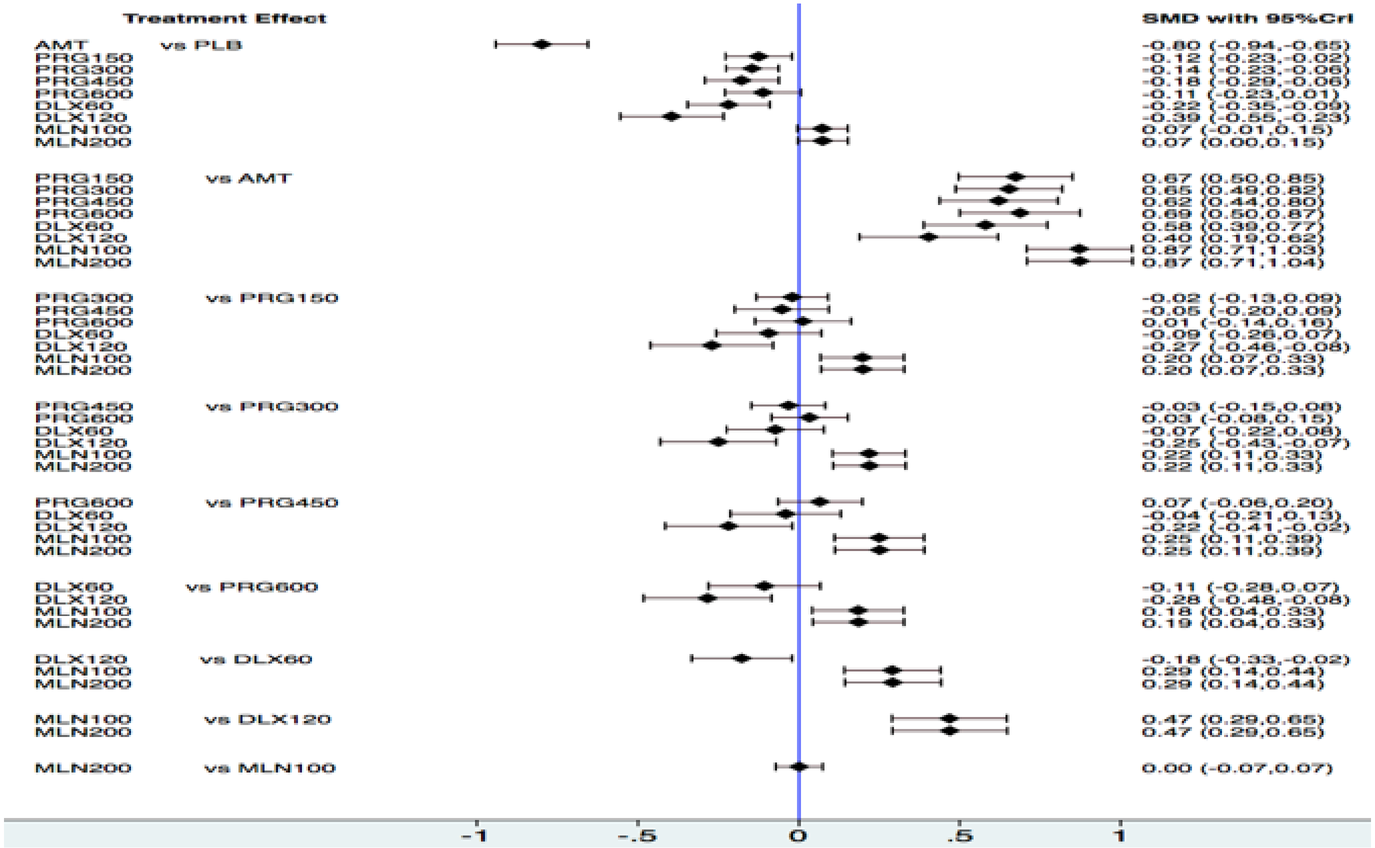

The black solid lines represent the credible intervals for summary standardized mean differences (SMD) for each comparison, and the blue line is the line of no effect (SMD equal to 0). CrI indicates credible interval; SMD, standardized mean difference. PLB: **Placebo;** AMT: **Amitriptyline;** PRG150: **Pregabalin 150mg;** PRG300: **Pregabalin 300mg;** PRG450: **Pregabalin 450mg;** PRG600: **Pregabalin 600mg;** DLX60: **Duloxetine 60mg;** DLX120: **Duloxetine 120mg;** MLN100: **Milnacipran 100mg;** MLN200: **Milnacipran 200mg.**

eFigure 6C. Quality of life outcome: interval plot (continued)

© 2022 Farag HM et al. *JAMA Network Open.*

_Comparison	_Effect_Size	_Standard_Error	_LCI	_UCI	_LPrI	_UPrI
AMT vs PLB	-.7977592	.0733483	-.9415192	-.6539991	-1.02572	-.5697982
PRG150 vs PLB	-.1243182	.0523985	-.2270174	-.0216191	-.3172852	.0686487
PRG300 vs PLB	-.1447249	.0413866	-.2258412	-.0636086	-.3220994	.0326495
PRG450 vs PLB	-.1769812	.058441	-.2915235	-.0624388	-.3794191	.0254568
PRG600 vs PLB	-.1109269	.0603885	-.2292863	.0074324	-.3165361	.0946822
DLX60 vs PLB	-.2177209	.0650238	-.3451653	-.0902766	-.4310881	-.0043538
DLX120 vs PLB	-.3944381	.0816128	-.5543962	-.23448	-.6375986	-.1512776
MLN100 vs PLB	.0732051	.0400259	-.0052442	.1516544	-.1024146	.2488247
MLN200 vs PLB	.074541	.0397228	-.0033142	.1523962	-.1006934	.2497754
PRG150 vs AMT	.6734409	.0900935	.4968609	.8500209	.4140778	.9328041
PRG300 vs AMT	.6530342	.0841796	.4880452	.8180232	.4050292	.9010392
PRG450 vs AMT	.620778	.0937641	.4370038	.8045522	.3542397	.8873163
PRG600 vs AMT	.6868322	.0950182	.5005999	.8730645	.4178219	.9558425
DLX60 vs AMT	.5800382	.0980349	.3878933	.7721832	.305042	.8550345
DLX120 vs AMT	.4033211	.1097044	.1883044	.6183377	.1046947	.7019475
MLN100 vs AMT	.8709642	.0836353	.7070421	1.034886	.6239911	1.117937
MLN200 vs AMT	.8723001	.0836167	.7084145	1.036186	.6253622	1.119238
PRG300 vs PRG150	-.0204067	.0568474	-.1318256	.0910123	-.2202913	.1794779
PRG450 vs PRG150	-.0526629	.0744837	-.1986483	.0933225	-.2826732	.1773474
PRG600 vs PRG150	.0133913	.0760299	-.1356245	.1624071	-.2194304	.246213
DLX60 vs PRG150	-.0934027	.0835125	-.2570842	.0702789	-.3401435	.1533381
DLX120 vs PRG150	-.2701198	.0969792	-.4601956	-.0800441	-.5430149	.0027752
MLN100 vs PRG150	.1975233	.0659593	.0682454	.3268012	-.0174434	.4124901
MLN200 vs PRG150	.1988592	.0658128	.0698685	.32785	-.0158563	.4135748
PRG450 vs PRG300	-.0322562	.058449	-.1468143	.0823018	-.2347071	.1701947
PRG600 vs PRG300	.033798	.0604053	-.0845943	.1521903	-.1718388	.2394347
DLX60 vs PRG300	-.072996	.0770807	-.2240714	.0780794	-.3077418	.1617498
DLX120 vs PRG300	-.2497132	.0915015	-.4290528	-.0703735	-.5118179	.0123916
MLN100 vs PRG300	.21793	.0575949	.1050461	.3308139	.0168525	.4190074
MLN200 vs PRG300	.2192659	.0574168	.1067311	.3318007	.0184735	.4200584
PRG600 vs PRG450	.0660542	.0667405	-.0647548	.1968632	-.1502565	.2823649
DLX60 vs PRG450	-.0407398	.0874284	-.2120962	.1306166	-.2949519	.2134724
DLX120 vs PRG450	-.217457	.1003766	-.4141915	-.0207224	-.4971368	.0622229
MLN100 vs PRG450	.2501862	.0708425	.1113375	.3890349	.0267004	.473672
MLN200 vs PRG450	.2515222	.0706859	.1129802	.3900641	.0283136	.4747307
DLX60 vs PRG600	-.106794	.0887398	-.2807207	.0671328	-.3635343	.1499464
DLX120 vs PRG600	-.2835112	.1015267	-.4824999	-.0845224	-.5655026	-.0015197
MLN100 vs PRG600	.184132	.0724449	.0421426	.3261214	-.0422079	.4104719
MLN200 vs PRG600	.185468	.0722713	.0438189	.3271171	-.0405614	.4114973
DLX120 vs DLX60	-.1767172	.0793552	-.3322506	-.0211838	-.415663	.0622287
MLN100 vs DLX60	.290926	.0763494	.141284	.440568	.0575204	.5243316
MLN200 vs DLX60	.2922619	.0761807	.1429504	.4415734	.0591646	.5253592
MLN100 vs DLX120	.4676432	.0909099	.2894631	.6458232	.2066921	.7285943
MLN200 vs DLX120	.4689791	.0907939	.2910263	.6469319	.2082538	.7297044
MLN200 vs MLN100	.0013359	.037302	-.0717745	.0744464	-.1708979	.1735698

PLB: **Placebo;** AMT: **Amitriptyline;** PRG150: **Pregabalin 150mg;** PRG300: **Pregabalin 300mg;** PRG450: **Pregabalin 450mg;** PRG600: **Pregabalin 600mg;** DLX60: **Duloxetine 60mg;** DLX120: **Duloxetine 120mg;** MLN100: **Milnacipran 100mg;** MLN200: **Milnacipran 200mg.**

© 2022 Farag HM et al. *JAMA Network Open.*

eFigure 6D. Quality of life outcome: SUCRA plots

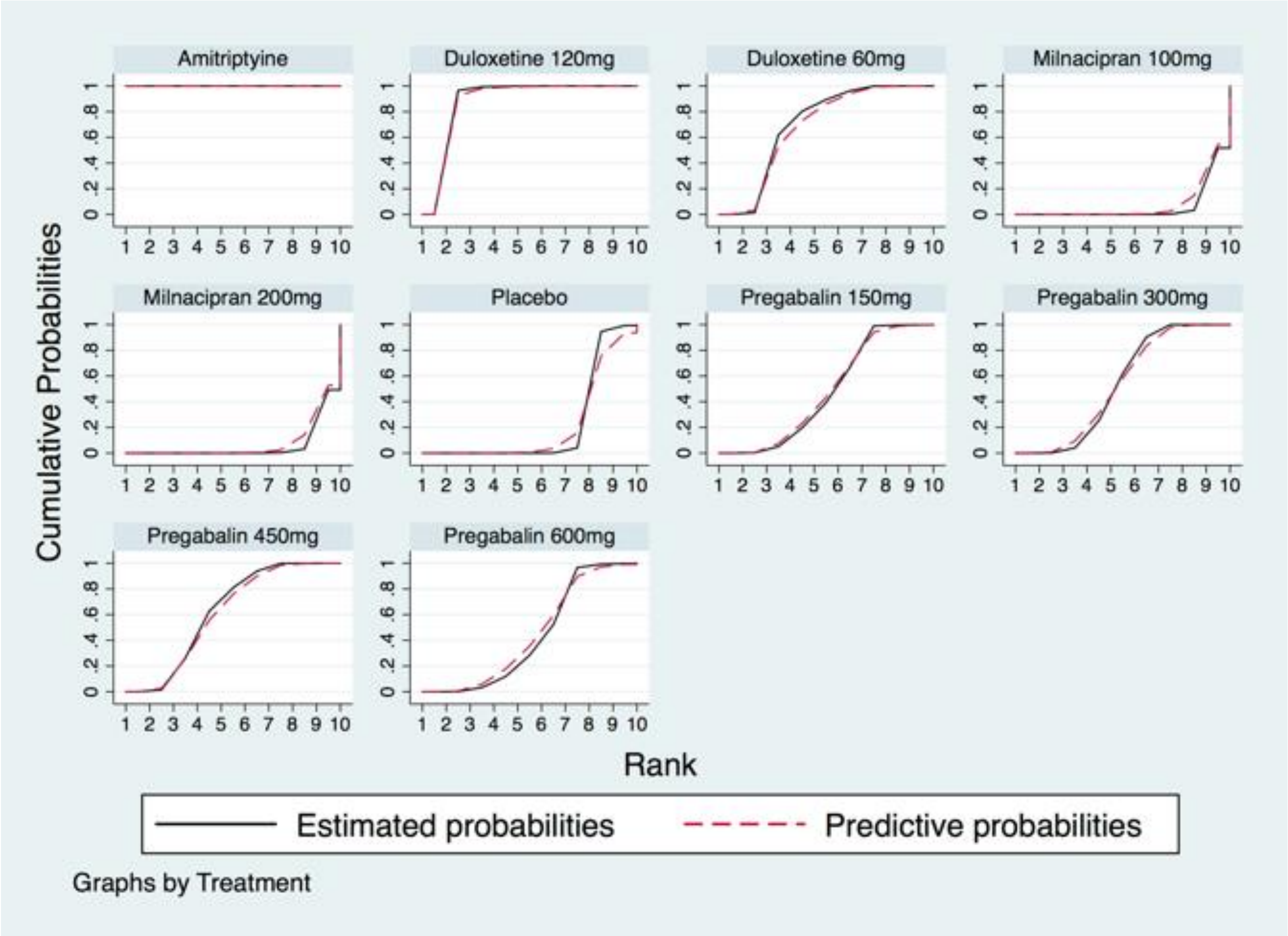

Cumulative probability curves for the fibromyalgia treatments network showing the estimated and predictive probabilities for each treatment being up to a specific rank.

eFigure 6D. Quality of life outcome: SUCRA plots (continued)

© 2022 Farag HM et al. *JAMA Network Open.*

Treatment	SUCRA	PrBest	MeanRank
Placebo	22.0	0.0	8.0
Amitriptyine	100.0	100.0	1.0
Pregabalin 150mg	47.8	0.0	5.7
Pregabalin 300mg	53.5	0.0	5.2
Pregabalin 450mg	62.8	0.0	4.4
Pregabalin 600mg	43.5	0.0	6.1
Duloxetine 60mg	70.0	0.0	3.7
Duloxetine 120mg	88.4	0.0	2.0
Milnacipran 100mg	6.1	0.0	9.4
Milnacipran 200mg	5.9	0.0	9.5

© 2022 Farag HM et al. *JAMA Network Open.*

eFigure 6E. Quality of life outcome: rankogram plots

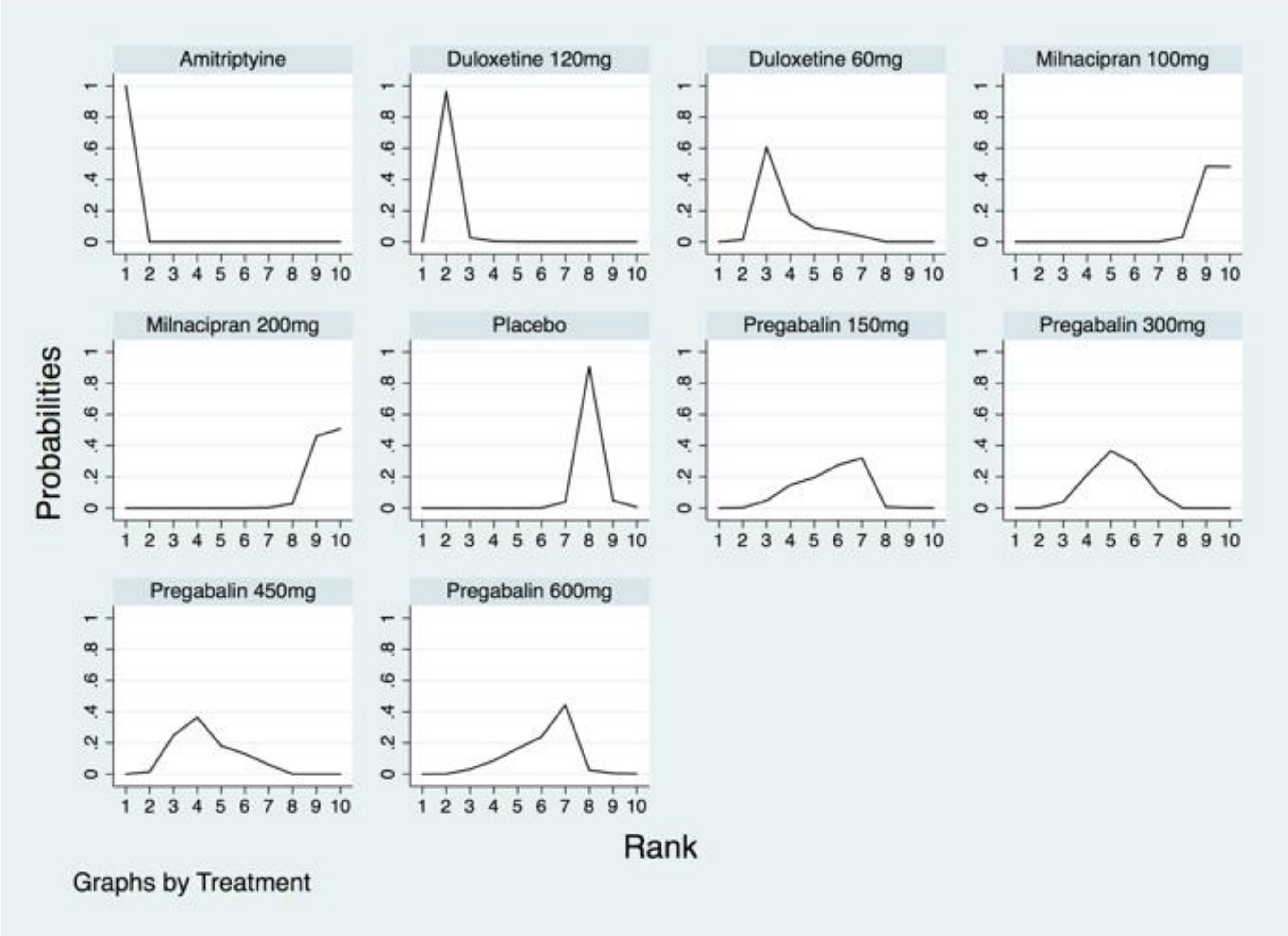

Rankogram plots for the fibromyalgia treatments network showing the probability for every treatment being at particular order.

eFigure 6E. Quality of life outcome: rankogram plots (continued)

© 2022 Farag HM et al. *JAMA Network Open.*

id and Rank	Treatment 1	2	3	4	5	6	7	8	9	10
1										
Best	0.0	100.0	0.0	0.0	0.0	0.0	0.0	0.0	0.0	0.0
2nd	0.0	0.0	0.3	0.1	1.2	0.1	1.1	97.2	0.0	0.0
3rd	0.0	0.0	5.0	4.7	24.6	3.4	60.3	2.0	0.0	0.0
4th	0.0	0.0	14.2	20.9	37.0	9.3	18.2	0.4	0.0	0.0
5th	0.0	0.0	20.3	34.5	19.2	16.2	9.7	0.1	0.0	0.0
6th	0.0	0.0	26.0	29.3	12.3	25.2	7.0	0.1	0.0	0.0
7th	3.8	0.0	33.2	10.4	5.6	42.7	3.8	0.0	0.3	0.1
8th	91.0	0.0	0.9	0.1	0.1	2.4	0.0	0.0	2.9	2.6
9th	4.5	0.0	0.0	0.0	0.0	0.4	0.0	0.0	49.0	46.0
Worst	0.7	0.0	0.0	0.0	0.0	0.3	0.0	0.0	47.7	51.2

1: **Placebo** 2: **Amitriptyline** 3: **Pregabalin 150mg** 4: **Pregabalin 300mg** 5: **Pregabalin 450mg** 6: **Pregabalin 600mg** 7: **Duloxetine 60mg** 8: **Duloxetine 120mg** 9: **Milnacipran 100mg** 10: **Milnacipran 200mg.**

© 2022 Farag HM et al. *JAMA Network Open.*

eFigure 6F. Quality of life outcome: comparison adjusted funnel plot

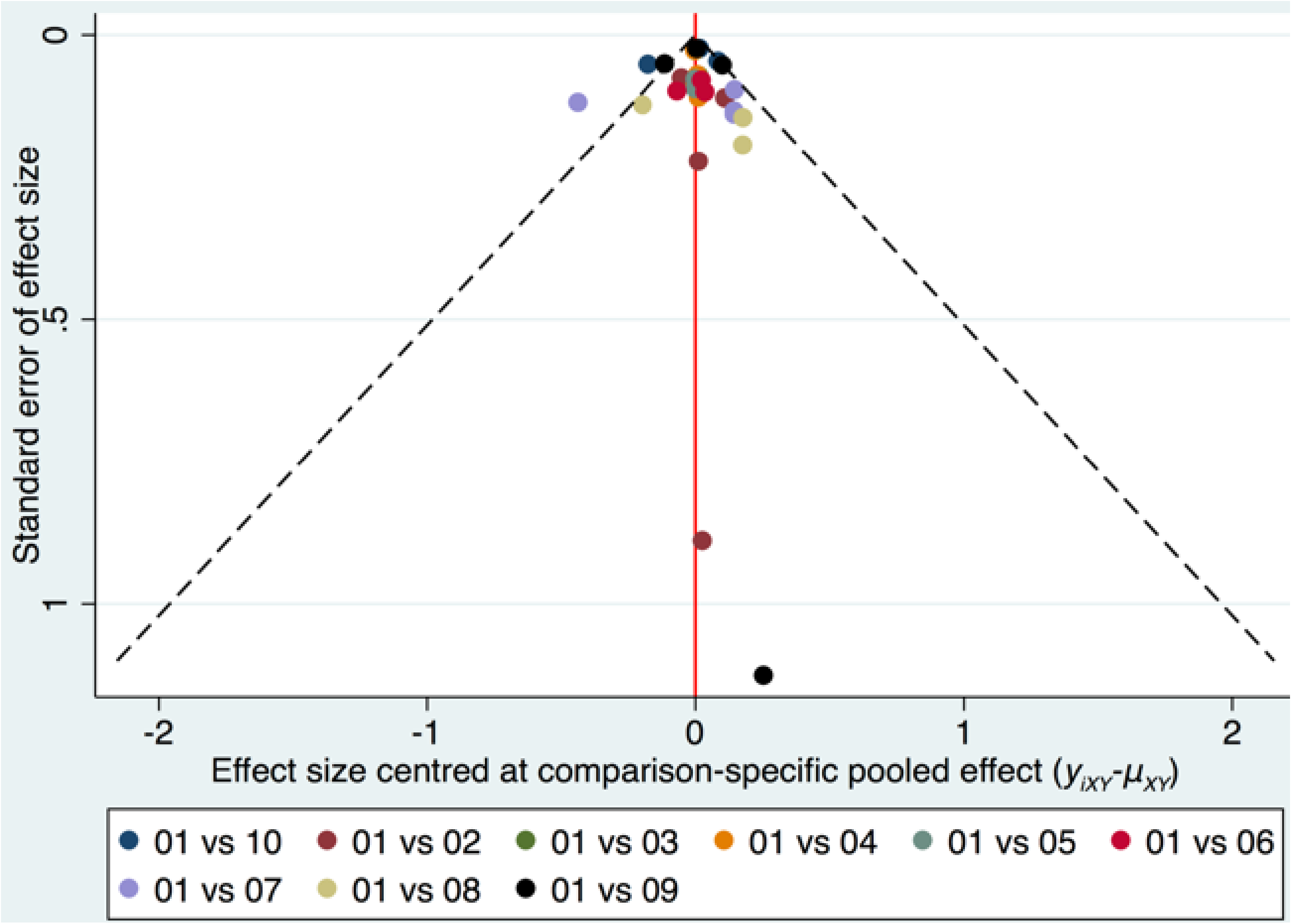

The red line represents the null hypothesis that the study-specific effect sizes do not differ from the respective comparison-specific pooled effect estimates. Different colors correspond to different comparisons. 1: **Placebo** 2: **Amitriptyline** 3: **Pregabalin 150mg** 4: **Pregabalin 300mg** 5: **Pregabalin 450mg** 6: **Pregabalin 600mg** 7: **Duloxetine 60mg** 8: **Duloxetine 120mg** 9: **Milnacipran 100mg** 10: **Milnacipran 200mg.**

© 2022 Farag HM et al. *JAMA Network Open.*

eFigure 6G. Quality of life outcome: network pattern

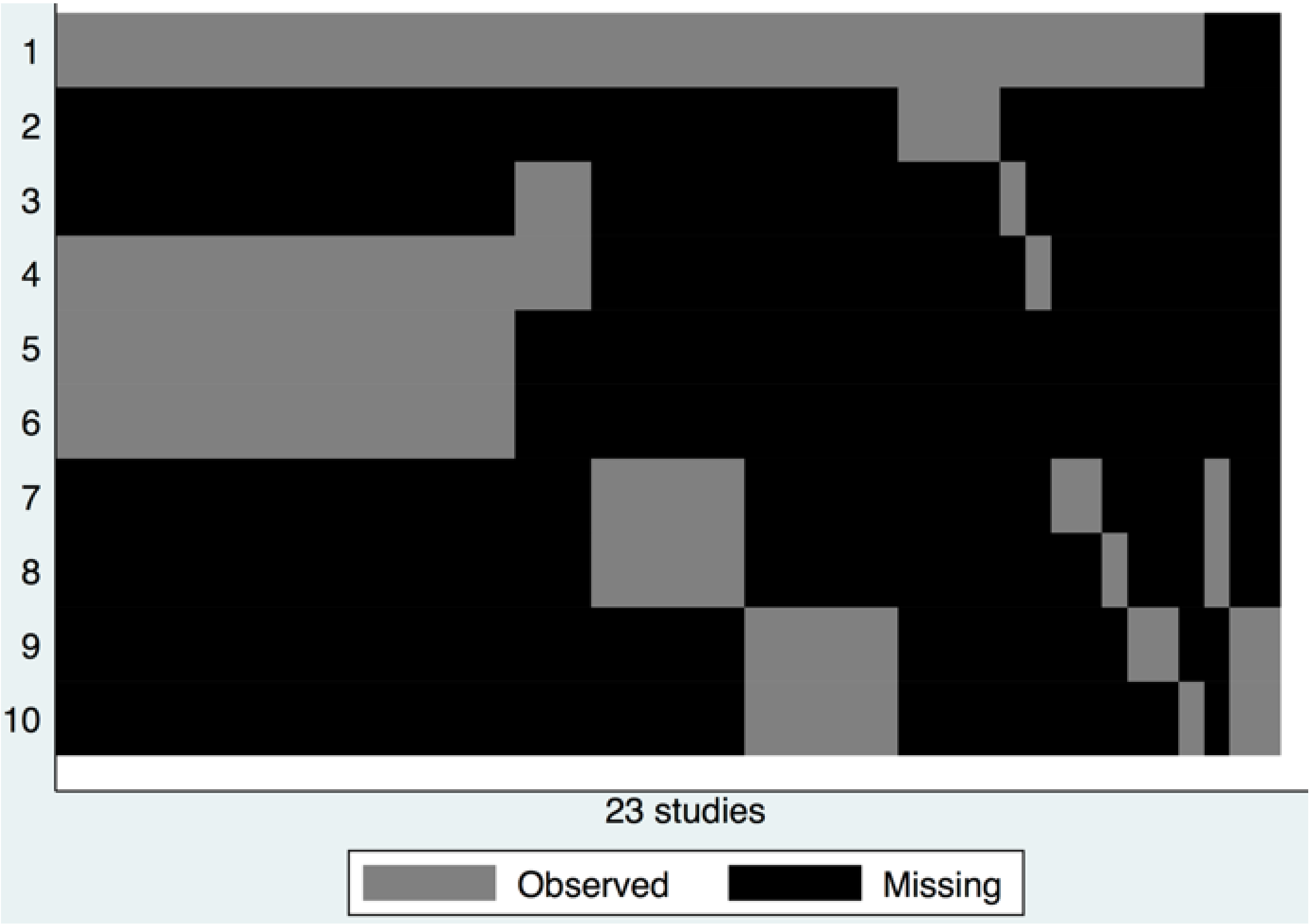

eFigure 7. Acceptability Outcome: Results

© 2022 Farag HM et al. *JAMA Network Open.*

eFigure 7A. Acceptability outcome: contribution plot

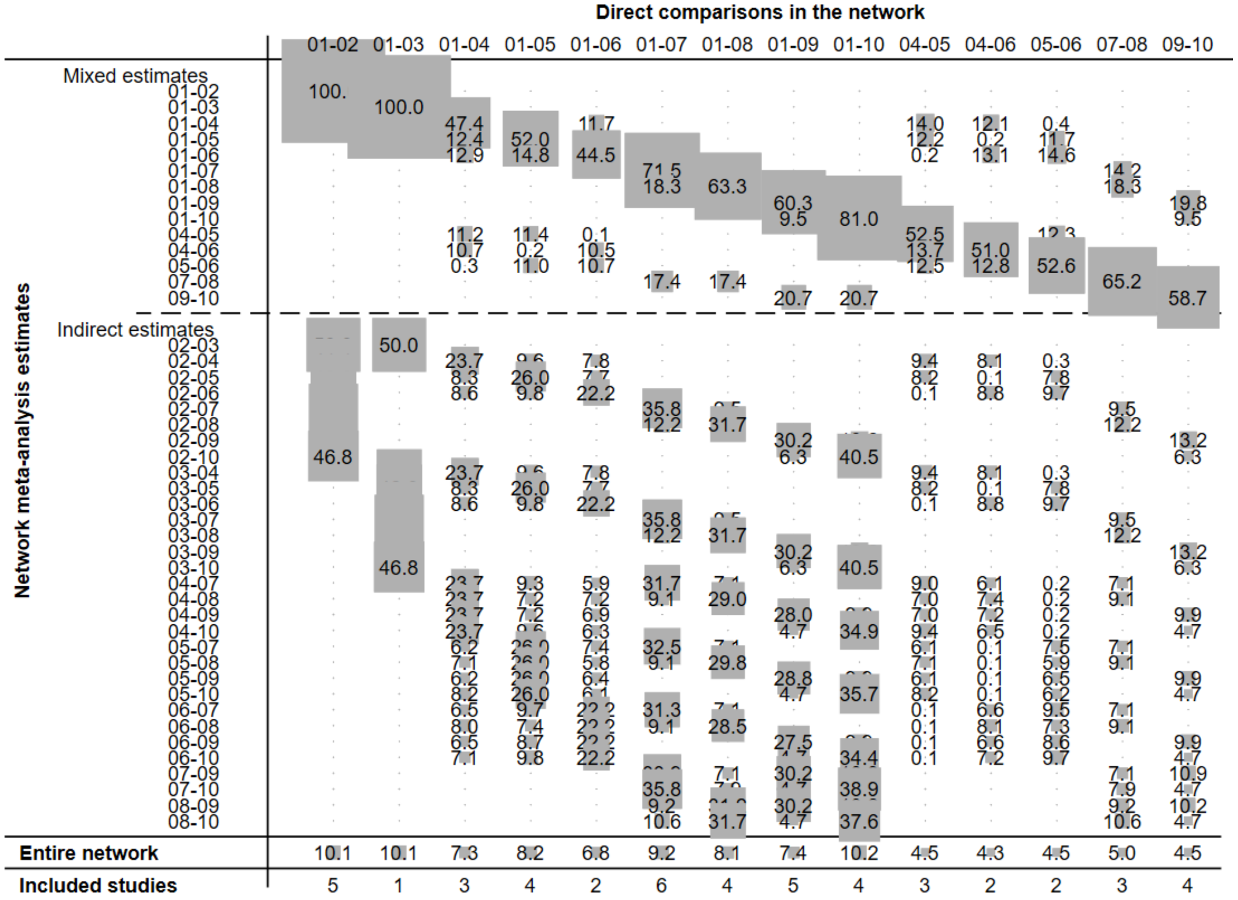

The size of each square is proportional to the weight attached to each direct summary effect (horizontal axis) for the estimation of each network summary effects (vertical axis). The numbers re-express the weights as percentages. 1: **Placebo** 2: **Amitriptyline** 3: **Pregabalin 150mg** 4: **Pregabalin 300mg** 5: **Pregabalin 450mg** 6: **Pregabalin 600mg** 7: **Duloxetine 60mg** 8: **Duloxetine 120mg** 9: **Milnacipran 100mg** 10: **Milnacipran 200mg.**

eTable 11. Acceptability Outcome: Direct Pairwise Comparisons (Estimates as Odds Ratios [ORs] and 95% Credible Intervals [95% CrI])

Comparison	Odds Ratio	95% CrI		Median	Standard deviation	Monte Carlo error
Placebo vs. Amitriptyline	0.7842	0.3109	1.658	0.3568	0.7133	0.008541

© 2022 Farag HM et al. *JAMA Network Open.*

Placebo vs. Pregabalin 150mg	4.159	1.336	10.52	2.538	3.565	0.04166
Placebo vs. Pregabalin 300mg	1.622	1.041	2.399	0.347	1.591	0.004673
Placebo vs. Pregabalin 450mg	1.884	1.236	2.744	0.3825	1.851	0.005447
Placebo vs. Pregabalin 6000mg	2.202	1.359	3.365	0.5161	2.147	0.006242
Placebo vs. Duloxetine 60mg	1.744	1.276	2.315	0.2635	1.726	0.00367
Placebo vs. Duloxetine 120mg	2.505	1.777	3.408	0.414	2.474	0.005123
Placebo vs. Milnacipran 100mg	1.914	1.377	2.761	0.3544	1.864	0.005302
Placebo vs. Milnacipran 200mg	2.581	1.855	3.569	0.4404	2.537	0.005863

**Comparison**	**Odds Ratio**	**2.50%**	**97.50%**	**Standard deviation**	**median**	**Monte Carlo error**
Amitriptyline vs. Pregabalin 150mg	6.349	1.41	19.11	5.03	5.05	0.09318
Amitriptyline vs. Pregabalin 300mg	2.488	0.8728	5.618	1.26	2.216	0.02704

© 2022 Farag HM et al. *JAMA Network Open.*

Amitriptyline vs. Pregabalin 450mg	2.889	1.018	6.455	1.451	2.577	0.03125
Amitriptyline vs. Pregabalin 600mg	3.382	1.133	7.717	1.766	3.017	0.03706
Amitriptyline vs. Duloxetine 60mg	2.669	1.002	5.818	1.277	2.397	0.02855
Amitriptyline vs. Duloxetine 120mg	3.834	1.42	8.356	1.84	3.456	0.04031
Amitriptyline vs. Milnacipran 100mg	2.936	1.049	6.534	1.448	2.637	0.03145
Amitriptyline vs. Milnacipran 200mg	3.964	1.435	8.727	1.946	3.576	0.04308
Pregabalin 150mg vs. Pregabalin 300mg	0.513	0.1396	1.294	0.306	0.4429	0.004736
Pregabalin 150mg vs. Pregabalin 450mg	0.5958	0.1623	1.486	0.3517	0.5181	0.005449
Pregabalin 150mg vs. Pregabalin 600mg	0.695	0.1888	1.777	0.4202	0.5968	0.006132

© 2022 Farag HM et al. *JAMA Network Open.*

Pregabalin 150mg vs. Duloxetine 60mg	0.5509	0.1591	1.339	0.3127	0.4829	0.004842
Pregabalin 150mg vs. Duloxetine 120mg	0.7912	0.2265	1.945	0.4531	0.6919	0.006899
Pregabalin 150mg vs. Milnacipran 100mg	0.6067	0.1723	1.528	0.3625	0.5243	0.0054
Pregabalin 150mg vs. Milnacipran 200mg	0.8163	0.2288	2.024	0.4735	0.7087	0.007188
**Comparison**	**Odds Ratio**	**2.50%**	**97.50%**	**Standard deviation**	**median**	**Monte Carlo error**
Pregabalin 300mg vs. Pregabalin 450mg	1.187	0.7853	1.714	0.2369	1.166	0.002738
Pregabalin 300mg vs. Pregabalin 600mg	1.42	0.7311	2.515	0.4624	1.356	0.005676
Pregabalin 300mg vs. Duloxetine 60mg	1.124	0.653	1.801	0.2963	1.087	0.003846
Pregabalin 300mg vs.	1.615	0.9161	2.636	0.441	1.557	0.005447

© 2022 Farag HM et al. *JAMA Network Open.*

Duloxetine 120mg						
Pregabalin 300mg vs. Milnacipran 100mg	1.236	0.7106	2.109	0.3662	1.175	0.00512
Pregabalin 300mg vs. Milnacipran 200mg	1.665	0.9591	2.77	0.4684	1.599	0.006217
Pregabalin 450mg vs. Pregabalin 600mg	1.218	0.6372	2.148	0.3914	1.161	0.005128
Pregabalin 450mg vs. Duloxetine 60mg	0.9636	0.5685	1.532	0.2473	0.9317	0.003386
Pregabalin 450mg vs. Duloxetine 120mg	1.385	0.8031	2.247	0.3691	1.334	0.004862
Pregabalin 450mg vs. Milnacipran 100mg	1.059	0.6224	1.793	0.3057	1.012	0.004405
Pregabalin 450mg vs. Milnacipran 200mg	1.427	0.8362	2.346	0.3914	1.371	0.005308
Pregabalin 600mg vs.	0.8354	0.4645	1.388	0.2368	0.8029	0.003332

© 2022 Farag HM et al. *JAMA Network Open.*

Duloxetine 60mg						
Pregabalin 600mg vs. Duloxetine 120mg	1.2	0.654	2.013	0.3476	1.154	0.004415
**Comparison**	**Odds Ratio**	**2.50%**	**97.50%**	**Standard deviation**	**median**	**Monte Carlo error**
Pregabalin 600mg vs. Milnacipran 100mg	0.9168	0.5162	1.592	0.2828	0.8702	0.003482
Pregabalin 600mg vs. Milnacipran 200mg	1.236	0.689	2.098	0.3652	1.183	0.004458
Duloxetine 60mg vs. Duloxetine 120mg	1.453	1.045	1.983	0.2383	1.433	0.002271
Duloxetine 60mg vs. Milnacipran 100mg	1.125	0.7106	1.805	0.2831	1.079	0.004119
Duloxetine 60mg vs. Milnacipran 200mg	1.515	0.9565	2.351	0.3577	1.467	0.00478
Duloxetine 120mg vs. Milnacipran 100mg	0.786	0.4886	1.28	0.206	0.7539	0.002792
Duloxetine 120mg vs.	1.059	0.6579	1.664	0.2602	1.024	0.003302

© 2022 Farag HM et al. *JAMA Network Open.*

Milnacipran 200mg						
Milnacipran 100mg vs. Milnacipran 200mg	1.371	0.9337	1.861	0.2331	1.362	0.002947

eFigure 7B. Acceptability outcome: surface under the cumulative ranking (SUCRA) plots

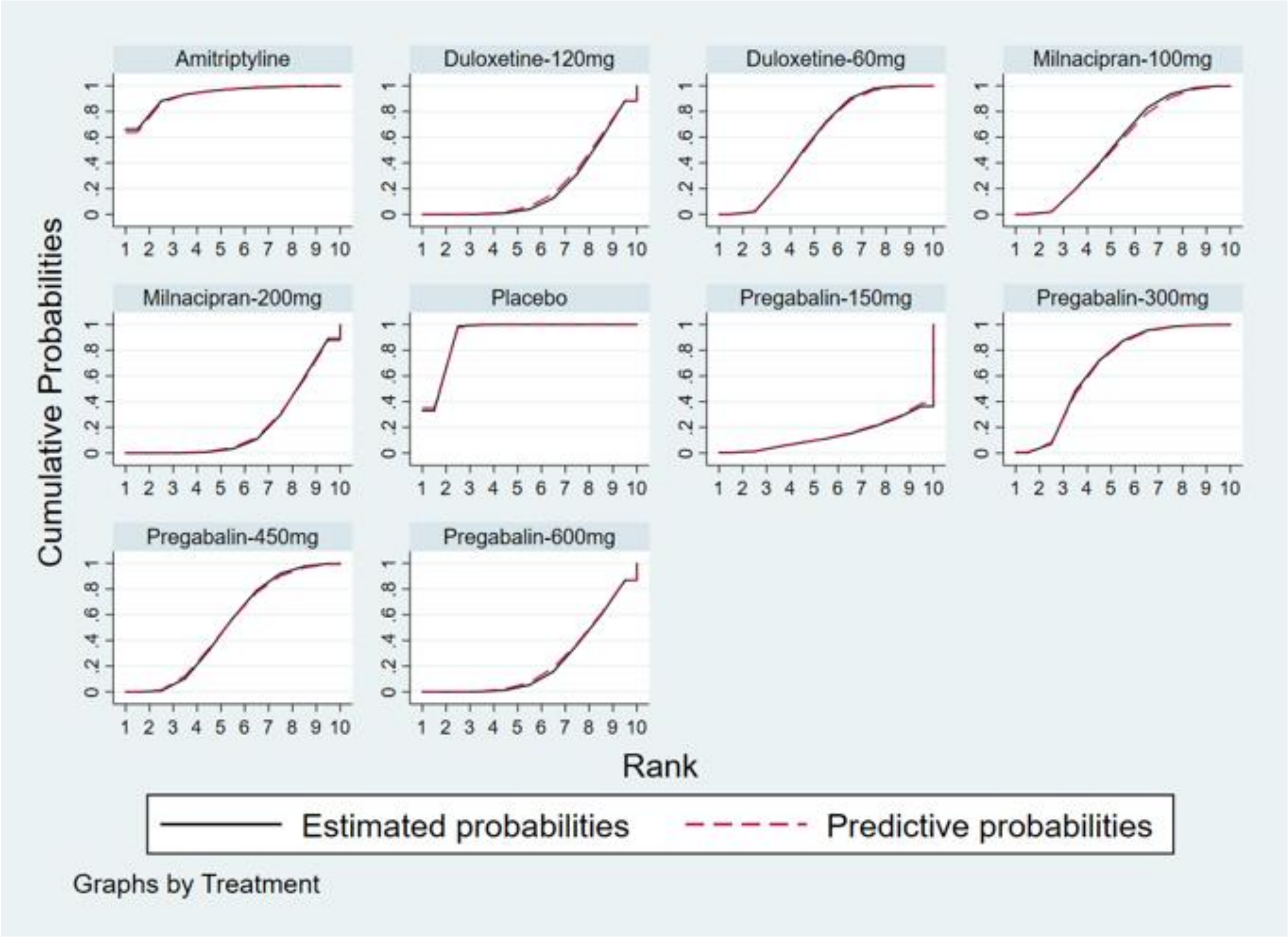

Cumulative probability curves for the fibromyalgia treatments network showing the estimated and predictive probabilities for each treatment being up to a specific rank.

eFigure 7B. Acceptability outcome: SUCRA plots (continued)

© 2022 Farag HM et al. *JAMA Network Open.*

Treatment Relative Ranking of Estimated probabilities

Treatment	SUCRA	PrBest	MeanRank
Placebo	92.4	33.0	01.7
Amitriptyline	93.2	66.2	01.6
Pregabalin-150mg	14.0	00.5	08.7
Pregabalin-300mg	67.6	00.3	03.9
Pregabalin-450mg	52.2	00.0	05.3
Pregabalin-600mg	22.9	00.0	07.9
Duloxetine-60mg	59.3	00.0	04.7
Duloxetine-120mg	21.8	00.0	08.0
Milnacipran-100mg	55.2	00.0	05.0
Milnacipran-200mg	21.5	00.0	08.1

Treatment Relative Ranking of Predictive probabilities

Treatment	SUCRA	PrBest	MeanRank
Placebo	92.5	35.1	01.7
Amitriptyline	92.7	63.9	01.7
Pregabalin-150mg	14.5	00.3	08.7
Pregabalin-300mg	67.1	00.7	04.0
Pregabalin-450mg	52.0	00.0	05.3
Pregabalin-600mg	23.8	00.0	07.9
Duloxetine-60mg	58.8	00.0	04.7
Duloxetine-120mg	23.1	00.0	07.9
Milnacipran-100mg	53.9	00.0	05.2
Milnacipran-200mg	21.6	00.0	08.1

eFigure 7C. Acceptability outcome: rankogram plots

© 2022 Farag HM et al. *JAMA Network Open.*

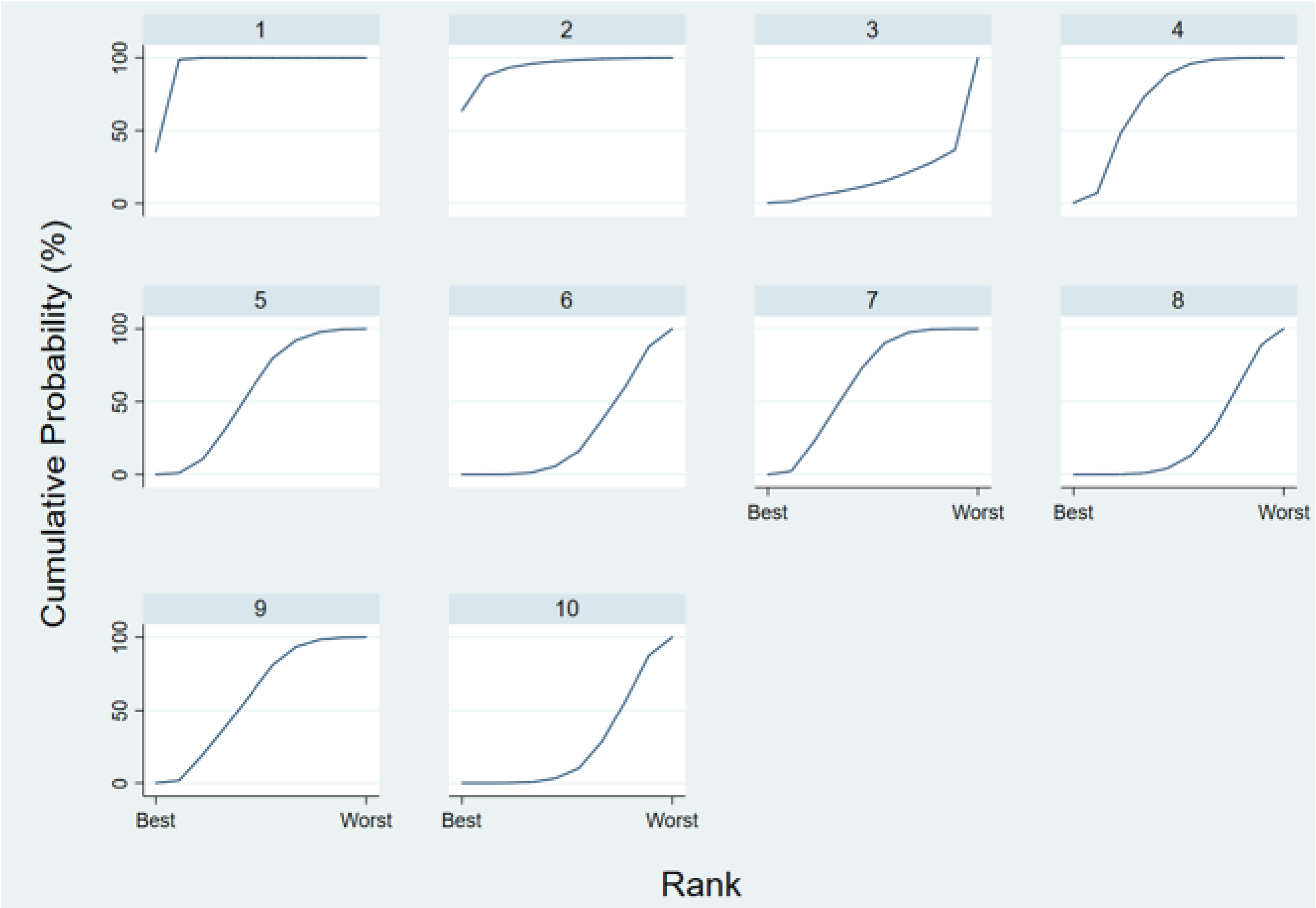

1: **Placebo** 2: **Amitriptyline** 3: **Pregabalin 150mg** 4: **Pregabalin 300mg** 5: **Pregabalin 450mg** 6: **Pregabalin 600mg** 7: **Duloxetine 60mg** 8: **Duloxetine 120mg** 9: **Milnacipran 100mg** 10: **Milnacipran 200mg.**

eFigure 7C. Acceptability outcome: rankogram plots (continued)

© 2022 Farag HM et al. *JAMA Network Open.*

id and Rank	Treatment 1	2	3	4	5	6	7	8	9	10
1										
Best	35.6	63.7	0.3	0.4	0.0	0.0	0.0	0.0	0.0	0.0
2nd	63.3	23.8	1.0	6.8	1.1	0.0	2.3	0.0	1.8	0.0
3rd	1.2	5.8	3.8	41.0	9.4	0.1	21.1	0.1	17.5	0.1
4th	0.0	2.8	2.8	25.1	21.8	1.2	24.9	0.9	19.9	0.6
5th	0.0	1.7	3.3	15.2	24.6	4.4	24.2	3.1	21.0	2.5
6th	0.0	0.9	4.0	7.5	22.9	10.5	17.8	8.7	20.9	6.8
7th	0.0	0.7	5.9	2.9	12.3	21.5	7.2	18.7	12.3	18.4
8th	0.0	0.4	6.8	0.9	5.5	22.7	2.1	28.6	5.0	28.1
9th	0.0	0.2	8.7	0.2	2.0	27.4	0.4	28.6	1.4	31.0
Worst	0.0	0.1	63.2	0.0	0.3	12.2	0.0	11.3	0.3	12.5
MEAN RANK	1.7	1.6	8.7	3.9	5.3	7.9	4.7	8.0	5.1	8.1
SUCRA	0.9	0.9	0.1	0.7	0.5	0.2	0.6	0.2	0.5	0.2

1: **Placebo** 2: **Amitriptyline** 3: **Pregabalin 150mg** 4: **Pregabalin 300mg** 5: **Pregabalin 450mg** 6: **Pregabalin 600mg** 7: **Duloxetine 60mg** 8: **Duloxetine 120mg** 9: **Milnacipran 100mg** 10: **Milnacipran 200mg.**

© 2022 Farag HM et al. *JAMA Network Open.*

eFigure 7D. Acceptability outcome: comparison adjusted funnel plot

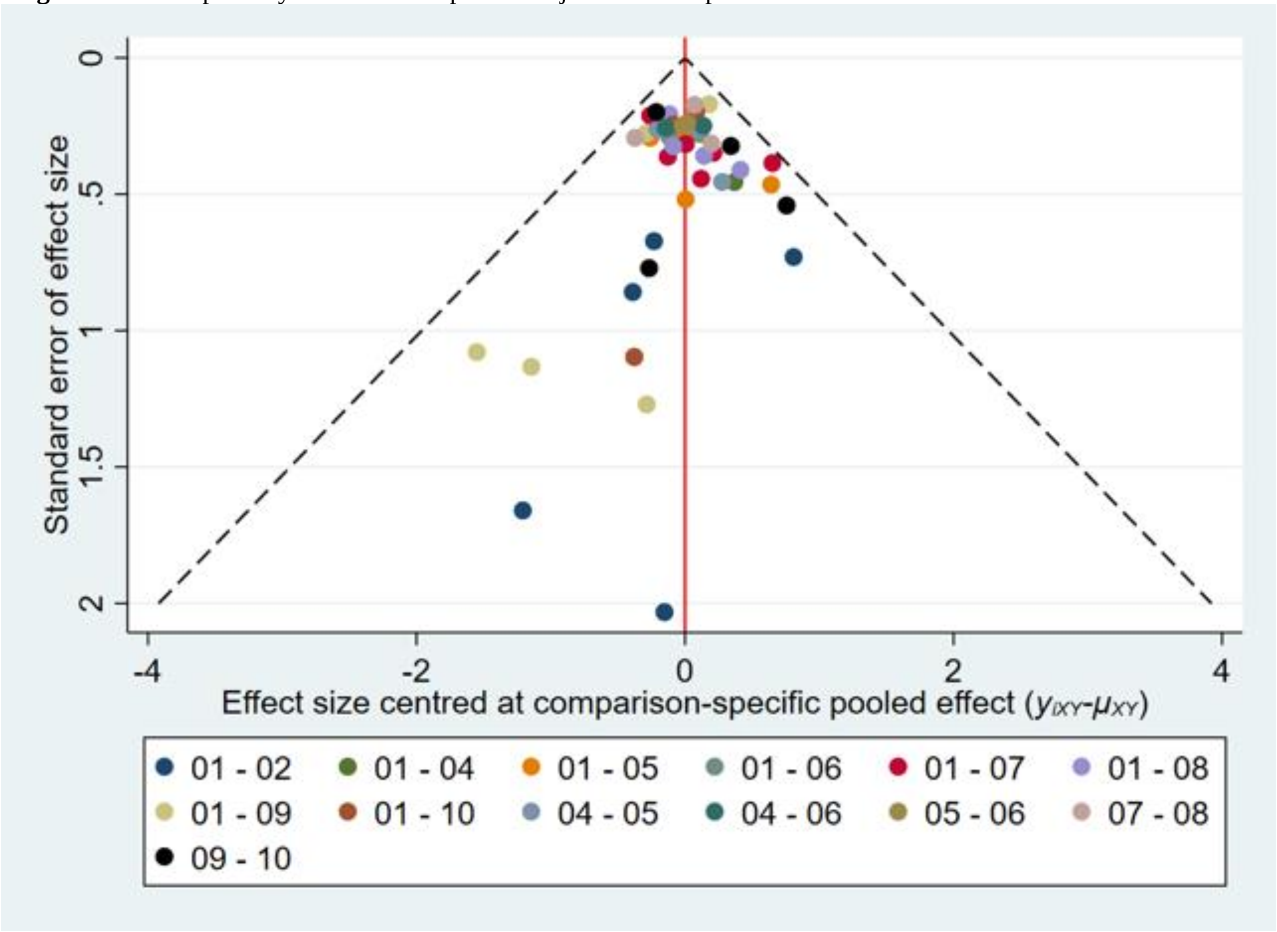

The red line represents the null hypothesis that the study-specific effect sizes do not differ from the respective comparison-specific pooled effect estimates. Different colors correspond to different comparisons. 1: **Placebo** 2: **Amitriptyline** 3: **Pregabalin 150mg** 4: **Pregabalin 300mg** 5: **Pregabalin 450mg** 6: **Pregabalin 600mg** 7: **Duloxetine 60mg** 8: **Duloxetine 120mg** 9: **Milnacipran 100mg** 10: **Milnacipran 200mg.**

eFigures 8. Cluster Ranking Plots for Relative Effectiveness and Acceptability. Each plot shows SUCRA values on a scale of 0% to 100% for 2 outcomes. Each color represents a group of treatments that belongs to the same cluster. The upper right quadrant represents the more favorable interventions on the joint outcomes; lower right quadrant, more favorable on the horizontal axis

© 2022 Farag HM et al. *JAMA Network Open.*

outcome but less on the vertical axis outcome; lower left quadrant, less favorable on both outcomes; the upper left quadrant, more favorable on the vertical axis outcome but less on the horizontal axis outcome.

eFigure 8A. Cluster ranking for sleep vs acceptability

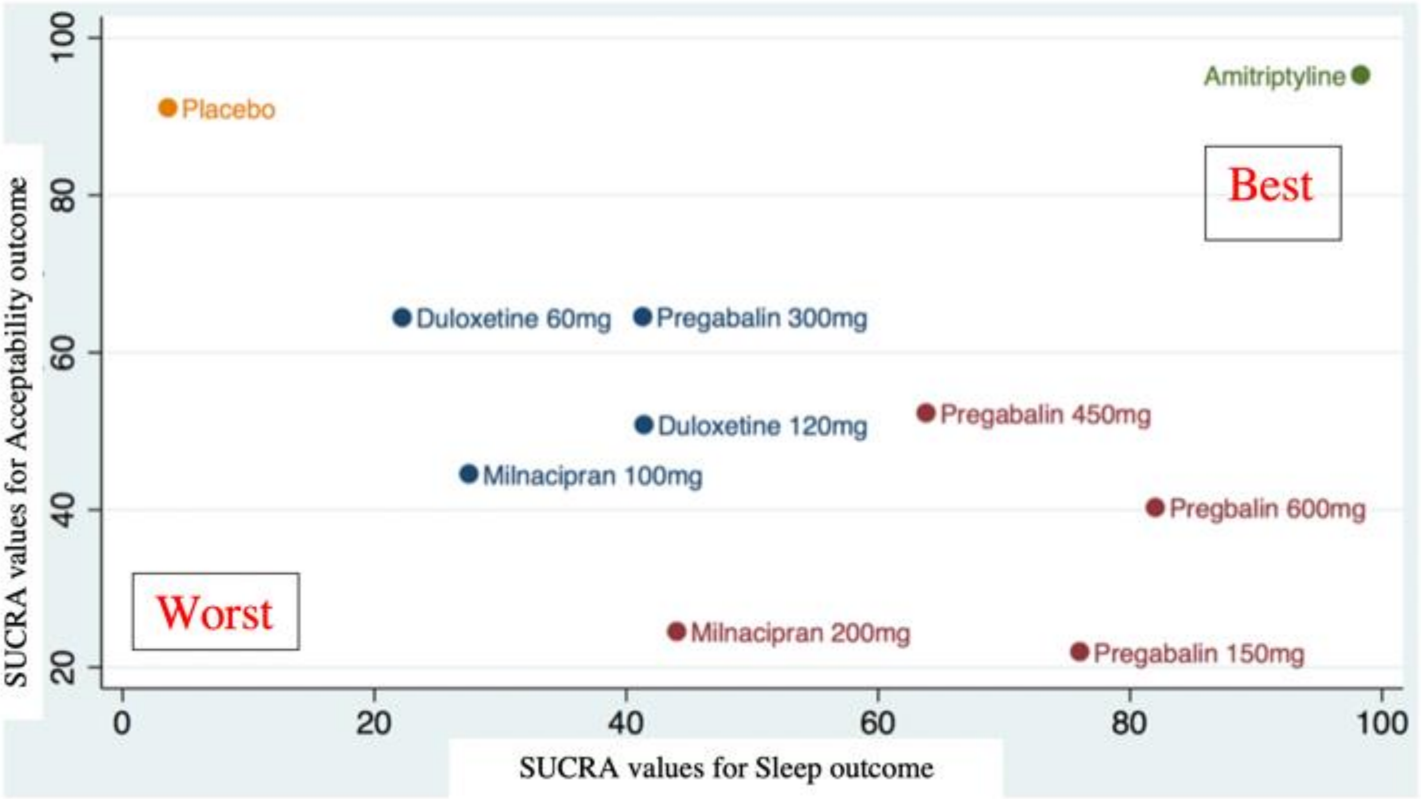

eFigure 8B. Cluster ranking for depression vs acceptability (duloxetine 60mg "right" and pregabalin 300mg "left" labels overlap)

© 2022 Farag HM et al. *JAMA Network Open.*

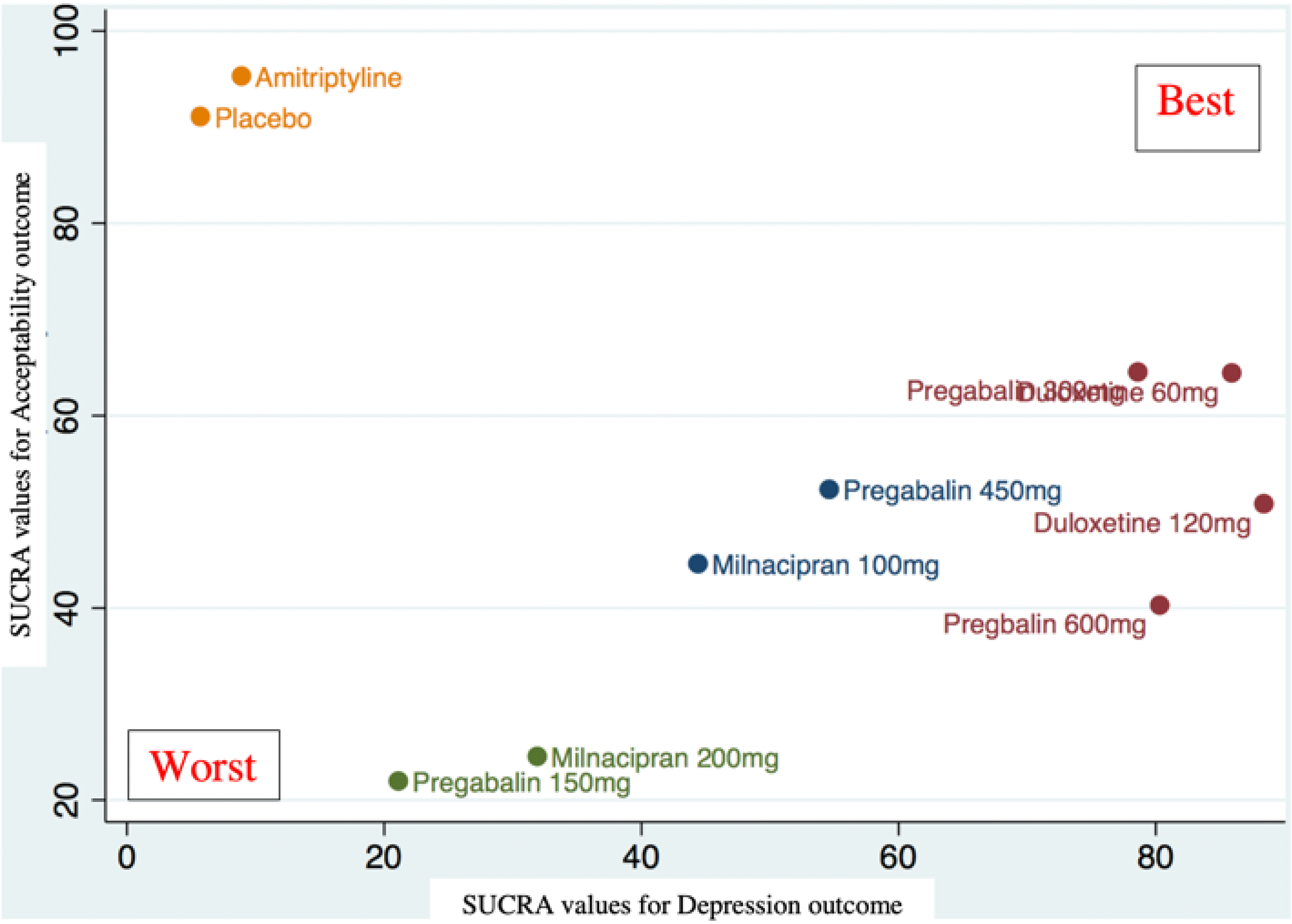

eFigure 8C. Cluster ranking for fatigue vs acceptability

© 2022 Farag HM et al. *JAMA Network Open.*

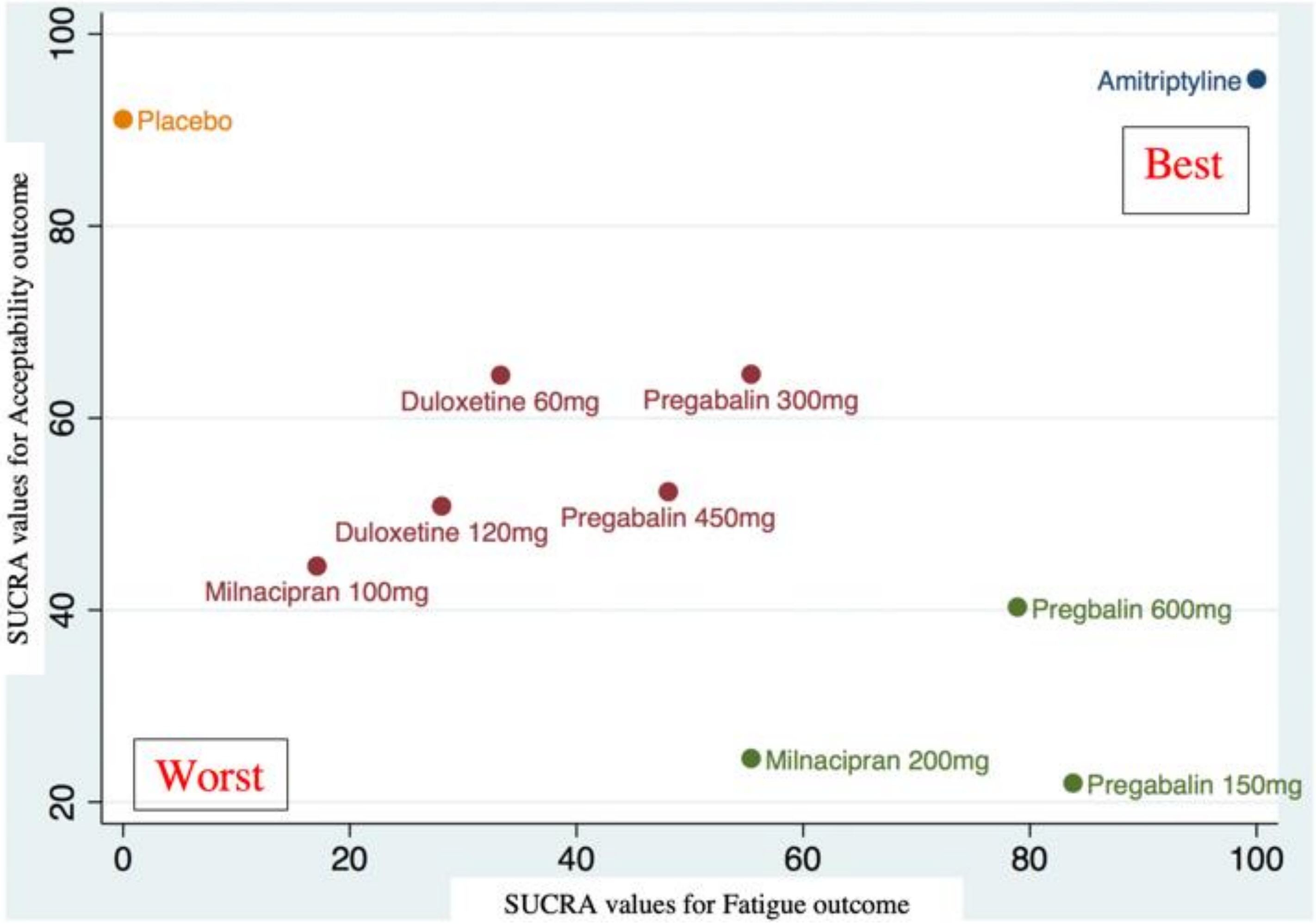

© 2022 Farag HM et al. *JAMA Network Open.*

eFigure 8D. Cluster ranking for quality of life (QoL) vs acceptability

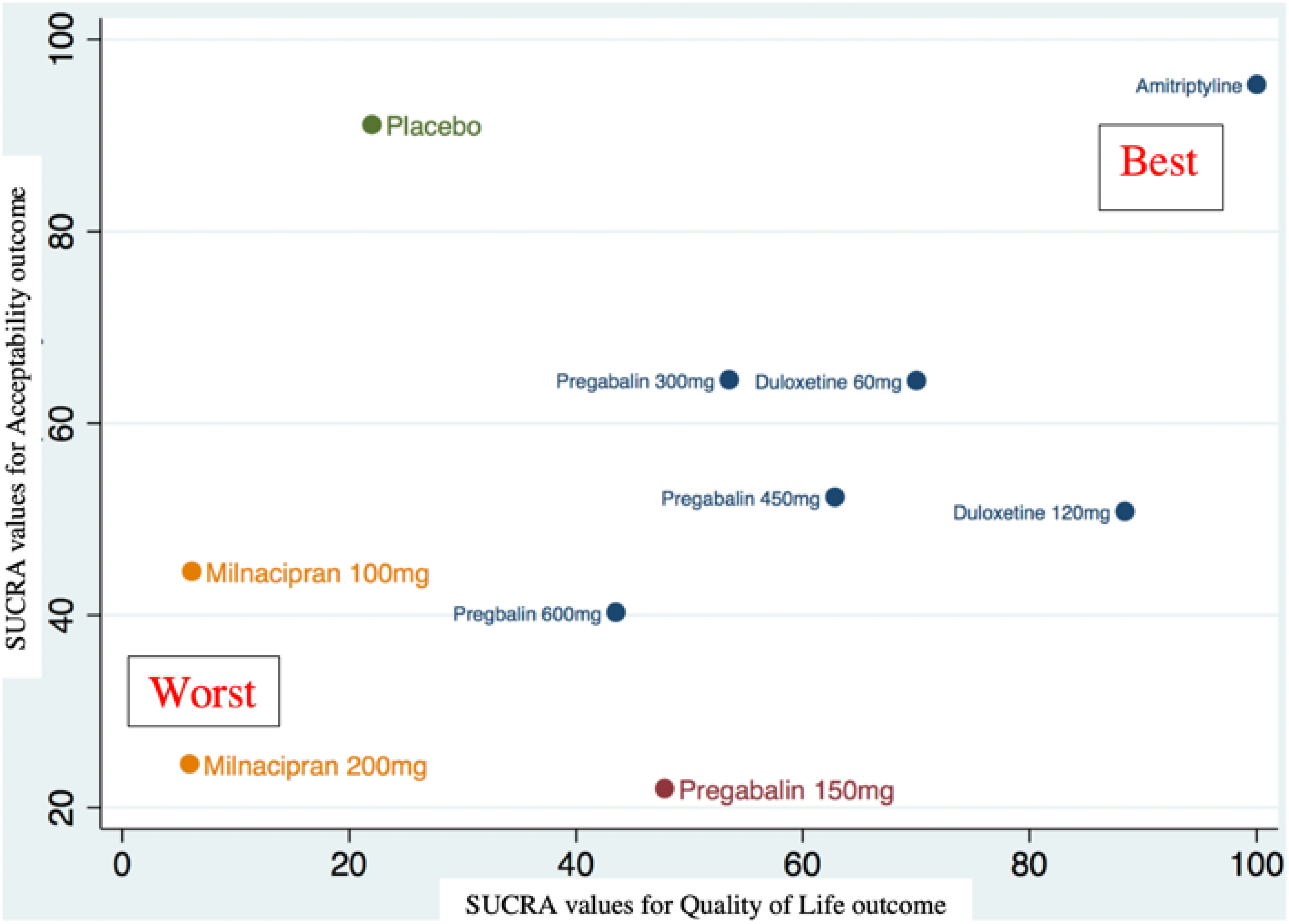

eFigure 8E. Cluster ranking for pain vs fatigue (milnacipran 200mg "left" and pregabalin 300mg "right" labels overlap)

© 2022 Farag HM et al. *JAMA Network Open.*

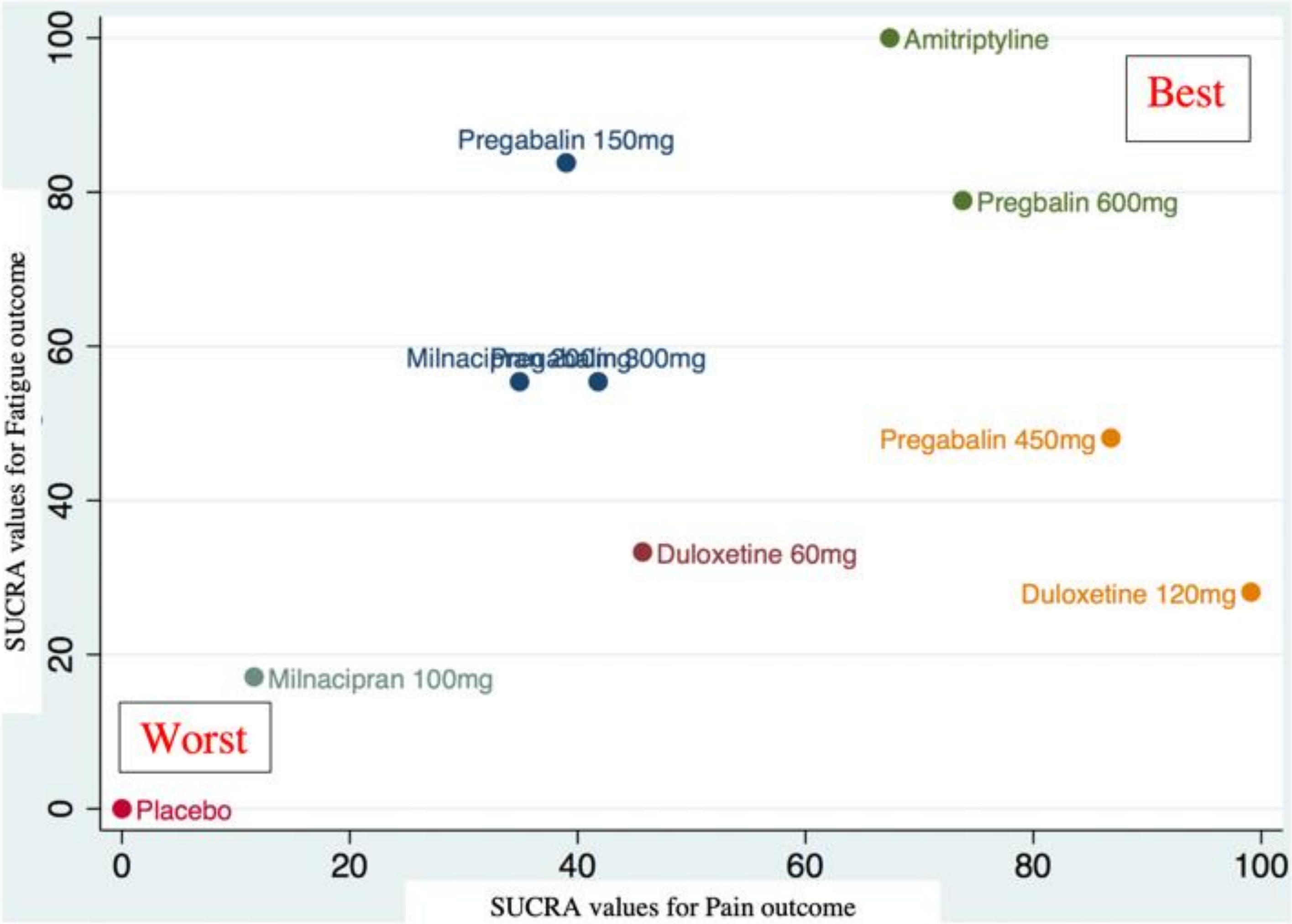

eFigure 8F. Cluster ranking for fatigue vs depression

© 2022 Farag HM et al. *JAMA Network Open.*

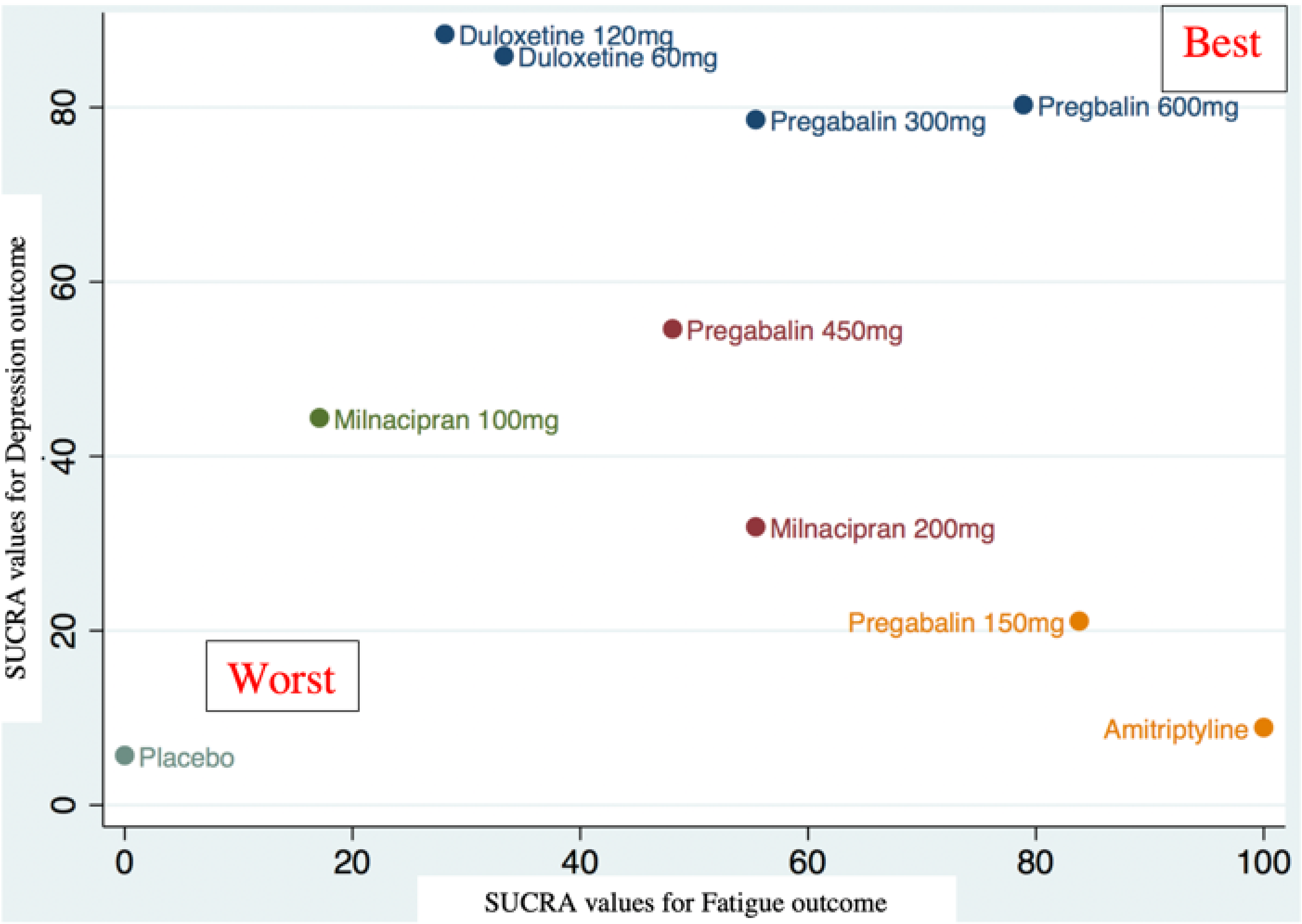

© 2022 Farag HM et al. *JAMA Network Open.*

eFigure 8G. Cluster ranking for quality of life (QoL) vs sleep

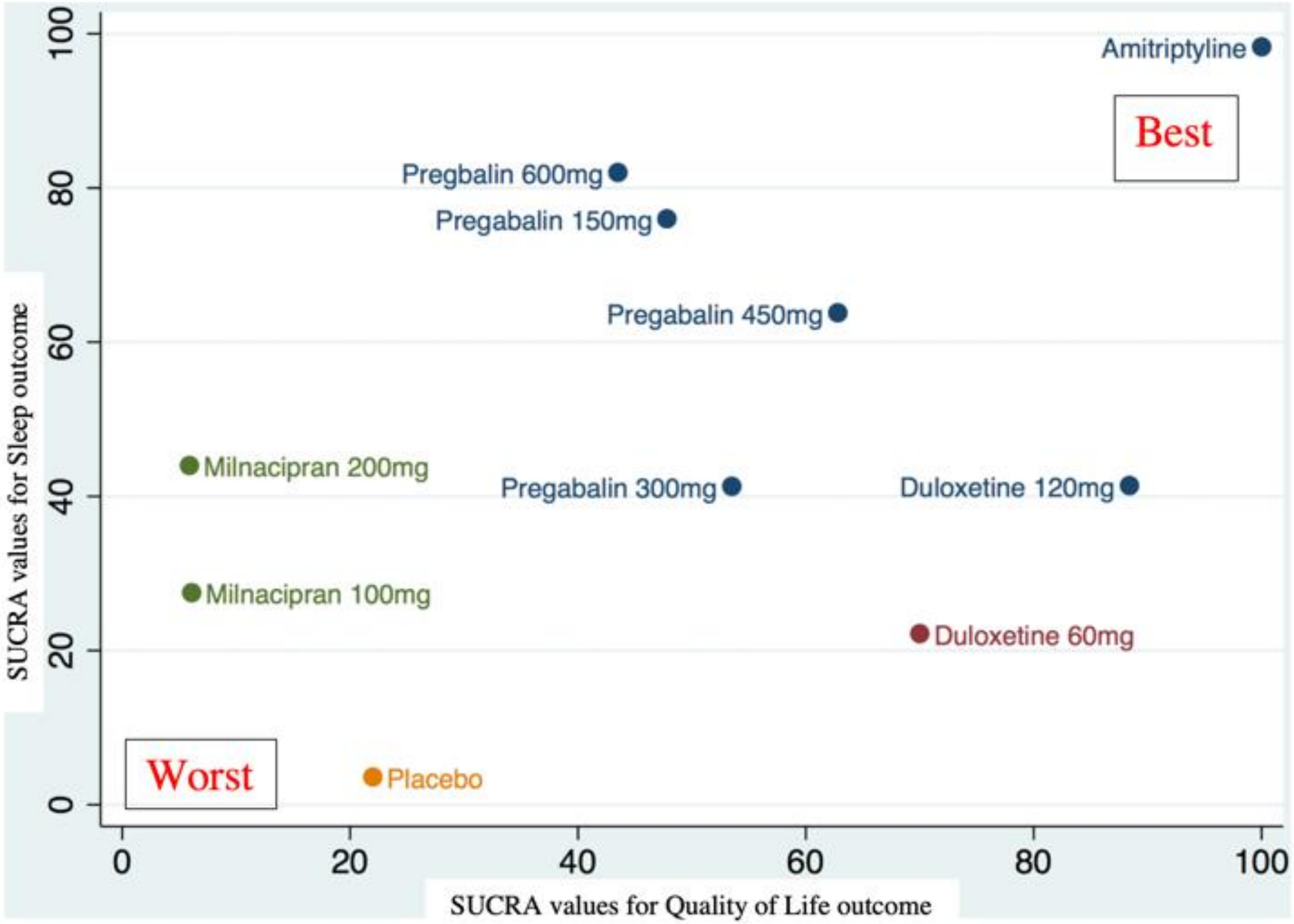

© 2022 Farag HM et al. *JAMA Network Open.*

eFigure 8H. Cluster ranking for quality of life (QoL) vs depression

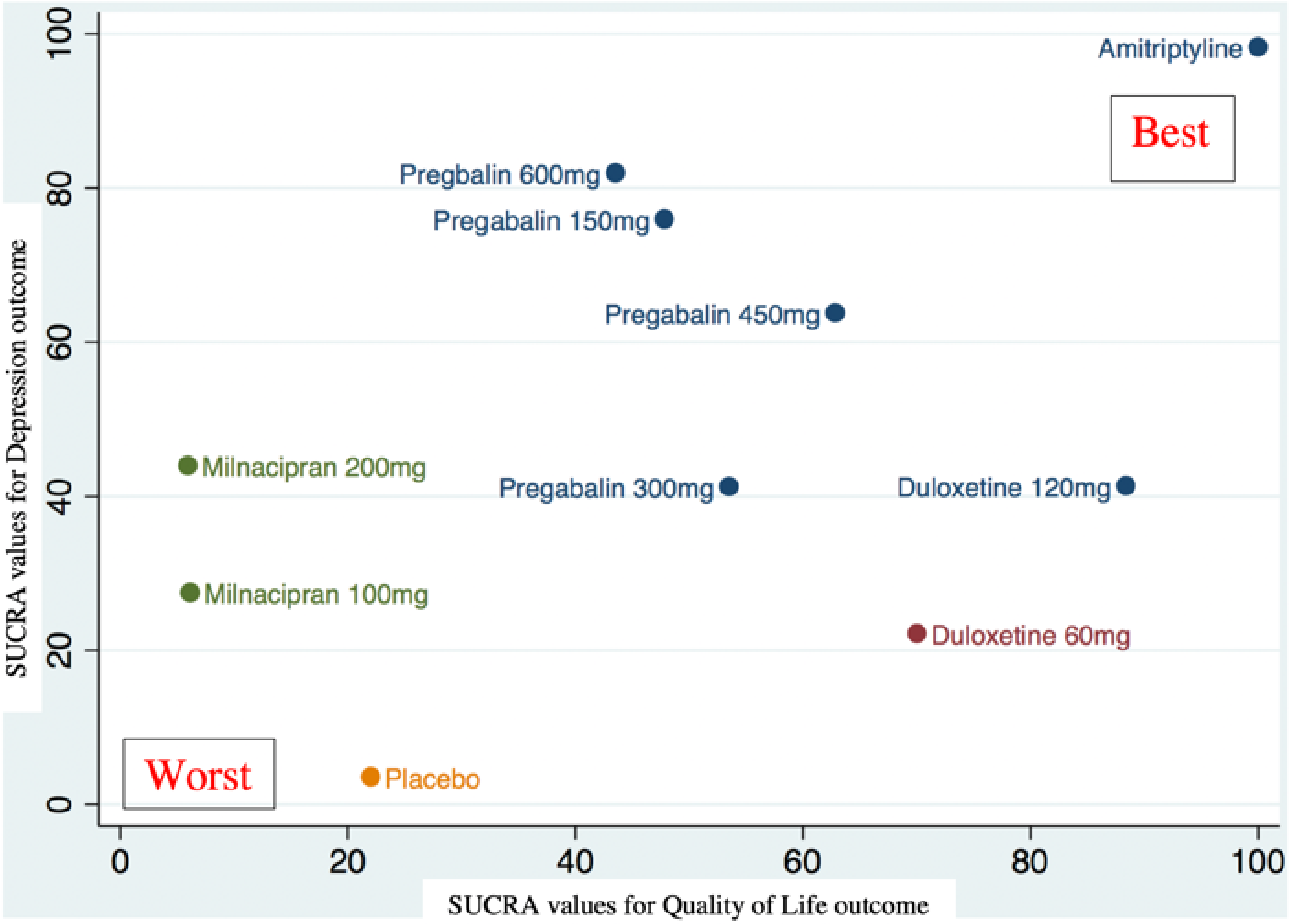

© 2022 Farag HM et al. *JAMA Network Open.*

eFigure 8I. Cluster ranking for fatigue vs quality of life (QoL)

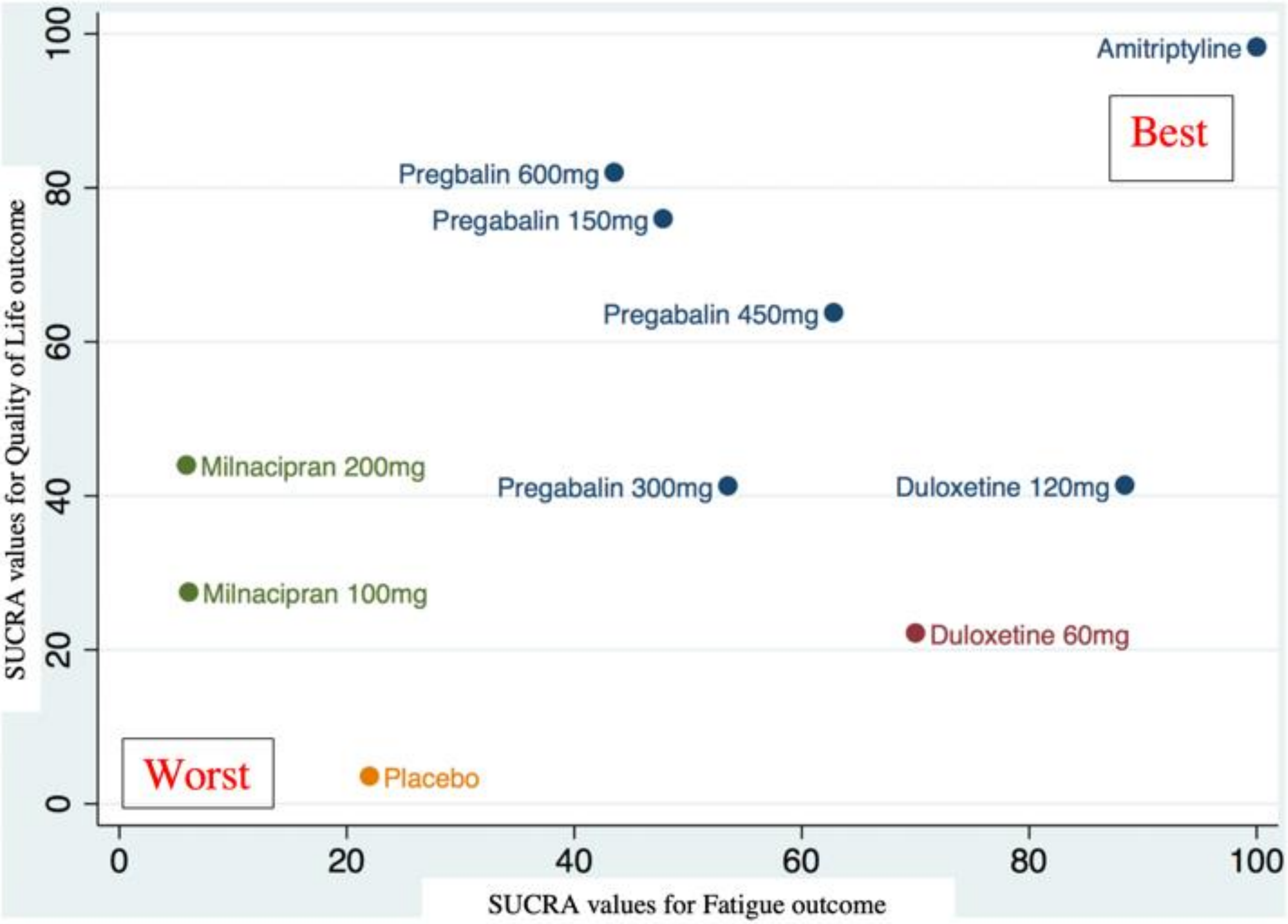

eFigure 9. Sensitivity Analysis Removing Studies With Small Sample Size for Each Outcome (Presented as Interval Plots)

© 2022 Farag HM et al. *JAMA Network Open.*

eFigure 9A. Interval plot for pain outcome when studies with a sample size of 100 or less were removed

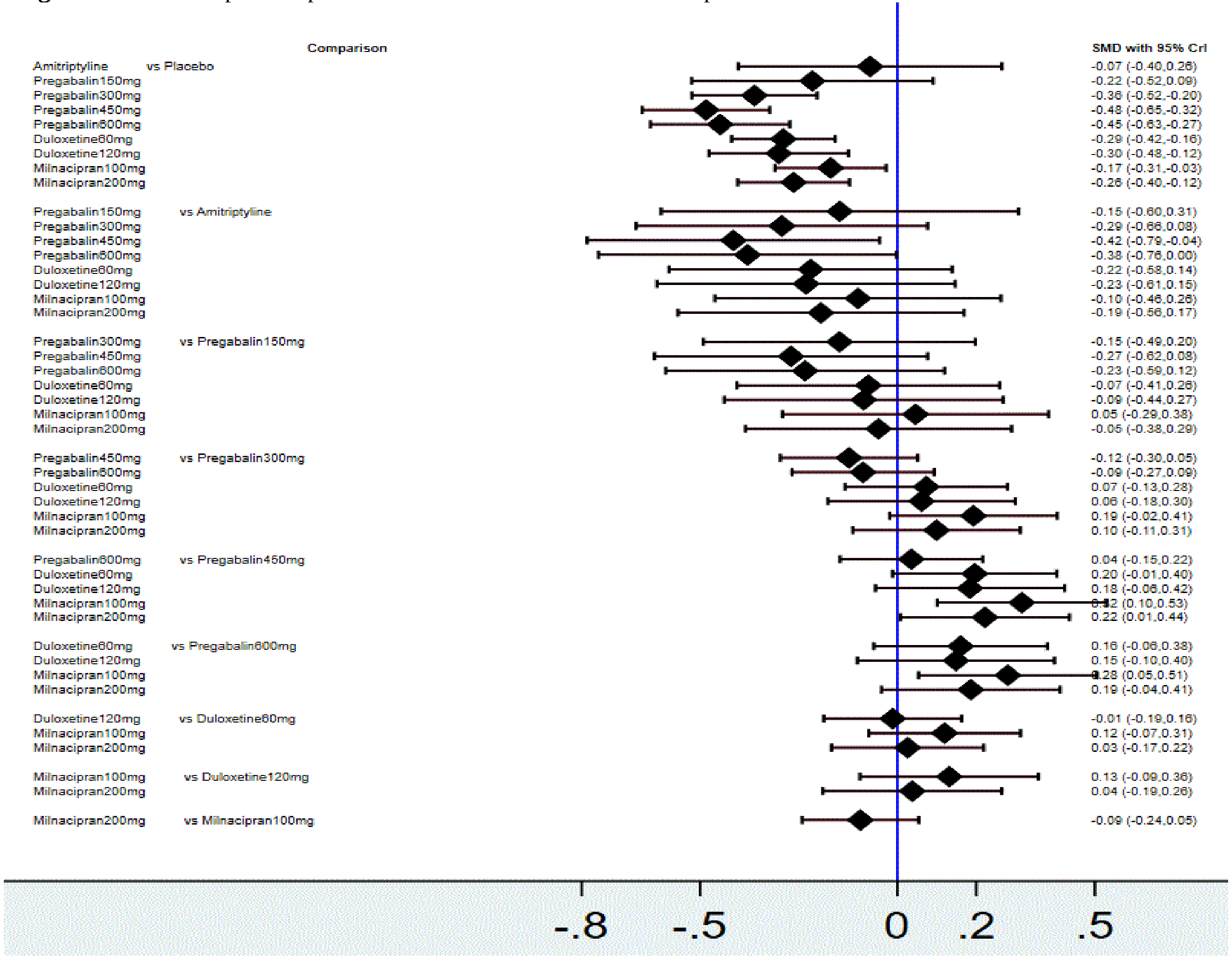

eFigure 9B. Interval plot for sleep outcome when studies with a sample size of 100 or less were removed

© 2022 Farag HM et al. *JAMA Network Open.*

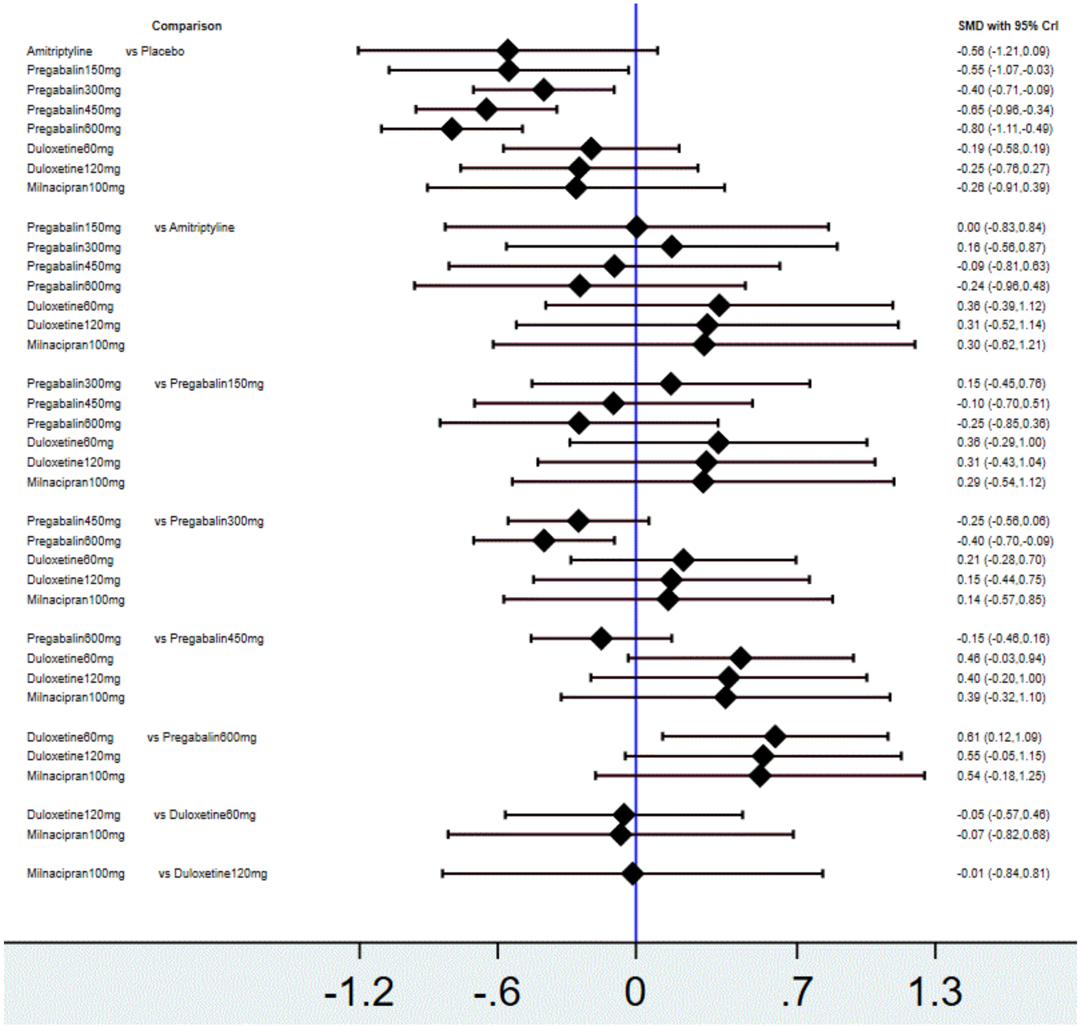

eFigure 9C. Interval plot for depression outcome when studies with a sample size of 100 or less were removed

© 2022 Farag HM et al. *JAMA Network Open.*

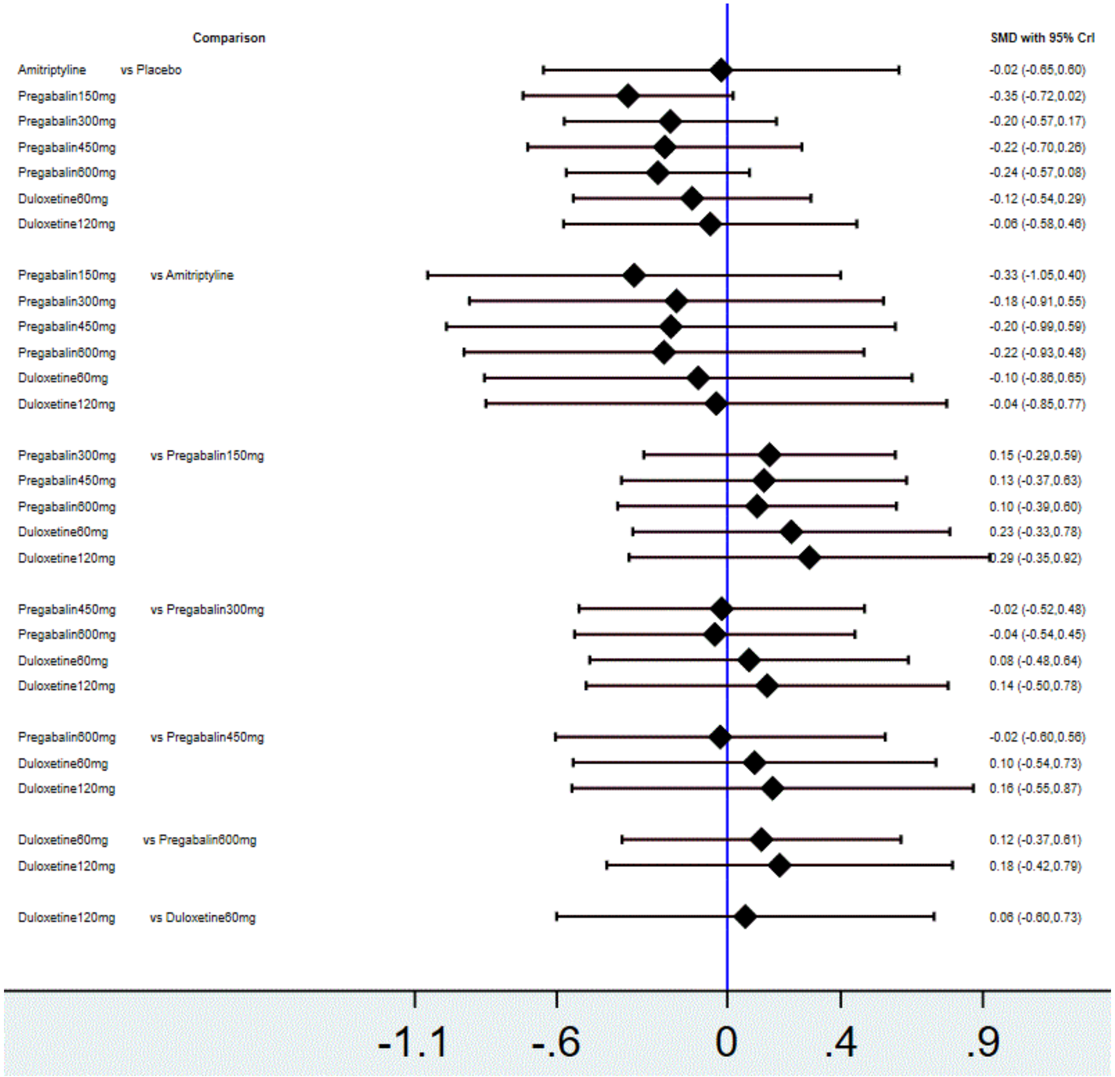

eFigure 9D. Interval plot for fatigue outcome when studies with a sample size of 100 or less were removed

© 2022 Farag HM et al. *JAMA Network Open.*

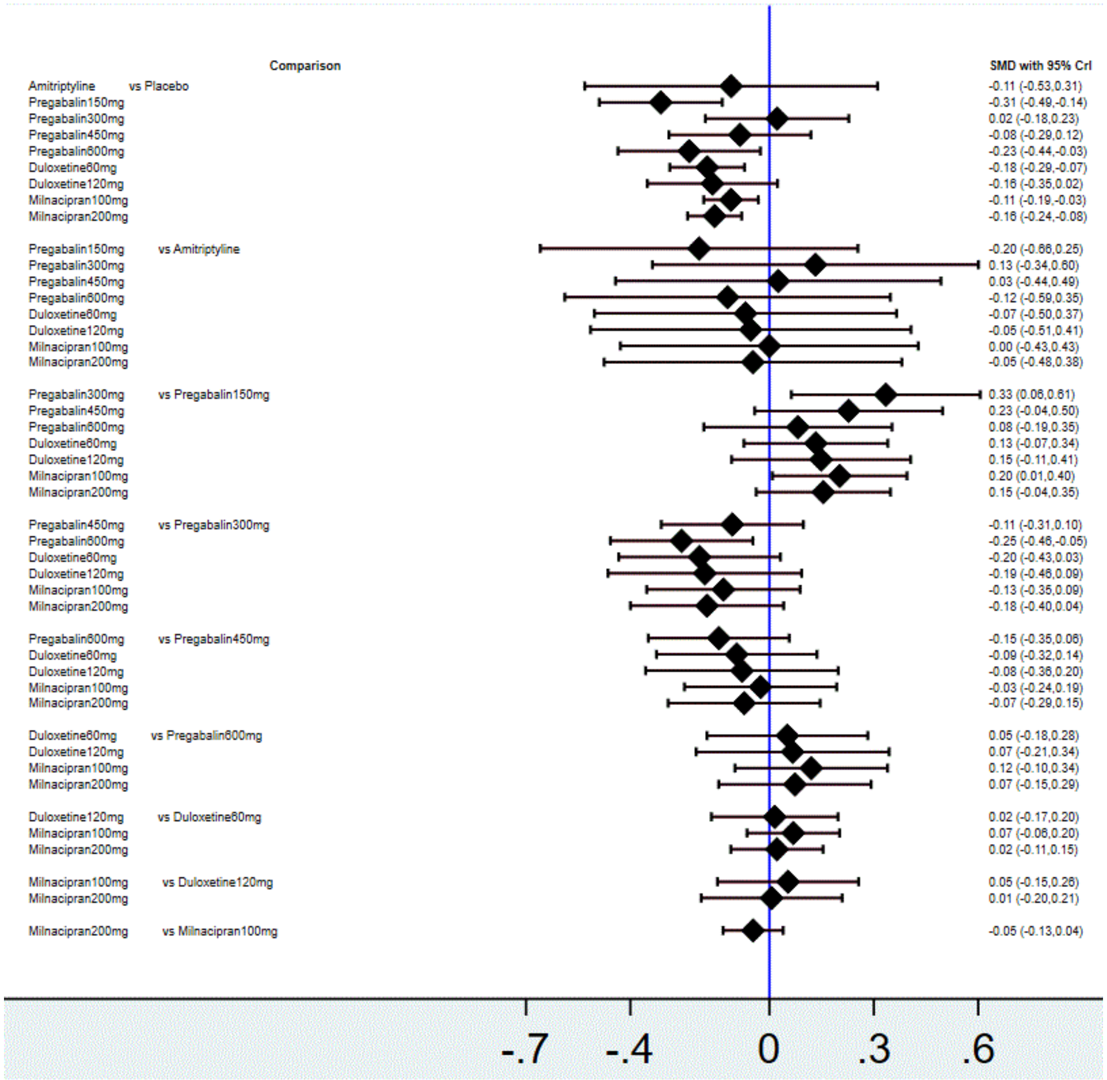

eFigure 9E. Interval plot for quality of life outcome when studies with a sample size of 100 or less were removed

© 2022 Farag HM et al. *JAMA Network Open.*

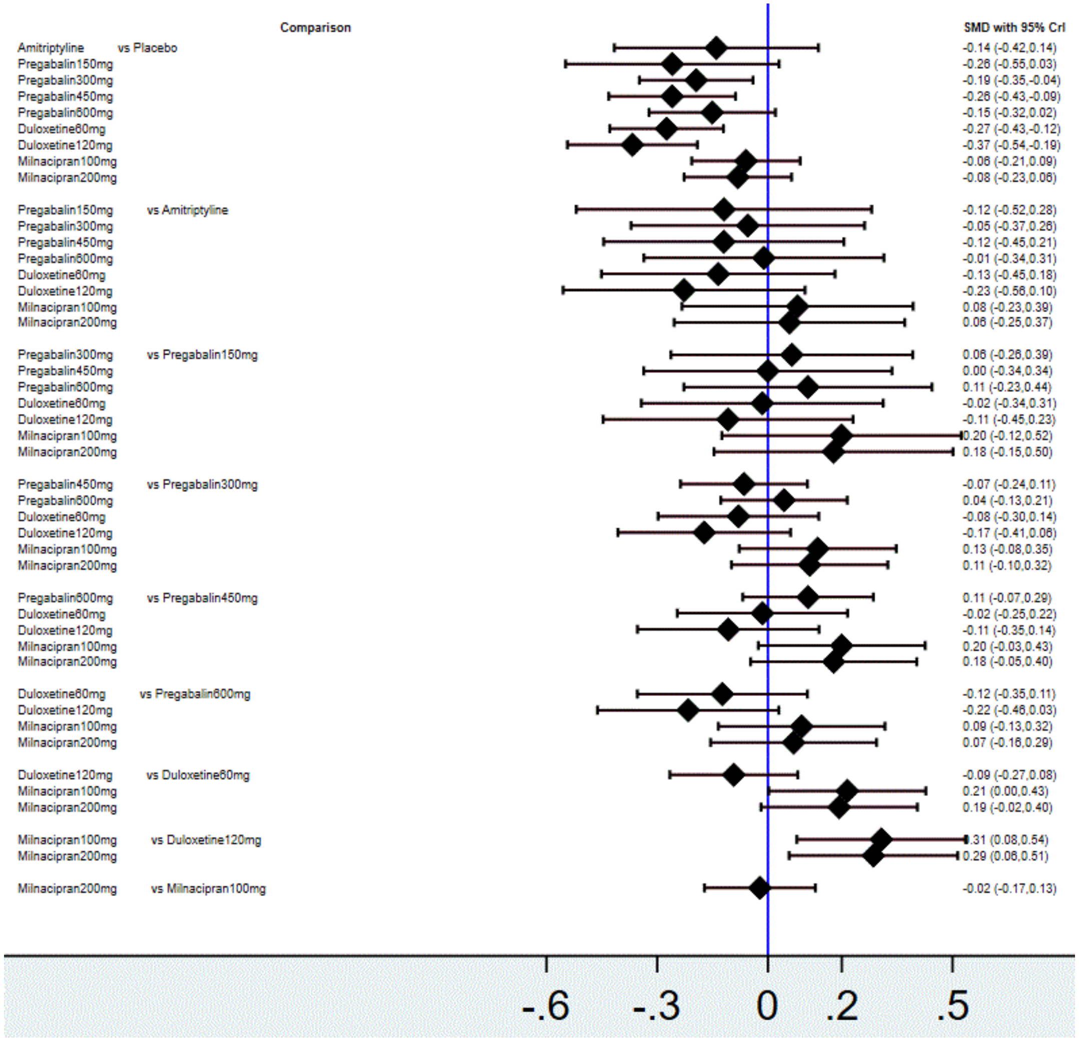

eTable 12. Sensitivity Analysis Removing Studies With Small Sample Size for Each Outcome (Presented as League Tables)

© 2022 Farag HM et al. *JAMA Network Open.*

eTable 12A. League table for pain outcome when studies with a sample size of 100 or less were removed

Milnacipran 200mg	0.09 (0.24,-0.05)	-0.04 (0.19,-0.26)	-0.03 (0.17,-0.22)	-0.19 (0.04,-0.41)	-0.22 (-0.01,-0.44)	-0.10 (0.11,-0.31)	0.05 (0.38,-0.29)	0.19 (0.56,-0.17)	0.26 (0.40,0.12)
-0.09 (-0.24,0.05)	Milnacipran 100mg	-0.13 (0.09,-0.36)	-0.12 (0.07,-0.31)	-0.28 (-0.05,-0.51)	-0.32 (-0.10,-0.53)	-0.19 (0.02,-0.41)	-0.05 (0.29,-0.38)	0.10 (0.46,-0.26)	0.17 (0.31,0.03)
0.04 (-0.19,0.26)	0.13 (-0.09,0.36)	Duloxetine 120mg	0.01 (0.19,-0.16)	-0.15 (0.10,-0.40)	-0.18 (0.06,-0.42)	-0.06 (0.18,-0.30)	0.09 (0.44,-0.27)	0.23 (0.61,-0.15)	0.30 (0.48,0.12)
0.03 (-0.17,0.22)	0.12 (-0.07,0.31)	-0.01 (-0.19,0.16)	Duloxetine 60mg	-0.16 (0.06,-0.38)	-0.20 (0.01,-0.40)	-0.07 (0.13,-0.28)	0.07 (0.41,-0.26)	0.22 (0.58,-0.14)	0.29 (0.42,0.16)
0.19 (-0.04,0.41)	**0.28 (0.05,0.51)**	0.15 (-0.10,0.40)	0.16 (-0.06,0.38)	Pregabalin 600mg	-0.04 (0.15,-0.22)	0.09 (0.27,-0.09)	0.23 (0.59,-0.12)	0.38 (0.76,0.00)	0.45 (0.63,0.27)
**0.22 (0.01,0.44)**	**0.32 (0.10,0.53)**	0.18 (-0.06,0.42)	0.20 (-0.01,0.40)	0.04 (-0.15,0.22)	Pregabalin 450mg	0.12 (0.30,-0.05)	0.27 (0.62,-0.08)	0.42 (0.79,0.04)	0.48 (0.65,0.32)
0.10 (-0.11,0.31)	0.19 (-0.02,0.41)	0.06 (-0.18,0.30)	0.07 (-0.13,0.28)	-0.09 (-0.27,0.09)	-0.12 (-0.30,0.05)	Pregabalin 300mg	0.15 (0.49,-0.20)	0.29 (0.66,-0.08)	0.36 (0.52,0.20)
-0.05 (-0.38,0.29)	0.05 (-0.29,0.38)	-0.09 (-0.44,0.27)	-0.07 (-0.41,0.26)	-0.23 (-0.59,0.12)	-0.27 (-0.62,0.08)	-0.15 (-0.49,0.20)	Pregabalin 150mg	0.15 (0.60,-0.31)	0.22 (0.52,-0.09)
-0.19 (-0.56,0.17)	-0.10 (-0.46,0.26)	-0.23 (-0.61,0.15)	-0.22 (-0.58,0.14)	-0.38 (-0.76,-0.00)	**-0.42 (-0.79,-0.04)**	-0.29 (-0.66,0.08)	-0.15 (-0.60,0.31)	Amitriptyline	0.07 (0.40,-0.26)
**-0.26 (-0.40,-0.12)**	**-0.17 (-0.31,-0.03)**	**-0.30 (-0.48,-0.12)**	**-0.29 (-0.42,-0.16)**	**-0.45 (-0.63,-0.27)**	**-0.48 (-0.65,-0.32)**	**-0.36 (-0.52,-0.20)**	-0.22 (-0.52,0.09)	-0.07 (-0.40,0.26)	Placebo

eTable 12B. League table for sleep outcome when studies with a sample size of 100 or less were removed

Placebo	-0.26 (-0.91,0.39)	-0.25 (-0.76,0.27)	-0.19 (-0.58,0.19)	-0.80 (-1.11,-0.49)	-0.65 (-0.96,-0.34)	-0.40 (-0.71,-0.09)	-0.55 (-1.07,-0.03)	-0.56 (-1.21,0.09)
0.26 (0.91,-0.39)	Milnacipran 100mg	0.01 (0.84,-0.81)	0.07 (0.82,-0.68)	-0.54 (0.18,-1.25)	-0.39 (0.32,-1.10)	-0.14 (0.57,-0.85)	-0.29 (0.54,-1.12)	-0.30 (0.62,-1.21)
0.25 (0.76,-0.27)	-0.01 (-0.84,0.81)	Duloxetine 120mg	0.05 (0.57,-0.46)	-0.55 (0.05,-1.15)	-0.40 (0.20,-1.00)	-0.15 (0.44,-0.75)	-0.31 (0.43,-1.04)	-0.31 (0.52,-1.14)
0.19 (0.58,-0.19)	-0.07 (-0.82,0.68)	-0.05 (-0.57,0.46)	Duloxetine 60mg	-0.61 (-0.12,-1.09)	-0.46 (0.03,-0.94)	-0.21 (0.28,-0.70)	-0.36 (0.29,-1.00)	-0.36 (0.39,-1.12)
**0.80 (1.11,0.49)**	0.54 (-0.18,1.25)	0.55 (-0.05,1.15)	**0.61 (0.12,1.09)**	Pregabalin 600mg	0.15 (0.46,-0.16)	0.40 (0.70,0.09)	0.25 (0.85,-0.36)	0.24 (0.96,-0.48)
**0.65 (0.96,0.34)**	0.39 (-0.32,1.10)	0.40 (-0.20,1.00)	0.46 (-0.03,0.94)	-0.15 (-0.46,0.16)	Pregabalin 450mg	0.25 (0.56,-0.06)	0.10 (0.70,-0.51)	0.09 (0.81,-0.63)
**0.40 (0.71,0.09)**	0.14 (-0.57,0.85)	0.15 (-0.44,0.75)	0.21 (-0.28,0.70)	**-0.40 (-0.70,-0.09)**	-0.25 (-0.56,0.06)	Pregabalin 300mg	-0.15 (0.45,-0.76)	-0.16 (0.56,-0.87)
**0.55 (1.07,0.03)**	0.29 (-0.54,1.12)	0.31 (-0.43,1.04)	0.36 (-0.29,1.00)	-0.25 (-0.85,0.36)	-0.10 (-0.70,0.51)	0.15 (-0.45,0.76)	Pregabalin 150mg	-0.00 (0.83,-0.84)
0.56 (1.21,-0.09)	0.30 (-0.62,1.21)	0.31 (-0.52,1.14)	0.36 (-0.39,1.12)	-0.24 (-0.96,0.48)	-0.09 (-0.81,0.63)	0.16 (-0.56,0.87)	0.00 (-0.83,0.84)	Amitriptyline

eTable 12C. League table for depression outcome when studies with a sample size of 100 or less were removed

© 2022 Farag HM et al. *JAMA Network Open.*

Placebo	-0.02 (-0.65,0.60)	-0.06 (-0.58,0.46)	-0.12 (-0.54,0.29)	-0.24 (-0.57,0.08)	-0.22 (-0.70,0.26)	-0.20 (-0.57,0.17)	-0.35 (-0.72,0.02)
0.02 (0.65,-0.60)	Amitriptyline	-0.04 (-0.85,0.77)	-0.10 (-0.86,0.65)	-0.22 (-0.93,0.48)	-0.20 (-0.99,0.59)	-0.18 (-0.91,0.55)	-0.33 (-1.05,0.40)
0.06 (0.58,-0.46)	0.04 (0.85,-0.77)	Duloxetine 120mg	-0.06 (0.60,-0.73)	-0.18 (0.42,-0.79)	-0.16 (0.55,-0.87)	-0.14 (0.50,-0.78)	-0.29 (0.35,-0.92)
0.12 (0.54,-0.29)	0.10 (0.86,-0.65)	0.06 (-0.60,0.73)	Duloxetine 60mg	-0.12 (0.37,-0.61)	-0.10 (0.54,-0.73)	-0.08 (0.48,-0.64)	-0.23 (0.33,-0.78)
0.24 (0.57,-0.08)	0.22 (0.93,-0.48)	0.18 (-0.42,0.79)	0.12 (-0.37,0.61)	Pregabalin 600mg	0.02 (0.60,-0.56)	0.04 (0.54,-0.45)	-0.10 (0.39,-0.60)
0.22 (0.70,-0.26)	0.20 (0.99,-0.59)	0.16 (-0.55,0.87)	0.10 (-0.54,0.73)	-0.02 (-0.60,0.56)	Pregabalin 450mg	0.02 (0.52,-0.48)	-0.13 (0.37,-0.63)
0.20 (0.57,-0.17)	0.18 (0.91,-0.55)	0.14 (-0.50,0.78)	0.08 (-0.48,0.64)	-0.04 (-0.54,0.45)	-0.02 (-0.52,0.48)	Pregabalin 300mg	-0.15 (0.29,-0.59)
0.35 (0.72,-0.02)	0.33 (1.05,-0.40)	0.29 (-0.35,0.92)	0.23 (-0.33,0.78)	0.10 (-0.39,0.60)	0.13 (-0.37,0.63)	0.15 (-0.29,0.59)	Pregabalin 150mg

eTable 12D. League table for fatigue outcome when studies with a sample size of 100 or less were removed

Milnacipran 200mg	0.05 (0.13,-0.04)	-0.01 (0.20,-0.21)	-0.02 (0.11,-0.15)	-0.07 (0.15,-0.29)	0.07 (0.29,-0.15)	0.18 (0.40,-0.04)	-0.15 (0.04,-0.35)	0.05 (0.48,-0.38)	0.16 (0.24,0.08
-0.05 (-0.13,0.04)	Milnacipran 100mg	-0.05 (0.15,-0.26)	-0.07 (0.06,-0.20)	-0.12 (0.10,-0.34)	0.03 (0.24,-0.19)	0.13 (0.35,-0.09)	-0.20 (-0.01,-0.40)	0.00 (0.43,-0.43)	0.11 (0.19,0.03
0.01 (-0.20,0.21)	0.05 (-0.15,0.26)	Duloxetine 120mg	-0.02 (0.17,-0.20)	-0.07 (0.21,-0.34)	0.08 (0.36,-0.20)	0.19 (0.46,-0.09)	-0.15 (0.11,-0.41)	0.05 (0.51,-0.41)	0.16 (0.35,-0.02
0.02 (-0.11,0.15)	0.07 (-0.06,0.20)	0.02 (-0.17,0.20)	Duloxetine 60mg	-0.05 (0.18,-0.28)	0.09 (0.32,-0.14)	0.20 (0.43,-0.03)	-0.13 (0.07,-0.34)	0.07 (0.50,-0.37)	0.18 (0.29,0.07
0.07 (-0.15,0.29)	0.12 (-0.10,0.34)	0.07 (-0.21,0.34)	0.05 (-0.18,0.28)	Pregabalin 600mg	0.15 (0.35,-0.06)	0.25 (0.46,0.05)	-0.08 (0.19,-0.35)	0.12 (0.59,-0.35)	0.23 (0.44,0.03
-0.07 (-0.29,0.15)	-0.03 (-0.24,0.19)	-0.08 (-0.36,0.20)	-0.09 (-0.32,0.14)	-0.15 (-0.35,0.06)	Pregabalin 450mg	0.11 (0.31,-0.10)	-0.23 (0.04,-0.50)	-0.03 (0.44,-0.49)	0.08 (0.29,-0.12
-0.18 (-0.40,0.04)	-0.13 (-0.35,0.09)	-0.19 (-0.46,0.09)	-0.20 (-0.43,0.03)	-0.25 (-0.46,-0.05)	-0.11 (-0.31,0.10)	Pregabalin 300mg	-0.33 (-0.06,-0.61)	-0.13 (0.34,-0.60)	-0.02 (0.18,-0.2
0.15 (-0.04,0.35)	**0.20 (0.01,0.40)**	0.15 (-0.11,0.41)	0.13 (-0.07,0.34)	0.08 (-0.19,0.35)	0.23 (-0.04,0.50)	0.33 (0.06,0.61)	Pregabalin 150mg	0.20 (0.66,-0.25)	0.31 (0.49,0.14
-0.05 (-0.48,0.38)	-0.00 (-0.43,0.43)	-0.05 (-0.51,0.41)	-0.07 (-0.50,0.37)	-0.12 (-0.59,0.35)	0.03 (-0.44,0.49)	0.13 (-0.34,0.60)	-0.20 (-0.66,0.25)	Amitriptyline	0.11 (0.53,-0.31
**-0.16 (-0.24,-0.08)**	**-0.11 (-0.19,-0.03)**	-0.16 (-0.35,0.02)	**-0.18 (-0.29,-0.07)**	**-0.23 (-0.44,-0.03)**	-0.08 (-0.29,0.12)	0.02 (-0.18,0.23)	**-0.31 (-0.49,-0.14)**	-0.11 (-0.53,0.31)	Placebo

eTable 12E. League table for quality of life outcome when studies with a sample size of 100 or less were removed

Milnacipran 200mg	0.02 (0.17,-0.13)	-0.29 (-0.06,-0.51)	-0.19 (0.02,-0.40)	-0.07 (0.16,-0.29)	-0.18 (0.05,-0.40)	-0.11 (0.10,-0.32)	-0.18 (0.15,-0.50)	-0.06 (0.25,-0.37)	0.08 (0.23,-0.06)
-0.02 (-0.17,0.13)	Milnacipran 100mg	-0.31 (-0.08,-0.54)	-0.21 (-0.00,-0.43)	-0.09 (0.13,-0.32)	-0.20 (0.03,-0.43)	-0.13 (0.08,-0.35)	-0.20 (0.12,-0.52)	-0.08 (0.23,-0.39)	0.06 (0.21,-0.09)
**0.29 (0.06,0.51)**	**0.31 (0.08,0.54)**	Duloxetine 120mg	0.09 (0.27,-0.08)	0.22 (0.46,-0.03)	0.11 (0.35,-0.14)	0.17 (0.41,-0.06)	0.11 (0.45,-0.23)	0.23 (0.56,-0.10)	0.37 (0.54,0.19)
0.19 (-0.02,0.40)	0.21 (0.00,0.43)	-0.09 (-0.27,0.08)	Duloxetine 60mg	0.12 (0.35,-0.11)	0.02 (0.25,-0.22)	0.08 (0.30,-0.14)	0.02 (0.34,-0.31)	0.13 (0.45,-0.18)	0.27 (0.43,0.12)
0.07 (-0.16,0.29)	0.09 (-0.13,0.32)	-0.22 (-0.46,0.03)	-0.12 (-0.35,0.11)	Pregabalin 600mg	-0.11 (0.07,-0.29)	-0.04 (0.13,-0.21)	-0.11 (0.23,-0.44)	0.01 (0.34,-0.31)	0.15 (0.32,-0.02)
0.18 (-0.05,0.40)	0.20 (-0.03,0.43)	-0.11 (-0.35,0.14)	-0.02 (-0.25,0.22)	0.11 (-0.07,0.29)	Pregabalin 450mg	0.07 (0.24,-0.11)	0.00 (0.34,-0.34)	0.12 (0.45,-0.21)	0.26 (0.43,0.09)
0.11 (-0.10,0.32)	0.13 (-0.08,0.35)	-0.17 (-0.41,0.06)	-0.08 (-0.30,0.14)	0.04 (-0.13,0.21)	-0.07 (-0.24,0.11)	Pregabalin 300mg	-0.06 (0.26,-0.39)	0.05 (0.37,-0.26)	0.19 (0.35,0.04)
0.18 (-0.15,0.50)	0.20 (-0.12,0.52)	-0.11 (-0.45,0.23)	-0.02 (-0.34,0.31)	0.11 (-0.23,0.44)	-0.00 (-0.34,0.34)	0.06 (-0.26,0.39)	Pregabalin 150mg	0.12 (0.52,-0.28)	0.26 (0.55,-0.03)
0.06 (-0.25,0.37)	0.08 (-0.23,0.39)	-0.23 (-0.56,0.10)	-0.13 (-0.45,0.18)	-0.01 (-0.34,0.31)	-0.12 (-0.45,0.21)	-0.05 (-0.37,0.26)	-0.12 (-0.52,0.28)	Amitriptyline	0.14 (0.42,-0.14)
-0.08 (-0.23,0.06)	-0.06 (-0.21,0.09)	**-0.37 (-0.54,-0.19)**	**-0.27 (-0.43,-0.12)**	-0.15 (-0.32,0.02)	**-0.26 (-0.43,-0.09)**	**-0.19 (-0.35,-0.04)**	-0.26 (-0.55,0.03)	-0.14 (-0.42,0.14)	Placebo

© 2022 Farag HM et al. *JAMA Network Open.*
